# Universal Battery Capacity Degradation Forecasting Driven by Foundation Models Across Diverse Chemistries and Conditions

**DOI:** 10.1002/advs.77261

**Published:** 2026-08-24

**Authors:** Joey Chan, Huan Wang, Zhen Chen, Haoyu Pan, Wei Wu, Zirong Wang, Ershun Pan, Min Xie, Lifeng Xi

**Affiliations:** ^1^ Department of Industrial Engineering and Management Shanghai Jiaotong University Shanghai China; ^2^ Department of Systems Engineering City University of Hong Kong Hong Kong China

**Keywords:** adaptation, computer science, energy management, energy storage, fade, feature learning, scalability, unified model

## Abstract

Accurate forecasting of battery capacity fade is essential for the safety, reliability, and long‐term efficiency of energy storage systems. However, the heterogeneity across cell chemistries, form factors, and operating conditions makes it difficult to build a single model that generalizes beyond its training domain. This work proposes a unified capacity forecasting framework that maintains robust performance across diverse chemistries and usage scenarios. We curate 20 public aging datasets into a large‐scale corpus covering 1704 cells and 3 961 195 charge–discharge cycle segments, spanning temperatures from $-5^{\circ }C$ to $45^{\circ }C$, multiple C‐rates, and application‐oriented profiles such as fast charging and partial cycling. On this corpus, we adopt a Time‐Series Foundation Model (TSFM) backbone and apply parameter‐efficient Low‐Rank Adaptation (LoRA) together with physics‐guided contrastive representation learning to capture shared degradation patterns. Experiments on both “known” and held‐out “unknown” datasets show that a single unified model achieves competitive or superior accuracy compared with recent state‐of‐the‐art time‐series forecasting methods trained separately for each dataset, while retaining stable performance on chemistries, capacity scales, and operating conditions excluded from training. These results demonstrate the potential of TSFM‐based architectures as a scalable and transferable solution for capacity degradation forecasting in battery management systems.

## Graphical abstract

A unified time‐series forecasting framework learns transferable battery capacity‐degradation patterns from 20 heterogeneous datasets spanning chemistries, formats, temperatures, and cycling conditions. A single model delivers competitive predictions on both known and previously unseen datasets, while physics‐guided representation learning improves robustness without requiring physical inputs at inference. The results point toward scalable cross‐domain battery health forecasting.

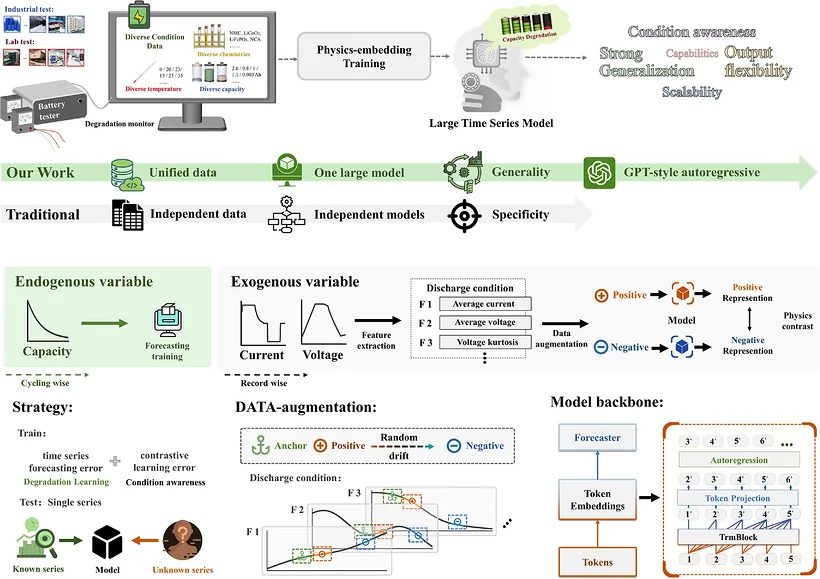

## 1 Introduction

Lithium‐ion batteries have become a central pillar of modern energy infrastructures, supporting applications that range from electric vehicles (EVs) and portable electronics to grid‐scale storage, frequency regulation, and distributed renewable integration. In 2023, global EV sales exceeded 14 million units, corresponding to a battery demand of more than 1 TWh and a projected global demand of 3–6 TWh by 2030 [1, 2, 3]. The global battery market was valued at approximately USD 134.6 billion in 2024 and is expected to reach USD 329.8 billion by 2030, with a compound annual growth rate of about 16.4% [4]. In parallel with this rapid expansion, the landscape of battery chemistries is becoming increasingly diverse, including lithium iron phosphate (LFP), nickel cobalt manganese/aluminum (NCM/NCA), sodium‐ion, solid‐state chemistries, and hybrid systems, deployed across cylindrical, pouch, prismatic, and coin formats with widely varying energy densities, thermal stability, and charging characteristics [5, 6]. For instance, the market share of LFP has risen sharply from about 6% in 2020 to roughly 30% in 2022, while NCM/NCA systems continue to dominate long‐range EV applications due to their high specific energy [7]. This technological diversification places stringent new demands on battery management systems (BMSs), especially on capacity degradation models that must operate reliably across chemistry families, form factors, and operating regimes [8, 9].

Accurate forecasting of capacity degradation is a key enabler for safe, reliable, and cost‐effective operation over the entire lifetime of energy storage assets [10, 11, 12]. However, capacity fade is simultaneously governed by chemistry‐specific reactions, electrode–electrolyte interfaces, cell design, and application‐dependent load patterns, which together give rise to highly heterogeneous temporal behaviors across use cases [13, 14, 15, 16]. Batteries deployed in EVs, grid‐scale BESS, and consumer electronics experience fundamentally different cycling depths, current profiles, temperature environments, and calendar aging effects, often extending far beyond the controlled conditions of laboratory tests [17, 18]. The gap between laboratory protocols and real‐world duty cycles means that models tuned for specific datasets, chemistries, or temperature ranges can suffer severe performance degradation when transferred to unknown devices or operating conditions [19]. This problem is further exacerbated by the emergence of new chemistries such as sodium‐ion [20, 21] and mixed‐chemistry systems [22, 23], whose interplay with complex operating conditions further complicates capacity degradation forecasting. As a result, the central challenge is no longer merely to improve accuracy in a single, well‐controlled scenario, but to build a capacity forecasting framework that can generalize across chemistries, structures, and usage conditions—and remain robust when encountering previously unknown regimes.

Hybrid modeling approaches that combine physics‐guided indicators with data‐driven techniques have shown promise in improving interpretability and prediction accuracy [24, 25]. Nevertheless, their generalization often remains tightly coupled to hand‐crafted features and task‐specific architectures [26]. When the target deviates from the training domain–for example, when the chemistry changes from LCO to NMC, the temperature range shifts, or the duty cycle becomes more dynamic–these models still tend to exhibit noticeable performance drops [18, 27, 28]. From a broader perspective, capacity forecasting is fundamentally a time‐series prediction problem: given a historical trajectory of capacity (and possibly auxiliary signals), the task is to infer its future evolution [29, 30, 31]. This formulation aligns closely with other sequence modeling problems such as language modeling, where future tokens are generated conditionally on past context. This conceptual similarity suggests that the same architectural tools that have transformed natural language processing–most notably Transformer‐based models–may provide a powerful and more universal modeling paradigm for battery capacity degradation.

Transformer architectures [32], originally developed for large language models (LLMs), have recently been adapted to time‐series tasks by replacing discrete token embeddings with continuous numerical segments and redefining the training objective from next‐token prediction to multi‐horizon forecasting [33]. Time‐Series Foundation Models (TSFMs) push this idea further by pretraining large, attention‐based models on diverse temporal datasets so that they learn general‐purpose temporal priors that can later be adapted to a variety of downstream tasks. In principle, such foundation models could provide a unified backbone for battery capacity forecasting: instead of designing bespoke models for each dataset or chemistry, one could leverage a pre‐trained TSFM and specialize it to degradation trajectories via lightweight adaptation. This raises an important research question: can a TSFM‐based framework, suitably adapted to battery domain characteristics, break the long‐standing generalization bottlenecks across chemistries, form factors, and operational conditions?

Building on this understanding, this study proposes a unified battery capacity forecasting framework that operates across multiple chemistries and operating conditions. We first systematically integrate and standardize 20 publicly available battery aging datasets to construct a large‐scale corpus of capacity degradation time series comprising 1704 cells, covering both lithium‐ion and sodium‐ion systems, including LCO, NCM, and NCA cathode chemistries as well as cylindrical, pouch, prismatic, and coin cell formats. This corpus spans a wide temperature range from 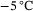 to 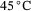 and includes complex operating regimes such as fast charging, partial cycling, and dynamic load profiles, thereby providing a solid data foundation for unified modeling.

On this basis, we adopt a time‐series foundation model as the forecasting backbone and develop a unified representation of capacity degradation dynamics through parameter‐efficient adaptation and physics‐guided representation learning. Experimental results show that the proposed method achieves competitive capacity‐forecasting performance across a range of heterogeneous scenarios, while retaining reasonable predictive performance on battery chemistries and operating conditions that are unknown during training. These findings indicate that our approach offers a potentially scalable and transferable framework for battery capacity degradation forecasting and provides empirical support for further exploring time‐series foundation models in battery health prediction under previously unseen operating conditions.

## 2 Results

### 2.1 Framework Overview

We propose a unified modeling framework for battery capacity degradation forecasting (Figure 1), designed to overcome the performance bottlenecks of traditional data‐driven methods under diverse physico‐chemical operating conditions. By incorporating large‐scale time‐series modeling techniques, the framework enables generalized degradation prediction across various battery chemistries, packaging formats, and application scenarios.

**FIGURE 1:**
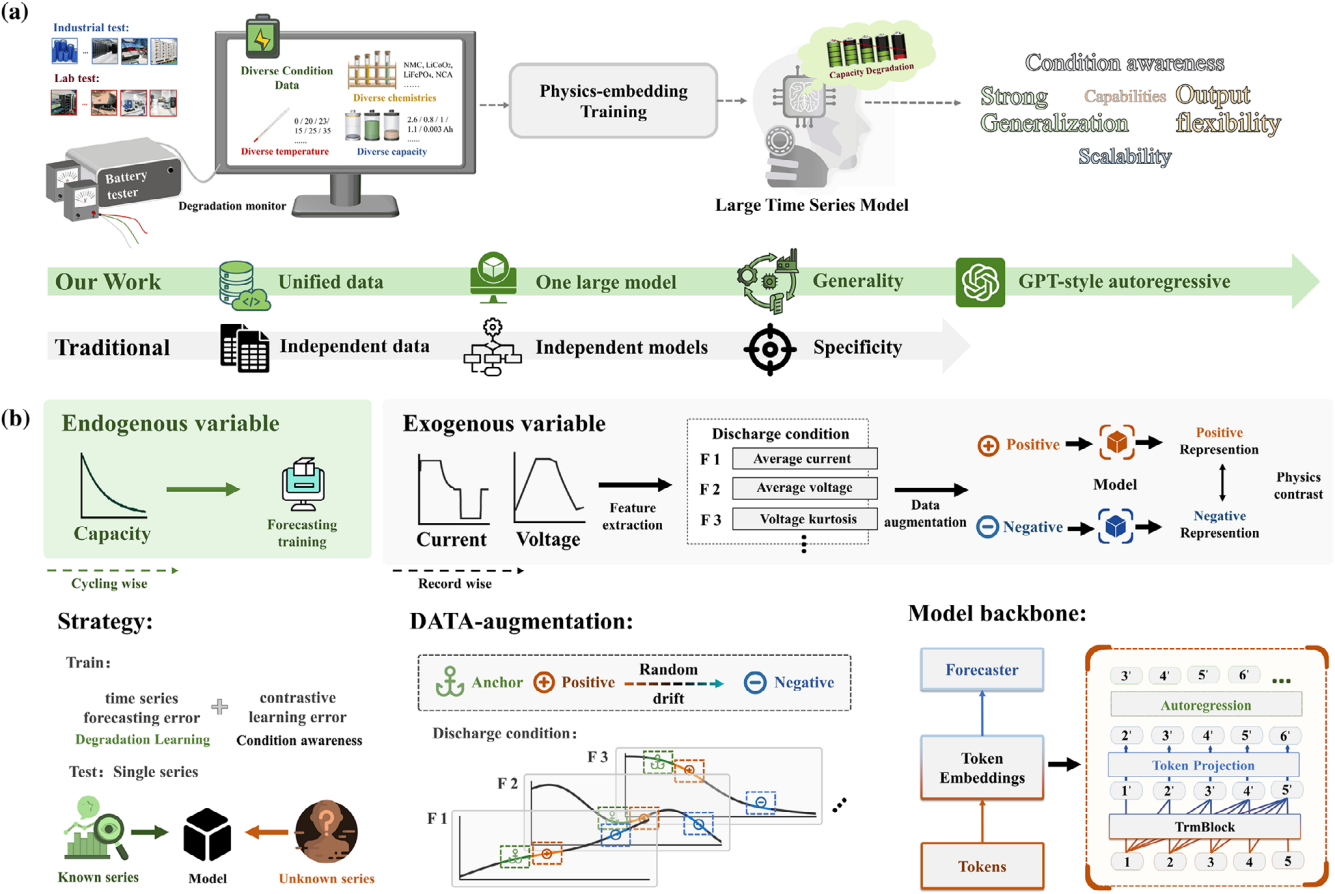
The flowchart of the large‐model‐driven battery capacity forecasting. (a) Unlike conventional, task‐specific capacity degradation models, our approach constructs a rich, standardized corpus of degradation trajectories and employs a single time‐series foundation model to forecast capacity under previously unknown conditions. (b) In the physics‐guided training strategy, the endogenous variable (capacity) and exogenous variables (operating conditions) are treated differently: charge–discharge features are incorporated as auxiliary physical embeddings during training to exploit domain knowledge, while the model remains independent of these potentially missing physical inputs at inference time.

Figure 1a illustrates the motivation and overall workflow of the proposed approach. To construct a time‐series corpus of battery cycle–capacity degradation trajectories for TSFM training, we aggregate 20 datasets collected from both laboratory experiments and production environments. These datasets cover a wide range of chemistries, test temperatures, cell formats, and nominal capacities. Using the proposed physics‐guided training strategy, this rich degradation corpus exposes the TSFM to sufficiently diverse operating scenarios. Our aim is that, given adequate data, Transformer‐based capacity forecasting can exhibit a level of universality, generalization, and scalability analogous to that of LLMs in text generation.

Figure 1b provides a more detailed view of the procedure. The upper row illustrates the separate processing pipelines for the endogenous cycle‐wise discharge‐capacity sequence and the exogenous physical‐feature sequence. The exogenous variables consist of cycle‐level statistical descriptors extracted from discharge current, terminal voltage, and discharge duration. The lower row summarizes the forecasting objective, the contrastive‐learning strategy and its time‐series augmentation procedure, and the shared Timer‐based model architecture. In our formulation, the sequence of capacity values evolving with cycle index is treated as the endogenous variable. In parallel, the training data record the charge–discharge behavior within each cycle; from these profiles we extract representative physics‐based features, which serve as auxiliary exogenous variables. At the model level, we adopt a hybrid objective. On the one hand, the capacity sequence is used as the primary target and is fed into the Timer architecture for supervised learning. On the other hand, the multidimensional physical features are embedded as auxiliary vectors and optimized via a contrastive learning objective. Through data augmentation, this strategy constructs positive and negative sample pairs, encouraging the model to automatically discriminate degradation patterns under different operating conditions and to develop a self‐supervised representation of the underlying physical states. Importantly, the exogenous variables are only used during training; no physical inputs are required at inference, thereby preserving the flexibility and applicability of the model in real‐world deployment. Additional methodological details are provided in the Methods section.

### 2.2 Data Generation

To train the proposed large time‐series model for capacity degradation forecasting, we first construct a unified corpus of cycle–capacity trajectories, as illustrated in Figure 2a. Specifically, we consolidate 20 publicly available battery aging datasets into a single mixed‐chemistry corpus comprising 1,704 cells and a total of 3 961 195 recorded cycles (Figure S1) For each cell, we extract the sequence of cycle indices and the corresponding discharge capacity, yielding standardized cycle–capacity degradation histories that can be directly used for TSFM training. A detailed summary of the datasets is provided in Table 1. The capacity range is reported as the 1st–99th percentile range of quality‐controlled cycle‐level discharge capacities in each processed dataset.

**TABLE 1:** Battery datasets grouped by material system.

Dataset	Cathode	Anode	Cell Num	Cycle Num	Capacity (Ah)	Capacity Range (Ah)	Temperature ( 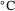 )	Format
**NMC family**								
RWTH [34]	NMC	Carbon	46	100 423	3.00	0.23–1.06	25	18650 cyl.
ISU_ILCC [35]	NMC	Graphite	240	1 194 846	0.25	3.69$\times 10^{-3}$–0.25	30	502030 pouch
MICH_EXP [36]	NMC111	Graphite	18	6545	5.00	1.37–4.75	−5 / 25 / 45	Pouch
MICH [36]	NMC111	Graphite	40	19 895	2.36	0.16–2.36	25 / 45	Pouch
Stanford_2 [37]	NMC532	Artificial Graphite	181	188 621	0.25	0.02–0.25	30	18650 cyl.
Stanford [37]	NMC532	Artificial Graphite	41	39 329	0.24	0.04–0.25	30	18650 cyl.
XJTU [37]	NMC532	Graphite	23	6938	2.00	1.62–1.98	20	18650 cyl.
EOCV2 [38]	NMC	Carbon–SiO	228	1 286 612	3.00	1.37–2.82	0 / 10 / 25 / 40	18650 cyl.
**LCO family**								
CALCE [39]	LCO	Graphite	13	14 299	1.10	0.29–1.32	25	Prismatic
Cambridge [24]	LCO	Graphite	10	2670	0.045	8.38$\times 10^{-3}$–0.04	25 / 35 / 45	LR2032 coin
**LFP family**								
HUST [40]	LFP	Graphite	77	146 120	1.10	0.90–1.22	30	18650 cyl.
MATR [41]	LFP	Graphite	168	139 313	1.10	0.81–1.09	30	18650 cyl.
WZU [42]	LFP	Graphite	242	106 060	1 / 0.8 / 3	0.05–3.31	Seasonal	Cells + parallel packs
**Na‐ion family**								
NA‐ion [20]	Unknown	Unknown	64	12 604	1.00	0.71–0.98	25 / 30	18650 cyl.
**Zn‐ion family**								
ZN‐coin [20]	Zn	MnO_2_	91	40 417	5.5$\times 10^{-4}$	8.89$\times 10^{-5}$–4.89$\times 10^{-4}$	25 / 30 / 40	Coin
**hybrid chemistries**								
UL_PUR [43]	LCO + NCM442	Graphite	10	2245	3.40	2.50–3.33	23	18650 cyl.
HNEI [44]	LCO + NCM442	Graphite	14	15 164	2.80	0.99–2.62	25	18650 cyl.
Tongji [22]	Mixed NCA/NCM	Graphite / Graphite+Si	130	59 038	3.5 / 2.5	1.69–3.29	Various	18650 cyl.
**Multi chemistry system**								
SNL [45]	LFP/NCA/NCM	Graphite / Graphite+Si	60	92 856	1.1 / 3.2 / 3.0	0.81–2.96	15 / 25 / 35	18650 cyl.
SJTU [46]	LFP/NMC	Graphite	8	487 200	13.0	9.09–13.31	Room Temp	IFR32135

**FIGURE 2:**
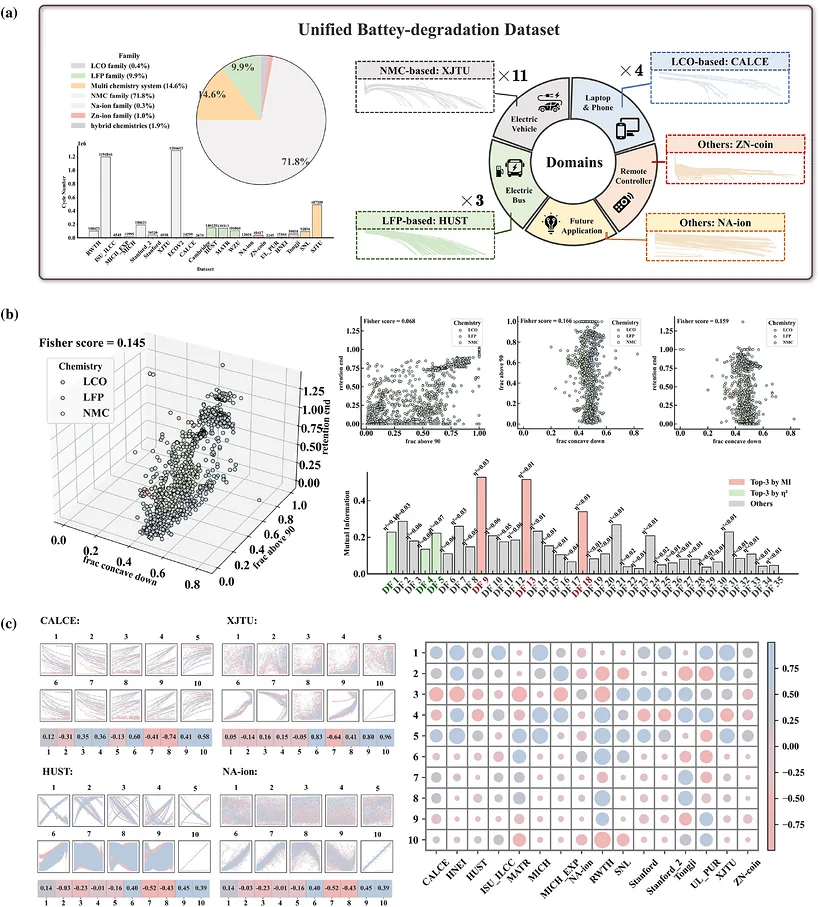
Dataset overview. (a) Overview of the unified battery capacity degradation corpus constructed in this study. The integrated dataset spans a broad range of electrochemical systems and application scenarios; NMC‐based cells account for the vast majority of samples, while emerging sodium‐ion, zinc‐ion, and mixed‐chemistry batteries occupy only a small fraction of the corpus. (b) Representative statistical analysis of DFs with respect to battery chemistry. The panel shows three‐dimensional scatter plots, two‐dimensional projections, and the corresponding mutual information and ANOVA effect sizes ($\eta^{2}$) between the DFs and chemistry labels. The overlap among chemistry‐coded point clouds indicates shared regions in the standardized DF space. Mutual information quantifies the dependence of the full feature distributions on chemistry, whereas $\eta^{2}$ quantifies the proportion of feature variance attributable to differences in chemistry‐specific means. (c) Correlation‐coefficient heat map and scatter plots between physical features and capacity. In each scatter plot, the x‐axis represents the cycle‐wise discharge capacity, whereas the y‐axis represents the value of the corresponding physical feature. The ten physical features are denoted by PF1–PF10, with their definitions provided in Note S5. Specifically, these features include statistical descriptors of discharge current and voltage, together with discharge duration.

In terms of form factor, the corpus spans most formats used in both laboratory experiments and industrial deployments. It includes widely adopted 18650 cylindrical cells (e.g., RWTH [34], HUST [40], HNEI [44], MATR [41]), pouch cells (e.g., MICH [36], ISU_ILCC [35]), prismatic cells for commercial applications (e.g., CALCE [39]), and coin cells used for fundamental electrochemical studies (e.g., ZN‐coin [20] and Cambridge [24]). This diversity in cell design ensures that the model is exposed to degradation behaviors typical of both small‐format cells and larger, application‐oriented devices.

From the perspective of electrode chemistry, the corpus covers both mainstream and emerging material systems. Cathode chemistries include lithium iron phosphate (LFP), lithium cobalt oxide (LCO), and several nickel‐rich oxide families (e.g., NMC and NCA), while anodes range from natural and artificial graphite to graphite–silicon composites and metallic zinc for aqueous systems. In addition, some datasets involve cells with electrolyte additives (e.g., VC‐enhanced formulations) or sodium‐ion batteries with partially undisclosed chemistries, introducing further compositional variability into the training corpus.

The datasets also exhibit substantial diversity in temperature conditions. Overall, the test temperatures range from approximately 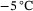 to 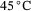, encompassing low‐temperature stress scenarios as well as high‐temperature accelerated aging. Beyond controlled thermal chambers, datasets such as WZU were collected under seasonal ambient conditions, thereby reflecting realistic environmental fluctuations including diurnal and seasonal temperature swings. Several datasets further provide measurements at multiple temperature levels within the same chemistry, enabling the TSFM to learn how thermal conditions modulate degradation dynamics.

In terms of usage scenarios, the corpus covers a broad spectrum from idealized laboratory protocols to application‐oriented cycling. It includes standard constant‐current/constant‐voltage (CC–CV) aging tests, as well as fast‐charging profiles, multi‐stage variable loads, partial cycling, and other complex duty cycles. Of particular note, the SJTU dataset was acquired by our group on large‐format IFR32135 cells designed for stationary energy storage. Its high nominal capacity and ESS‐oriented cycling protocols, detailed in Figure S1 and Table S1, complement the predominantly small‐capacity cells in other datasets and fill an important gap in the overall corpus. Collectively, this unified and diversified degradation corpus supports the development and evaluation of a TSFM‐based forecasting framework across a range of battery chemistries, form factors, temperatures, and usage conditions.

### 2.3 Feature Extraction

Feature extraction in this study is divided into two components: analysis of the endogenous characteristics of the degradation trajectories and extraction of exogenous variables from the charge–discharge process. Although time‐series forecasting is a mature research field, the single‐series setting often causes the intrinsic structure of the endogenous variable itself to be overlooked. To date, no study has systematically examined, across sufficiently diverse scenarios, the endogenous characteristics of capacity degradation during cycling for different battery chemistries. Here, we seek to characterize both the chemistry‐dependent variations and the cross‐chemistry commonalities in capacity‐fade trajectories. Specifically, we first extract 35 dimensionless degradation features (DFs) from the normalized cycle–capacity trajectories of three representative chemistries: LFP, LCO, and NMC (see Note S4 for detailed definitions). These DFs characterize the shape and temporal evolution of capacity fade, including trajectory curvature, capacity‐retention levels, global and local degradation rates, deviations from linear trends, and knee‐point behavior. As shown in Figure 2b and Figure S3, the chemistry‐coded samples occupy partially overlapping regions in the standardized DF space, indicating that cells with different chemistries can share similar combinations of trajectory‐level degradation characteristics. The associated mutual information values show that chemistry labels remain informative about the full DF distributions, whereas the relatively small ANOVA effect sizes ($\eta^{2}$) indicate that chemistry‐induced shifts in feature means explain only a limited proportion of the total variation. Together, these results suggest that cross‐chemistry commonalities coexist with chemistry‐specific differences in distributional spread, shape, and higher‐order characteristics. This shared structure in the endogenous variable provides an empirical basis for developing TSFM‐based capacity‐forecasting models that generalize across chemistries and operating conditions.

During training, our goal is to enhance the model–s ability to recognize embedded differences in physical operating conditions, thereby improving the generalization performance of capacity forecasts. Prior work such as TimeXer  [47] has demonstrated that appropriately incorporating covariate embeddings can substantially improve forecasting accuracy for the target series. However, most existing feature‐extraction schemes are designed for specific datasets or charge–discharge protocols and thus lack cross‐chemistry generality. To address this limitation, we leverage the diversity of our corpus and focus on extracting physical features within each discharge cycle. As illustrated in Figure 2c and Figures S4 and S5, the scatter relationships between these physical features and capacity exhibit pronounced chemistry‐dependent differences (Note S5). At the corpus level, our analysis corroborates the conclusion of Wang et al.  [48] that charge–discharge physical features are relatively insensitive to cycling protocols yet more strongly governed by the underlying chemistry. This complex dependence between endogenous and exogenous variables motivates the incorporation of physics‐based features into our time‐series capacity‐forecasting framework.

### 2.4 Battery Capacity Forecasting

We evaluate the proposed capacity forecasting framework on a heterogeneous corpus of 20 public datasets covering 1704 cells, multiple chemistries, form factors and operating conditions. Among these, 16 datasets are used for training and validation, while four datasets (EOCV2, SJTU, WZU and Cambridge) are completely excluded from training and only used for testing under unknown conditions. All methods are compared under a common window‐based prediction setting; detailed preprocessing procedures and splitting rules are provided in Note S6. Our approach trains a single TSFM‐based unified model on the aggregated training corpus, whereas each competing method (DLinear [49], FreTS [50], LightTS [51], PaiFilter [52], PatchTST [53], SegRNN [54]) is trained as an independent supervised model on each of the 20 datasets, making the evaluation protocol substantially more demanding in terms of cross‐dataset generalization.

The four held‐out datasets are selected to probe generalization under distinct types of distribution shift. EOCV2 contains high–energy‐density NMC cells with Carbon–SiO blended anodes and thus represents an electrode chemistry that does not appear in the training data. SJTU comprises large‐capacity (13 Ah) LFP/NMC IFR32135 cylindrical cells and tests extrapolation across capacity scale and industrial form factor. WZU combines LFP cells and parallel modules operating under seasonally varying ambient temperatures and nonstationary usage profiles. Cambridge consists of ultra‐small LCO coin cells (LR2032) with capacities of only several tens of mAh, providing a stringent test of generalization across extreme form factor and capacity scale.

On these unknown‐condition datasets, the unified TSFM model achieves Mean Absolute Error (MAE), Root Mean Squared Error (RMSE), and Mean Absolute Percentage Error (MAPE) values that are comparable to, and in many cases better than, those of per‐dataset supervised baselines (Figure 3a,c). The per‐cell error distributions for our method are generally compact, suggesting relatively consistent performance across individual cells. Friedman tests based on cell‐wise average errors show that the unified model attains the best average rank on three of the four held‐out datasets and remains within the top three on EOCV2. These results indicate that a single TSFM‐based model, trained once on heterogeneous degradation data, can achieve competitive cross‐dataset forecasting performance on previously unseen chemistries, capacity scales, formats, and operating regimes without retraining or adaptation.

**FIGURE 3:**
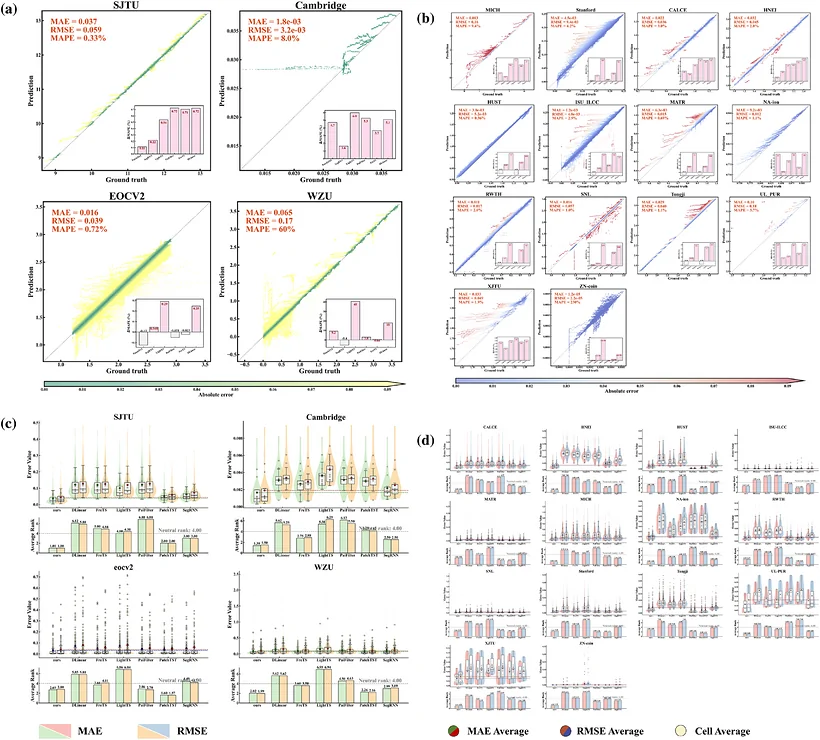
Illustrations of capacity forecasting results. (a) Capacity forecasting performance of the proposed method on unknown datasets, together with the MAPE differences relative to the predictions generated by recent state‐of‐the‐art time‐series forecasting models, showing that our approach maintains competitive forecasting accuracy under previously unobserved conditions. (b) Capacity forecasting results on known datasets, where the model exhibits robust performance across a wide spectrum of battery chemistries, form factors, and operating scenarios. Using a single unified time‐series forecasting model, our method achieves accuracy comparable to or better than that of multiple supervised state‐of‐the‐art time‐series forecasting baselines. (c) Distribution of per‐cell average errors and Friedman ranking on unknown datasets. (d) Distribution of per‐cell average errors and Friedman ranking on known datasets. The combined box–violin plots illustrate the comparative performance of the evaluated methods, and the Friedman rankings show that the proposed unified model ranks within the top three across all evaluated datasets and achieves the best rank on multiple datasets.

To directly assess forecasting performance in the more critical accelerated‐degradation stage, we conduct a stage‐wise analysis on four datasets with identifiable slow‐ and accelerated‐degradation regimes. As shown in Note S12 and Figure S8, all methods exhibit larger errors during accelerated degradation than during slow degradation. Nevertheless, the proposed model remains competitive in both stages, with a clear advantage on SJTU and best or near‐best performance on HUST, Stanford, and MICH.

To assess the performance of the framework within the training domain, we further perform large‐scale validation on all degradation trajectories from the remaining 16 datasets. Using the same window protocol (96‐step lookback and 96‐step forecast horizon), the unified TSFM and all baselines are applied to millions of forecast windows spanning lithium‐ion and sodium‐ion chemistries, LCO/NMC/NCA/LFP families, cylindrical, pouch, prismatic and coin formats, temperatures from 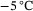 to 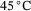, and usage profiles including constant‐current/constant‐voltage cycling, fast charging, partial cycling and dynamic loads. As summarized in Figure 3b,d, the unified TSFM achieves the lowest or near‐lowest MAE, RMSE and MAPE on most datasets and generally exhibits compact error distributions relative to the comparison methods. Friedman ranking based on per‐cell average errors confirms that, across this broad experimental space, the single TSFM model frequently attains rank 1 and never falls outside the top three. Taken together, the unknown‐condition experiments, the stage‐wise verification, and the large‐scale in‐domain validation provide empirical evidence that the proposed framework can support capacity degradation forecasting across a diverse range of chemistries, formats, operating conditions, and capacity scales.

### 2.5 LIME‐Based Interpretation

To further probe what the model has learned, we conduct an interpretability study based on time‐series LIME attribution maps. The complete LIME analysis and the corresponding generation procedure are provided in Supplementary Note 8 and Algorithm 4. For each sliding window, LIME perturbs the 96‐step lookback sequence and fits a local surrogate regressor to attribute the forecast to individual time steps. Aggregating these local explanations over all windows yields a two‐dimensional “LIME map,” whose horizontal axis corresponds to lookback positions and whose vertical axis indexes forecasting windows, with color encoding signed local importance. Positive values indicate historical positions that locally increase the predicted capacity, whereas negative values indicate positions that reduce the prediction.

Figure 4a presents both the cycle‐wise forecasting results and the corresponding LIME maps on the four unknown‐condition datasets, namely EOCV2, SJTU, WZU, and Cambridge. The complete LIME maps for the 16 known‐condition datasets have been moved to Note S8, where their variations across chemistries, form factors, and operating conditions are reported in detail. The LIME maps further show that the proposed model assigns structured positive and negative importance to different portions of the historical sequence. In contrast, several comparison methods place more uniform importance on recent observations or produce less structured attribution patterns. These results suggest that the proposed model uses information distributed across the historical capacity trajectory rather than relying exclusively on the most recent trend.

**FIGURE 4:**
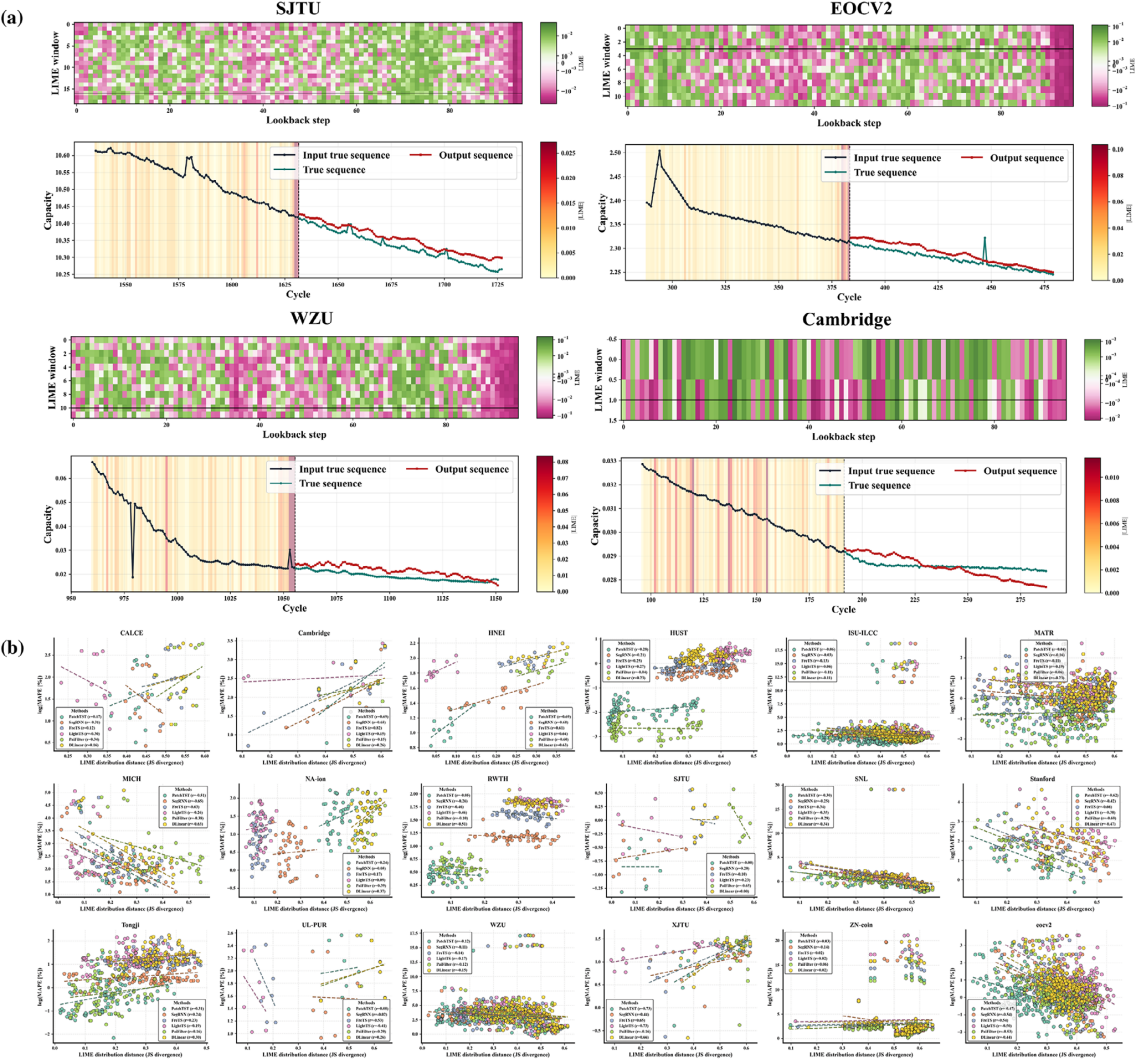
LIME‐based interpretation of capacity degradation forecasting. (a) Cycle‐wise comparison between the predicted and measured capacity trajectories and LIME importance maps on the four unknown‐condition datasets. (b) Relationship between explanation discrepancy and forecasting accuracy: scatter plots of LIME distribution distance versus log(MAPE) for all methods, linking differences in temporal attribution patterns to forecasting performance.

To quantitatively assess how these attribution patterns relate to predictive performance, we analyze the relationship between explanation discrepancy and forecast error in Note S9. For each dataset–method–cell combination, we compute a LIME distribution distance by flattening the corresponding LIME maps and measuring the Jensen–Shannon (JS) divergence between the comparison model's importance distribution and that of the proposed method. The associated prediction error is summarized by log(MAPE). The exact definitions of the JS divergence and log(MAPE) are provided in Note S9. As illustrated in Figure 4b, several representative datasets, including Cambridge, HNEI, and XJTU, exhibit a tendency for larger attribution discrepancies to be associated with higher forecasting errors. Trend fitting is also provided for all evaluated datasets. This relationship does not establish that a particular attribution pattern is uniquely correct, but it suggests that differences in the temporal evidence used by the forecasting models are associated with differences in predictive performance.

To assess the contribution of the physics‐guided embedding, we conduct five ablation experiments in Note S13 by removing different physical‐feature groups or the endogenous capacity sequence. The complete model achieves the best overall performance on the four unknown‐condition datasets. Removing voltage descriptors causes the most pronounced performance degradation, whereas the PF‐only variant yields the largest errors, indicating that physical features are most effective as auxiliary signals rather than replacements for the capacity trajectory. The paired full‐minus‐ablation LIME analysis further shows that the physics‐guided embedding redistributes temporal sensitivity across the lookback window, with voltage‐feature removal producing the largest structured attribution changes. These results suggest that auxiliary physical information improves forecasting by helping the model more effectively organize and use the temporal evidence contained in the historical capacity sequence.

Taken together, the cycle‐wise trajectory comparisons, LIME analysis, and physics‐guided ablation experiments provide complementary evidence that the proposed model can learn transferable temporal representations from heterogeneous degradation data. The results further suggest that the physics‐guided embedding improves forecasting by changing how the model uses historical capacity information, with voltage‐related descriptors contributing more strongly than discharge duration or current‐related statistics.

## 3 Discussion

Battery technologies are entering a period of rapid diversification driven by the growth of electric mobility, renewable integration, and large‐scale stationary storage. This diversification, which spans chemistries, capacities, form factors, and duty cycles, poses a central challenge for battery management systems: how to deliver reliable health prognostics across heterogeneous and evolving fleets rather than a single, well‐controlled cell type. This study addresses that challenge by reframing capacity forecasting as a foundation‐model problem and showing that a single TSFM‐based architecture, trained once on a sufficiently diverse degradation corpus, can generalize across previously unknown chemistries, scales, operating regimes, and measurement environments.

At the core of this work is the creation of a unified, multi‐chemistry degradation corpus of unprecedented scale and diversity. By harmonizing 20 public datasets encompassing 1704 cells and nearly four million cycle segments, spanning Li‐ion (LCO, NMC/NCA, LFP), sodium‐ion, zinc‐ion, hybrid systems, and both laboratory and application‐oriented profiles, we establish a time‐series analogue of the large text corpora that underpin foundation models in natural language processing. This corpus enables the model to learn shared temporal priors of degradation while still capturing chemistry‐specific nuances through physics‐guided contrastive representation learning.

Equally important is how this unified model behaves when faced with genuine distribution shifts. Entire datasets, such as large‐format IFR32135 energy‐storage cells, ultra‐small LCO coin cells, SiO‐enhanced NMC systems, and seasonal‐temperature LFP profiles, are withheld during training to create zero‐shot test conditions. Under these shifts, the unified TSFM achieves stable, often superior accuracy compared with baselines retrained separately on each dataset, demonstrating cross‐chemistry, cross‐capacity, cross‐format, and cross‐regime generalization. Its consistent ranking among top performers, even when there is no chemical or operational overlap with the training distribution, suggests that the model internalizes degradation patterns that are both structural and transferable.

The nature of the learned representations further supports this picture. LIME attribution maps show that the TSFM focuses on degradation‐relevant segments of history and produces coherent, physically meaningful attribution modes aligned with chemistry‐dependent aging behavior. Models whose attribution patterns deviate more strongly from those of the unified TSFM tend to yield higher forecasting error, indicating that the learned representations capture latent physical regularities across battery families rather than overfitting to dataset‐specific artefacts.

Taken together, these findings outline a practical route toward scalable, transferable, and maintenance‐efficient health prediction in real BMS deployments. Instead of maintaining numerous bespoke models for different chemistries and usage scenarios, operators could rely on a single forecasting backbone that adapts to new domains with lightweight updates. This approach reduces engineering overhead and improves cross‐platform consistency, which is an increasingly important requirement as battery supply chains and application ecosystems continue to diversify.

Looking ahead, enriching the degradation corpus with field telemetry, emerging chemistries (for example, solid‐state, sodium‐rich, and dual‐ion systems), and multiscale aging experiments will be key to extending the model–s coverage. Integrating such models into operational BMS workflows, including onboard prognostics, fleet analytics, adaptive fast‐charging control, and lifetime‐aware energy management, could ultimately establish TSFM‐based architectures as foundational tools for next‐generation battery technologies. In this sense, our study offers both a high‐performing forecasting model and a generalizable blueprint for translating foundation‐model principles to the domain of electrochemical degradation. Overall, the proposed framework is computationally feasible for cloud‐based BMS analytics and accelerator‐equipped edge platforms, while deployment on resource‐constrained microcontrollers would require further model compression; detailed profiling results are provided in Supplementary Note 11.

## 4 Methods

### 4.1 Problem Formulation

In practical battery health management, operators are primarily interested in forecasting how the **discharge capacity** of each cell will degrade over future cycles under unknown or changing operating conditions. We therefore treat the cycle‐wise discharge capacity as the **endogenous variable**, and use physically meaningful descriptors extracted from each charge–discharge cycle as **exogenous variables** that are available during training but may be missing or partially observed at deployment.

Formally, let the endogenous series be given in Equation (1).

(1) $$y_{1:T}=\left\{y_{1},y_{2},\text{…},y_{T}\right\}\in R^{T}.$$

Here, $y_{t}$ denotes the normalized discharge capacity at the t‐th cycle. For each cycle, we also compute C physical descriptors (e.g., charging time, the mean and variance of current and voltage, voltage kurtosis), organized into the multivariate exogenous sequence in Equation (2).

(2) $$X_{1:T_{x}}=\left\{x_{1},x_{2},\text{…},x_{T_{x}}\right\}\in R^{T_{x}\times C}.$$

Here, $x_{t}={\left[x_{t}^{\left(1\right)},\text{…},x_{t}^{\left(C\right)}\right]}^{⊤}$ collects the physical features at cycle t. In the present experiments, one physical‐feature vector is extracted for each valid charge–discharge cycle and paired with the capacity measured at the same cycle; therefore, the endogenous and exogenous sequences are cycle‐wise aligned and $T_{x}=T$. We retain the general notation $T_{x}$ to allow extensions to asynchronous sampling or partially observed exogenous variables, for which $T_{x}$ may differ from T.

Given a historical window of T cycles and its associated exogenous information, our goal is to learn a forecasting function $f_{\theta }$, parameterized by θ, that predicts the future capacity over a horizon of length S, as formalized in Equation (3).

(3) $${\hat{y}}_{T+1:T+S}=f_{\theta }\left(y_{1:T},X_{1:T_{x}}\right).$$

In this work, $f_{\theta }$ is instantiated as a time‐series foundation model built on the **Timer** architecture, and is trained with both a supervised forecasting objective and a physics‐guided contrastive objective so as to generalize across chemistries, form factors, and operating conditions.

### 4.2 Timer‐Based Time‐Series Foundation Model

To support capacity forecasting across heterogeneous and potentially unknown operating profiles, we adopt **Timer**  [33] as the backbone time‐series foundation model. Timer is a scalable, decoder‐only Transformer originally developed for multivariate time series and designed to capture long‐range temporal dependencies through an autoregressive generation paradigm similar to that used in LLMs. In our setting, Timer provides a shared *foundation* that encodes degradation dynamics and can be adapted to different cells via lightweight fine‐tuning (e.g., LoRA).

Given an input sequence, we follow the original Timer formulation and partition the series into non‐overlapping temporal tokens of length S, as shown in Equation (4).

(4) $$s_{i}=\left\{y_{(i-1)S+1},\text{…},y_{iS}\right\}\in R^{S\times 1},\;i=1,\text{…},N.$$

Here, NS is the total sequence length and T=NS. Each token $s_{i}$ is projected into a latent embedding space and augmented with temporal encoding, then passed through a stack of causal Transformer blocks, as described in Equation (5).

(5) $$\begin{array}{cc} & h_{i}^{0}=W_{e}s_{i}+{\text{TE}}_{i},\\ & h^{l}=\text{TrmBlock}\left(h^{l-1}\right),\;l=1,\text{…},L,\\ & {\hat{s}}_{i+1}=W_{d}h_{i}^{L}, \end{array}$$

where $W_{e},W_{d}\in R^{D\times (S\cdot C)}$ are the input/output projection matrices, $h_{i}^{L}$ is the final latent representation at step i after L decoder blocks, and ${\text{TE}}_{i}$ denotes the temporal encoding.

The model is trained with a generative forecasting objective that minimizes the mean squared error (MSE) between the predicted and true segments, as defined in Equation (6).

(6) $$L_{\text{forecasting}}=\frac{1}{NS}\sum_{i=2}^{N}{∥s_{i}-{\hat{s}}_{i}∥}_{2}^{2}.$$

In our application, this objective teaches the model to reproduce the capacity degradation trajectory in an autoregressive manner, using only the capacity history as input. Crucially, the same Timer backbone is also exposed to physical features through a dedicated physics‐embedding pathway during training, which we describe next.

### 4.3 Physics‐Embedded Contrastive Learning

Purely supervised training on capacity trajectories can lead to models that fit dataset‐specific patterns but fail to generalize across chemistries, protocols, and environments. At the same time, fully conditioning on all physical variables at inference time is often impractical in real‐world BMS deployments, where some signals may be noisy, missing, or unavailable. To bridge this gap, we introduce a *physics‐embedded* contrastive learning scheme: physical descriptors are used as an auxiliary training signal to shape the latent representation of the model, but are not required as inputs at inference.

#### 4.3.1 Triplet Construction From Physical Descriptors

From the exogenous sequence $X_{1:T_{x}}$, we construct fixed‐length segments of length L and form training triplets $\left(x_{\text{anchor}},x_{\text{positive}},x_{\text{negative}}\right)$, where

- **Anchor** $x_{\text{anchor}}\in R^{L\times C}$ is a segment of physical features corresponding to a given capacity window;
- **Positive** $x_{\text{positive}}\in R^{L\times C}$ is drawn from a nearby time window (e.g., a future window offset by L cycles), which typically reflects a similar degradation stage under slightly different operating conditions;
- **Negative** $x_{\text{negative}}\in R^{L\times C}$ is sampled from a distant part of the trajectory (e.g., early vs. late life), representing a substantially different degradation state.

This triplet construction encodes the inductive bias that degradation evolves gradually, so physically similar segments close in time should be mapped to nearby representations, while far‐apart segments should be separated in the latent space. From the perspective of battery degradation, the anchor represents a cycle‐aligned physical‐feature segment associated with a reference capacity window. The positive sample is selected from a temporally adjacent window and therefore generally corresponds to a similar degradation stage, whereas the negative sample is drawn from a more distant region of the trajectory and represents a substantially different degradation state. Here, “anchor,” “positive,” and “negative” describe the roles of the samples in the contrastive‐learning objective rather than distinct electrochemical mechanisms. The “random drift” operation is a stochastic time‐series augmentation applied during contrastive learning to generate perturbed representations and improve representation robustness;

#### 4.3.2 Physics Encoder and Timer Embedding

Each segment $x\in R^{L\times C}$ is first passed through a feature expansion module (implemented as a linear MLP) that maps the C‐dimensional physical descriptors into a D‐dimensional embedding compatible with the Timer backbone:

$$z=\Phi \left(x\right)\in R^{L\times D},$$

where Φ(·) denotes the physics encoder. The resulting embeddings are then fed into the Timer model as input embeddings (instead of capacity tokens), and the output hidden representations are aggregated into a single vector per segment. In this way, the same foundation model learns to encode both *capacity‐view* and *physics‐view* sequences within a shared latent space.

#### 4.3.3 Contrastive Objective

To align these physics‐view representations, we employ an InfoNCE‐style contrastive loss that brings the anchor and positive segments closer while pushing the negative segment away. Let $z_{a}$, $z_{p}$, and $z_{n}$ denote the aggregated embeddings of the anchor, positive, and negative segments, respectively. We first define the cosine similarities in Equations (7), and then specify the physics‐guided contrastive loss in Equation (8).

(7) $$s_{ap}=\mathrm{sim}\left(z_{a},z_{p}\right),s_{an}=\mathrm{sim}\left(z_{a},z_{n}\right)$$

(8) $$L_{\text{physics}}=-\log\frac{\exp(s_{ap}/\tau )}{\exp\left(s_{ap}/\tau \right)+\exp\left(s_{an}/\tau \right)}.$$

Here, sim(·,·) denotes cosine similarity and τ is a temperature hyperparameter. In practice, we further apply a small Gaussian perturbation to the embeddings before computing the loss, which encourages smoother, more robust representations. This physics‐guided contrastive learning forces the Timer backbone to organize its latent space according to degradation stage and physical context, even though capacity itself is not explicitly used in this branch.

### 4.4 Joint Training and Inference

The final training objective combines the supervised capacity‐forecasting loss and the physics‐embedded contrastive loss, as given in Equation (9).

(9) $$L_{\text{total}}=\alpha \cdot L_{\text{physics}}+\beta \cdot L_{\text{forecasting}}.$$

Here, α and β are weighting coefficients. The forecasting term $L_{\text{forecasting}}$ is defined in Equation (6), while $L_{\text{physics}}$ is given in Equation (8).

Intuitively, the forecasting loss teaches the model to reproduce capacity trajectories from historical capacity alone, which is exactly the information available in many real BMS logs. The physics contrastive loss, on the other hand, injects information from charge–discharge descriptors during training, shaping the foundation model to respect the underlying physical similarities and differences across chemistries, temperatures, and protocols. Because the exogenous variables are only required in the contrastive branch, they are not needed at inference time: deployment simply feeds past capacity measurements into the Timer backbone to obtain future capacity forecasts. This training strategy yields a physics‐aware yet input‐agnostic model that can generalize across multiple battery types, operating conditions, and datasets using a single time‐series foundation model. For more details and parameters of the methods, please refer to Note S10.

## Author Contributions

Joey Chan designed and implemented the numerical experiment protocol and led the initial manuscript drafting. Huan Wang conceived the overall study framework and co‐led the writing of the paper. Haoyu Pan contributed to manuscript drafting and performed detailed checks and revisions. Wei Wu and Zirong Wang were responsible for the procurement of experimental equipment and the acquisition of the SJTU dataset. Zhen Chen provided guidance on high‐level architecture design and language polishing. The remaining co‐authors offered critical feedback and domain‐specific insights throughout the study. The project was initiated, coordinated, and supervised by Prof. Xi.

## Conflicts of Interest

The authors declare no conflicts of interests.

## Supporting information


**Supporting File 1**: advs77261‐sup‐0001‐SuppMat.pdf

**Supporting File 2**: advs77261‐sup‐0002‐SuppMat.zip

**Supporting File 3**: advs77261‐sup‐0003‐SuppMat.pdf

## Data Availability

The data that support the findings of this study are openly available in Universal Battery Degradation at https://zenodo.org/records/21528816?token=eyJhbGciOiJIUzUxMiJ9.eyJpZCI6ImZkNTRlNWIxLWRkYzYtNDhhOC1iY2JiLWI1NDE0Y2M5NDBkYyIsImRhdGEiOnt9LCJyYW5kb20iOiJjZjVkYjY5MzA2YWE1MmVjYTA4YWEzZmFjMjIwNTYxNCJ9.aFz4aRQmer9YZQt7Z7h9EzM_NXDqhTUUDdB5isMbOVPihPWRH‐DAhxpcvIO, reference number https://doi.org/10.5281/zenodo.21528816.
