# Supplementary material for: Universal Battery Capacity Degradation Forecasting Driven by Foundation Models Across Diverse Chemistries and Conditions
Source: Adv Sci (Weinh). 2026 Aug 24;13(63):e77261. doi: 10.1002/advs.77261 (PMC13502271; doi:10.1002/advs.77261)
Supplement: Supplementary file 2 [file ADVS-13-e77261-s002.pdf]

All Experiment result

tri˙horizon˙shared˙axes

CALCE | MAE — Tri-Horizon Compare Board

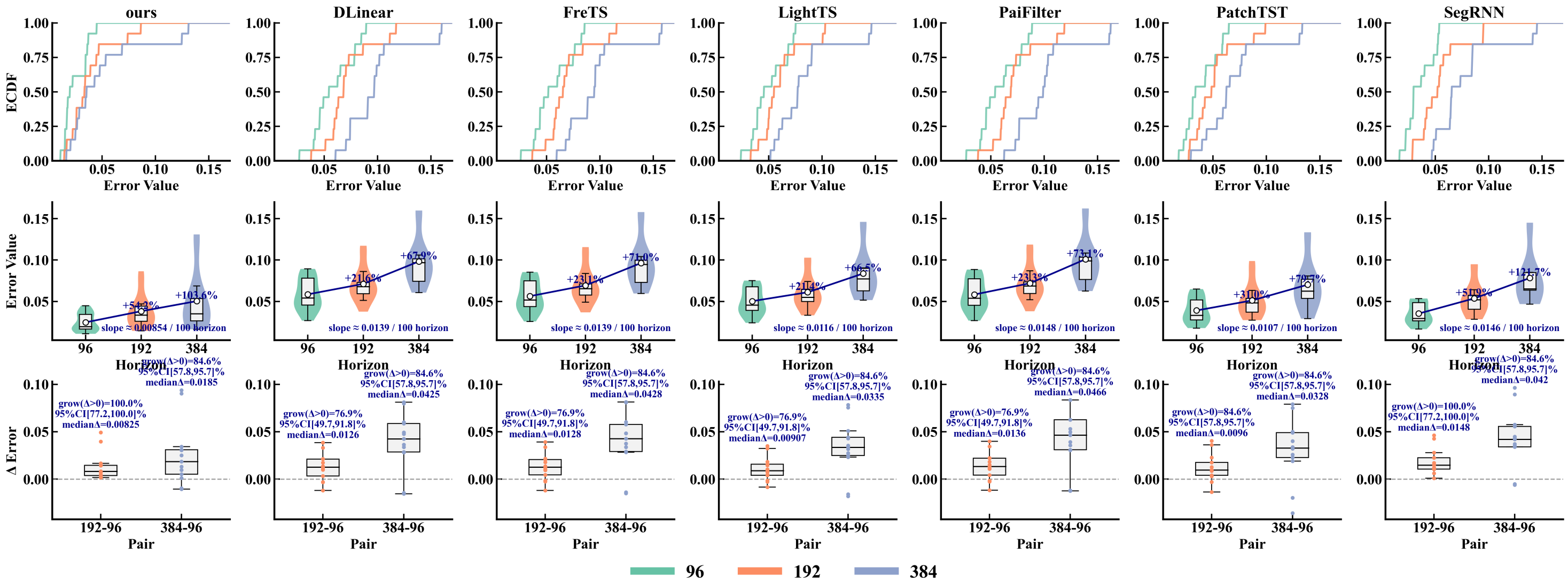

CALCE | RMSE — Tri-Horizon Compare Board

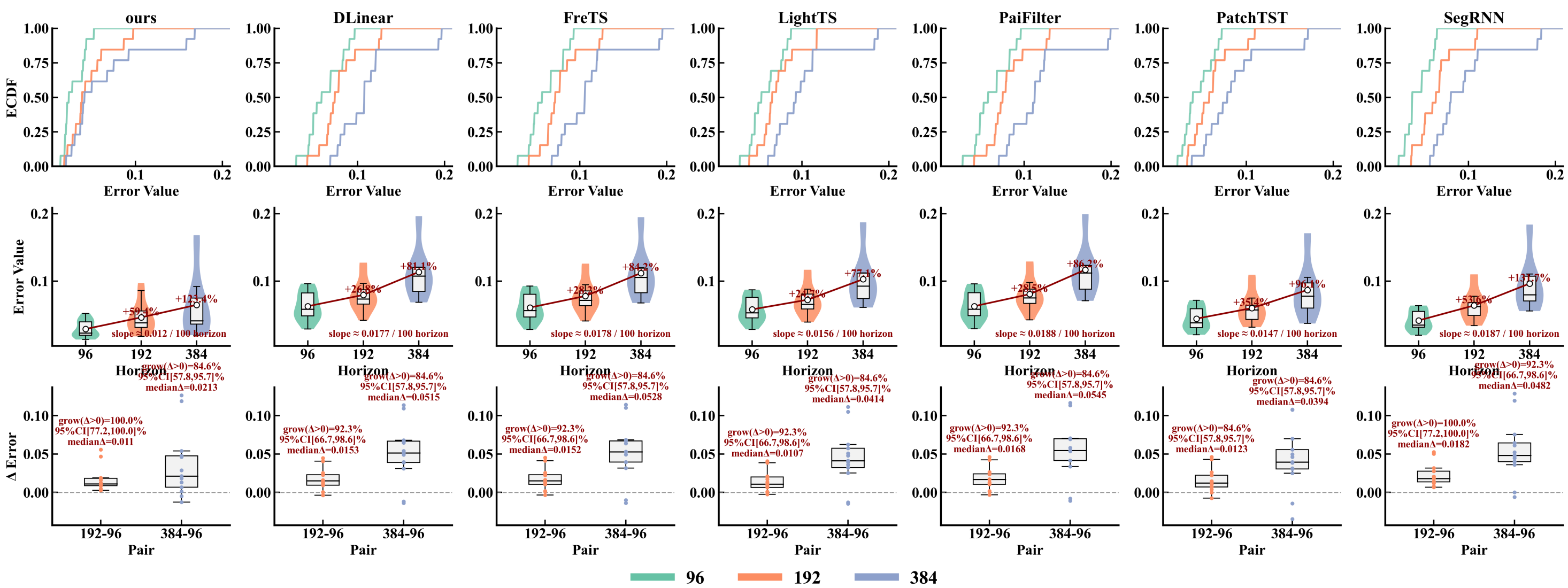

Cambridge | MAE — Tri-Horizon Compare Board

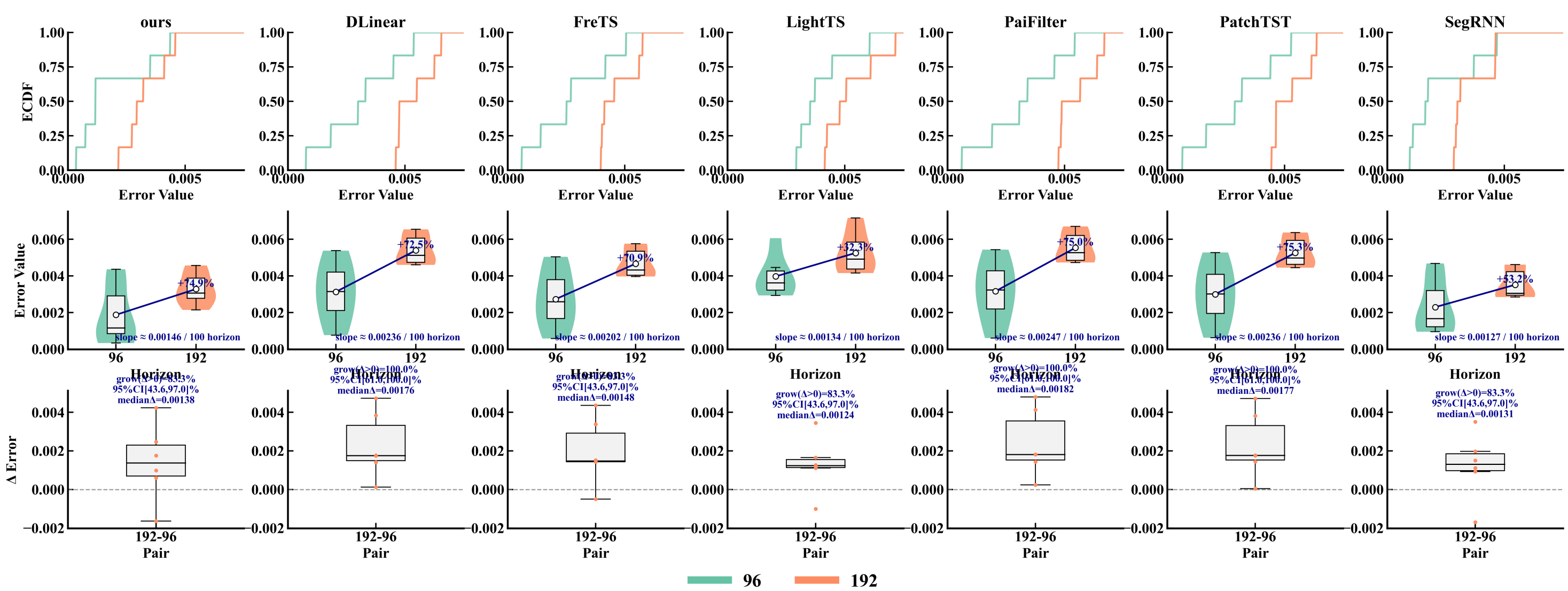

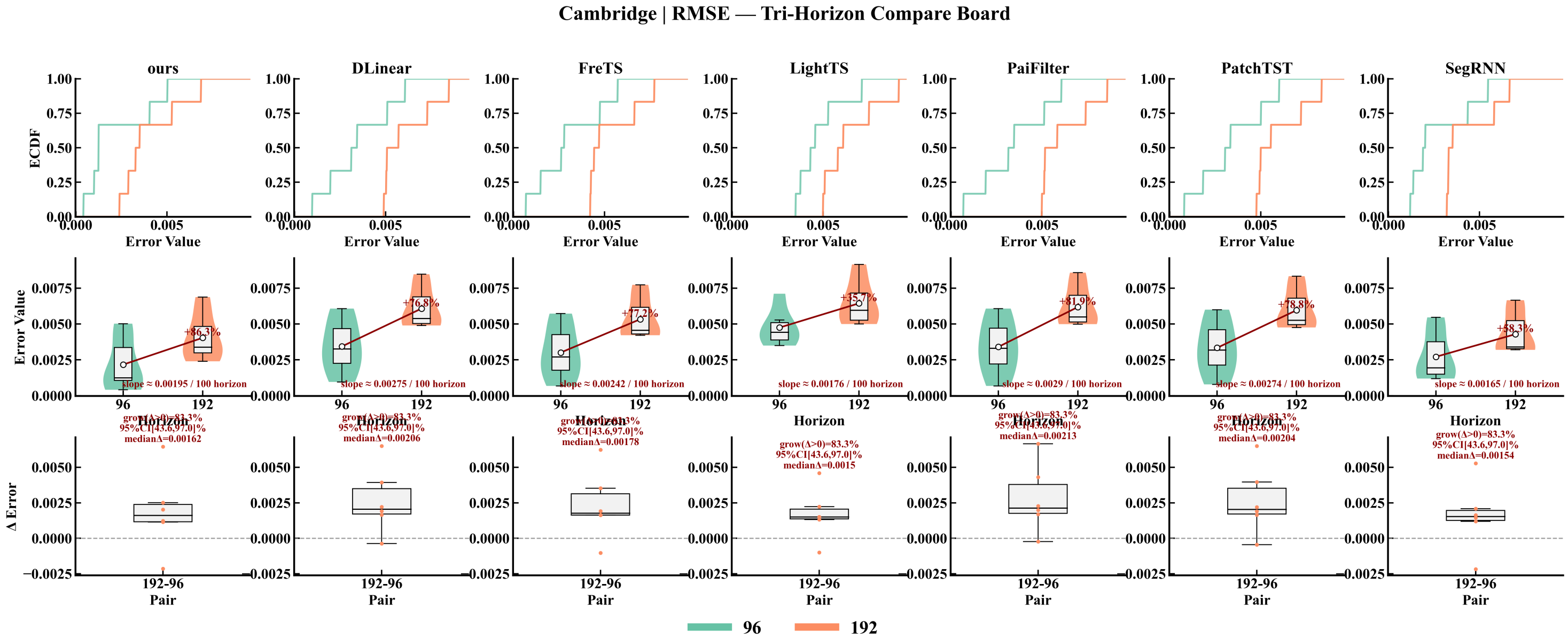
Cambridge | RMSE — Tri-Horizon Compare Board
ours
DLinear
FreTS
LightTS
PaiFilter
PatchTST
SegRNN
ECDF
Error Value
Horizon
Δ Error
192-96
Pair
96
192

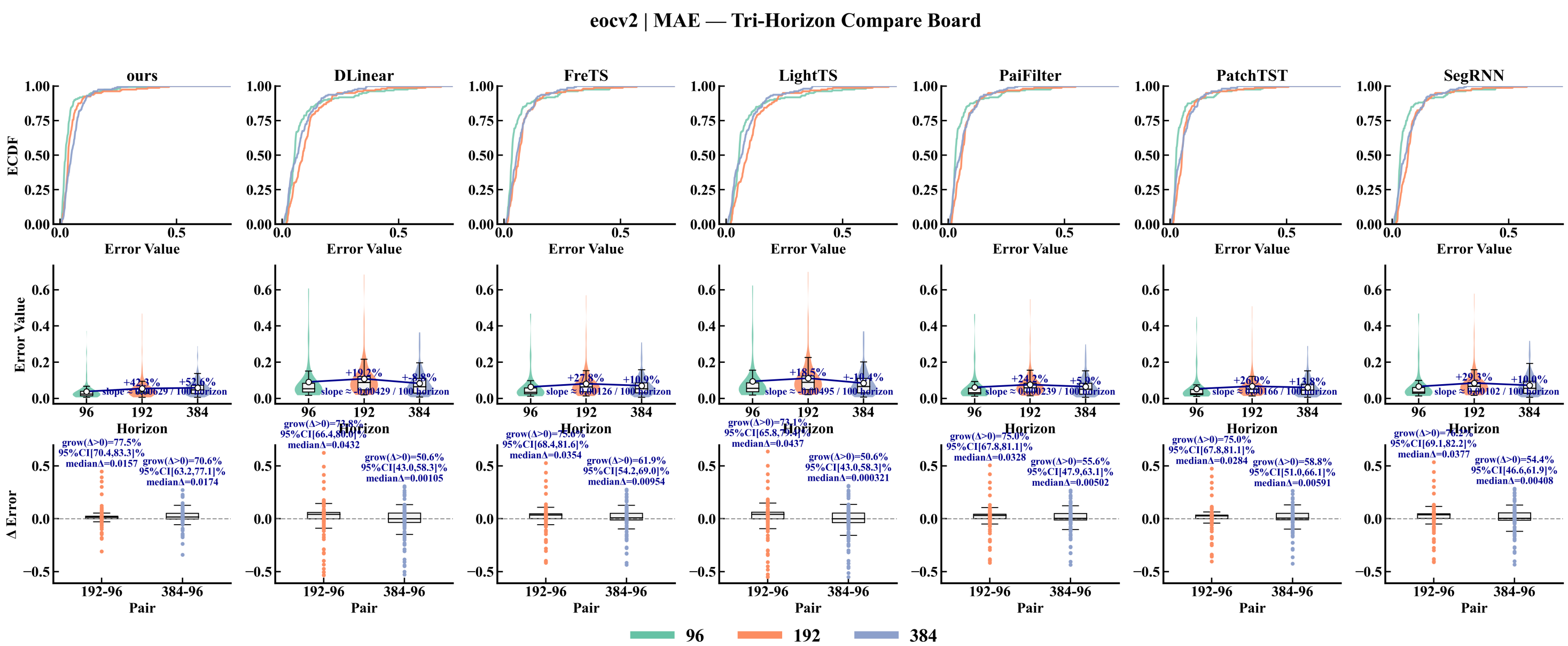
eocv2 | MAE — Tri-Horizon Compare Board
ours
DLinear
FreTS
LightTS
PaiFilter
PatchTST
SegRNN
ECDF
Error Value
Horizon
Δ Error
192-96
384-96
Pair
96
192
384

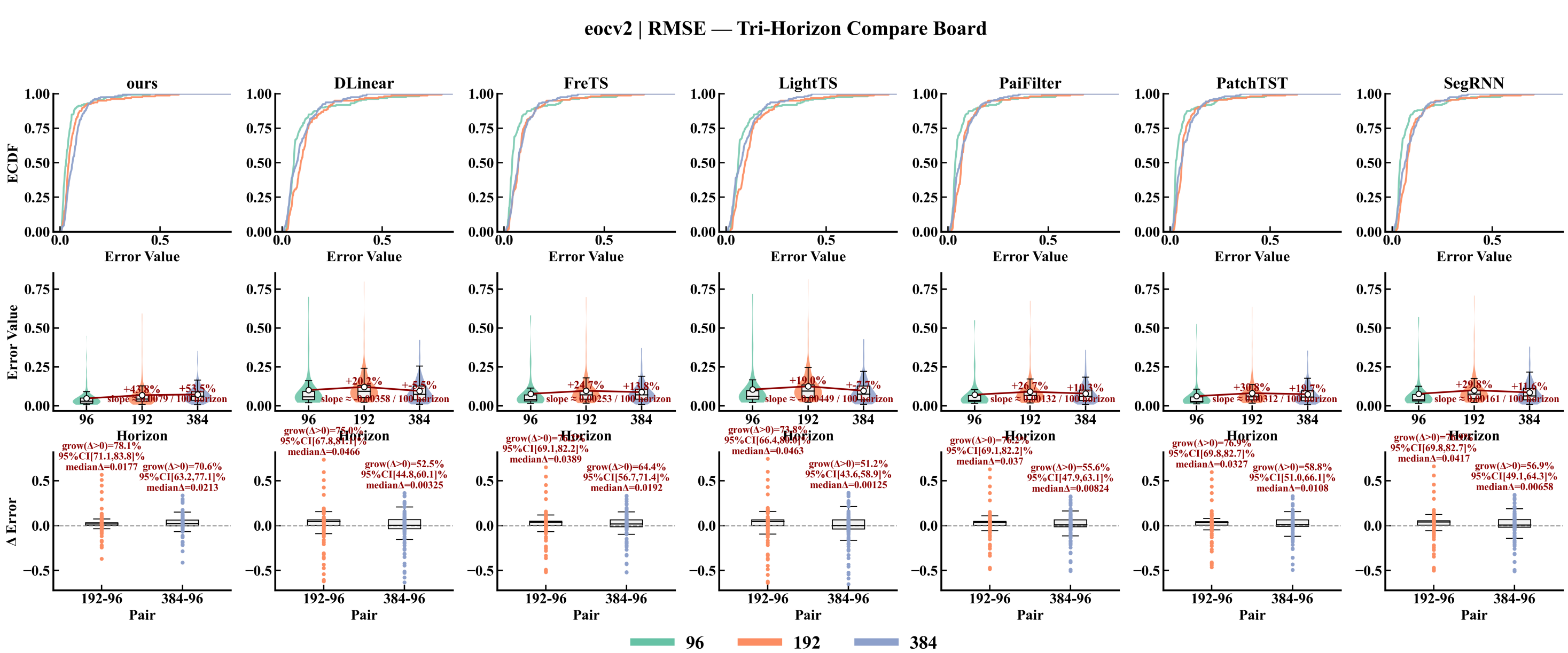
eocv2 | RMSE — Tri-Horizon Compare Board
ours
DLinear
FreTS
LightTS
PaiFilter
PatchTST
SegRNN
ECDF
Error Value
Horizon
Δ Error
192-96
384-96
Pair
96
192
384

HNEI | MAE — Tri-Horizon Compare Board

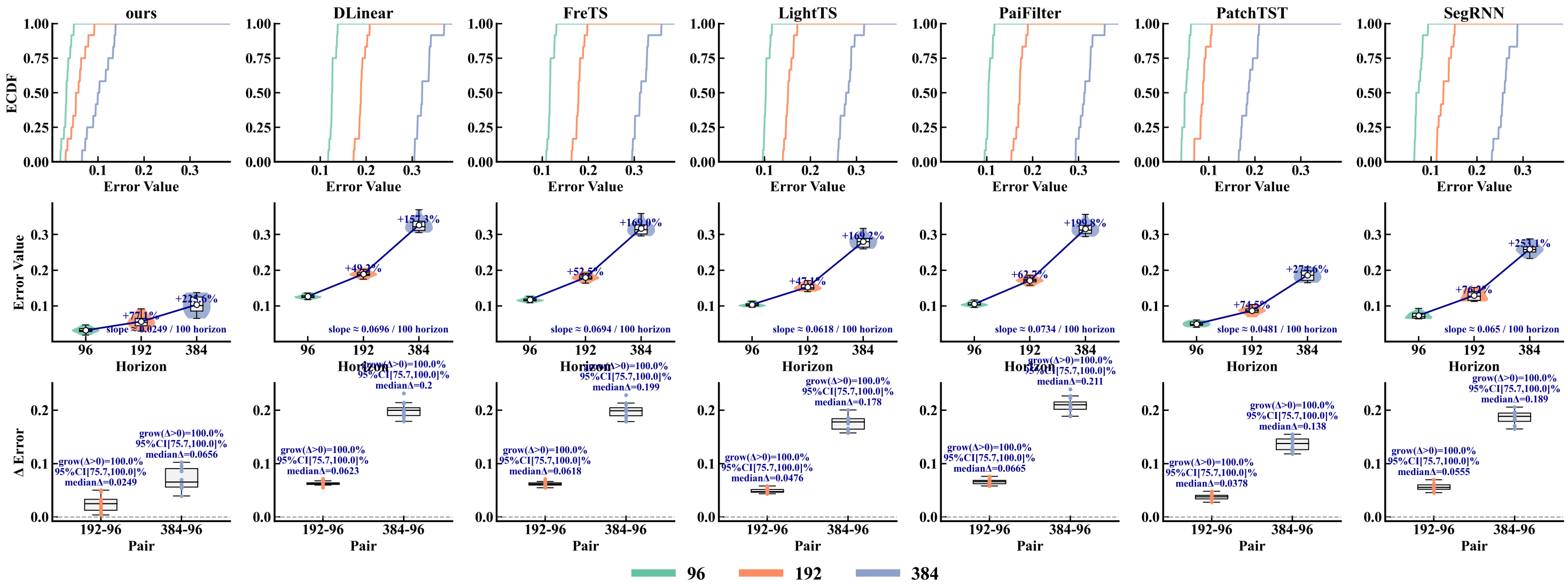

HNEI | RMSE — Tri-Horizon Compare Board

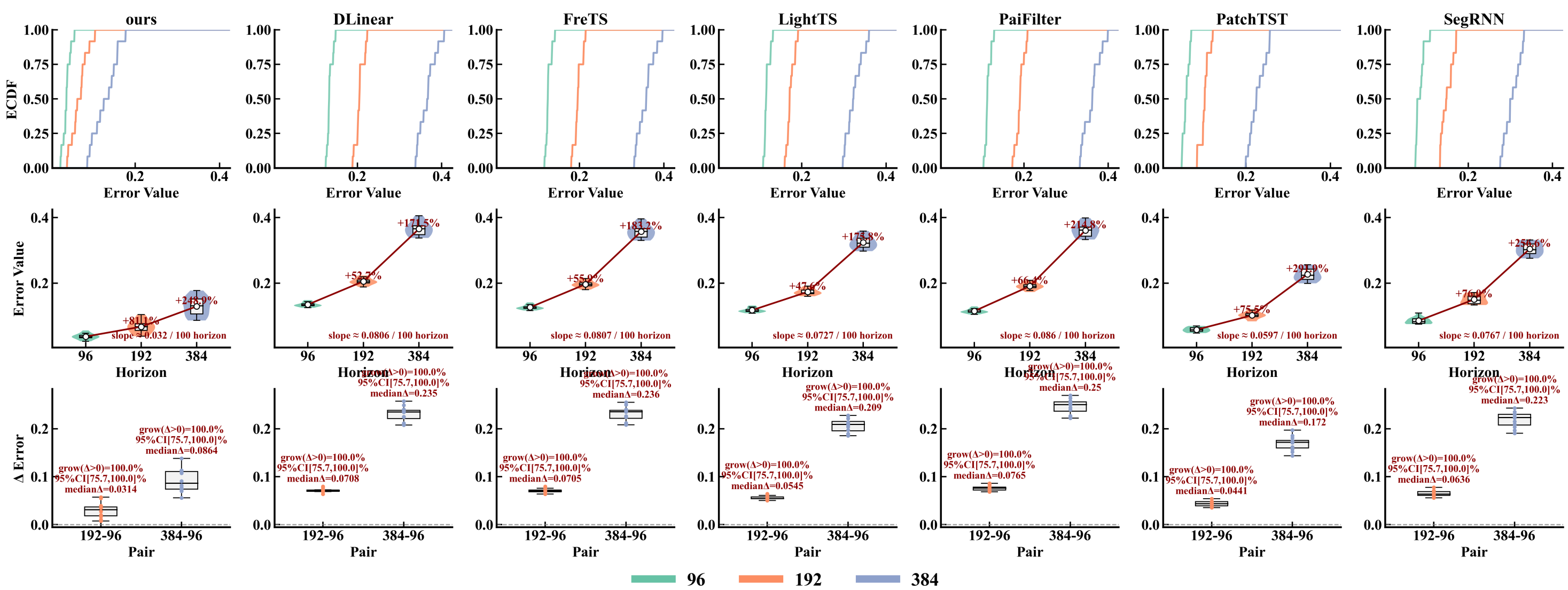

HUST | MAE — Tri-Horizon Compare Board

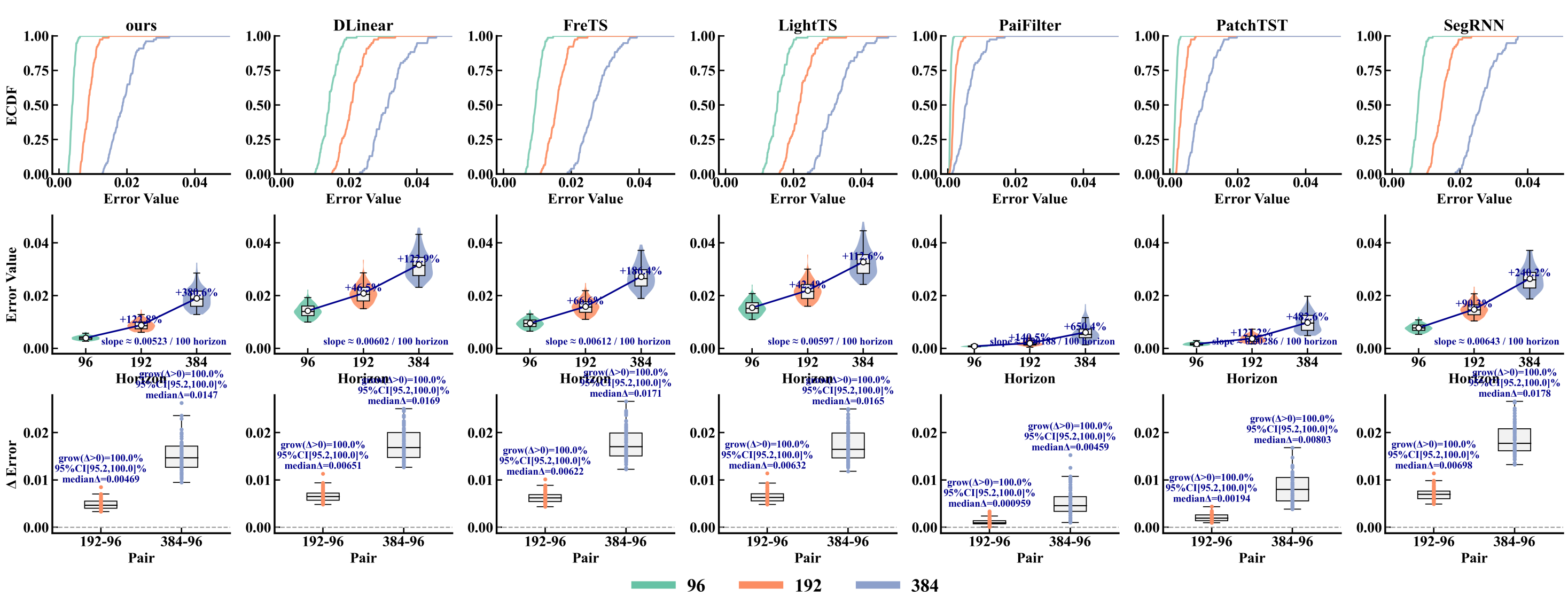

HUST | RMSE — Tri-Horizon Compare Board

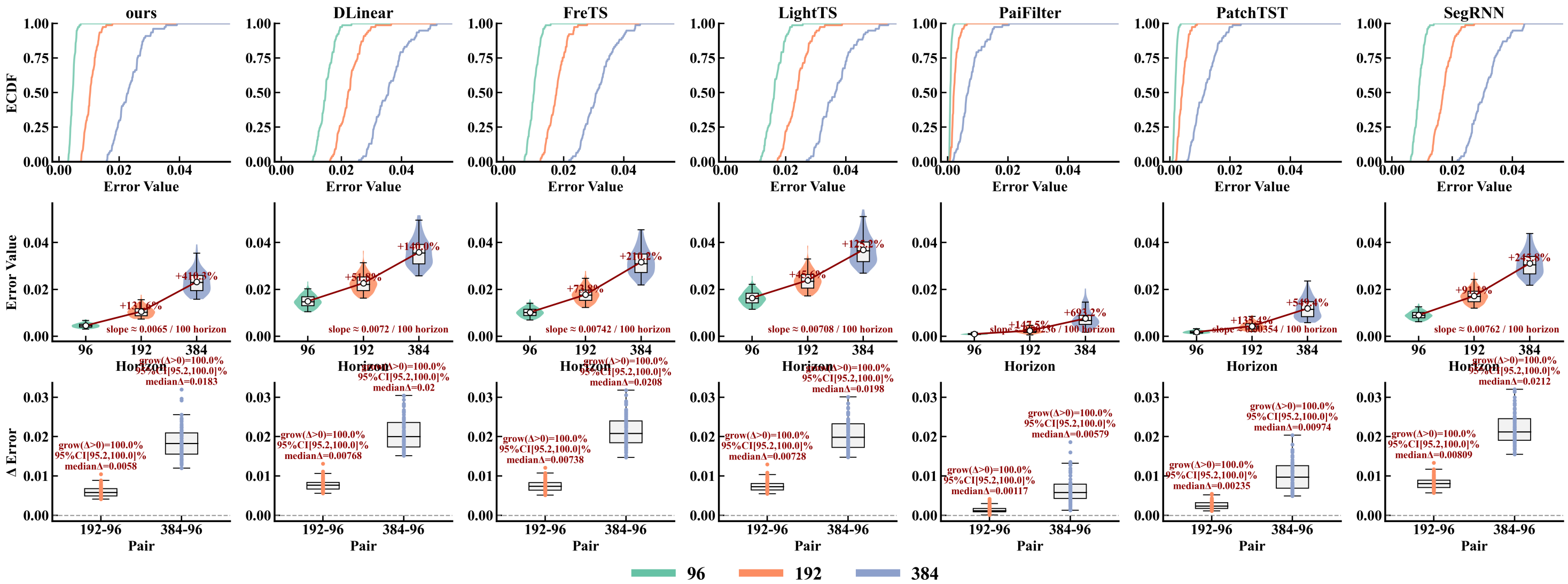

ISU-ILCC | MAE — Tri-Horizon Compare Board

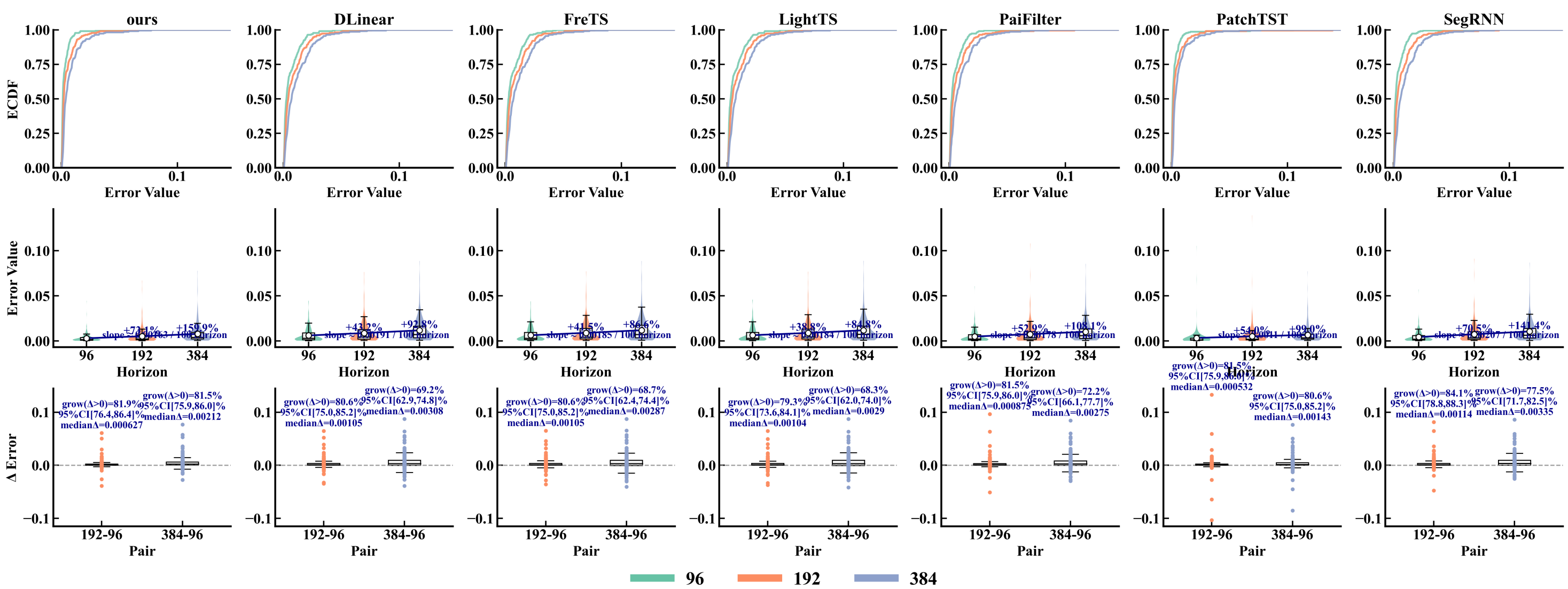

ISU-ILCC | RMSE — Tri-Horizon Compare Board

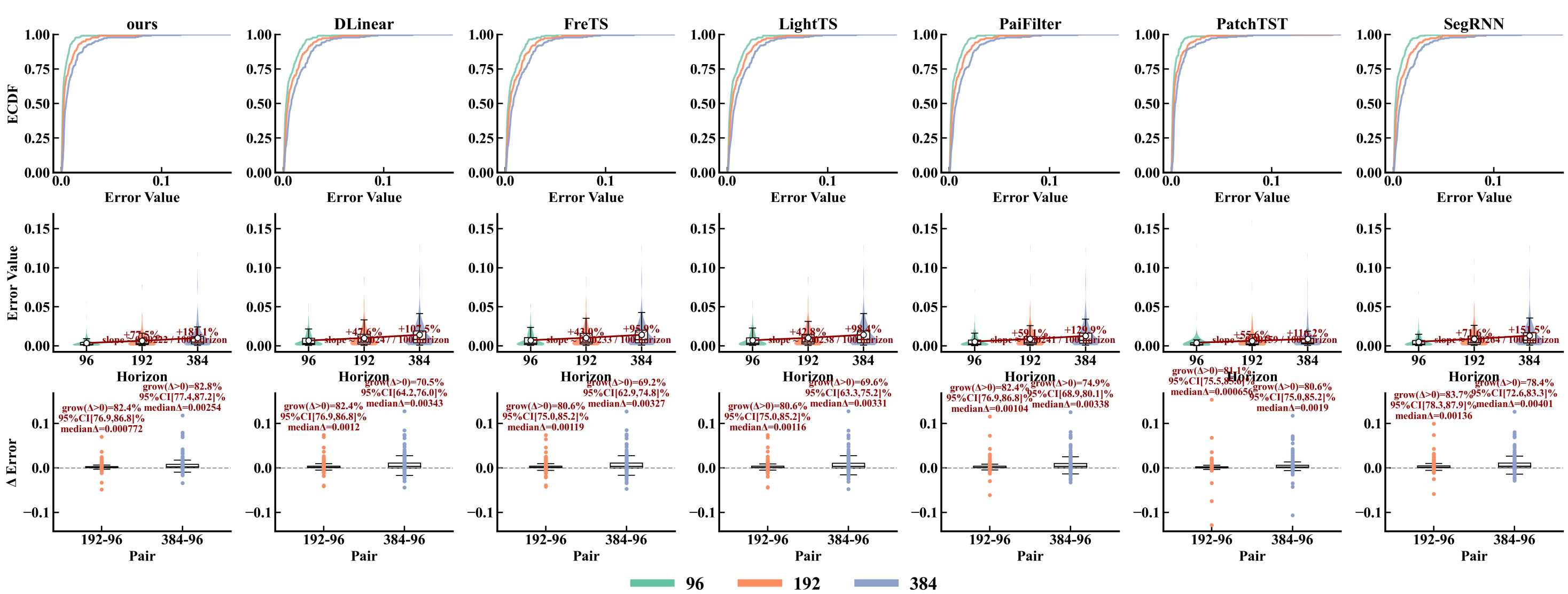

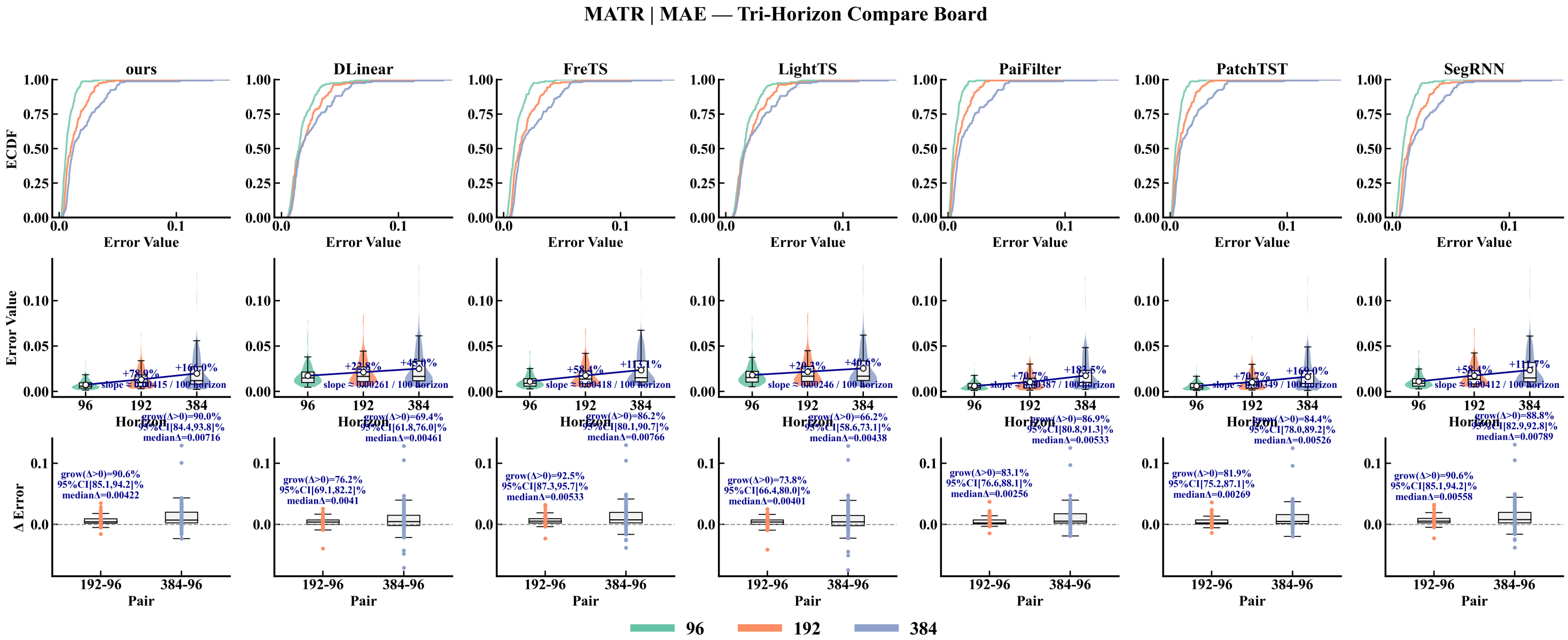

MATR | MAE — Tri-Horizon Compare Board
ours
DLinear
FreTS
LightTS
PaiFilter
PatchTST
SegRNN
ECDF
Error Value
Horizon
Δ Error
Pair
192-96
384-96
96
192
384

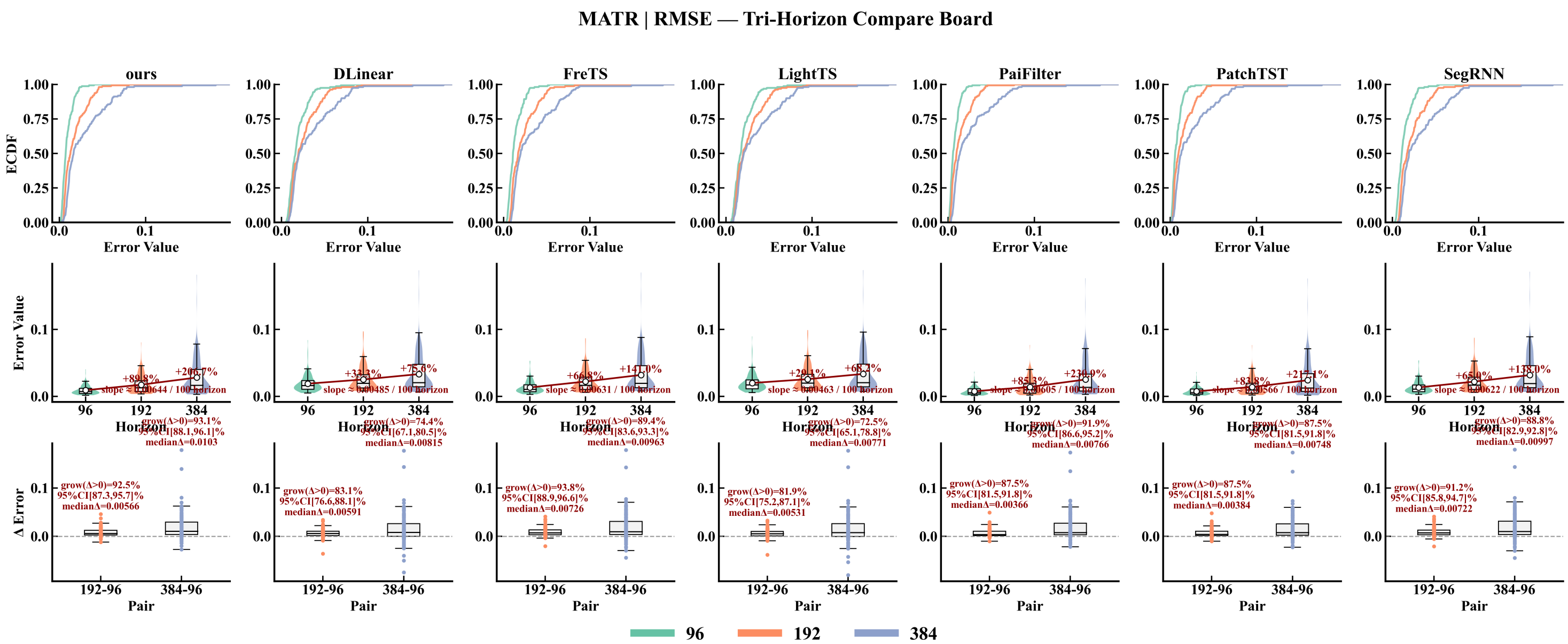

MATR | RMSE — Tri-Horizon Compare Board
ours
DLinear
FreTS
LightTS
PaiFilter
PatchTST
SegRNN
ECDF
Error Value
Horizon
Δ Error
Pair
192-96
384-96
96
192
384

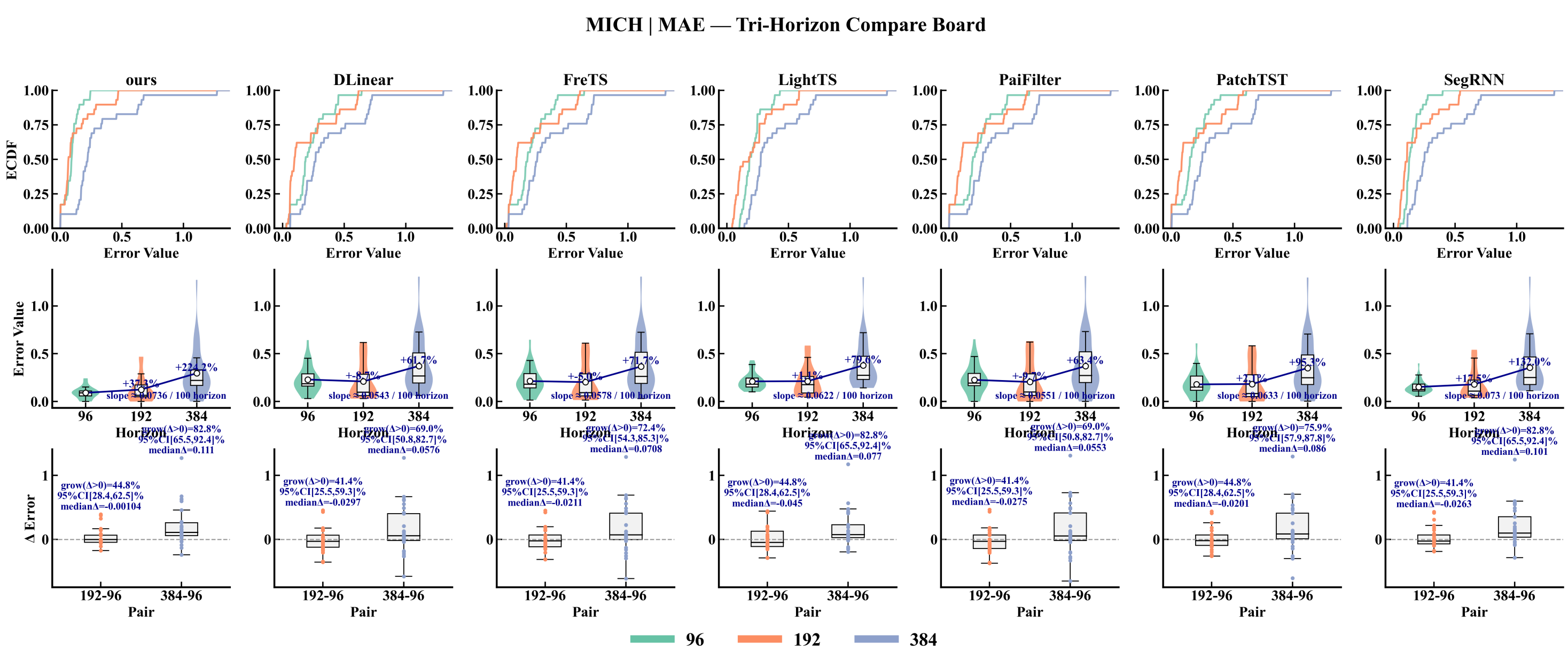

MICH | MAE — Tri-Horizon Compare Board
ours
DLinear
FreTS
LightTS
PaiFilter
PatchTST
SegRNN
ECDF
Error Value
Horizon
Δ Error
Pair
192-96
384-96
96
192
384

MICH | RMSE — Tri-Horizon Compare Board

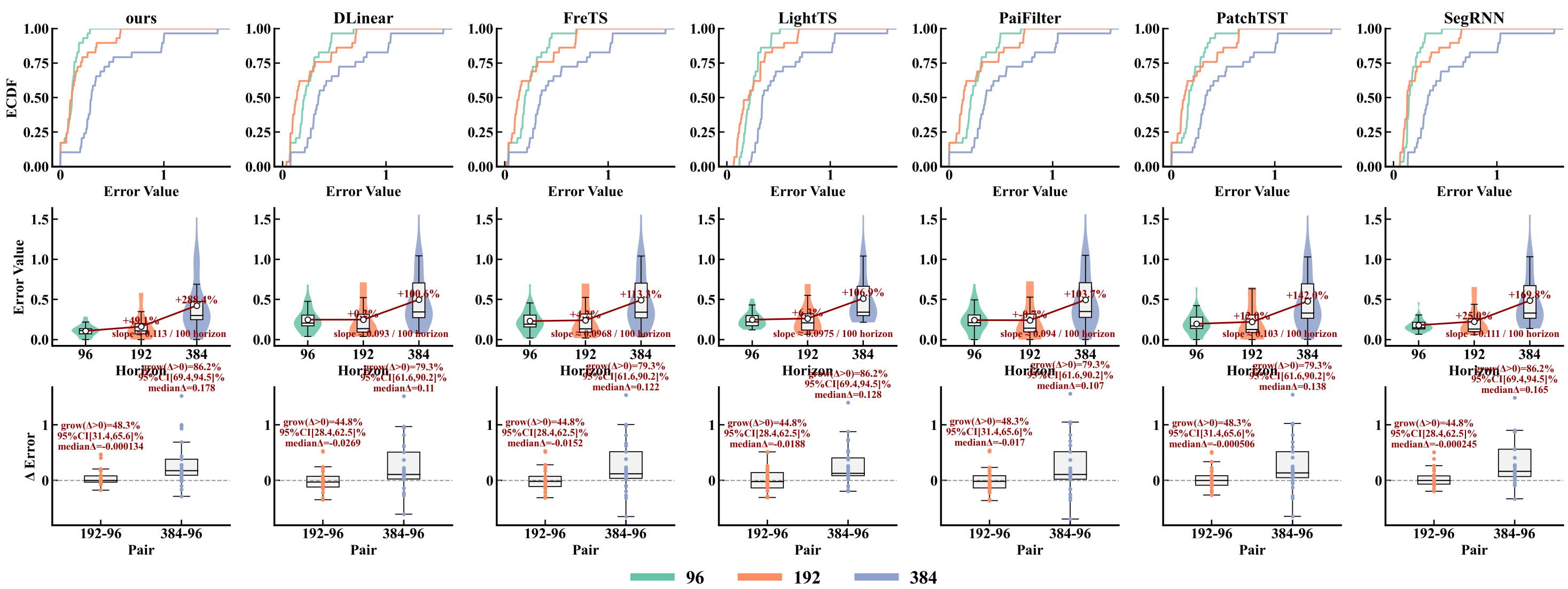

NA-ion | MAE — Tri-Horizon Compare Board

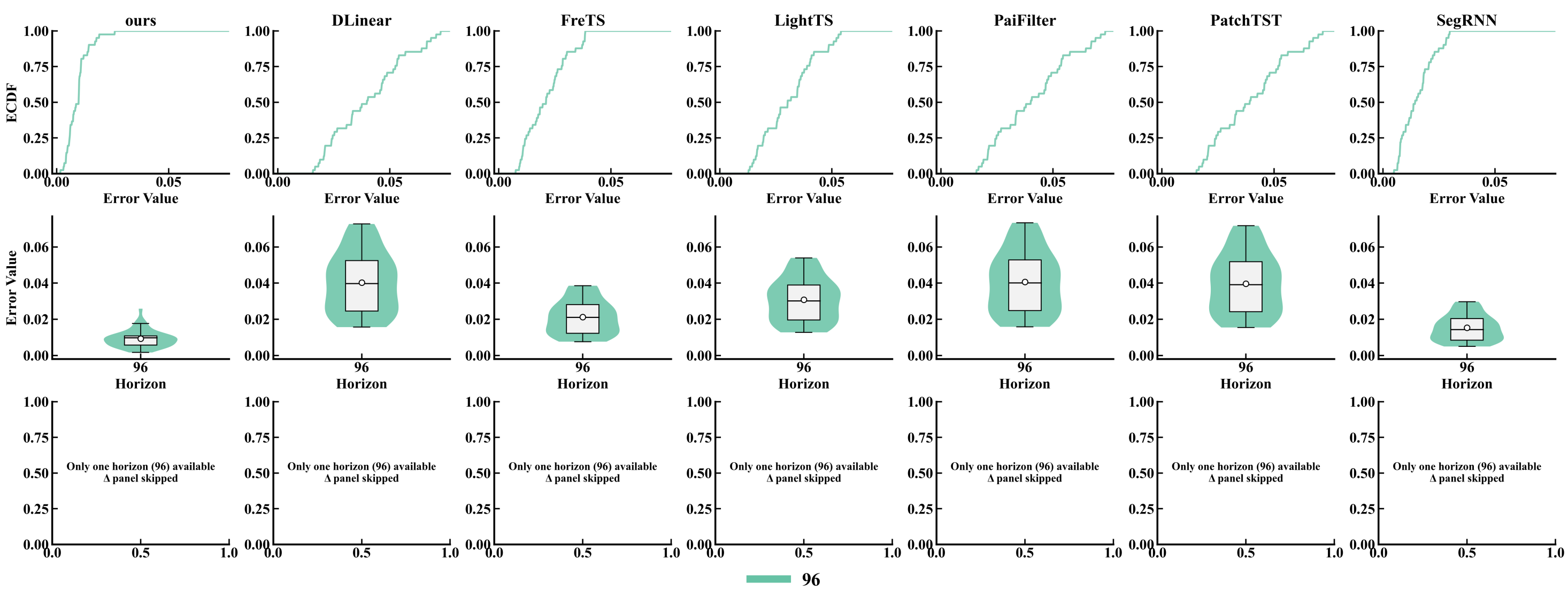

NA-ion | RMSE — Tri-Horizon Compare Board

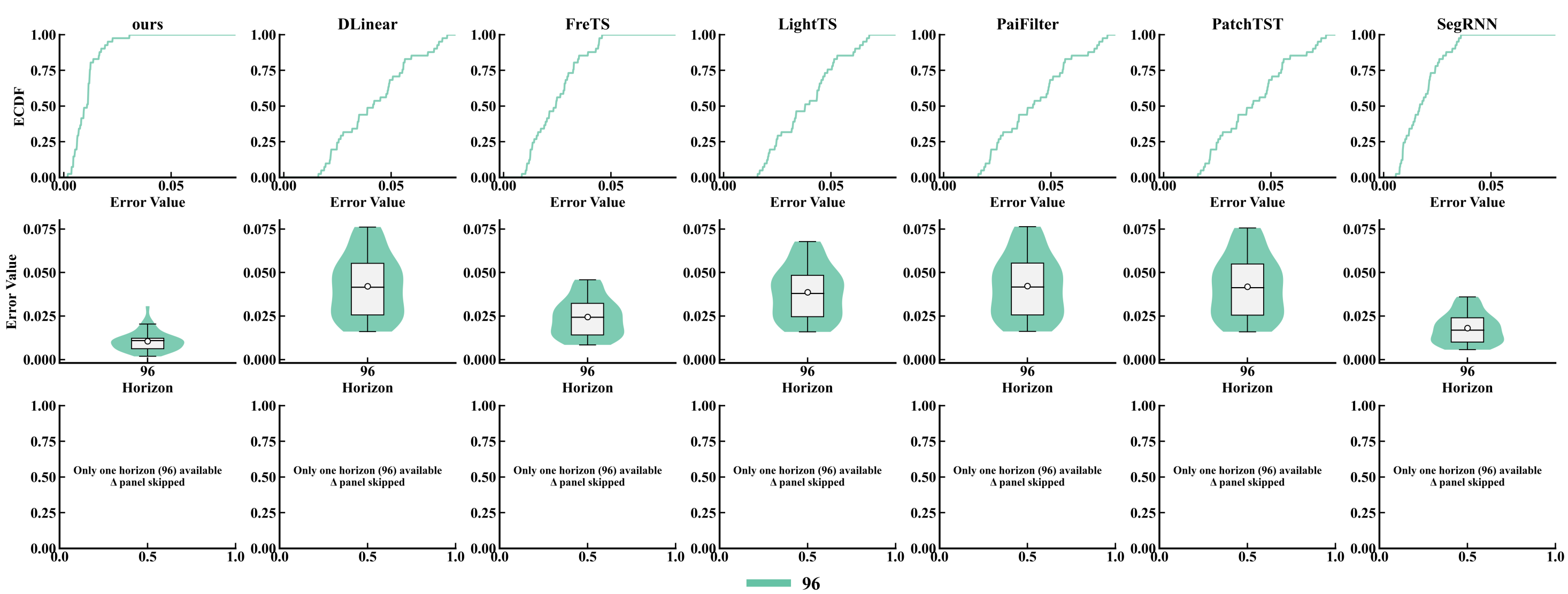

RWTH | MAE — Tri-Horizon Compare Board

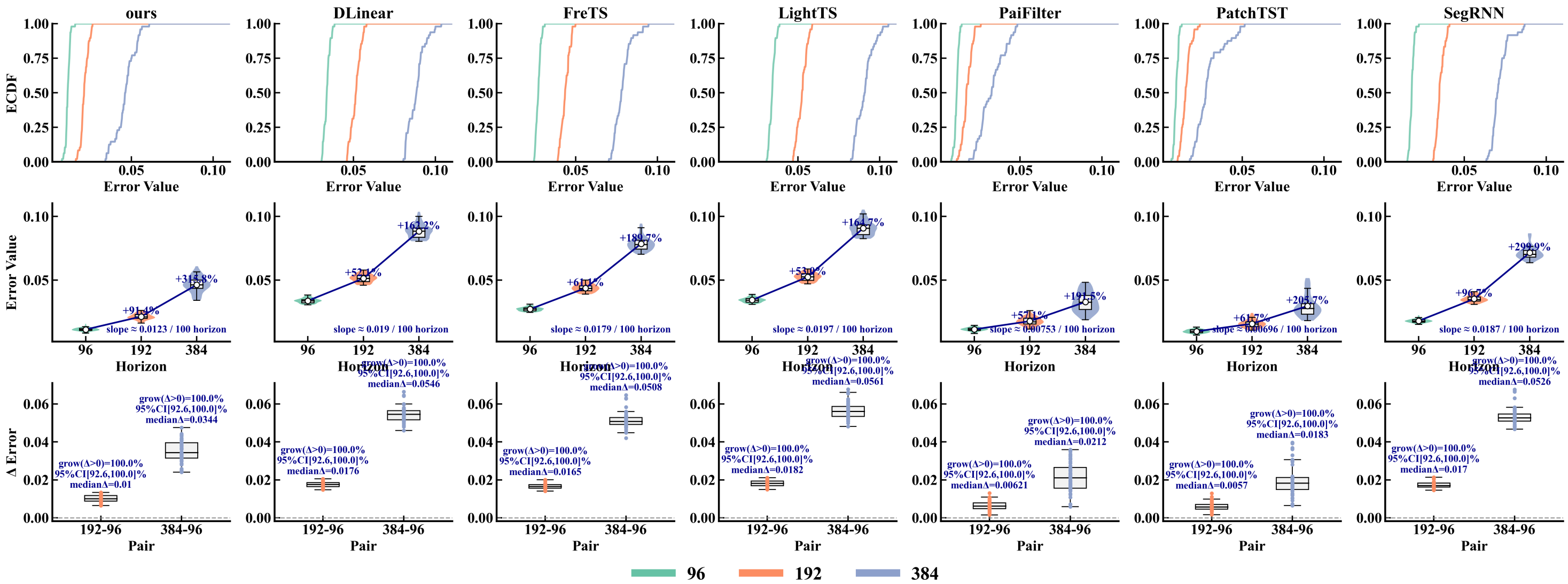

RWTH | RMSE — Tri-Horizon Compare Board

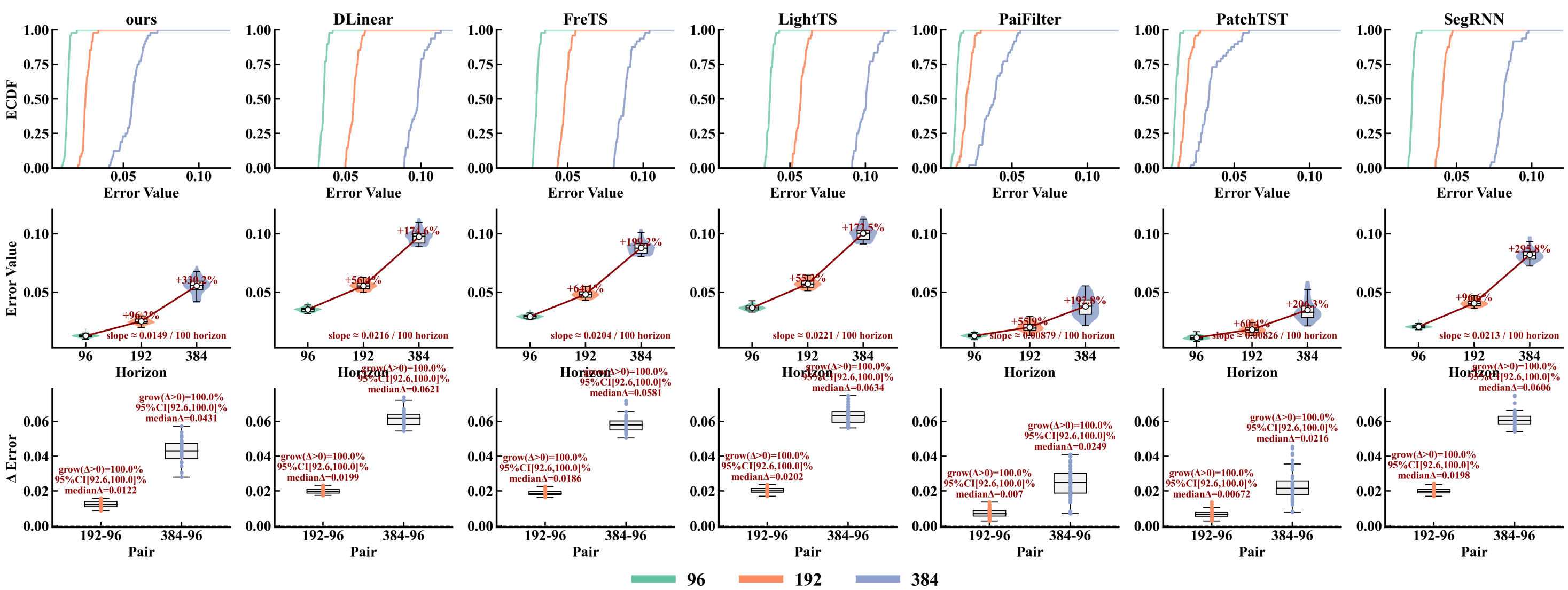

SJTU | MAE — Tri-Horizon Compare Board

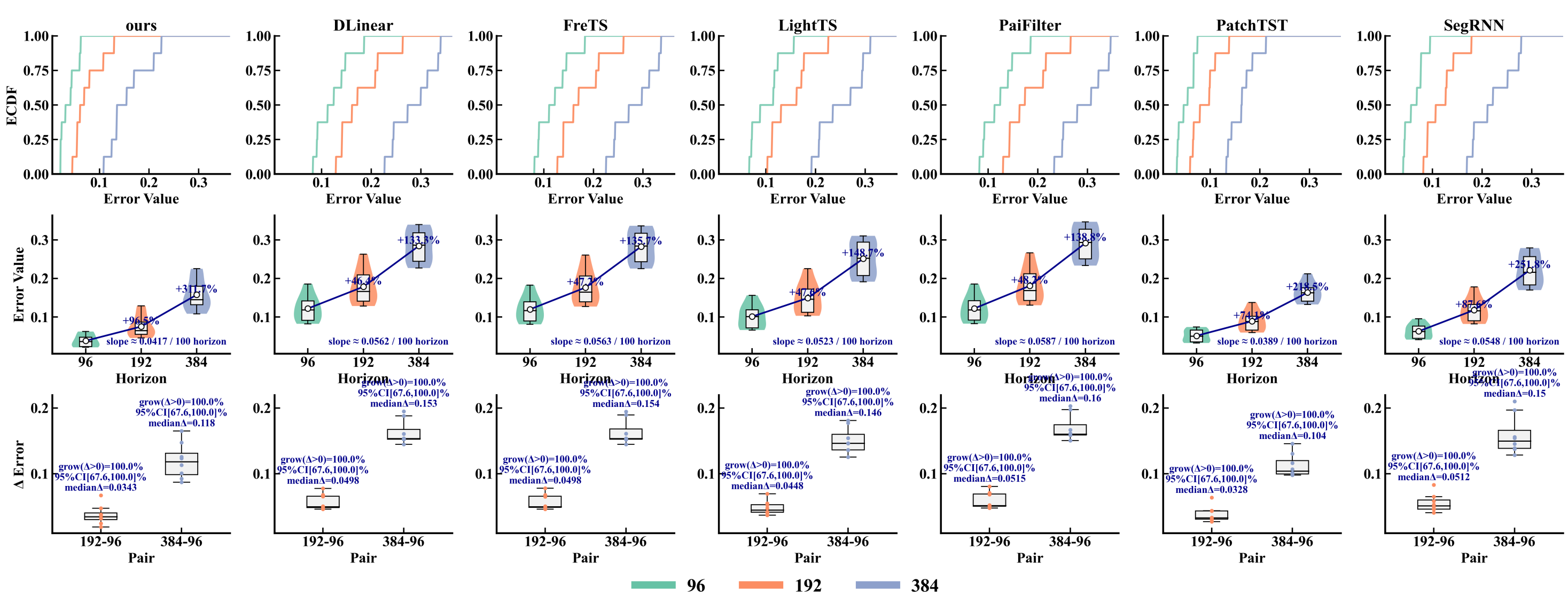

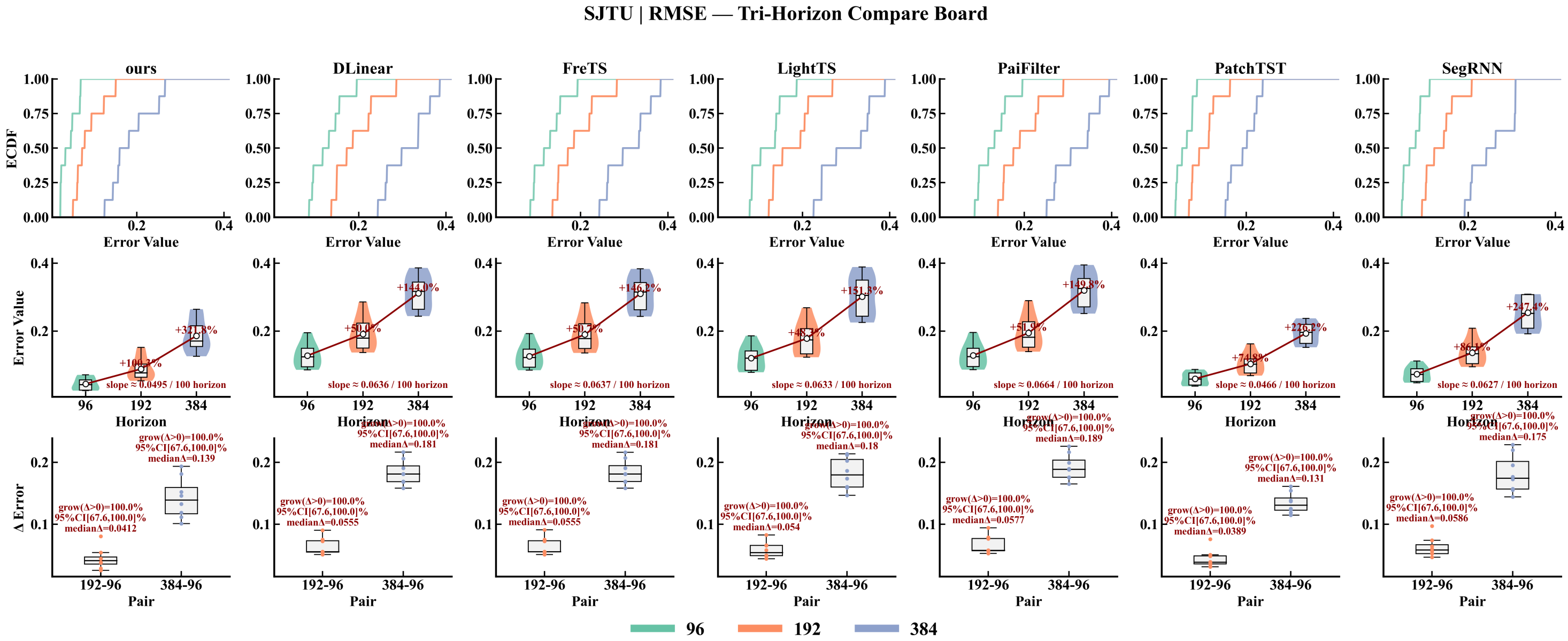

SJTU | RMSE — Tri-Horizon Compare Board
ours
DLinear
FreTS
LightTS
PaiFilter
PatchTST
SegRNN
ECDF
Error Value
Horizon
Δ Error
Pair
96
192
384

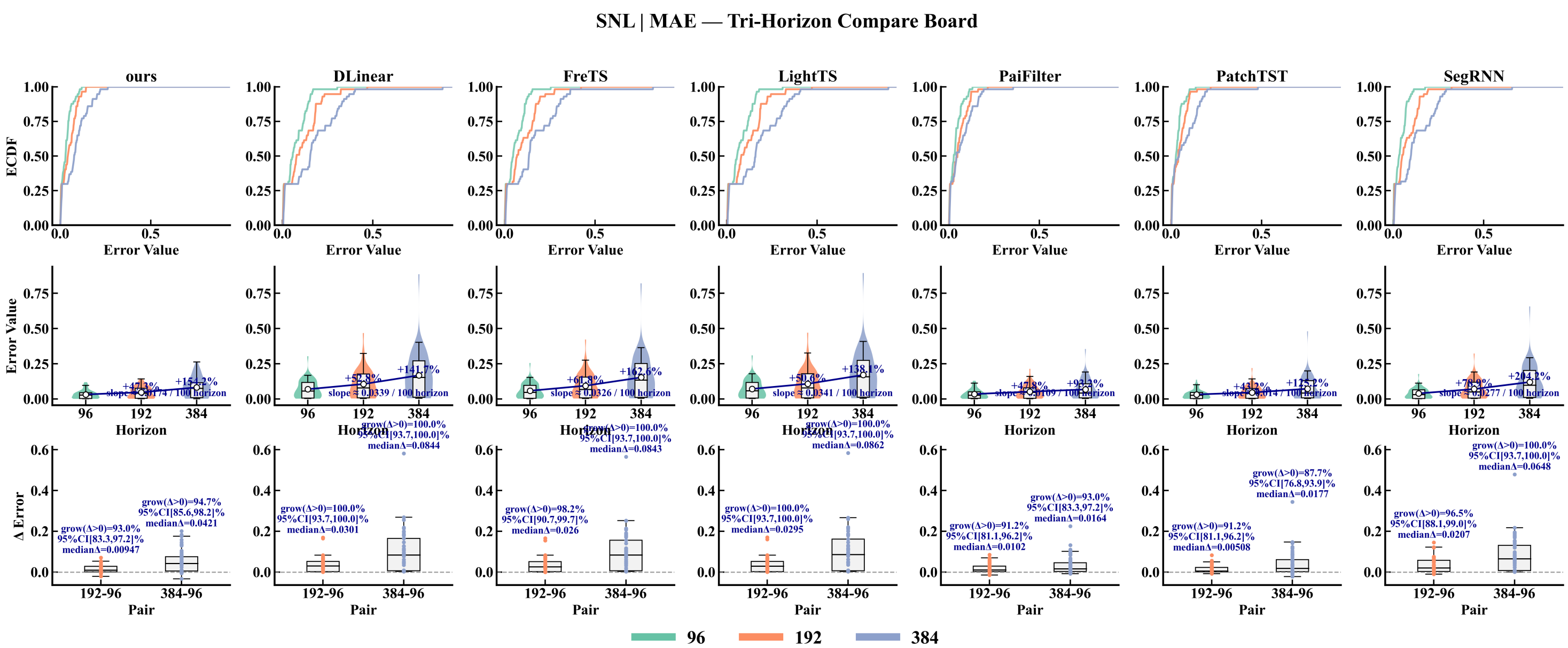

SNL | MAE — Tri-Horizon Compare Board
ours
DLinear
FreTS
LightTS
PaiFilter
PatchTST
SegRNN
ECDF
Error Value
Horizon
Δ Error
Pair
96
192
384

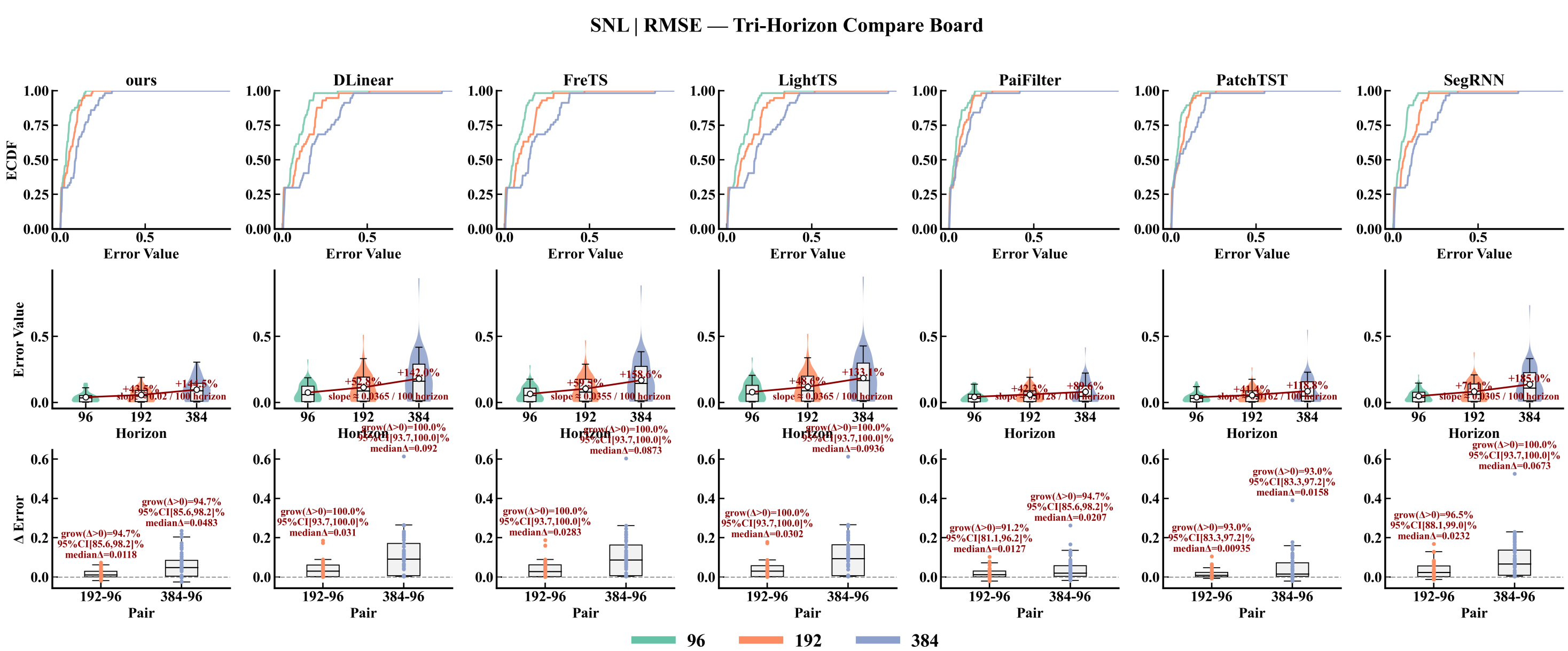

SNL | RMSE — Tri-Horizon Compare Board
ours
DLinear
FreTS
LightTS
PaiFilter
PatchTST
SegRNN
ECDF
Error Value
Horizon
Δ Error
Pair
96
192
384

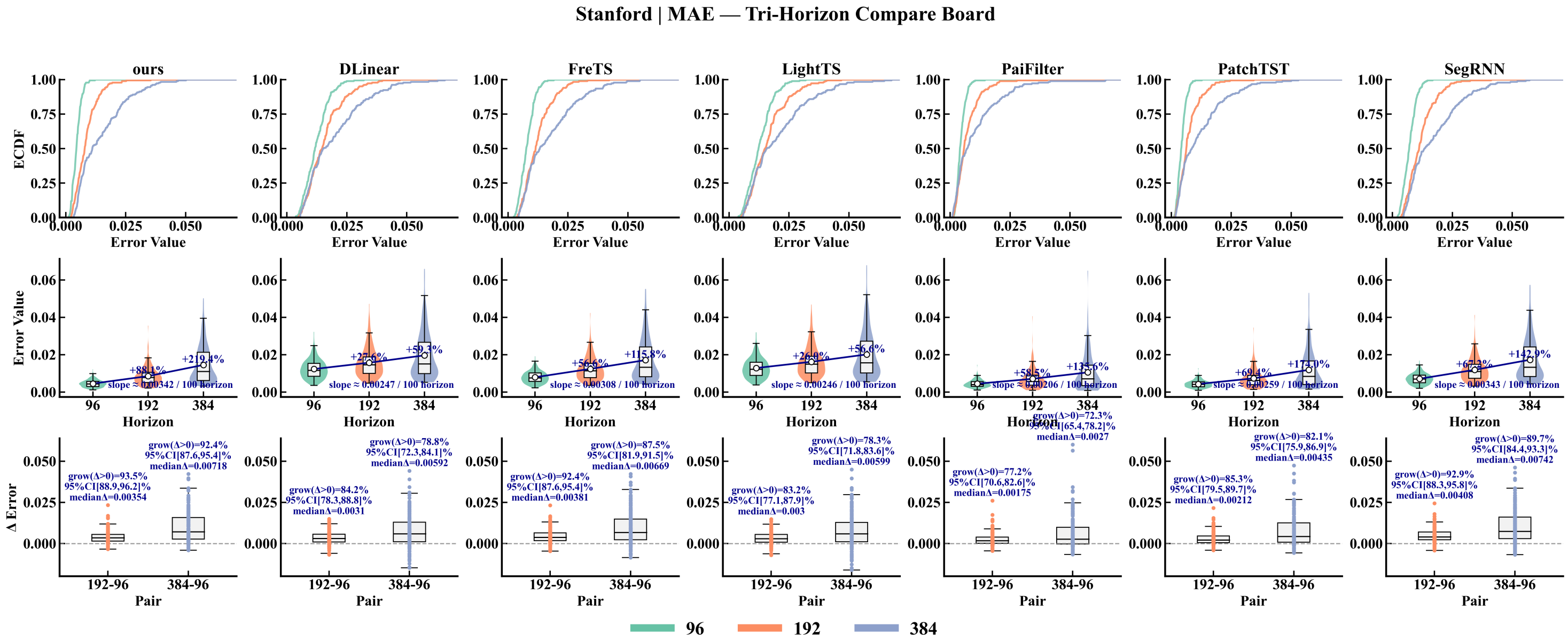
Stanford | MAE — Tri-Horizon Compare Board
ours
DLinear
FreTS
LightTS
PaiFilter
PatchTST
SegRNN
ECDF
Error Value
Horizon
Δ Error
Pair
96
192
384

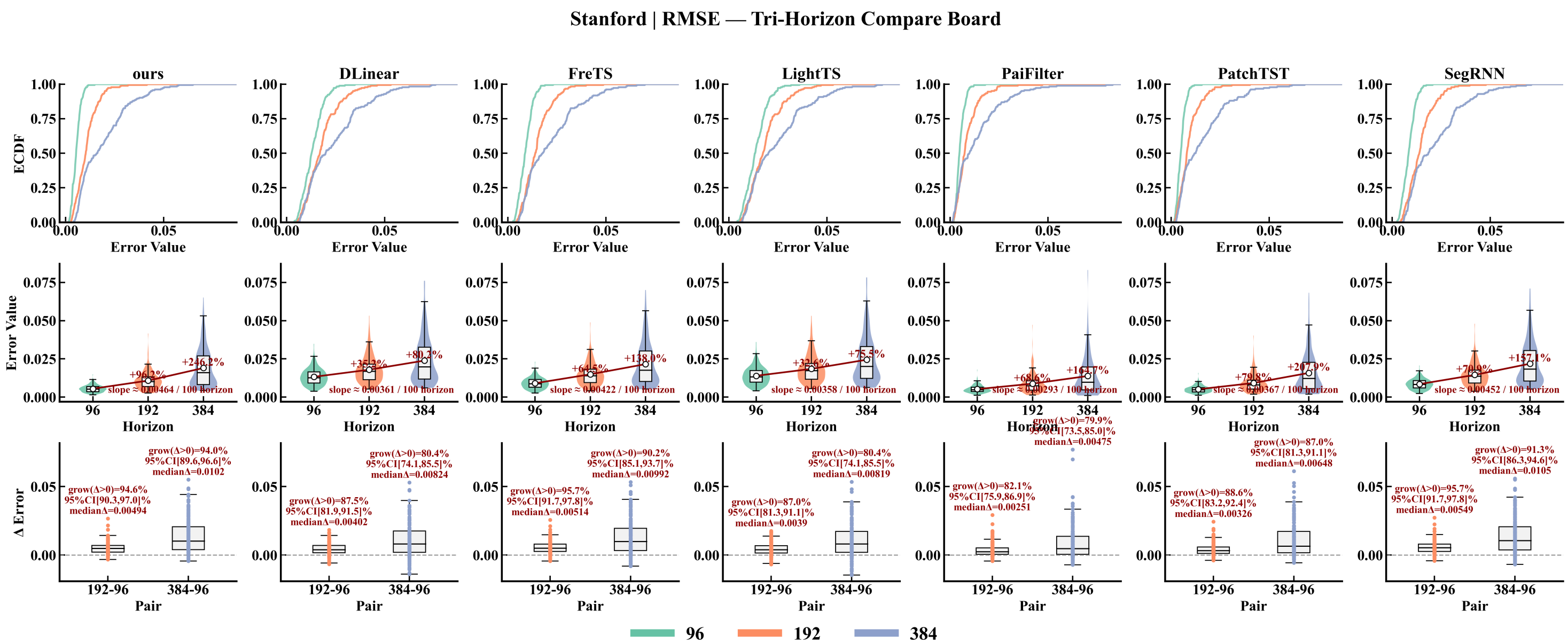
Stanford | RMSE — Tri-Horizon Compare Board
ours
DLinear
FreTS
LightTS
PaiFilter
PatchTST
SegRNN
ECDF
Error Value
Horizon
Δ Error
Pair
96
192
384

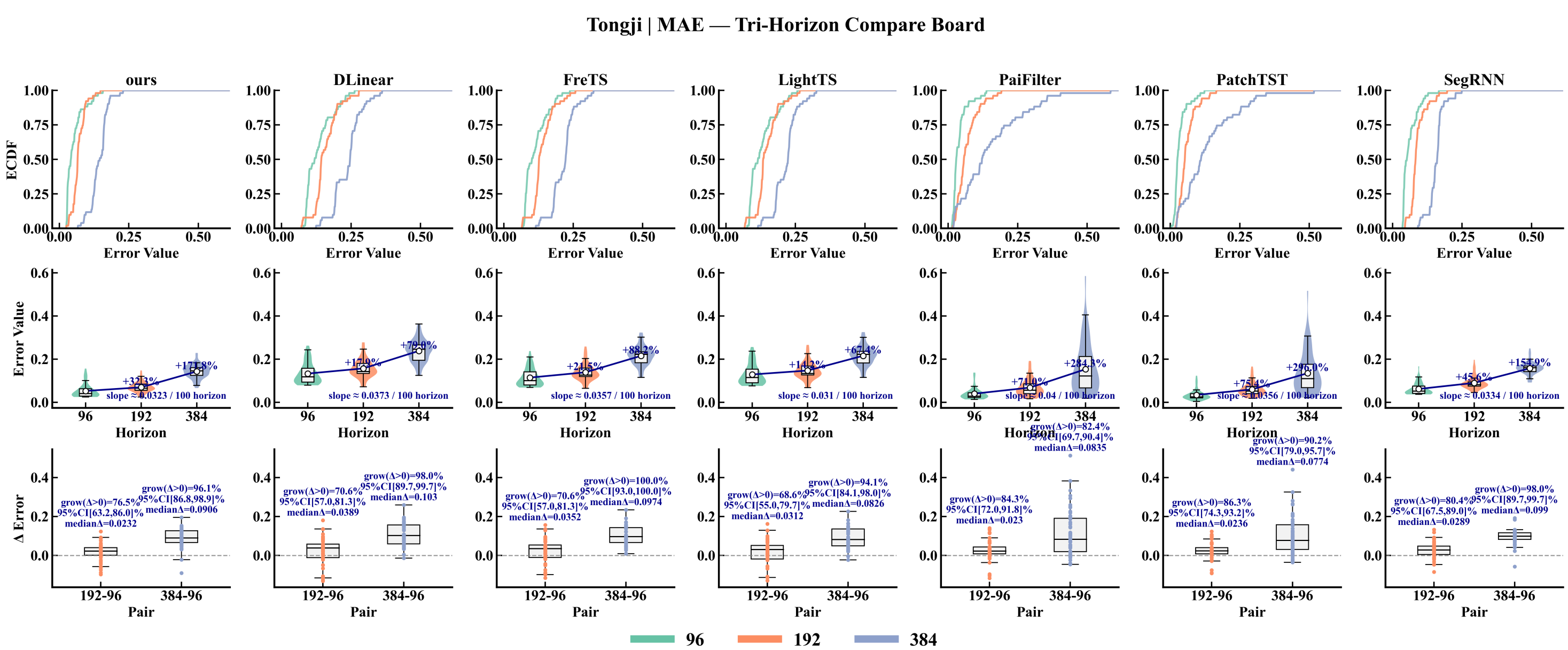
Tongji | MAE — Tri-Horizon Compare Board
ours
DLinear
FreTS
LightTS
PaiFilter
PatchTST
SegRNN
ECDF
Error Value
Horizon
Δ Error
Pair
96
192
384

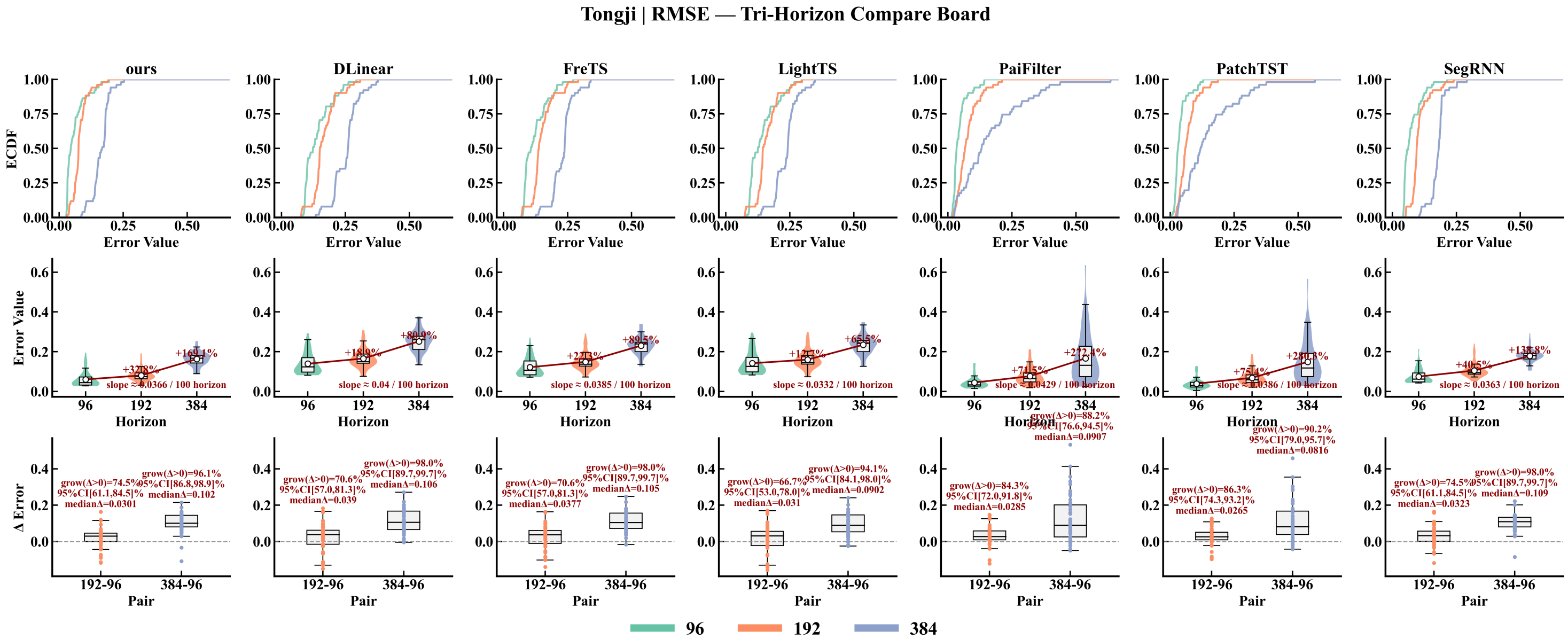
Tongji | RMSE — Tri-Horizon Compare Board
ours
DLinear
FreTS
LightTS
PaiFilter
PatchTST
SegRNN
ECDF
Error Value
Horizon
Δ Error
Pair
96
192
384

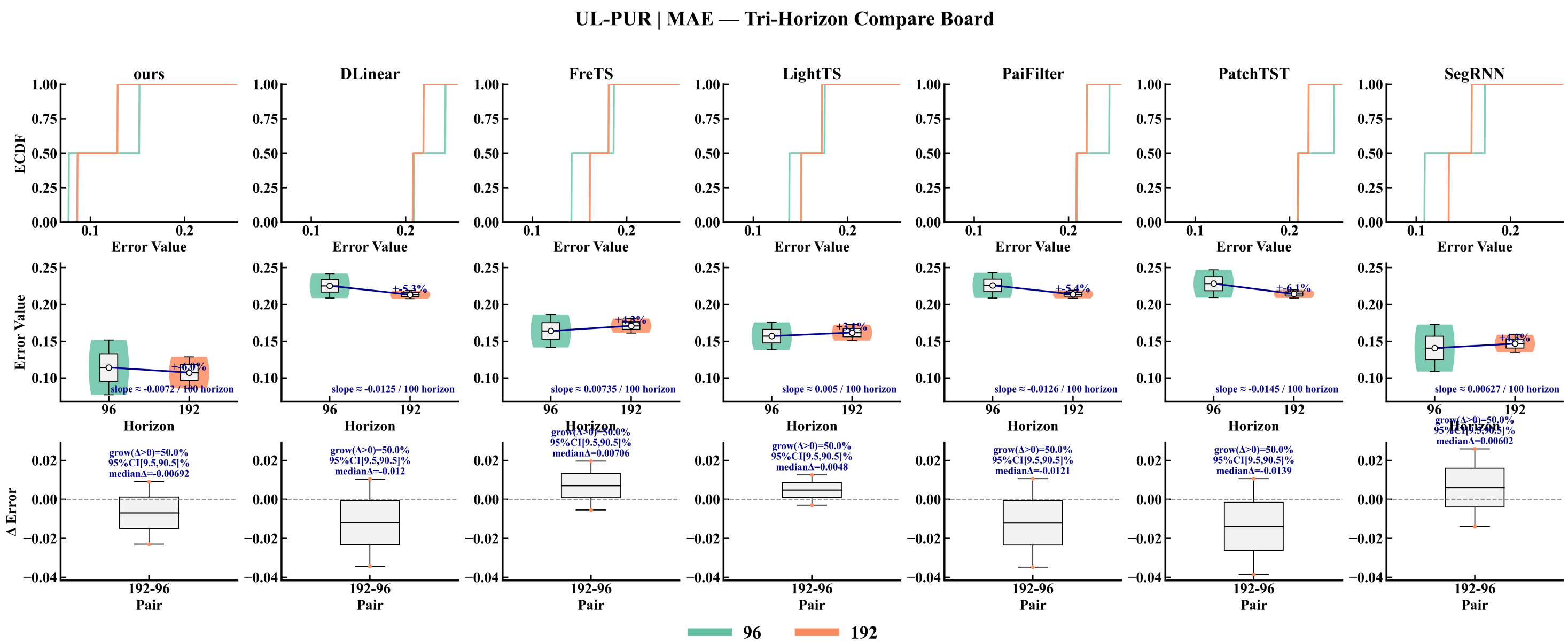
UL-PUR | MAE — Tri-Horizon Compare Board
ours
DLinear
FreTS
LightTS
PaiFilter
PatchTST
SegRNN
ECDF
Error Value
Horizon
Δ Error
Pair
96
192

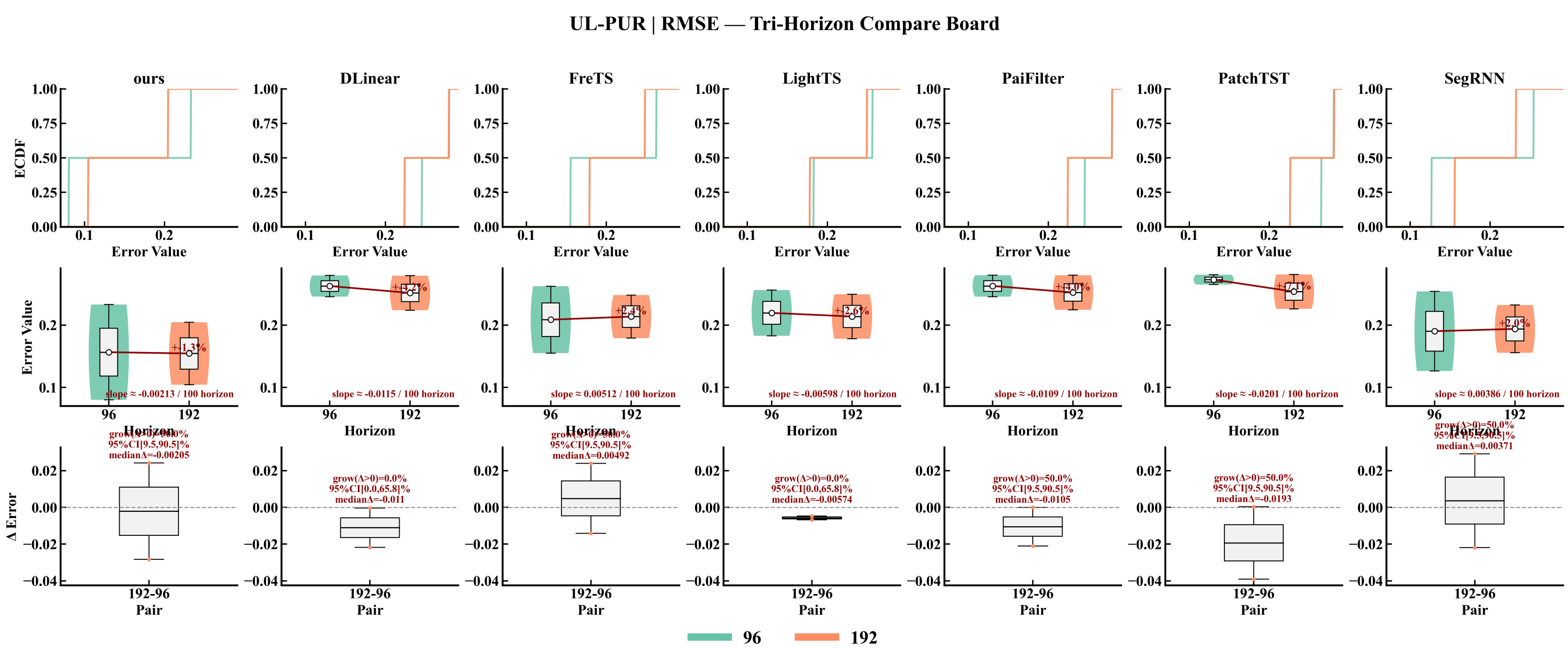
UL-PUR | RMSE — Tri-Horizon Compare Board
ours
DLinear
FreTS
LightTS
PaiFilter
PatchTST
SegRNN
ECDF
Error Value
Horizon
Δ Error
Pair
96
192

WZU | MAE — Tri-Horizon Compare Board

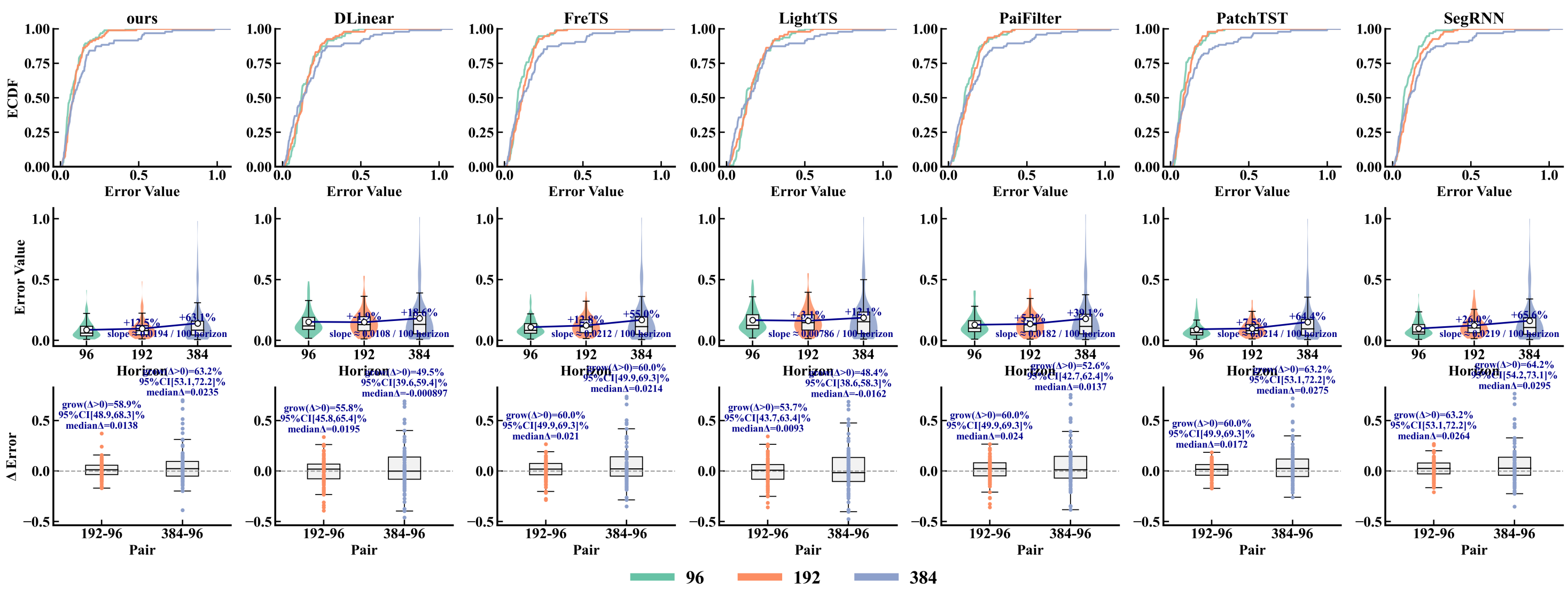

WZU | RMSE — Tri-Horizon Compare Board

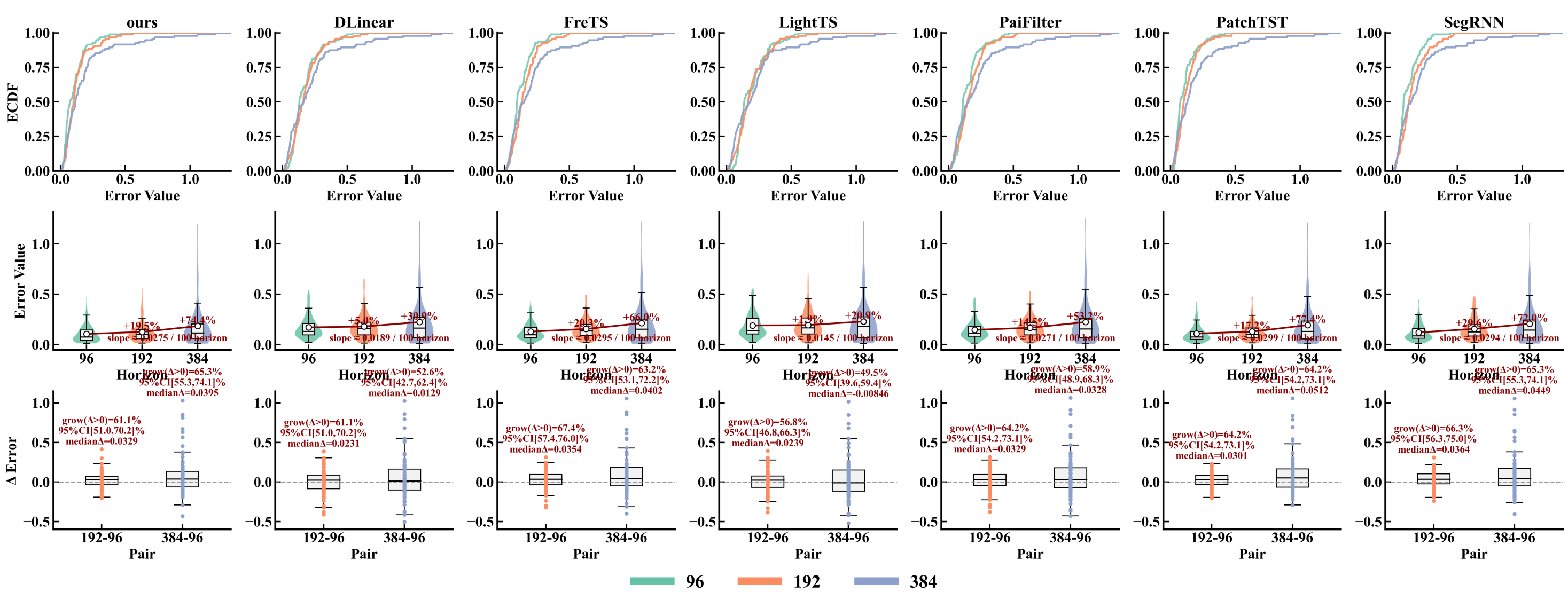

XJTU | MAE — Tri-Horizon Compare Board

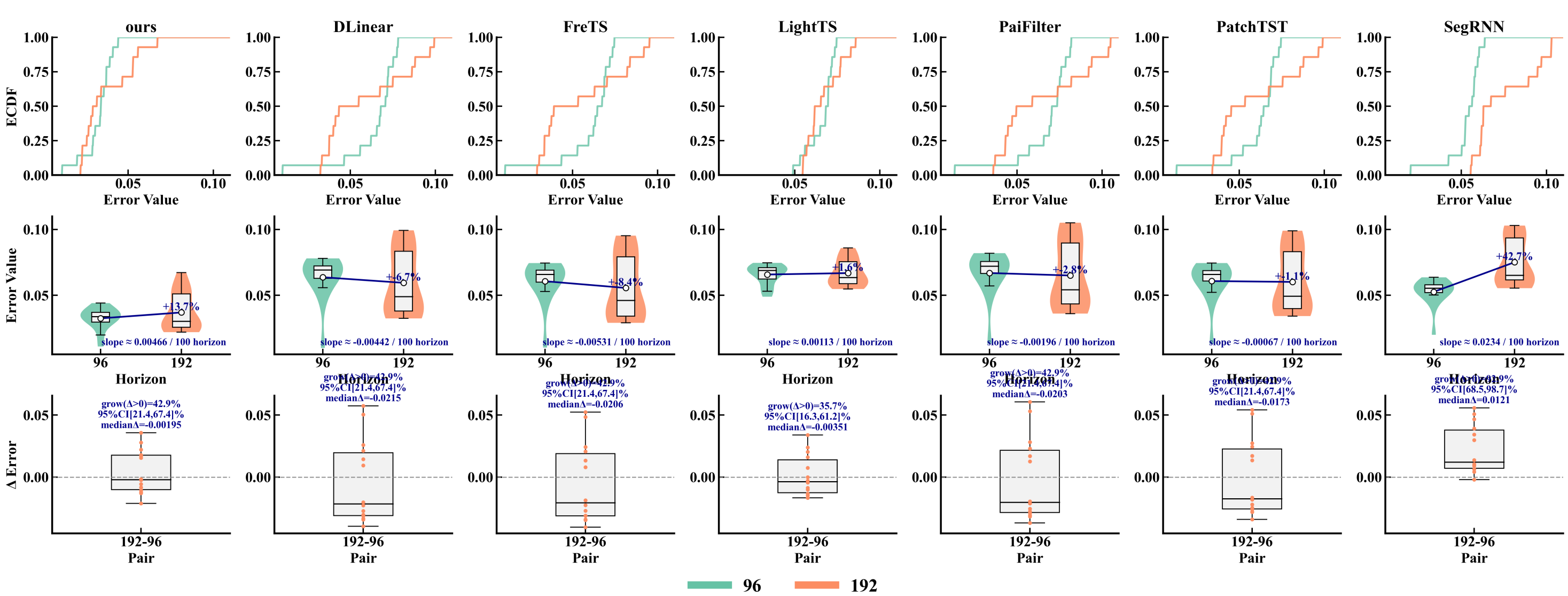

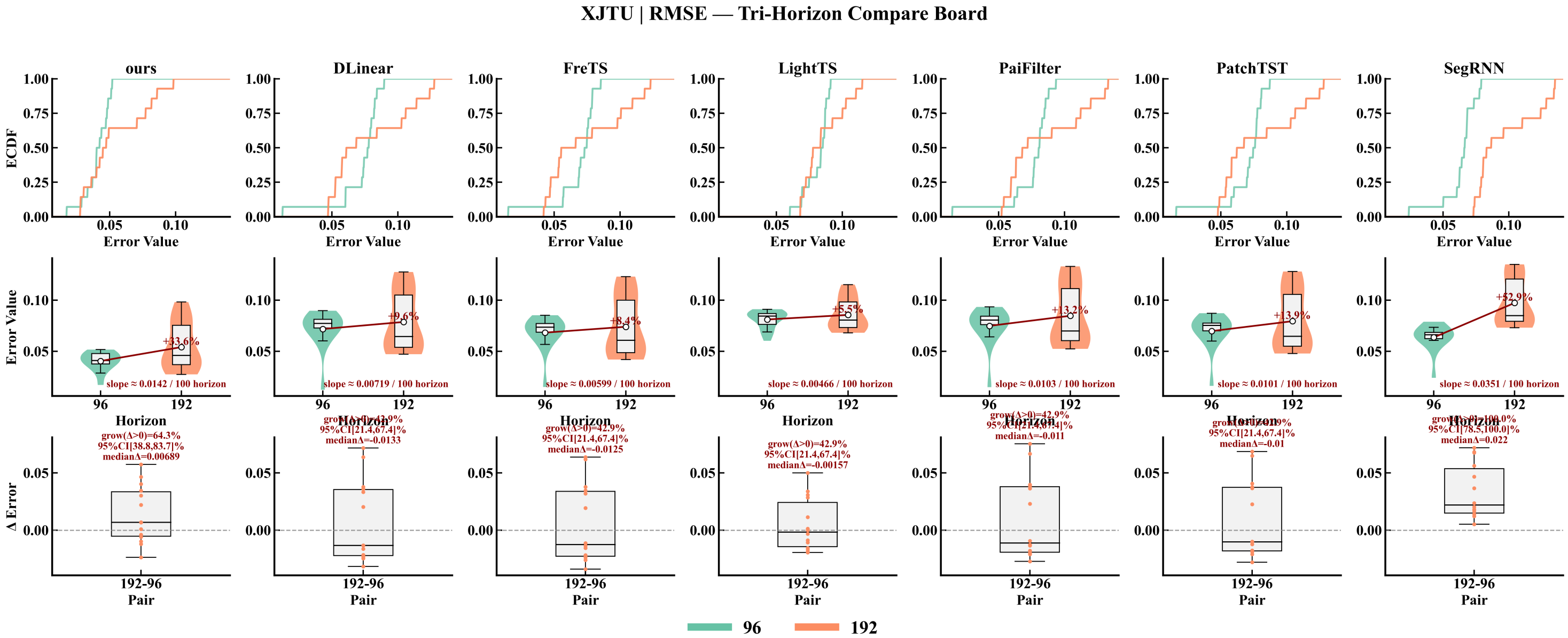
XJTU | RMSE — Tri-Horizon Compare Board
ours
DLinear
FreTS
LightTS
PaiFilter
PatchTST
SegRNN
96
192

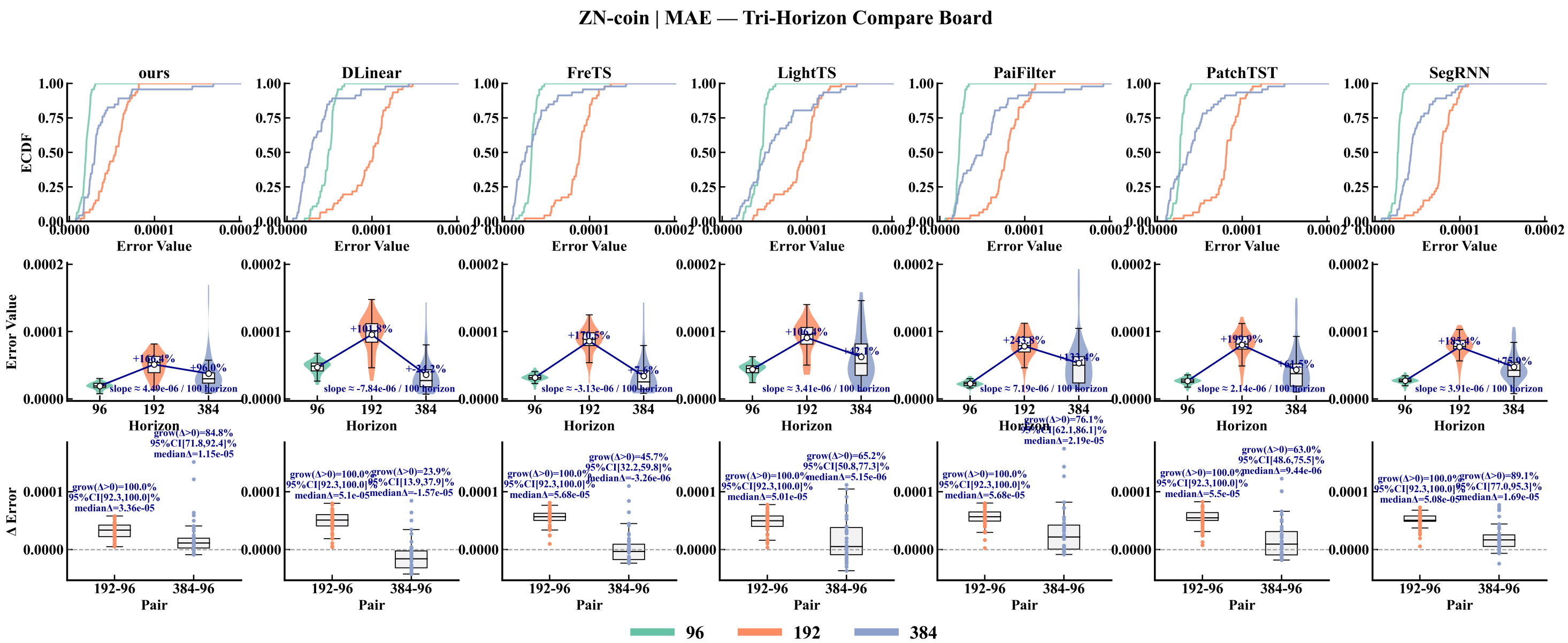
ZN-coin | MAE — Tri-Horizon Compare Board
ours
DLinear
FreTS
LightTS
PaiFilter
PatchTST
SegRNN
96
192
384

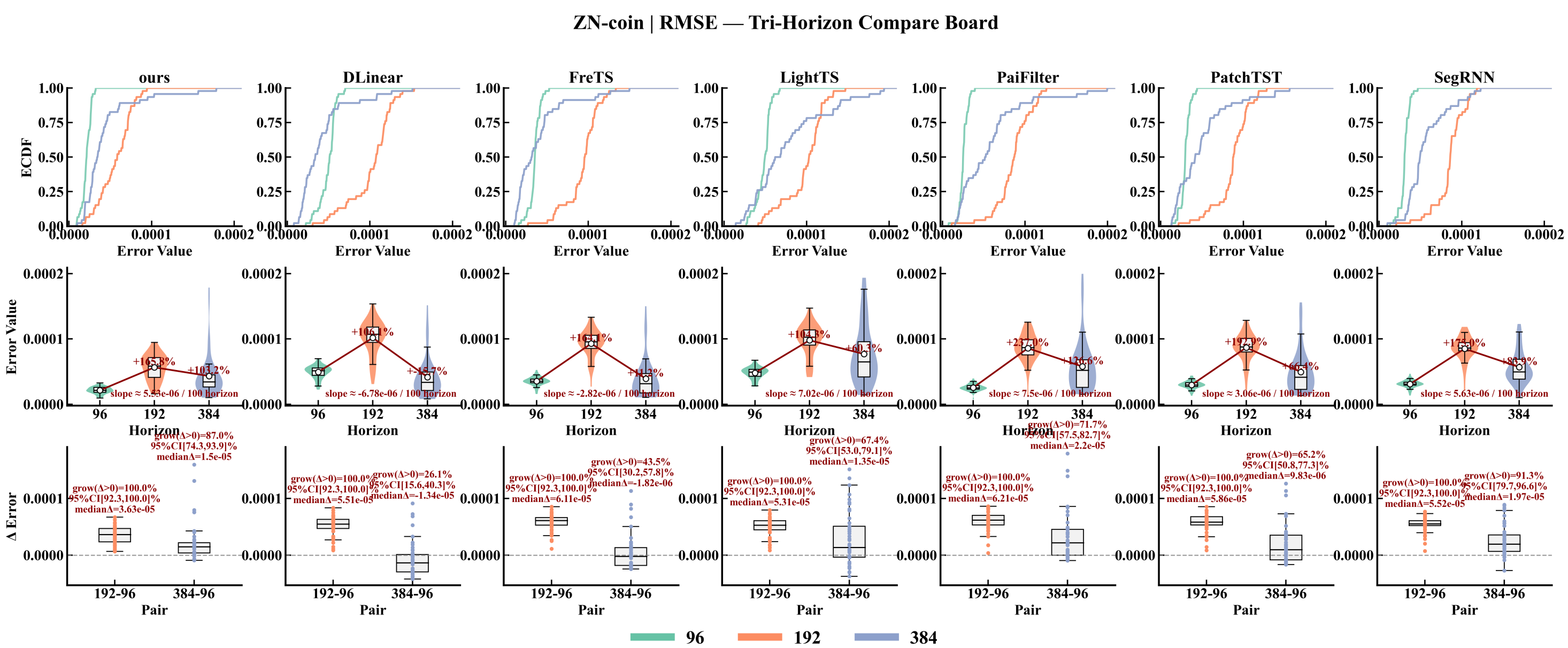
ZN-coin | RMSE — Tri-Horizon Compare Board
ours
DLinear
FreTS
LightTS
PaiFilter
PatchTST
SegRNN
96
192
384

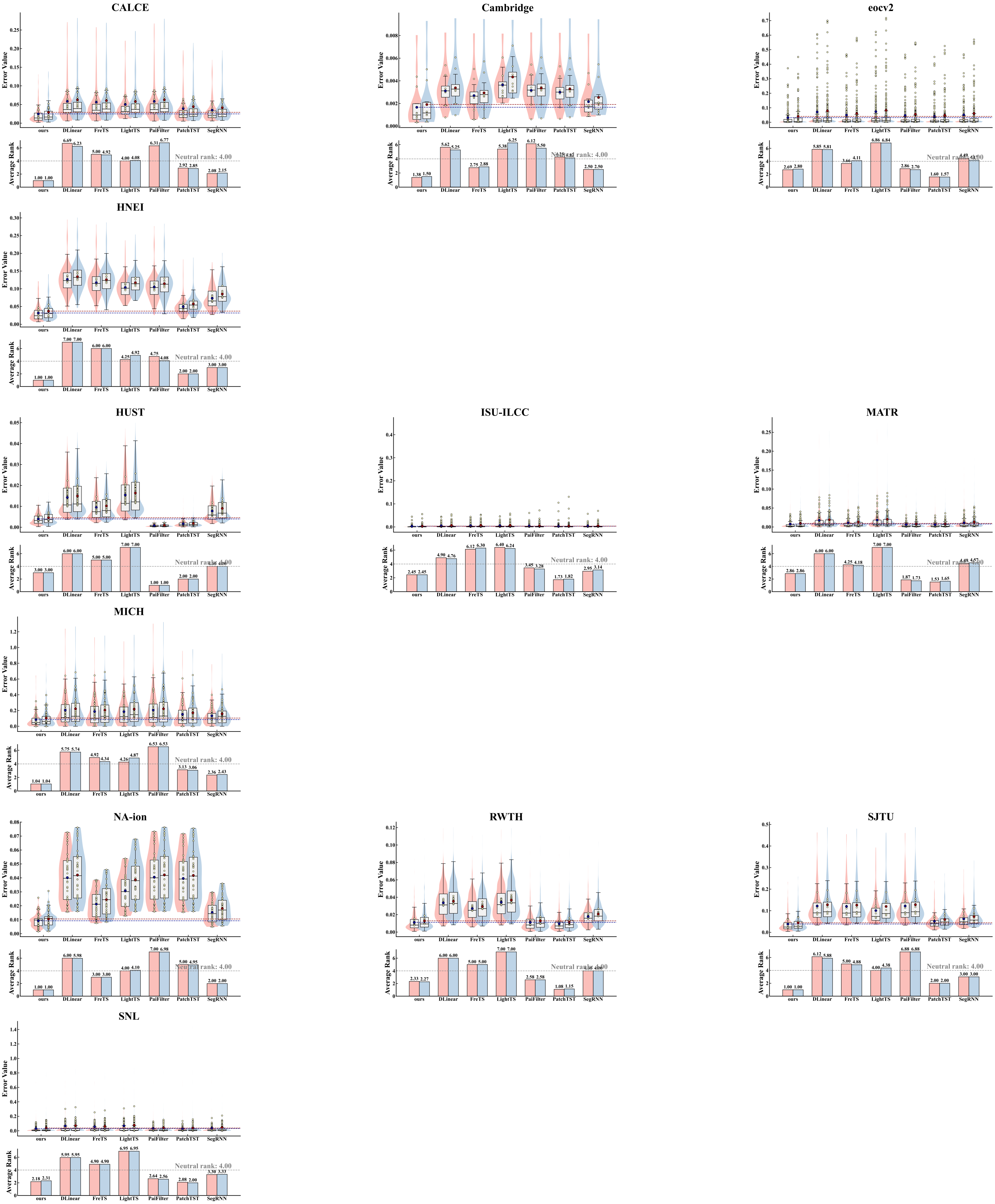
CALCE
Cambridge
eocv2
HNEI
HUST
ISU-ILCC
MATR
MICH
NA-ion
RWTH
SJTU
SNL
Error Value
Average Rank
Neutral rank: 4.00
ours
DLinear
FreTS
LightTS
PaiFilter
PatchTST
SegRNN

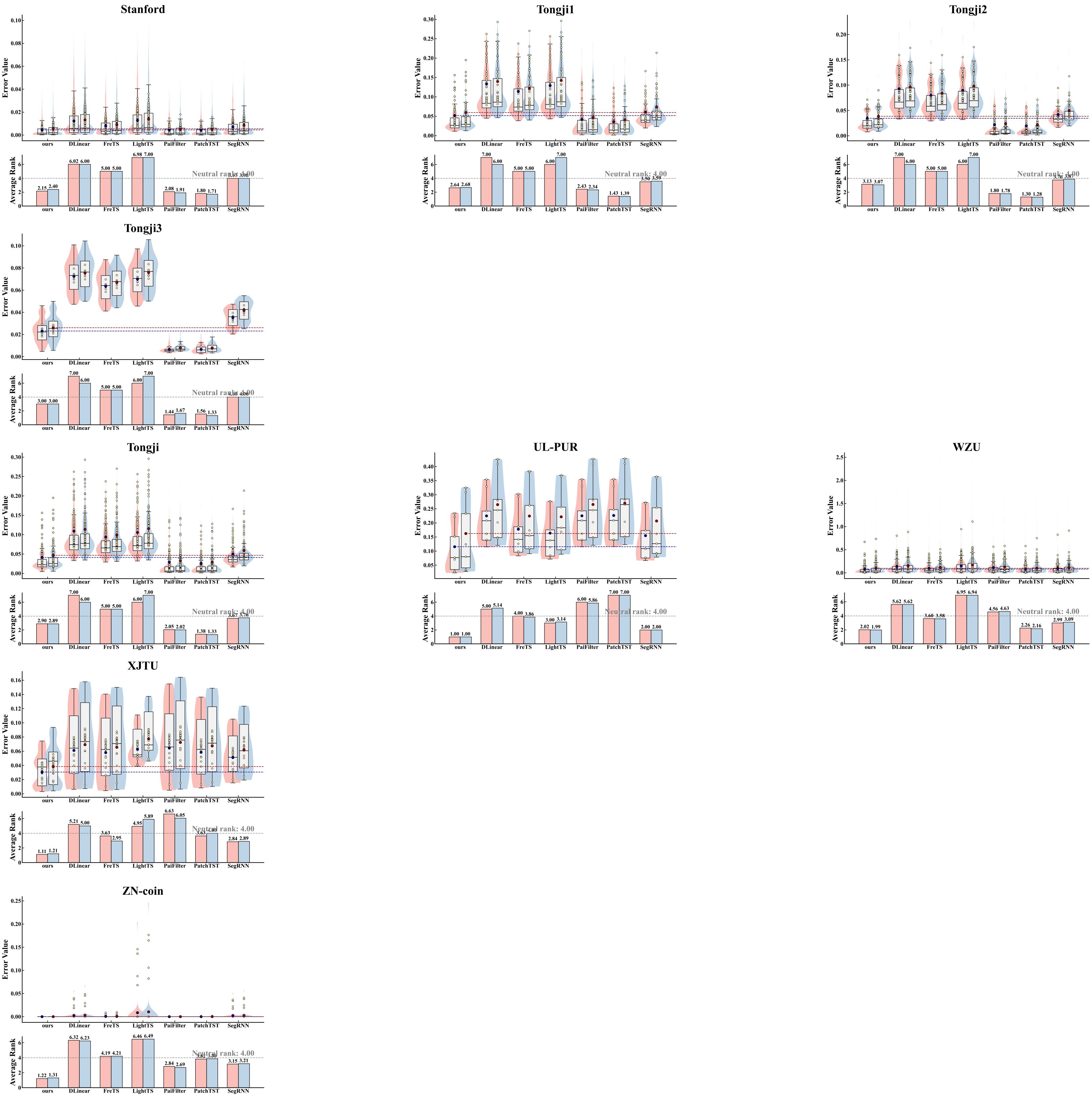

Stanford
Tongji1
Tongji2
Tongji3
Tongji
UL-PUR
WZU
XJTU
ZN-coin
Error Value
Average Rank
ours
DLinear
FreTS
LightTS
PaiFilter
PatchTST
SegRNN
Neutral rank: 4.00
2.15 2.40 6.02 6.00 5.00 5.00 6.98 7.00 2.08 1.91 1.80 1.71
2.64 2.68 7.00 6.00 5.00 5.00 6.00 7.00 2.43 2.34 1.43 1.39 3.50 3.59
3.13 3.07 7.00 6.00 5.00 5.00 6.00 7.00 1.80 1.78 1.30 1.28
3.00 3.00 7.00 6.00 5.00 5.00 6.00 7.00 1.44 1.67 1.56 1.33
2.90 2.89 7.00 6.00 5.00 5.00 6.00 7.00 2.05 2.02 1.38 1.33
1.00 1.00 5.00 5.14 4.00 3.86 3.00 3.14 6.00 5.86 7.00 7.00 2.00 2.00
2.02 1.99 5.62 5.62 3.60 3.58 6.95 6.94 4.56 4.63 2.26 2.16 2.99 3.09
1.11 1.21 5.21 5.00 3.63 2.95 4.95 5.89 6.63 6.05 2.84 2.89
1.22 1.31 6.32 6.23 4.19 4.21 6.46 6.49 2.84 2.69 3.15 3.21

192

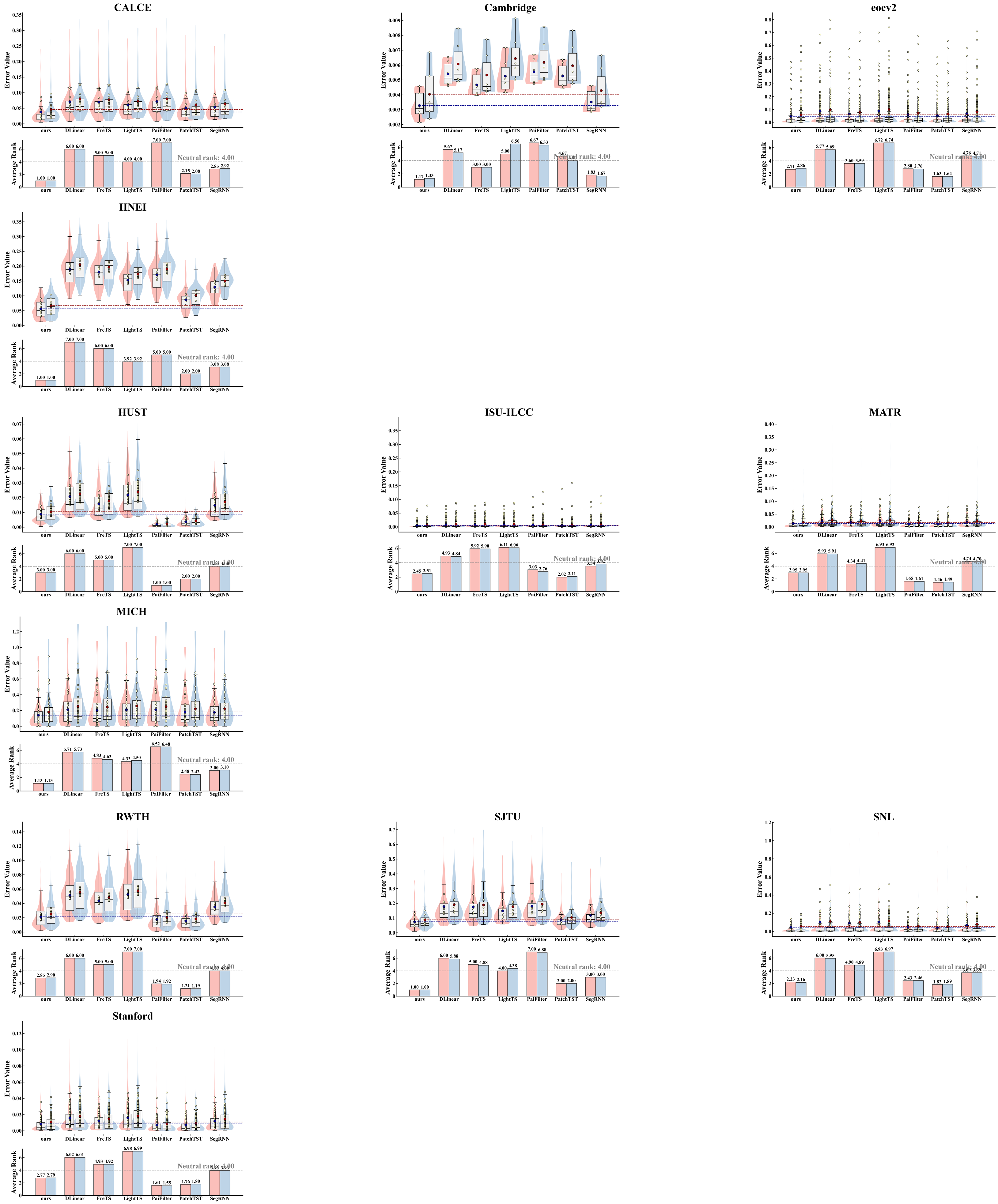

CALCE
Cambridge
eocv2
HNEI
HUST
ISU-ILCC
MATR
MICH
RWTH
SJTU
SNL
Stanford
Error Value
Average Rank
ours
DLinear
FreTS
LightTS
PaiFilter
PatchTST
SegRNN
Neutral rank: 4.00

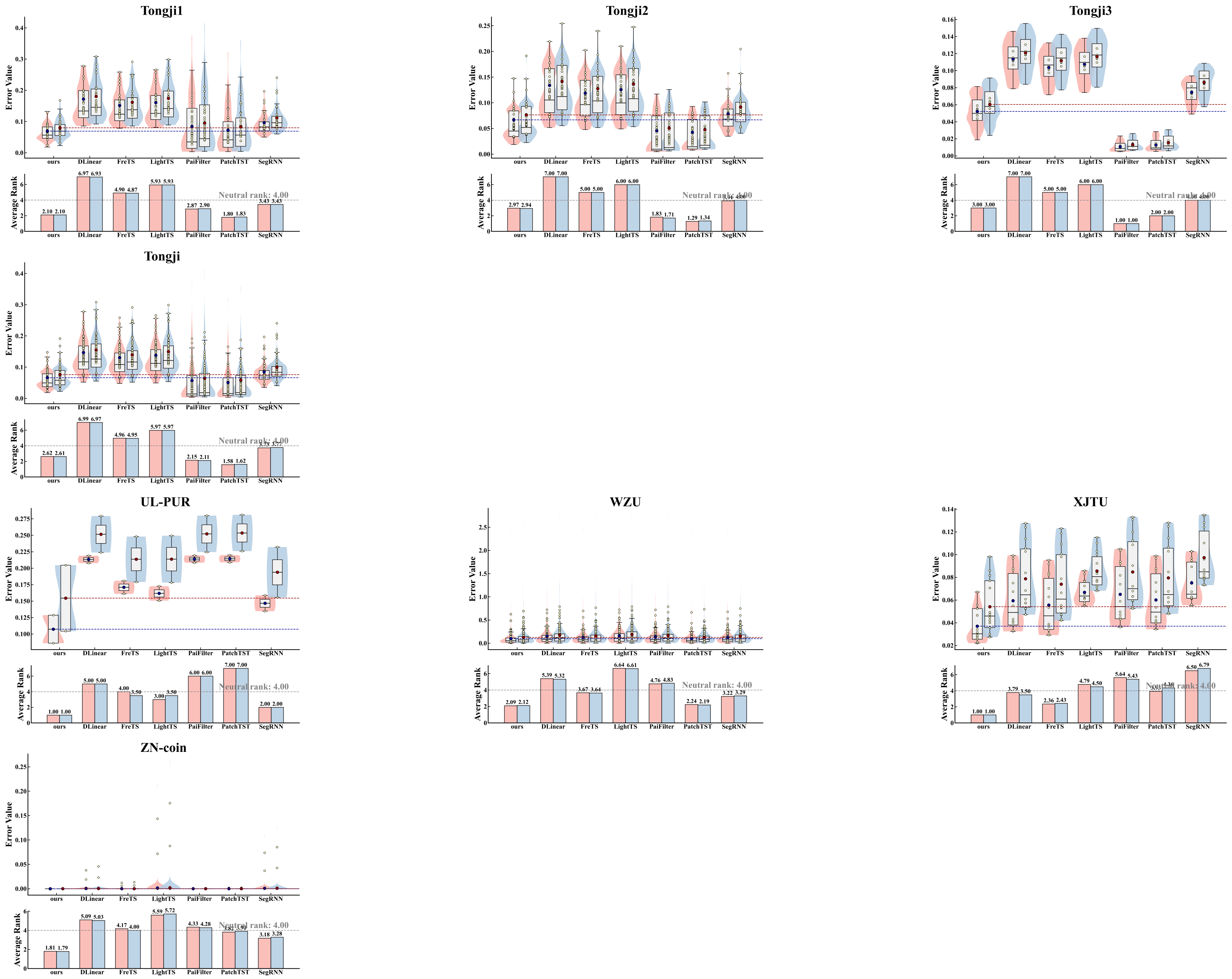
Tongji1
Tongji2
Tongji3
Tongji
UL-PUR
WZU
XJTU
ZN-coin
Error Value
Average Rank
Neutral rank: 4.00
ours
DLinear
FreTS
LightTS
PaiFilter
PatchTST
SegRNN

384

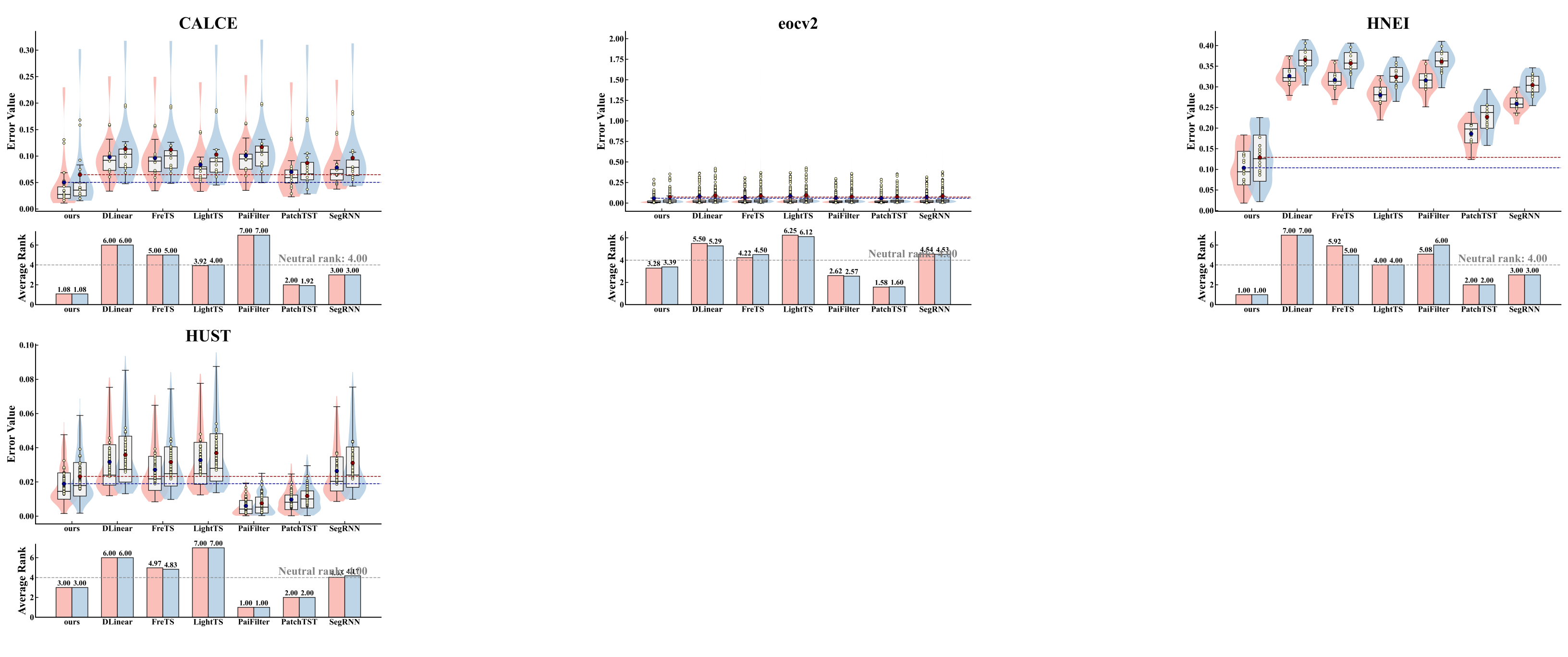
CALCE
eocv2
HNEI
HUST
Error Value
Average Rank
Neutral rank: 4.00
ours
DLinear
FreTS
LightTS
PaiFilter
PatchTST
SegRNN

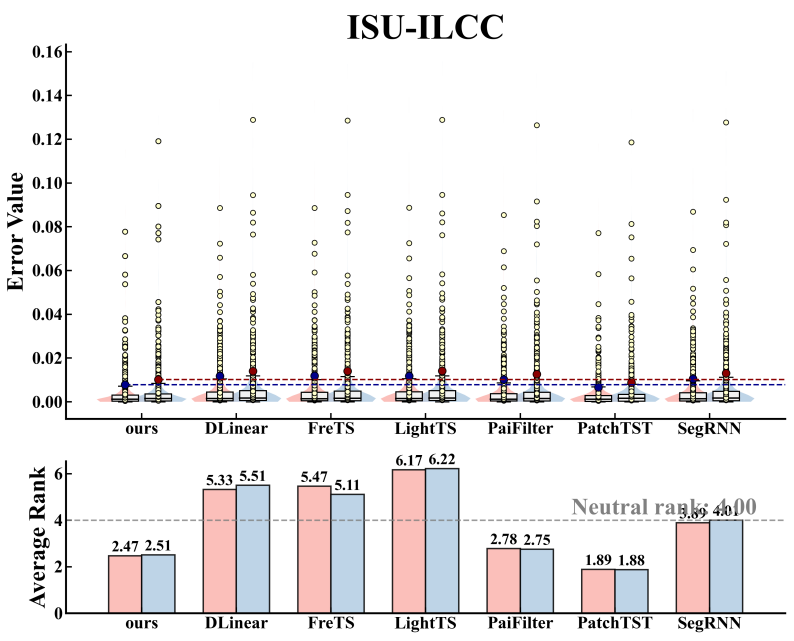
ISU-ILCC
Error Value
Average Rank
Neutral rank: 4.00
ours
DLinear
FreTS
LightTS
PaiFilter
PatchTST
SegRNN

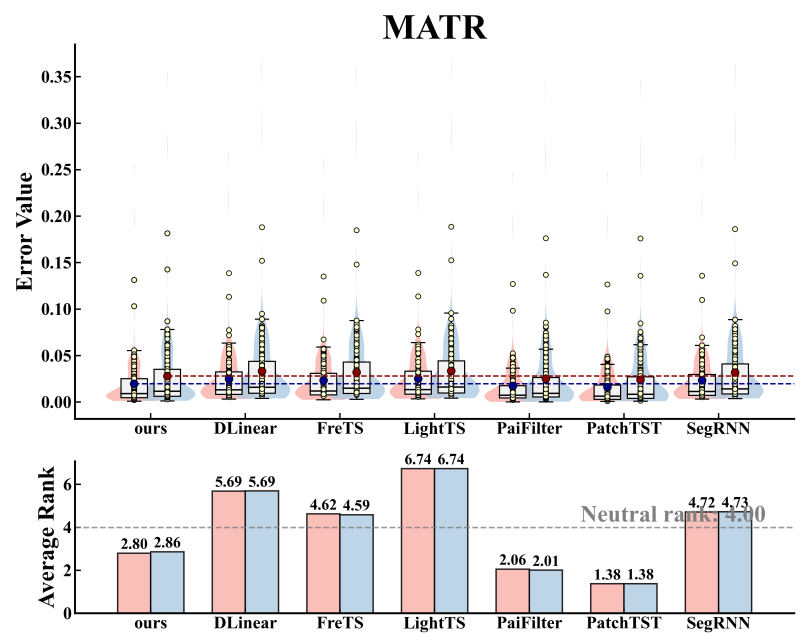
MATR
Error Value
Average Rank
Neutral rank: 4.00
ours
DLinear
FreTS
LightTS
PaiFilter
PatchTST
SegRNN

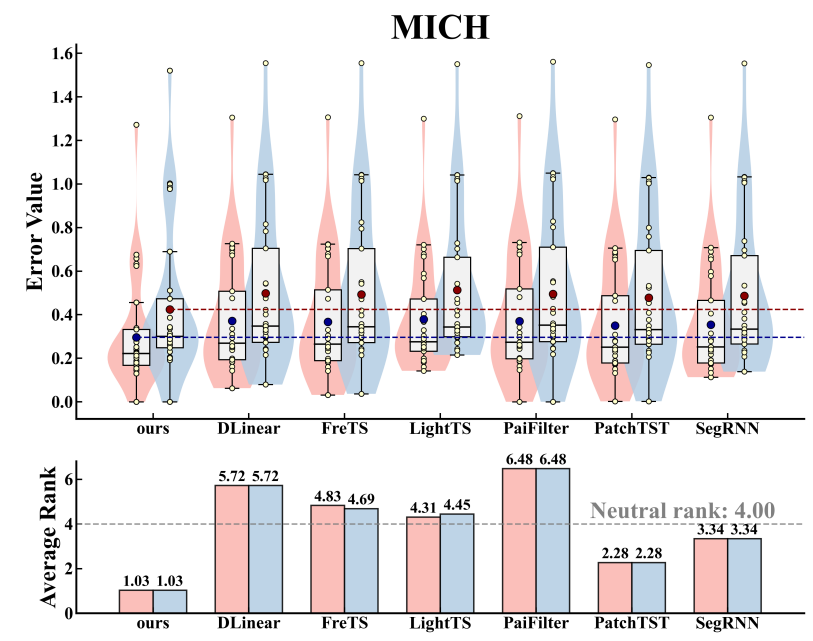
MICH
Error Value
Average Rank
Neutral rank: 4.00
ours
DLinear
FreTS
LightTS
PaiFilter
PatchTST
SegRNN

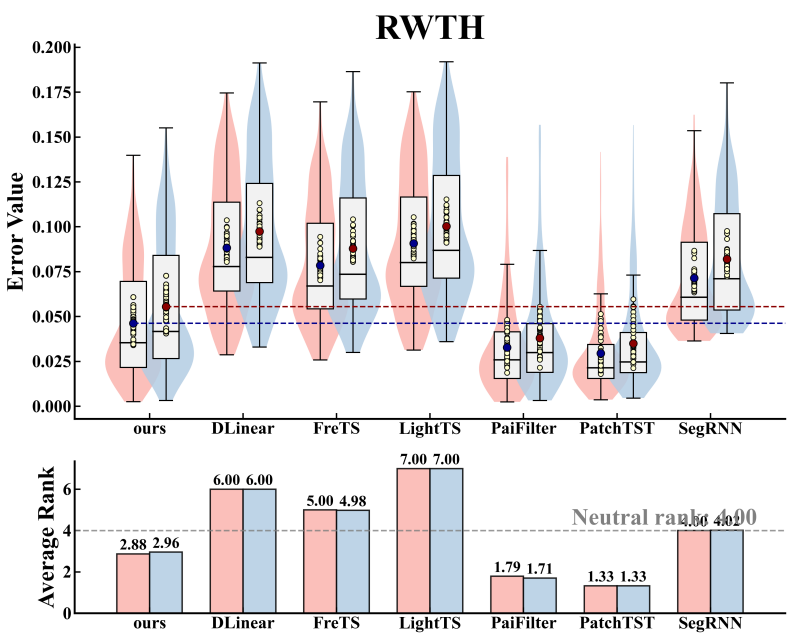
RWTH
Error Value
Average Rank
Neutral rank: 4.00
ours
DLinear
FreTS
LightTS
PaiFilter
PatchTST
SegRNN

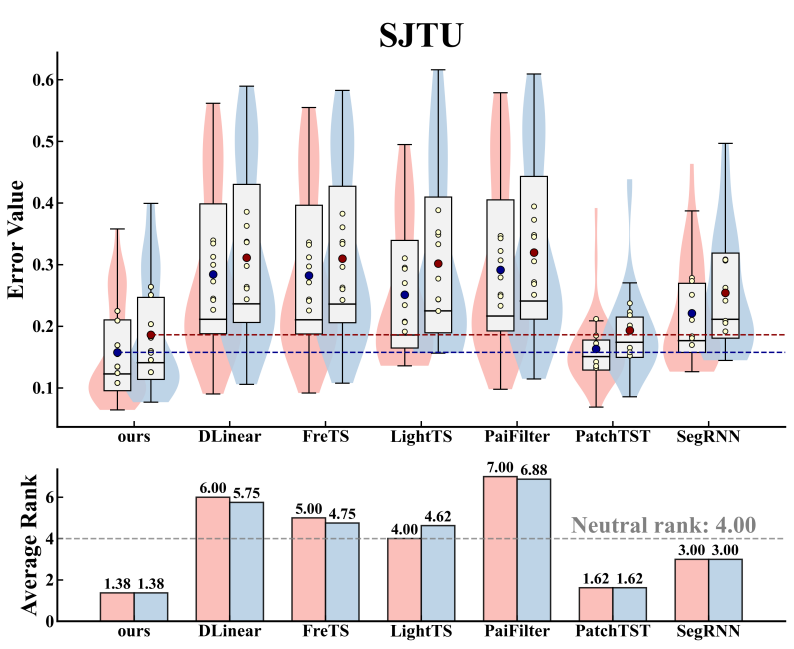
SJTU
Error Value
Average Rank
Neutral rank: 4.00
ours
DLinear
FreTS
LightTS
PaiFilter
PatchTST
SegRNN

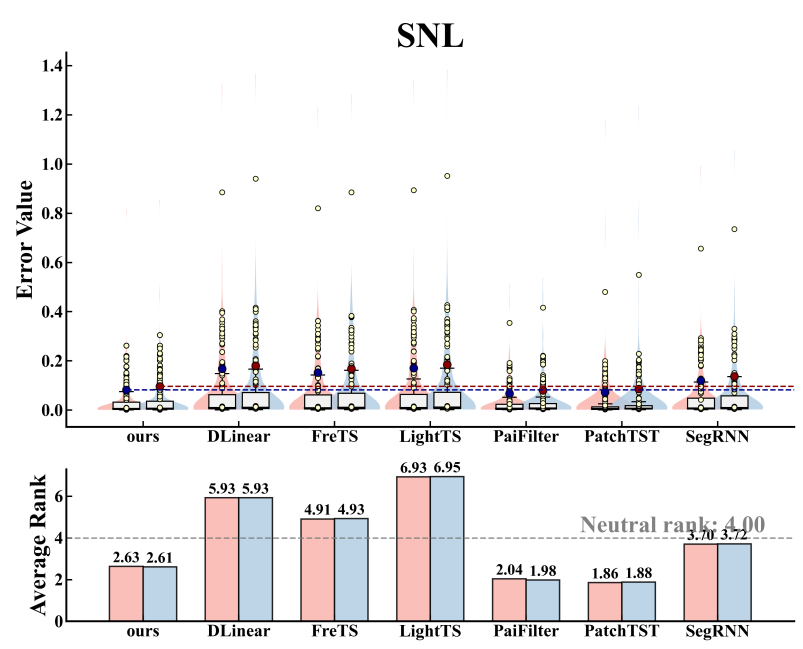
SNL
Error Value
Average Rank
Neutral rank: 4.00
ours
DLinear
FreTS
LightTS
PaiFilter
PatchTST
SegRNN

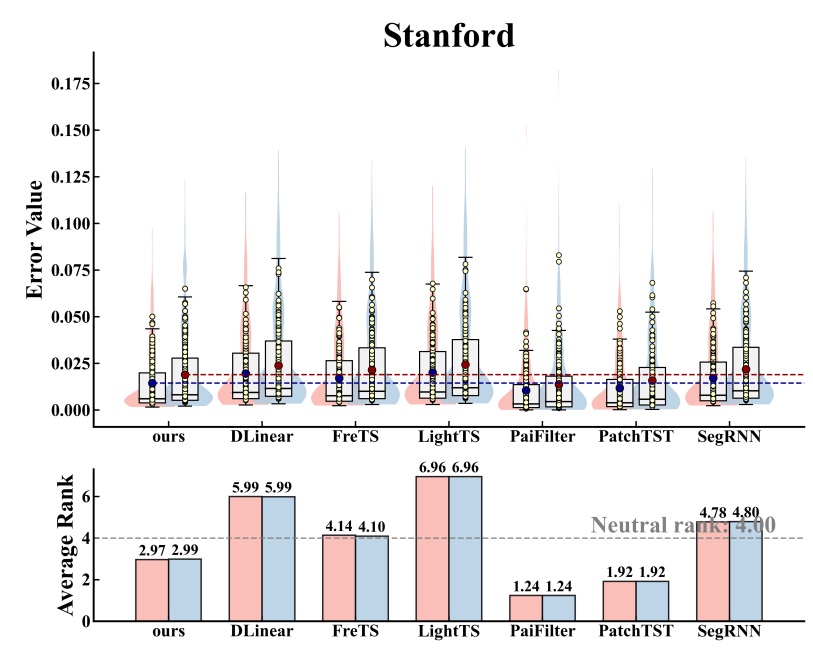
Stanford
Error Value
Average Rank
Neutral rank: 4.00
ours
DLinear
FreTS
LightTS
PaiFilter
PatchTST
SegRNN

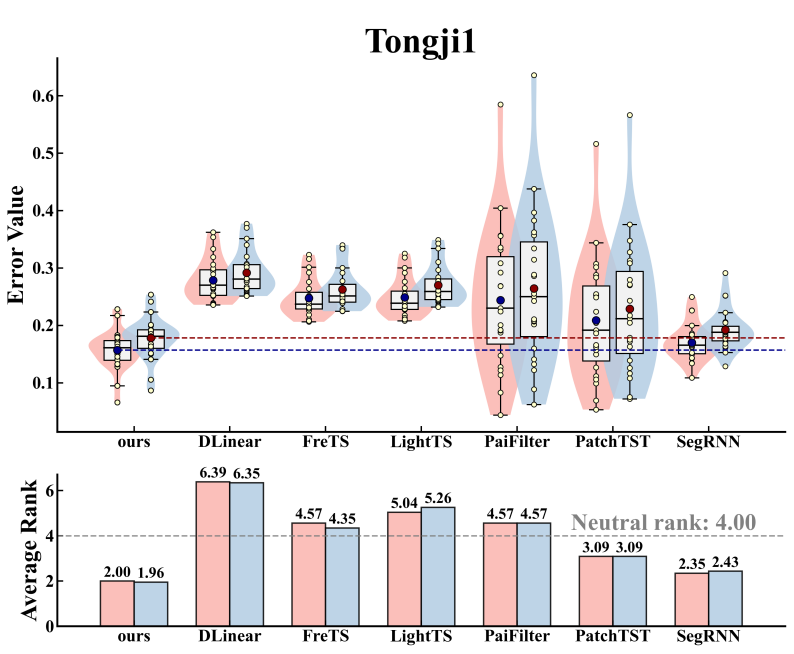
Tongji1
Error Value
Average Rank
Neutral rank: 4.00
ours
DLinear
FreTS
LightTS
PaiFilter
PatchTST
SegRNN

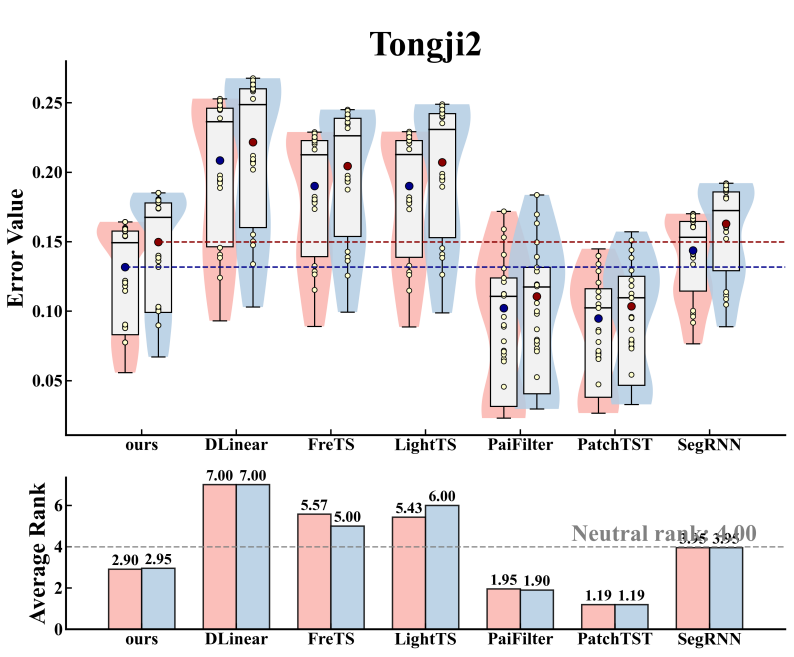
Tongji2
Error Value
Average Rank
Neutral rank: 4.00
ours
DLinear
FreTS
LightTS
PaiFilter
PatchTST
SegRNN

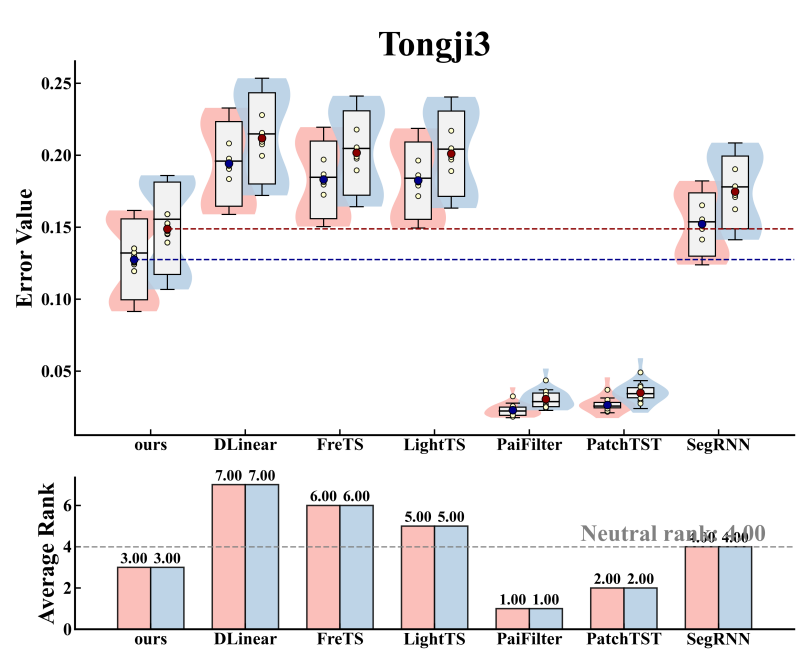
Tongji3
Error Value
Average Rank
Neutral rank: 4.00
ours
DLinear
FreTS
LightTS
PaiFilter
PatchTST
SegRNN

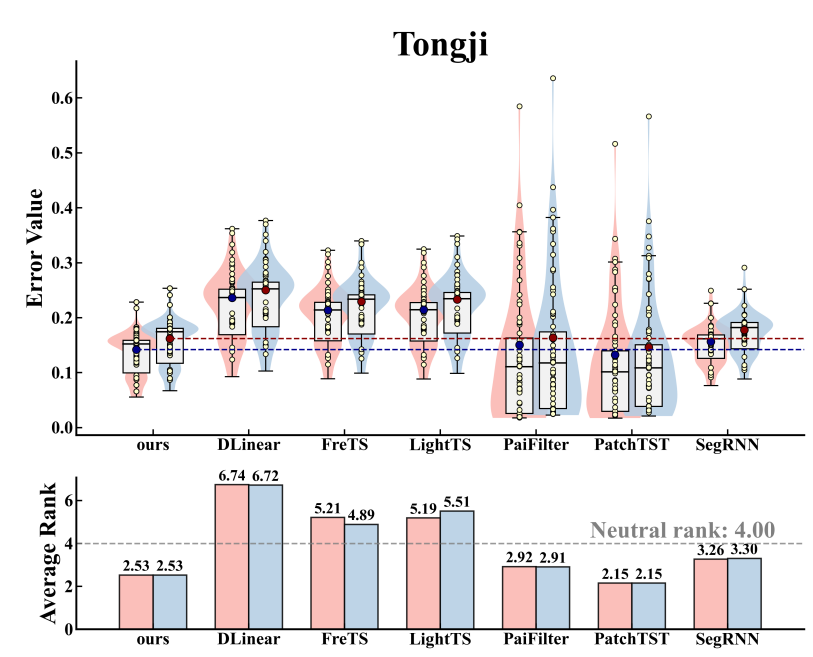
Tongji
Error Value
Average Rank
Neutral rank: 4.00
ours
DLinear
FreTS
LightTS
PaiFilter
PatchTST
SegRNN

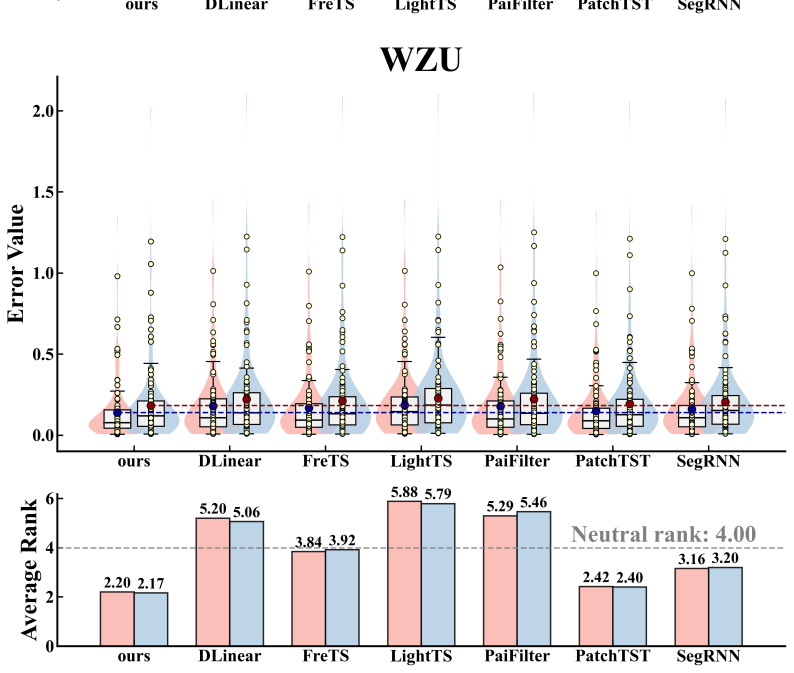
WZU
Error Value
Average Rank
Neutral rank: 4.00
ours
DLinear
FreTS
LightTS
PaiFilter
PatchTST
SegRNN

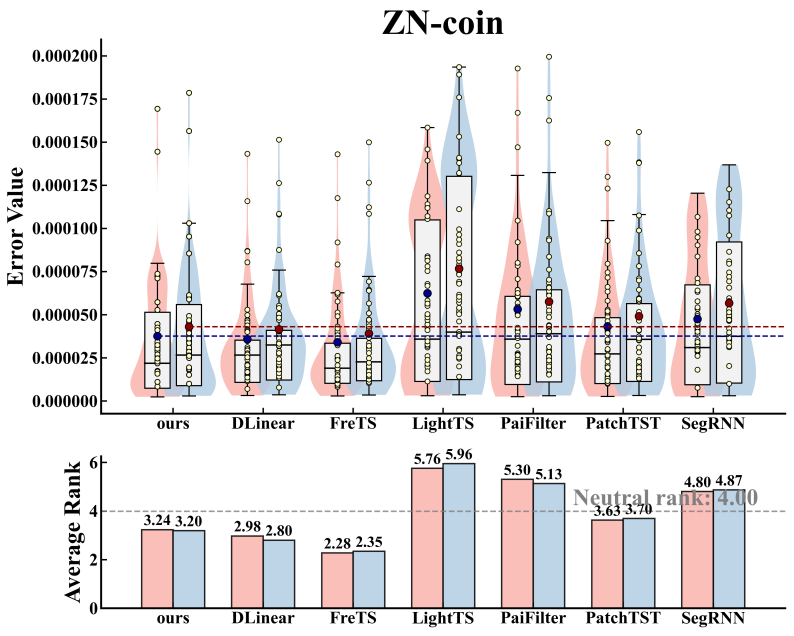
ZN-coin
Error Value
0.000200
0.000175
0.000150
0.000125
0.000100
0.000075
0.000050
0.000025
0.000000
ours
DLinear
FreTS
LightTS
PaiFilter
PatchTST
SegRNN
Average Rank
Neutral rank: 4.00
3.24
3.20
2.98
2.80
2.28
2.35
5.76
5.96
5.30
5.13
3.63
3.70
4.80
4.87
0
2
4
6
