# Supplementary material for: Universal Battery Capacity Degradation Forecasting Driven by Foundation Models Across Diverse Chemistries and Conditions
Source: Adv Sci (Weinh). 2026 Aug 24:e77261. Online ahead of print. doi: 10.1002/advs.77261 (PMC13502271; doi:10.1002/advs.77261)
Supplement: Supplementary file 1 [file ADVS-9999-e77261-s002.pdf]

Supplementary Information for "Universal Battery Capacity Degradation Forecasting Driven by Foundation Models Across Diverse Chemistries and Conditions"

Supplementary Note 1: Overview of the unified battery capacity degradation corpus

Figure S1 provides an overview of the cycle–capacity trajectories for all 20 battery aging datasets used in this study. Each curve corresponds to the measured discharge capacity of a single cell as a function of cycle index, after basic preprocessing and normalization. Together, these datasets constitute the core time-series resource on which our capacity forecasting framework is built.

The unified corpus aggregates laboratory and field measurements from a wide range of sources, including publicly available aging datasets collected and curated in the open-source *BatteryLife* repository (GitHub: `https://github.com/Ruifeng-Tan/BatteryLife`), as well as additional datasets from academic and industrial partners. To the best of our knowledge, this corpus represents the largest and most diverse collection of battery capacity degradation time-series data currently available, covering multiple chemistries (e.g., LFP, LCO, NMC/NCA, Na-ion, Zn-ion, hybrid systems), capacities, formats (cylindrical, pouch, prismatic, coin cells), and operating conditions (temperature ranges, standard CC–CV cycling, fast charging, partial cycling, and real-world dynamic profiles).

From a modeling perspective, this corpus plays a role analogous to the text corpora used to pretrain large language models: instead of sentences and documents, we work with cycle–capacity trajectories spanning 1,000+ cells and millions of cycles. The diversity of degradation behaviors visible in Figure S1—from nearly linear fades to highly nonlinear, accelerated end-of-life drops—highlights both the challenge and the opportunity: a single time-series foundation model must learn common degradation regularities while remaining flexible enough to generalize across previously unknown chemistries and protocols.

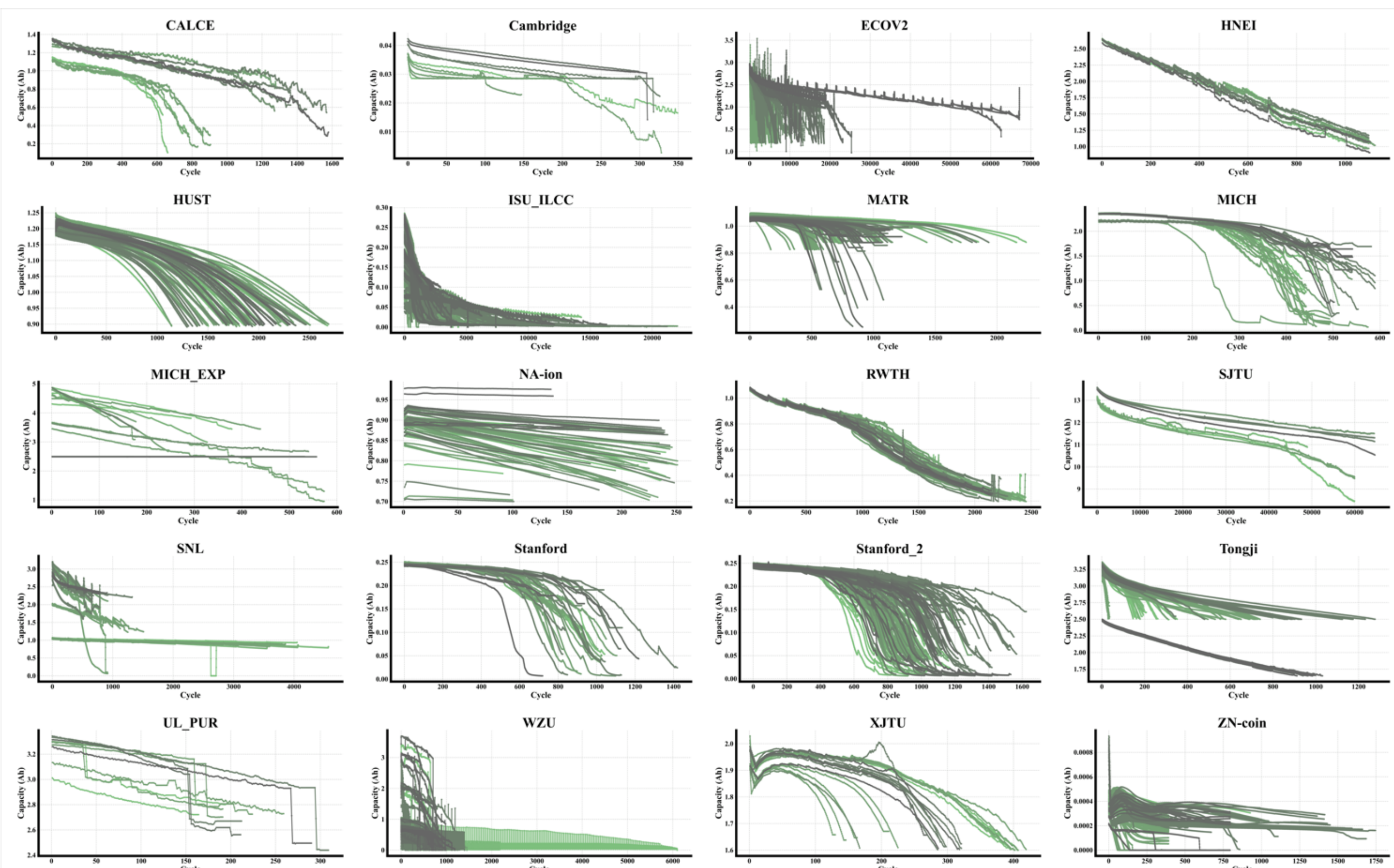

FIG. S1: **Cycle–capacity trajectories for the 20 battery aging datasets used in this study.** Each curve represents the discharge capacity of a single cell as a function of cycle index, aggregated across all datasets in the unified degradation corpus. The figure illustrates the diversity of degradation behaviors across chemistries, formats, capacities, and operating conditions, and serves as the time-series analogue of a large-scale pretraining corpus used for language models.

Supplementary Note 2: Data collection and Battery test system platform

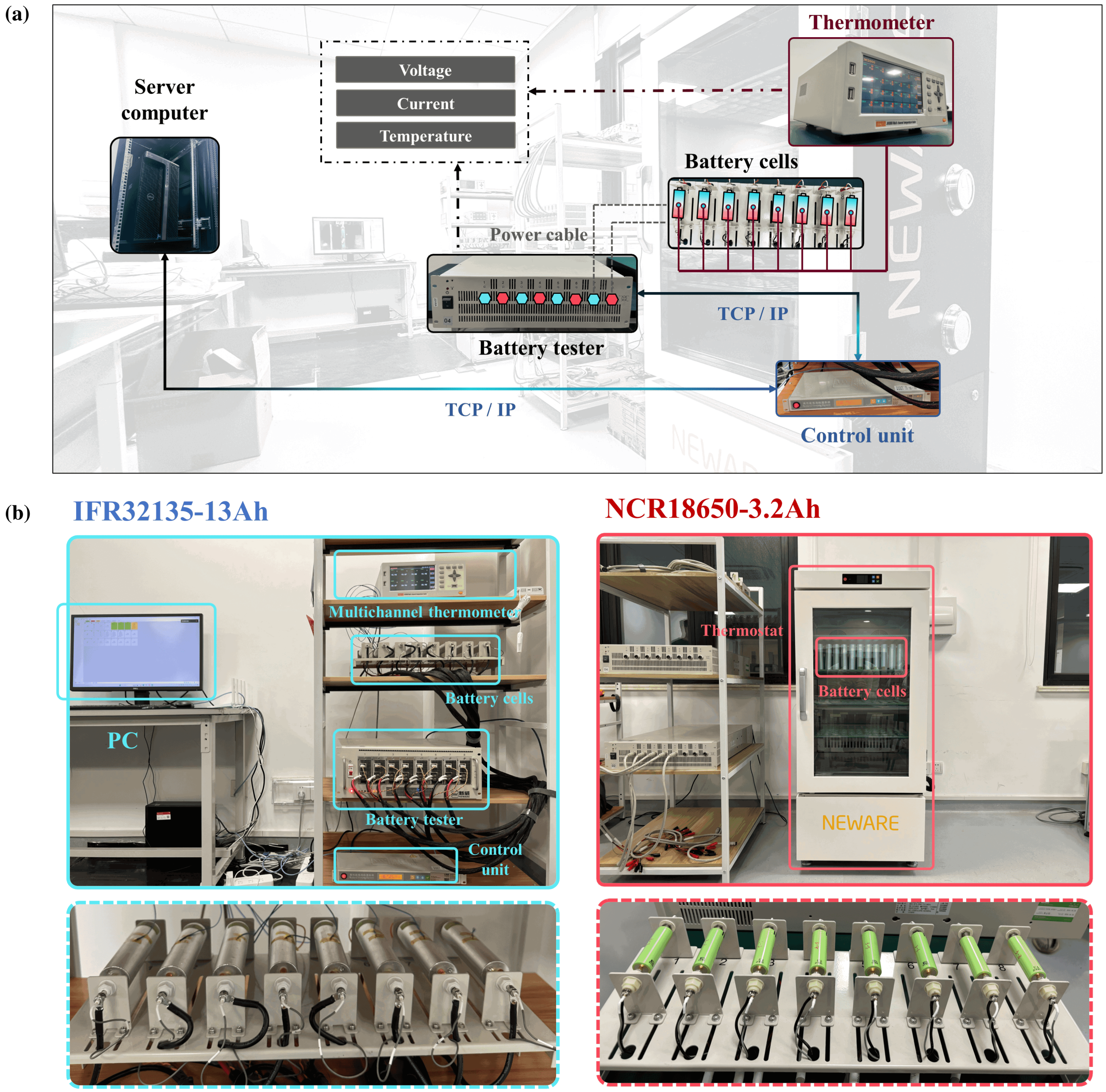

FIG. S2: **Battery test system platform and data acquisition.** **(a)** Schematic of the laboratory testing workflow used to collect degradation data from IFR32135–13 Ah energy-storage cells and NCR18650–3.2 Ah commercial cells, including cycling protocols, periodic capacity checks, and data logging. The 13 Ah IFR32135 degradation data supplement the unified corpus with large-capacity storage batteries that are underrepresented in existing public datasets. **(b)** Photographs of the battery test system, showing the tested cells mounted in fixed clamps, the temperature chamber, the multi-channel battery tester, measurement instruments, and the control computer.

Figure S2(a) illustrates the experimental workflow used in our laboratory to collect degradation data for both high-capacity energy-storage cells and commercial Li-ion cells. In particular, we test IFR32135–13 Ah prismatic cells that are of the same type as those deployed in real energy storage power stations, together with NCR18650–3.2 Ah cylindrical cells that represent typical commercial batteries. The resulting 13 Ah degradation trajectories fill an important gap in the unified corpus, which is otherwise dominated by small- to medium-capacity cells, and thus extend our dataset coverage toward realistic large-scale energy storage applications.

Figure S2(b) shows photographs of the battery test system platform, including the tested cells and the associated hardware. The platform consists of a programmable battery cycler, a temperature chamber with precise thermal control, fixed battery clamps and wiring fixtures, a thermometer, and a control computer/server. During cycling, the system continuously records voltage, current, and temperature for each channel, while routine capacity checks provide the cumulative maximum discharge capacity used as the primary health indicator in this study.

Previous works have also considered internal resistance growth and power fade as alternative or complementary markers of battery aging. Although simultaneous monitoring of multiple indicators can in principle improve state-of-health assessment, it also increases instrumentation complexity and operational cost. Moreover, many aging indicators are strongly correlated in practice. In this work, we therefore focus on capacity degradation as a unified, physically interpretable and widely available proxy for the aging process, and use the collected IFR32135 and NCR18650 datasets to enrich the degradation corpus for training and evaluating our capacity forecasting framework.

Supplementary Note 3: Cycling profile

TABLE S1: Summary of the battery cycling profiles and associated testing conditions

Datasets	Cell number	Material	Temperature	Cycling profile
Data 1	IFR32135-13Ah	LFP	Room Temp	CCCV-CC[a]
Data 2	IFR32135-13Ah	LFP	Room Temp	Intermittent profile 1
Data 3	NCR18650-3.2Ah	NCR	Temp Chamber	Intermittent profile 2

Cycling profile	Detailed descriptions
CCCV-CC	Charge at 1C(13Ah)-CC to 3.65V, then go to CV mode<br>and continue charging until current becomes 0.65A.<br>After 30 minutes relaxation, discharge at 1C(13Ah)-CC to 2V.<br>After 30 minutes of relaxation, proceed to the next cycle.
Intermittent profile 1	First stage: Charge at 1C(13Ah)-CC to 3.54V.<br>Discharge at 1C(13Ah)-CC to 2.97V.<br>Next stage: Charge at 1C(13Ah)-CC to 3.54V.<br>Discharge at 1.5C(19.5Ah)-CC to 2.97V.
Intermittent profile 2	CC charge mode: Charge at 1C(3.25Ah)-CC to 3.9V.<br>Multi CC discharge mode: Discharge at 0.5C/1C/1.5C to 2.8V.
Regular capacity tests 1	Frequency: every 60 cycles for Data 2<br>Cycle under CCCV-CC mode (2V-3.65V)<br>Then cycle under Intermittent profile 1.
Regular capacity tests 2	Frequency: every 5 cycles for Data 3<br>Cycle under CCCV-CC mode (2.5V-4.2V)<br>Then cycle under Intermittent profile 2.

[a]CC means charged at constant current. CV means charged at constant voltage.

Supplementary Note 4: Intrinsic capacity degradation as an endogenous variable

Inventory of degradation features (DF)

To characterize the intrinsic degradation of capacity as an endogenous variable, we first focus on cells whose chemistries are explicitly known, namely NMC, LFP, and LCO. For each cell, we aggregate its cycle–capacity trajectory across extremely diverse operating conditions (datasets, C-rates, temperatures, and cycling protocols), and extract a set of purely *shape-/trend-based* and dimensionless degradation features. The goal is to demonstrate, through large-scale statistics, that different chemistries exhibit common latent patterns in their capacity fading behavior, despite substantial variability in absolute values and test conditions.

Base definitions. Let x_t denote the cycle index and y_t the measured discharge capacity at cycle t, for $t = 1, \ldots, T$. We normalize the capacity with respect to the initial healthy state (e.g., the first valid cycle) as

$$\tilde{y}_t = \frac{y_t}{y_1}, \quad t = 1, \ldots, T. \tag{S1}$$

First- and second-order differences are defined as

$$\Delta\tilde{y}_t = \tilde{y}_{t+1} - \tilde{y}_t, \quad t = 1, \ldots, T-1, \tag{S2}$$

$$\Delta^2\tilde{y}_t = \Delta\tilde{y}_{t+1} - \Delta\tilde{y}_t, \quad t = 1, \ldots, T-2. \tag{S3}$$

We also fit a global linear model of normalized capacity versus cycle index,

$$\tilde{y}_t \approx \beta_0 + \beta_1 x_t, \tag{S4}$$

and define the fitted values and residuals as

$$\hat{y}_t = \beta_0 + \beta_1 x_t, \quad r_t = \tilde{y}_t - \hat{y}_t. \tag{S5}$$

Let $n = T$ be the number of observations. The root mean squared error (RMSE) of the linear fit is

$$\mathrm{RMSE}_{\mathrm{lin}} = \sqrt{\frac{1}{n}\sum_{t=1}^{n} r_t^2}, \tag{S6}$$

and the coefficient of determination is

$$R^2_{\mathrm{lin}} = 1 - \frac{\sum_{t=1}^{n} r_t^2}{\sum_{t=1}^{n}(\tilde{y}_t - \bar{y})^2}, \tag{S7}$$

where $\bar{y} = \frac{1}{n}\sum_{t=1}^{n}\tilde{y}_t$.

We denote the mean, median, standard deviation, and median absolute deviation (MAD) of a sequence $\{z_t\}$ by $\mathrm{mean}(z)$, $\mathrm{median}(z)$, $\mathrm{std}(z)$, and

$$\mathrm{MAD}(z) = \mathrm{median}\big(|z_t - \mathrm{median}(z)|\big), \tag{S8}$$

respectively. The α-quantile of $\{z_t\}$ is written as $Q_\alpha(z)$.

The life at a relative capacity threshold $q \in \{0.9, 0.8\}$ is defined as

$$\mathrm{life}_q = \min\{x_t \mid \tilde{y}_t \le q\}, \tag{S9}$$

if such t exists; otherwise, life_q is treated as missing.

We further define the normalized area under the degradation curve as

$$\mathrm{AUC}_{\mathrm{norm}} = \frac{\int_{x_1}^{x_T}\tilde{y}(x)\,dx}{x_T - x_1} \approx \frac{1}{x_T - x_1}\sum_{t=1}^{T-1}\tilde{y}_t(x_{t+1} - x_t). \tag{S10}$$

To capture late-stage acceleration, we perform a single-break segmented linear regression on $(x_t, \tilde{y}_t)$, yielding a candidate knee index t_k and corresponding cycle $\mathrm{knee\_cycle} = x_{t_k}$, as well as left and right slopes β_1^L and β_1^R. We denote the improvement in Bayesian information criterion (BIC) of the segmented model over the global linear model by knee_bic_gain.

Finally, let $\mathrm{sign}(z_t) \in \{-1, 0, 1\}$ be the sign of z_t, and let $\mathbf{1}(\cdot)$ be the indicator function that equals 1 if the condition holds and 0 otherwise.

Definitions of the 35 degradation features. Using the notation above, we define 35 scalar degradation features denoted DFk, $k = 1, \ldots, 35$. Each DFk corresponds to the implementation names listed in parentheses.

Curvature and concavity:

$$\mathrm{DF1}\ (\texttt{frac\_concave\_down}) = \frac{1}{T-2}\sum_{t=1}^{T-2} \mathbf{1}\big(\Delta^2 \tilde{y}_t < 0\big). \tag{S11}$$

$$\mathrm{DF2}\ (\texttt{mean\_second\_diff}) = \mathrm{mean}\big(\Delta^2 \tilde{y}_t\big). \tag{S12}$$

Global level and life fractions:

$$\mathrm{DF3}\ (\texttt{auc\_norm}) = \mathrm{AUC}_{\mathrm{norm}}. \tag{S13}$$

$$\mathrm{DF4}\ (\texttt{frac\_above\_90}) = \frac{1}{T}\sum_{t=1}^{T} \mathbf{1}\big(\tilde{y}_t \geq 0.9\big). \tag{S14}$$

$$\mathrm{DF8}\ (\texttt{frac\_above\_80}) = \frac{1}{T}\sum_{t=1}^{T} \mathbf{1}\big(\tilde{y}_t \geq 0.8\big). \tag{S15}$$

$$\mathrm{DF11}\ (\texttt{retention\_end}) = \tilde{y}_T. \tag{S16}$$

$$\mathrm{DF14}\ (\texttt{life\_90}) = \mathrm{life}_{0.9}. \tag{S17}$$

$$\mathrm{DF18}\ (\texttt{life\_80}) = \mathrm{life}_{0.8}. \tag{S18}$$

Linear trend and robustness:

$$\mathrm{DF10}\ (\texttt{slope\_linear}) = \beta_1. \tag{S19}$$

$$\mathrm{DF28}\ (\texttt{rmse\_linear}) = \mathrm{RMSE}_{\mathrm{lin}}. \tag{S20}$$

$$\mathrm{DF29}\ (\texttt{r2\_linear}) = R^2_{\mathrm{lin}}. \tag{S21}$$

Let $\hat{\beta}_1^{\mathrm{TS}}$ denote the Theil–Sen slope estimator,

$$\hat{\beta}_1^{\mathrm{TS}} = \mathrm{median}_{1 \leq i < j \leq T} \left(\frac{\tilde{y}_j - \tilde{y}_i}{x_j - x_i}\right), \tag{S22}$$

then

$$\mathrm{DF12}\ (\texttt{theil\_sen\_slope}) = \hat{\beta}_1^{\mathrm{TS}}. \tag{S23}$$

Spearman's rank correlation between x_t and $\tilde{y}_t$ is

$$\rho_{\mathrm{Spear}} = \mathrm{corr}_{\mathrm{rank}}(x_t, \tilde{y}_t), \tag{S24}$$

and

$$\mathrm{DF20}\ (\texttt{spearman\_rho}) = \rho_{\mathrm{Spear}}. \tag{S25}$$

First-order difference statistics (degradation rate):

$$\mathrm{DF5}\ (\texttt{mean\_delta}) = \mathrm{mean}\big(\Delta \tilde{y}_t\big). \tag{S26}$$

$$\text{DF31 } (\texttt{median\_delta}) = \operatorname{median}\big(\Delta\tilde{y}_t\big). \tag{S27}$$

$$\text{DF13 } (\texttt{std\_delta}) = \operatorname{std}\big(\Delta\tilde{y}_t\big). \tag{S28}$$

$$\text{DF24 } (\texttt{mad\_delta}) = \operatorname{MAD}\big(\Delta\tilde{y}_t\big). \tag{S29}$$

$$\text{DF33 } (\texttt{frac\_positive\_delta}) = \frac{1}{T-1}\sum_{t=1}^{T-1} \mathbf{1}\big(\Delta\tilde{y}_t > 0\big). \tag{S30}$$

$$\text{DF9 } (\texttt{min\_step\_drop}) = \min_{1\le t\le T-1} \Delta\tilde{y}_t. \tag{S31}$$

$$\text{DF21 } (\texttt{q05\_delta}) = Q_{0.05}\big(\Delta\tilde{y}_t\big). \tag{S32}$$

$$\text{DF30 } (\texttt{q95\_delta}) = Q_{0.95}\big(\Delta\tilde{y}_t\big). \tag{S33}$$

To capture acceleration in the degradation rate, we regress $\Delta\tilde{y}_t$ on x_{t+1} for $t = 1,\ldots,T-1$:

$$\Delta\tilde{y}_t \approx \gamma_0 + \gamma_1 x_{t+1}, \tag{S34}$$

and define

$$\text{DF15 } (\texttt{slope\_of\_delta}) = \gamma_1. \tag{S35}$$

Residual statistics (deviation from linear trend): Let $\sigma_r = \operatorname{std}(r_t)$ and $\mathrm{MAD}_r = \operatorname{MAD}(r_t)$.

$$\text{DF27 } (\texttt{resid\_std}) = \sigma_r. \tag{S36}$$

$$\text{DF16 } (\texttt{resid\_mad}) = \mathrm{MAD}_r. \tag{S37}$$

If $\sigma_r > 0$, we define standardized residuals $z_t = (r_t - \bar{r})/\sigma_r$, with $\bar{r} = \frac{1}{n}\sum_t r_t$, and compute

$$\operatorname{skew}(r) = \frac{1}{n}\sum_{t=1}^{n} z_t^3, \tag{S38}$$

$$\operatorname{kurt}_{\text{excess}}(r) = \frac{1}{n}\sum_{t=1}^{n} z_t^4 - 3. \tag{S39}$$

Then

$$\text{DF22 } (\texttt{resid\_skew}) = \operatorname{skew}(r), \tag{S40}$$

$$\text{DF23 } (\texttt{resid\_kurt\_excess}) = \operatorname{kurt}_{\text{excess}}(r). \tag{S41}$$

Early/late-stage slopes and their contrast: Let $n_e = \max\{5, \lceil 0.1T \rceil\}$ be the length of the early/late segments. We fit two separate linear models on the first and last n_e points:

$$\tilde{y}_t \approx \beta_0^{\text{early}} + \beta_1^{\text{early}} x_t, \quad t = 1,\ldots,n_e, \tag{S42}$$

$$\tilde{y}_t \approx \beta_0^{\text{late}} + \beta_1^{\text{late}} x_t, \quad t = T-n_e+1,\ldots,T. \tag{S43}$$

Then

$$\mathrm{DF}\,7\;(\texttt{early\_slope}) = \beta_1^{\text{early}}, \tag{S44}$$

$$\mathrm{DF}\,17\;(\texttt{late\_slope}) = \beta_1^{\text{late}}. \tag{S45}$$

The change and ratio of slopes are

$$\mathrm{DF}\,26\;(\texttt{slope\_change}) = \beta_1^{\text{late}} - \beta_1^{\text{early}}, \tag{S46}$$

$$\mathrm{DF}\,35\;(\texttt{slope\_ratio\_abs}) = \frac{|\beta_1^{\text{late}}|}{|\beta_1^{\text{early}}| + \varepsilon}, \tag{S47}$$

where ε is a small positive constant (e.g., 10^{-12}) to avoid division by zero.

Knee-point features (shape change): From the single-break segmented regression, we obtain a knee index t_k, knee cycle x_{t_k}, left slope β_1^L, right slope β_1^R, and BIC values for the base and segmented models, $\mathrm{BIC}_{\text{base}}$ and $\mathrm{BIC}_{\text{seg}}$. We define

$$\mathrm{DF}\,19\;(\texttt{knee\_cycle}) = x_{t_k}, \tag{S48}$$

$$\mathrm{DF}\,6\;(\texttt{knee\_bic\_gain}) = \mathrm{BIC}_{\text{base}} - \mathrm{BIC}_{\text{seg}}, \tag{S49}$$

$$\mathrm{DF}\,25\;(\texttt{knee\_slope\_delta}) = \beta_1^R - \beta_1^L, \tag{S50}$$

$$\mathrm{DF}\,34\;(\texttt{knee\_slope\_ratio}) = \frac{|\beta_1^R|}{|\beta_1^L| + \varepsilon}. \tag{S51}$$

Runs test on sign of degradation rate: Let $s_t = \operatorname{sign}(\Delta\tilde{y}_t) \in \{-1,0,1\}$ for $t = 1,\dots,T-1$, and discard zeros. Let $n = n_1 + n_2$ be the number of nonzero signs, where n_1 is the number of positive signs and n_2 the number of negative signs. Let R be the number of runs (maximal contiguous subsequences of equal sign). For the standard runs test, the expected value and variance of R are

$$\mu_R = 1 + \frac{2n_1n_2}{n_1+n_2}, \tag{S52}$$

$$\sigma_R^2 = \frac{2n_1n_2\,(2n_1n_2 - n_1 - n_2)}{(n_1+n_2)^2\,(n_1+n_2-1)}. \tag{S53}$$

The Z-score of the runs test is

$$Z_{\text{runs}} = \frac{R-\mu_R}{\sigma_R}, \tag{S54}$$

and we define

$$\mathrm{DF}\,32\;(\texttt{runs\_test\_z}) = Z_{\text{runs}}. \tag{S55}$$

Collectively, the 35 features DF 1–DF 35 provide a comprehensive, purely relative description of the degradation trajectory shape, independent of absolute capacity, C-rate, or temperature scales. We compute these features for a large number of NMC, LFP, and LCO cells across multiple datasets. As shown in the main text and supplementary figures, despite clear differences in numerical values and degradation rates, the distributions of DF k reveal shared structural patterns across chemistries, which supports the feasibility of learning a unified TSFM-based capacity degradation forecaster across heterogeneous operating conditions.

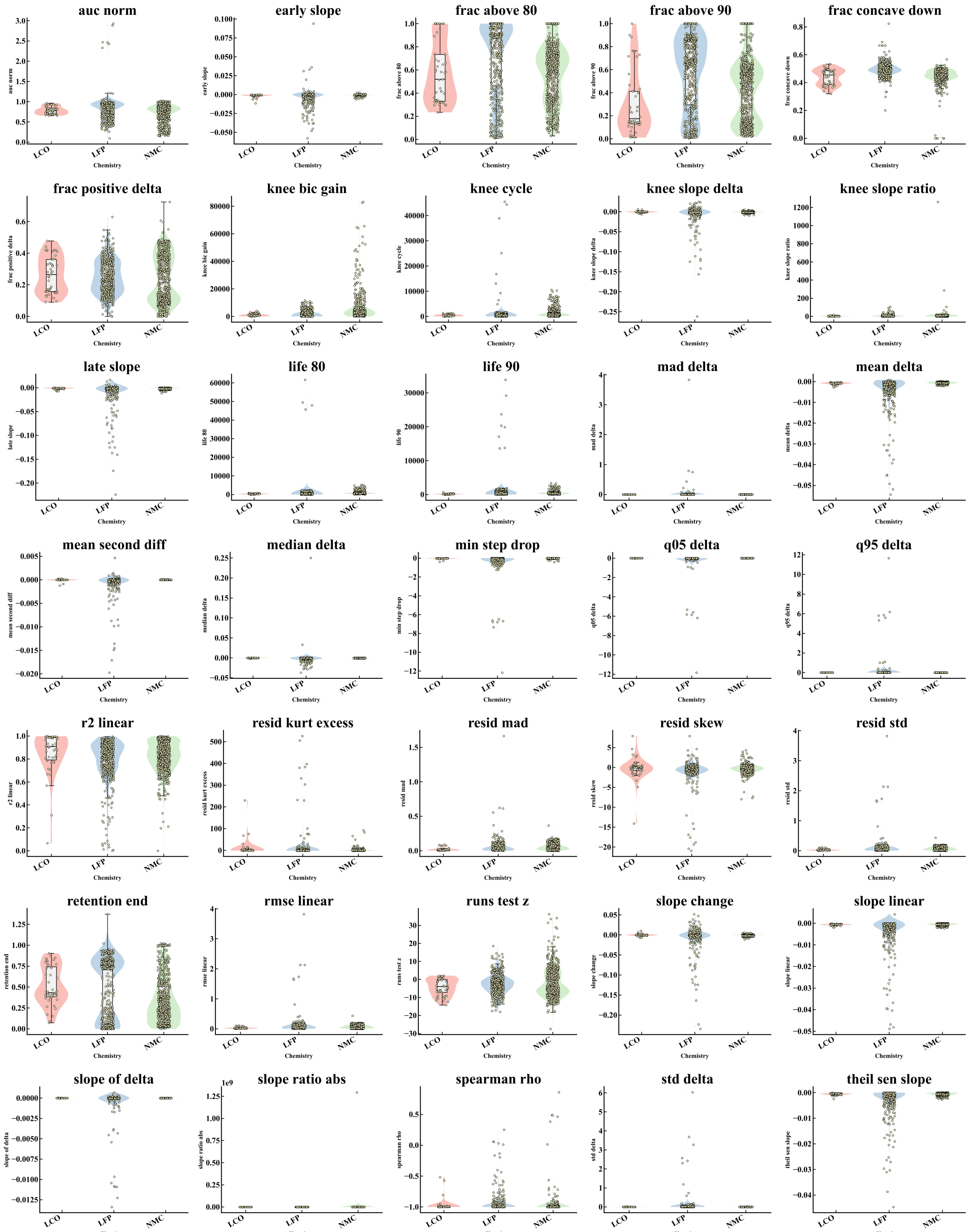

FIG. S3: **Distribution of 35 degradation features across battery chemistries.** Each panel shows a combined violin–box plot for one degradation feature (DF.1–DF.35, as defined in Supplementary Note 4), stratified by chemistry. Boxes highlight median and interquartile ranges, while violins depict the full empirical distribution for each chemistry group. Despite clear chemistry-dependent differences in distributional shape, the centers of many features remain comparable across chemistries, consistent with the "high mutual information, low η^2" pattern quantified in Fig. 2(b). These results indicate that capacity degradation features exhibit strong cross-chemistry commonality with superimposed chemistry-specific variations.

Summary of degradation feature measurements

Figure S3 summarizes the empirical distributions of all 35 degradation features DF.1–DF.35 across different battery chemistries using violin–box plots. Each point in these plots corresponds to a single cell, and each violin shows the distribution of a specific

degradation feature within a given chemistry group. This visualization provides a direct view of both the shared structure and the chemistry-specific variability of the extracted features.

To quantify how strongly each feature depends on chemistry, we further compute two complementary statistics for every degradation feature: the one-way ANOVA effect size η^2 and the mutual information $\mathrm{MI}(\mathrm{DF}_k;\mathrm{chemistry})$. The effect size η^2 is sensitive mainly to differences in group means, while mutual information captures any distributional dependence, including differences in spread, skewness, and multimodality. As shown in the main text (Fig. 2(b)), many features exhibit a "high MI, low η^2" pattern, indicating that different chemistries share similar average degradation levels but differ more strongly in the shape of their feature distributions (e.g., tails or variability). This finding supports our claim that degradation trajectories possess strong cross-chemistry commonalities while still encoding chemistry-specific nuances that a unified TSFM can exploit.

Notation. Let v_i denote the value of a particular degradation feature (e.g., DF.7, DF.20) for cell i and let $c_i \in \{1,\dots,G\}$ denote its chemistry label (e.g., LFP, LCO, NMC). We write $\mathscr{I} = \{1,\dots,N\}$ for the index set of all valid cells.

Algorithm : One-way ANOVA effect size η^2

We compute the ANOVA effect size η^2 for a given degradation feature and chemistry labels as summarized in Algorithm 1.

Algorithm1 Computation of one-way ANOVA effect size η^2

Require: Feature values $\{v_i\}_{i=1}^N$, chemistry labels $\{c_i\}_{i=1}^N$.
Ensure: Effect size $\eta^2 \in [0,1]$.

Define the valid index set $\mathscr{I}_{\text{valid}} = \{i \in \mathscr{I} \mid v_i \text{ is finite and not missing}\}$. If $|\mathscr{I}_{\text{valid}}| < 2$, **return** $\eta^2 = \text{NaN}$.

Compute the overall mean $\bar{v} = \frac{1}{|\mathscr{I}_{\text{valid}}|} \sum_{i \in \mathscr{I}_{\text{valid}}} v_i$.

For each chemistry group $g \in \{1,\dots,G\}$, define $\mathscr{I}_g = \{i \in \mathscr{I}_{\text{valid}} \mid c_i = g\}$ and compute $\bar{v}_g = \frac{1}{|\mathscr{I}_g|} \sum_{i \in \mathscr{I}_g} v_i$ and $n_g = |\mathscr{I}_g|$.

Compute the total sum of squares $SS_{\text{tot}} = \sum_{i \in \mathscr{I}_{\text{valid}}} (v_i - \bar{v})^2$ and the between-group sum of squares $SS_{\text{between}} = \sum_{g=1}^{G} n_g\,(\bar{v}_g - \bar{v})^2$.

if $SS_{\text{tot}} > 0$ **then**
  Set $\eta^2 = SS_{\text{between}}/SS_{\text{tot}}$.
else
  Set $\eta^2 = \text{NaN}$.
end if
return η^2.

A small η^2 indicates that the feature means are similar across chemistries, even if the full distributions differ. In our analysis, many degradation features exhibit low η^2 despite having non-negligible mutual information with chemistry, suggesting that chemistry affects higher-order aspects of the feature distributions more than their mean levels.

Algorithm : Mutual information between feature and chemistry

We estimate the mutual information between a continuous degradation feature and discrete chemistry labels by quantile-based discretization followed by empirical probability estimation, as shown in Algorithm 2.

A larger MI value indicates a stronger dependence between the feature and chemistry labels, regardless of whether that dependence arises from shifts in mean, changes in variance, or more complex distributional differences. In our measurements, many degradation features display moderate-to-high mutual information but small η^2. This combination confirms that chemistry influences the *shape* of the degradation feature distributions more strongly than their overall mean levels, which is consistent with the visual patterns in Fig. S3.

Algorithm2 Computation of mutual information between feature and chemistry

Require: Feature values $\{v_i\}_{i=1}^N$, chemistry labels $\{c_i\}_{i=1}^N$, number of bins B (here $B = 6$).
Ensure: Mutual information MI in bits.

1: Define $\mathscr{I}_{\text{valid}}$ as in Algorithm 1. If $|\mathscr{I}_{\text{valid}}| = 0$, **return** MI = NaN.
2: Form valid vectors $\mathbf{v} = (v_i)_{i\in\mathscr{I}_{\text{valid}}}$ and $\mathbf{c} = (c_i)_{i\in\mathscr{I}_{\text{valid}}}$, and set $N_{\text{valid}} = |\mathscr{I}_{\text{valid}}|$.
3: Compute empirical quantiles of $\mathbf{v}$: $q_j = \text{quantile}(\mathbf{v}, j/B)$ for $j = 0,\dots,B$, and collect unique edges $\{e_0 < e_1 < \cdots < e_K\}$ from $\{q_j\}$ with $K \le B$. If $K < 1$, **return** MI = 0.
4: Discretize the feature: for each $i \in \mathscr{I}_{\text{valid}}$, assign a bin index $x_i = k$ such that $e_{k-1} \le v_i < e_k$ with $k \in \{1,\dots,K\}$.
5: Estimate empirical marginal and joint probabilities for X (binned feature) and Y (chemistry):

$$p_X(k) = \frac{1}{N_{\text{valid}}} \sum_{i\in\mathscr{I}_{\text{valid}}} \mathbf{1}(x_i = k),$$

$$p_Y(g) = \frac{1}{N_{\text{valid}}} \sum_{i\in\mathscr{I}_{\text{valid}}} \mathbf{1}(c_i = g),$$

$$p_{X,Y}(k,g) = \frac{1}{N_{\text{valid}}} \sum_{i\in\mathscr{I}_{\text{valid}}} \mathbf{1}(x_i = k,\, c_i = g).$$

6: Compute the mutual information

$$\text{MI} = \sum_{k=1}^{K} \sum_{g=1}^{G} p_{X,Y}(k,g) \log_2\left(\frac{p_{X,Y}(k,g)}{p_X(k)\, p_Y(g) + \varepsilon}\right),$$

where terms with $p_{X,Y}(k,g) = 0$ are omitted and ε is a small constant (e.g., 10^{-12}) to avoid numerical division by zero.
7: **return** MI.

Supplementary Note 5: Charge–discharge physical features (PF)

In this study, charge–discharge physical features are extracted *only* from the training portion of the unified degradation corpus; the detailed selection of training datasets and cells is described in the experimental setup. Among the 20 datasets in the corpus, 16 provide cycle-resolved charge–discharge records, but the available channels (e.g., temperature, internal resistance) vary substantially across datasets. To ensure a conservative and broadly applicable design, we restrict our physical descriptors to statistics derived from discharge current and terminal voltage, which are consistently available across all 16 training datasets. These features are used as auxiliary, physics-guided signals to shape the time-series foundation model during training; the final capacity forecasting task itself does *not* require any physical feature inputs at inference time and operates purely on cycle-wise capacity trajectories.

Base notation. For a given cell and a given cycle, let

$$\{I_j\}_{j=1}^N, \quad \{V_j\}_{j=1}^N, \quad \{t_j\}_{j=1}^N$$

denote the sequences of current (in A), voltage (in V), and time stamps (in s) sampled during that cycle. We focus on the *discharge segment* of the cycle, identified by

$$\mathscr{D} = \left\{ j \in \{1,\dots,N\} \,\middle|\, I_j < \theta_{\text{neg}} \right\},$$

where $\theta_{\text{neg}} \le 0$ is a small threshold (in practice $\theta_{\text{neg}} = 0$ A). If $\mathscr{D}$ is empty for a given cycle, that cycle is excluded from the physical-feature analysis.

For notational simplicity, all feature definitions below are understood to be computed over the discharge subset $\{I_j, V_j, t_j : j \in \mathscr{D}\}$. Let $M = |\mathscr{D}|$ denote the number of discharge samples in that cycle. We further define the sample mean, standard deviation, skewness, and kurtosis of a generic sequence $\{z_j\}_{j\in\mathscr{D}}$ as

$$\text{mean}(z) = \frac{1}{M} \sum_{j\in\mathscr{D}} z_j, \quad \text{std}(z) = \sqrt{\frac{1}{M-1} \sum_{j\in\mathscr{D}} \left(z_j - \text{mean}(z)\right)^2}, \tag{S56}$$

$$\text{skew}(z) = \frac{1}{M} \sum_{j\in\mathscr{D}} \left(\frac{z_j - \text{mean}(z)}{\text{std}(z)}\right)^3, \quad \text{kurt}(z) = \frac{1}{M} \sum_{j\in\mathscr{D}} \left(\frac{z_j - \text{mean}(z)}{\text{std}(z)}\right)^4. \tag{S57}$$

The root-mean-square (RMS) of $\{z_j\}$ is defined as

$$\text{RMS}(z) = \sqrt{\frac{1}{M} \sum_{j\in\mathscr{D}} z_j^2}. \tag{S58}$$

To measure the effective discharge duration within a cycle, we use the sum of positive time increments:

$$\Delta t_j = \max\left(0, t_{j+1} - t_j\right), \quad j \in \mathscr{D} \setminus \{\max \mathscr{D}\}, \tag{S59}$$

$$\text{duration}_s = \sum_{j \in \mathscr{D} \setminus \{\max \mathscr{D}\}} \Delta t_j, \tag{S60}$$

where negative or zero time differences (e.g., caused by duplicated or out-of-order time stamps) are clipped to zero.

Definitions of the 10 discharge physical features. For each valid discharge cycle, we compute 10 scalar physical features, denoted PF 1–PF 10, corresponding to the implementation names `current_in_A_avg`, `current_in_A_std`, `current_in_A_skew`, `current_in_A_kurtosis`, `current_in_A_rms`, `voltage_in_V_avg`, `voltage_in_V_std`, `voltage_in_V_skew`, `voltage_in_V_kurtosis`, and `duration_s`:

Current-based features:

$$\text{PF}\,1\ (\texttt{current\_in\_A\_avg}) = \text{mean}(I), \tag{S61}$$

$$\text{PF}\,2\ (\texttt{current\_in\_A\_std}) = \text{std}(I), \tag{S62}$$

$$\text{PF}\,3\ (\texttt{current\_in\_A\_skew}) = \text{skew}(I), \tag{S63}$$

$$\text{PF}\,4\ (\texttt{current\_in\_A\_kurtosis}) = \text{kurt}(I), \tag{S64}$$

$$\text{PF}\,5\ (\texttt{current\_in\_A\_rms}) = \text{RMS}(I). \tag{S65}$$

Voltage-based features:

$$\text{PF}\,6\ (\texttt{voltage\_in\_V\_avg}) = \text{mean}(V), \tag{S66}$$

$$\text{PF}\,7\ (\texttt{voltage\_in\_V\_std}) = \text{std}(V), \tag{S67}$$

$$\text{PF}\,8\ (\texttt{voltage\_in\_V\_skew}) = \text{skew}(V), \tag{S68}$$

$$\text{PF}\,9\ (\texttt{voltage\_in\_V\_kurtosis}) = \text{kurt}(V). \tag{S69}$$

Discharge duration:

$$\text{PF}\,10\ (\texttt{duration\_s}) = \text{duration}_s, \tag{S70}$$

where duration_s is defined in Eq. (S60).

For each training cycle that contains a valid discharge segment, we thus obtain a 10-dimensional physical feature vector

$$\left(\text{PF}\,1, \text{PF}\,2, \ldots, \text{PF}\,10\right),$$

which is subsequently paired with the corresponding cycle-wise discharge capacity and used in the physics-embedded contrastive learning branch. The scatter plots and correlation heatmaps between these ten features and normalized capacity, aggregated over multiple datasets and chemistries, are shown in Fig. S4 and Fig. S5. These results indicate that charge–discharge physical features exhibit non-trivial, chemistry-dependent correlations with capacity degradation, with both shared trends and dataset-specific variations across different battery types.

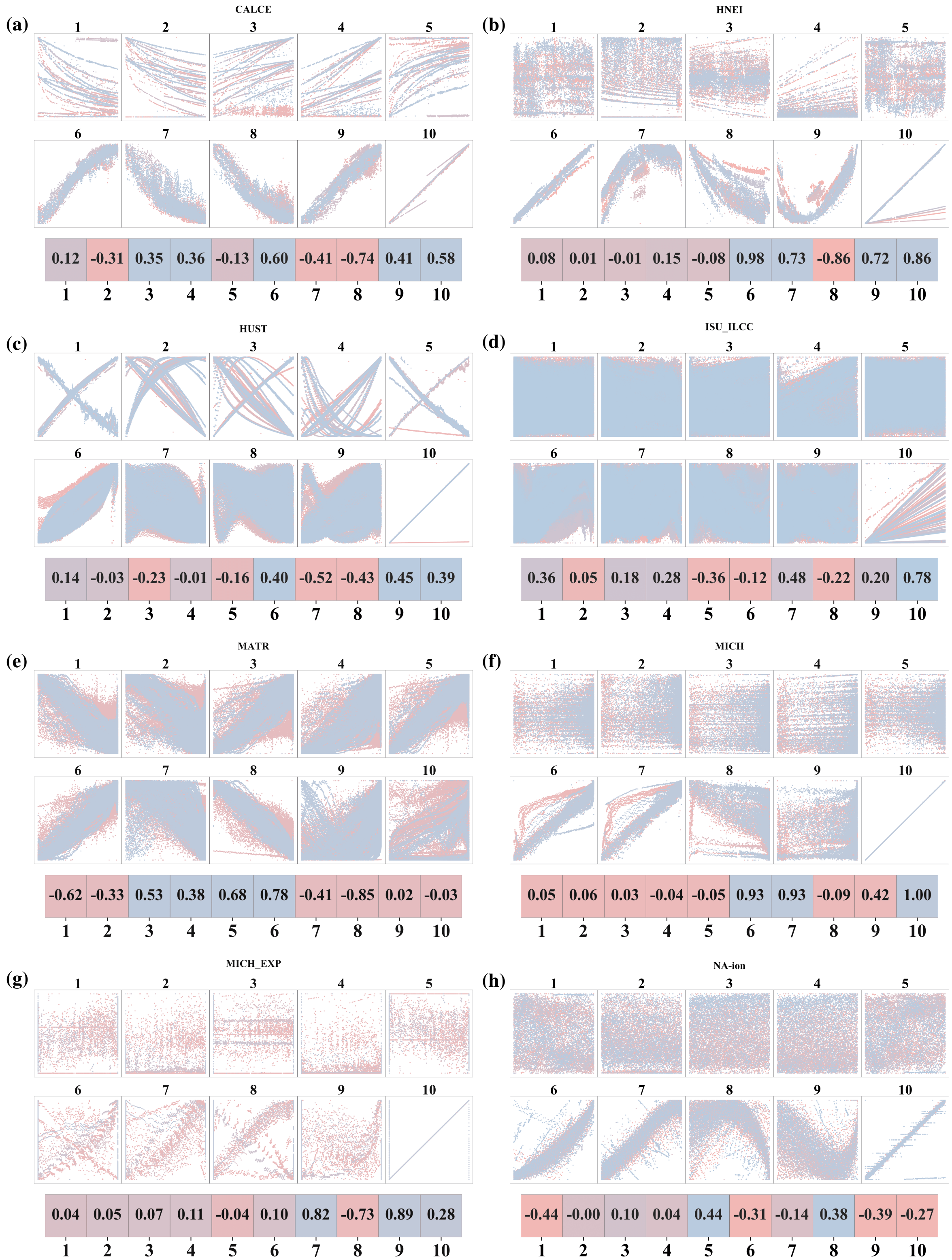

FIG. S4: **Scatter plots of charge/discharge features versus capacity, where the x-axis is the capacity and the y-axis is the physical feature.** **(a)** Result for CALCE. **(b)** Result for HNEI. **(c)** Result for HUST. **(d)** Result for ISU_ILCC. **(e)** Result for MATR. **(f)** Result for MICH. **(g)** Result for MICH_EXP. **(h)** Result for NA-ion.

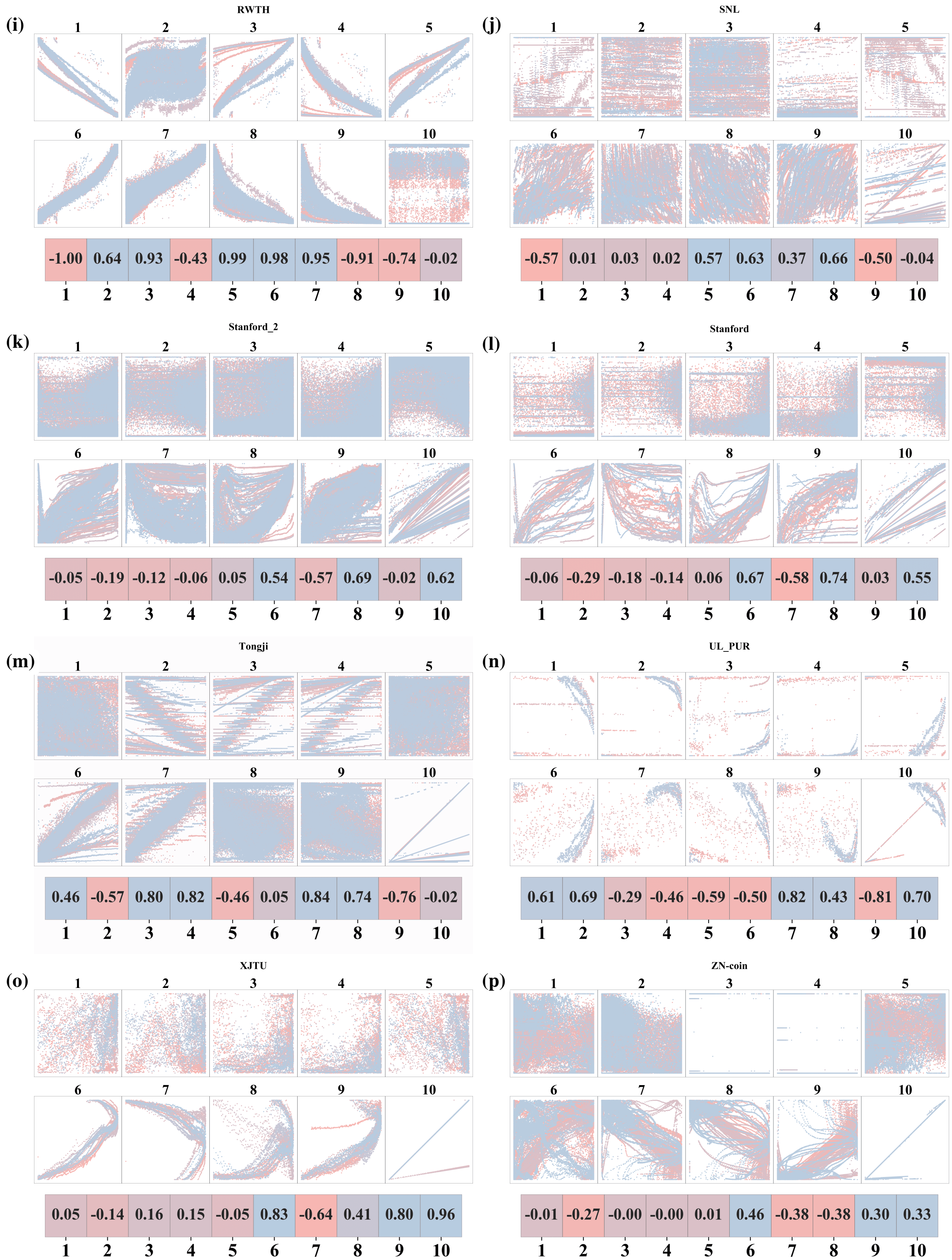

FIG. S5: **Scatter plots of charge/discharge features versus capacity, where the x-axis is the capacity and the y-axis is the physical feature.** **(i)** Result for RWTH. **(j)** Result for SNL. **(k)** Result for Stanford_2. **(l)** Result for Stanford. **(m)** Result for Tongji. **(n)** Result for UL_PUR. **(o)** Result for XJTU. **(p)** Result for ZN-coin.

Supplementary Note 6: Experiment setup

Experiment design

We adopt a window-based procedure for all forecasting experiments. Each capacity degradation trajectory is partitioned into contiguous windows of length 192 time steps. Given that the forecaster is trained with a base horizon of 96, the first 96 steps in each window serve as the input context and the remaining 96 steps constitute the forecast target. This windowing scheme is applied consistently across all datasets and models: for every cell, the entire life-cycle trajectory is segmented into such context–target pairs without using any future information when predicting.

Training/validation protocol on known datasets. As summarized in Table 1, we collect capacity degradation trajectories from a diverse set of 20 public datasets, covering NMC, LCO, LFP, Na-ion, Zn-ion, hybrid chemistries, and multi-chemistry systems with different formats (coin, cylindrical, pouch, prismatic) and capacity levels ranging from 4.5×10^{-2} Ah (Cambridge coin cells) to 13 Ah (SJTU packs). Among these, we treat 16 datasets as *known* during training: RWTH, ISU_ILCC, MICH_EXP, MICH, Stanford, Stanford_2, XJTU, CALCE, HUST, MATR, NA-ion, ZN-coin, UL_PUR, HNEI, Tongji, and SNL. For these datasets, all available cells and their full degradation trajectories are used to construct training and validation windows according to the 192-step protocol above (i.e., every valid 192-point slice of a trajectory contributes one training sample, with 96-step input and 96-step target).

The same windowing procedure is also used to create evaluation windows on these known datasets. Thus, for each method, we can report performance both at the window level (e.g., MAE, RMSE, MAPE averaged over all windows) and at the cell level (aggregating over all windows belonging to the same cell), providing an in-distribution assessment of predictive accuracy under the same chemistries, formats, and capacity ranges as those encountered during training. All auxiliary analyses based on charge–discharge physical features (current and voltage statistics) and degradation features (DF.1–DF.35) are likewise computed *only* on this training subset, ensuring that no information from held-out datasets leaks into model fitting or feature selection.

unknown datasets for zero-shot evaluation. To rigorously test the zero-shot generalization (ZSL) capability of the proposed TSFM-based forecaster, we designate four datasets in Table 1 as *unknown* during training and use them exclusively for validation:

- **EOCV2** (NMC / Carbon–SiO, 18650 cylindrical): this dataset employs an NMC cathode paired with a carbon–SiO composite anode, a chemistry combination that never appears in the training pool. In addition, it spans a wide temperature range (0 °C, 10 °C, 25 °C, 40 °C) and contains a very large number of cycles (1,286,612 in total), making it a stringent cross-chemistry and cross-condition test for zero-shot forecasting.

- **WZU** (LFP / Graphite, mixed cells and parallel packs): although LFP/graphite chemistry is present in the training data (e.g., HUST and MATR), the WZU dataset differs significantly in both capacity and operating conditions. It includes cells and parallel packs with nominal capacities around 1 Ah, 0.8 Ah, and 3 Ah, as well as strongly seasonal outdoor operating temperatures. By excluding WZU from training, we test whether the model can transfer from laboratory-style LFP data to more complex field-like LFP scenarios with different capacity scales and environmental variability.

- **Cambridge** (LCO / Graphite, LR2032 coin cells): this dataset consists of very small coin cells (capacity ≈ 0.045 Ah) under multiple temperatures. Its cell format (coin) and capacity scale are far from the cylindrical and prismatic cells that dominate the training pool. Treating Cambridge as unknown therefore probes the model's robustness to drastic changes in cell format and absolute capacity, i.e., its ability to extrapolate learned degradation patterns down to miniature cells.

- **SJTU** (multi-chemistry IFR32135 packs, LFP/NMC, 13 Ah): the SJTU dataset represents large-format IFR32135 cells in multi-chemistry packs (LFP/NMC) with extremely high nominal capacity (13 Ah) and very long operation (487,200 recorded cycles). None of the training datasets reach such capacity, pack configuration, or cycle count. We therefore treat SJTU as a strongly out-of-distribution benchmark to assess whether the model can produce meaningful degradation forecasts for grid-scale systems whose capacity and lifetime are orders of magnitude larger than the cells used for training.

Crucially, all windows derived from EOCV2, WZU, Cambridge, and SJTU are *completely removed* from the training corpus: they are not used for parameter learning, hyperparameter tuning, degradation feature statistics, or physical feature analysis. During evaluation, we apply the trained forecaster to these four datasets in a pure zero-shot manner, using the same 192-step window protocol (96-step context and 96-step forecast) as for the known datasets. This design allows us to disentangle two validation regimes:

- **Within-support evaluation**: performance on datasets whose chemistries, capacities, and formats are represented in the training pool (e.g., RWTH, ISU_ILCC, CALCE, MATR, Tongji, SNL). This measures how well the model fits and interpolates within the training domain.

- **unknown-dataset evaluation (zero-shot)**: performance on EOCV2, WZU, Cambridge, and SJTU, which differ from the training data along at least one of the following axes: chemistry combination (e.g., NMC with SiO anode), cell format (coin vs cylindrical/prismatic), capacity scale (from 0.045 Ah to 13 Ah), and operating conditions (seasonal outdoor vs controlled laboratory environments). This regime evaluates the model's ability to transfer the learned degradation representations across chemistries, formats, and capacity ranges without any task-specific fine-tuning.

By aligning the window-based forecasting protocol across all datasets while carefully separating known and unknown sources at the dataset level, our experimental design directly tests the proposed method's capability for cross-chemistry and cross-capacity zero-shot learning, rather than relying on narrow, dataset-specific tuning.

Evaluation Metrics and Friedman Test

In this study, we evaluate the performance of the proposed model using several key metrics, including Root Mean Squared Error (RMSE) , Mean Absolute Error (MAE) , and Mean Absolute Percentage Error (MAPE) . Additionally, we perform a Friedman Test to compare the models' performance across different cells, based on the average RMSE and MSE scores for each method. The following sections outline these evaluation metrics and the Friedman test process in detail.

Evaluation Metrics

Root Mean Squared Error (RMSE) : The RMSE is a commonly used metric to measure the average magnitude of the error between predicted values and actual values. It is defined as:

$$\text{RMSE} = \sqrt{\frac{1}{N}\sum_{i=1}^{N}(y_i - \hat{y}_i)^2}$$

where y_i represents the true value, $\hat{y}_i$ represents the predicted value, and N is the total number of data points.

Mean Absolute Error (MAE) : The MAE measures the average magnitude of errors in a set of predictions, without considering their direction. It is defined as:

$$\text{MAE} = \frac{1}{N}\sum_{i=1}^{N}|y_i - \hat{y}_i|$$

Mean Absolute Percentage Error (MAPE) : The MAPE is a metric used to express the accuracy as a percentage. It is defined as:

$$\text{MAPE} = \frac{1}{N}\sum_{i=1}^{N}\left|\frac{y_i - \hat{y}_i}{y_i}\right| \times 100$$

where y_i is the true value and $\hat{y}_i$ is the predicted value.

Friedman Test for Model Comparison

In addition to the point-wise error metrics, we perform a Friedman Test to assess the differences in model performance across various methods. The Friedman test is a non-parametric statistical test used to compare the performance of multiple models (methods) based on their average rankings for each cell. The test is particularly useful when comparing more than two models, especially when the data does not follow a normal distribution.

The process for performing the Friedman test is as follows:

- **Input Data**: The input to the test consists of the models' performance on each dataset (cell). Specifically, we use the RMSE and MAE values computed for each model on each cell.

- **Ranking**: For each dataset (cell), we rank the models based on their performance in terms of RMSE or MAE. The model with the best performance (i.e., the lowest error) is assigned the rank of 1, the second-best model gets rank 2, and so on.

- **Compute the Friedman Test Statistic**: For each metric (RMSE and MAE), we compute the average ranks across all datasets (cells). The Friedman statistic is given by:

$$\chi_F^2 = \frac{12}{k \cdot m \cdot (m+1)}\sum_{j=1}^{m} R_j^2 - 3 \cdot k \cdot (m+1)$$

  where k is the number of datasets (cells), m is the number of models, and R_j is the sum of the ranks for each model across all datasets.

- **Interpretation**: A significant Friedman test result (typically when p-value ¡ 0.05) indicates that there are significant differences in the performance of the models. Post-hoc analysis, such as pairwise comparisons with corrections for multiple testing (e.g., using Nemenyi test), can be conducted to determine which models differ significantly from each other.

Algorithm: Friedman Test Process

The process of performing the Friedman test is illustrated in the following algorithm:

Algorithm3 Friedman Test for Model Comparison

Require: Performance values of models on each dataset (e.g., RMSE or MAE), where each row corresponds to a dataset and each column corresponds to a model.

Ensure: Test statistic χ_F^2 and the corresponding p-value for model comparison.

For each dataset i and model j, rank the models based on performance (lower error gets a higher rank).

Calculate the average rank for each model R_j across all datasets.

Compute the Friedman statistic:

$$\chi_F^2 = \frac{12}{k \cdot m \cdot (m+1)} \sum_{j=1}^{m} R_j^2 - 3 \cdot k \cdot (m+1)$$

where:

- k is the number of datasets (cells),
- m is the number of models,
- R_j is the sum of ranks for model j across all datasets.

Compute the p-value corresponding to the Friedman statistic using the Chi-square distribution with $m-1$ degrees of freedom.

if p-value ¡ 0.05 **then**

    Reject the null hypothesis and conclude that there is a significant difference in model performance.

else

    Fail to reject the null hypothesis and conclude that there is no significant difference in model performance.

end if

return χ_F^2, p-value.

The algorithm describes the steps for computing the Friedman test for model comparison based on average ranking, helping us determine whether the models exhibit significant performance differences across multiple datasets (cells).

Supplementary Note 7: Long-horizon rolling forecasting experiments

In typical practice, the model is optimized under a fixed training horizon, for example 96 steps, by minimizing point-wise errors such as mean squared error. The model either produces all future steps in a single forward pass, or is unrolled only over a limited number of autoregressive steps during training. When a much longer horizon is required at inference time, a natural strategy is to feed the model's own predictions back as inputs and perform recursive forecasting as in Eq. S71:

$$\hat{y}_{t+1} = f(x_{\leq t}), \quad \hat{y}_{t+2} = f(x_{\leq t}, \hat{y}_{t+1}), \quad \hat{y}_{t+3} = f(x_{\leq t}, \hat{y}_{t+1}, \hat{y}_{t+2}), \ldots \tag{S71}$$

In this autoregressive mode, the model gradually departs from the training distribution, because the historical context is increasingly dominated by its own prediction errors instead of true observations. This mismatch between training and inference, often described as exposure bias and error accumulation, drives the model into regions of the state space that have not been covered during training. Under a squared-error objective and given the intrinsic smoothing tendency of deep sequence models, the resulting behaviour is a contraction toward a stable mean trajectory, so that free-running forecasts converge to a fixed point and empirically collapse into a nearly straight line as the horizon grows.

To quantitatively and visually assess this phenomenon for battery capacity degradation forecasting, we perform dedicated long-horizon experiments on all methods, including the LoRA-adapted Timer model. All forecasters are first trained under a fixed window-based protocol with a base horizon of 96 steps: each degradation trajectory is segmented into windows of length 192, where the first 96 time steps in each window serve as the input context and the remaining 96 steps constitute the forecast target. During long-horizon evaluation, we keep this training setup unchanged, but at inference time we apply the recursive scheme in Eq. S71 to iteratively extend the forecast beyond the supervised horizon. Concretely, starting from an observed context of length 96, the model repeatedly consumes its latest 96 points (initially all ground truth, and then gradually replaced by its own predictions) and generates the next 96-step block, until the desired forecast length is reached. In this work, we consider two extended horizons: a medium-range horizon of 196 steps and a longer horizon of 384 steps, both strictly larger than the 96-step horizon known during training.

Due to space limitations in the main supplementary document, the detailed long-horizon results are reported in two separate files:

- `Long_horizon_results.pdf`, which visualizes representative multi-step free-running forecasts under horizons 196 and 384 for different datasets and methods, highlighting how the predicted capacity trajectories evolve as the autoregressive rollout proceeds;
- `table_results.zip`, which summarizes the corresponding quantitative metrics (e.g., MAE, RMSE and other aggregated indicators) for all methods, horizons, and datasets.

Together, these results provide a systematic evaluation of how each forecaster behaves when extrapolating far beyond its training horizon, and they complement our main findings on exposure bias, error accumulation and the robustness of the proposed Timer-based, LoRA-adapted framework for long-horizon battery capacity degradation prediction.

Note: Not all degradation data sequences are long enough to support long-horizon experiments. However, we will conduct these experiments for each available cell to the extent possible, ensuring that we maximize the coverage of long-horizon predictions across different datasets and cell types.

 WILEY-VCH

Supplementary Note 8: LIME-based temporal attribution maps

To visualize how the time-series a TSFM-based framework for strongly generalizable battery capacity forecasting, we design a LIME-based temporal attribution scheme, referred to as the *LIME map*. Instead of explaining static tabular features, we treat each time step in the look-back capacity window as an individual feature and apply LIME [1] to obtain a local linear approximation of the model output around that window. By scanning along the degradation trajectory, this procedure produces a two-dimensional importance map that highlights which portions of the past capacity sequence are most influential for the prediction of future capacity.

Consider a single cell with a cycle-wise discharge capacity sequence $\{y_t\}_{t=1}^{T}$ (in Ah). For a given look-back length L and prediction horizon S, we construct a set of non-overlapping windows, where each window consists of: (i) an input segment of length L used as model input, and (ii) a prediction segment of length S used to evaluate forecasting accuracy and define the LIME regression target. Within each window, the input segment is rescaled by a per-window min–max transform so that all time steps lie in a comparable normalized range, and LIME treats the L normalized time steps as L input features.

Algorithm : Construction of temporal LIME importance matrix

Algorithm4 Construction of LIME-based temporal attribution maps

---

Require: Fine-tuned model f_θ (Timer + LoRA), capacity sequence $\{y_t\}_{t=1}^{T}$, look-back length L, prediction horizon S, number of LIME samples N_{LIME}, number of windows to explain $N_{\text{exp}} \le N_w$.
Ensure: LIME importance matrix $\mathbf{W} \in \mathbb{R}^{N_w \times L}$ and per-window prediction metrics.

```
Initialize the list of window start indices 𝒮 ← [].
for s = 1, 1+S, 1+2S, ... do
    if s+L+S−1 ≤ T then
        Append s to 𝒮.
    else
        break.
    end if
end for
Let N_w ← |𝒮| be the number of windows.
Initialize a matrix X^LIME ∈ ℝ^(N_w×L).
for i = 1 to N_w do
    Let s_i be the i-th element of 𝒮.
    Extract the look-back segment y_in^(i) = (y_{s_i}, y_{s_i+1}, ..., y_{s_i+L−1}).
    Compute the per-window min and max as y_min^(i) = min_ℓ y_{in,ℓ}^(i) and y_max^(i) = max_ℓ y_{in,ℓ}^(i).
    Normalize each point by x_ℓ^(i) = (y_{in,ℓ}^(i) − y_min^(i)) / (y_max^(i) − y_min^(i) + ε) for ℓ = 1, ..., L, where ε > 0 avoids division by zero.
    Store the normalized vector x^(i) = (x_1^(i), ..., x_L^(i)) as the i-th row of X^LIME.
end for
Define the LIME regression target g(x) for any normalized input x ∈ ℝ^L as the average scaled prediction over the next S steps: g(x) = (1/S) Σ_{τ=1}^{S} ŷ_τ^(scaled)(x), where ŷ_τ^(scaled)(x) is the τ-th step of the model output produced by f_θ when fed with x.
Construct a LIME tabular explainer Explainer ← LIME_Tabular_Explainer(X^LIME, mode = "regression").
Initialize the importance matrix W ∈ ℝ^(N_w×L) with zeros.
for i = 1 to N_exp do
    Take the i-th normalized window x^(i) from X^LIME.
    Obtain a local LIME explanation ℰ_i ← Explainer.explain_instance(x^(i), g, num_features = L, num_samples = N_LIME).
    Extract feature–weight pairs {(k, w_k^(i))} from ℰ_i, where k is a feature index (time step) and w_k^(i) its local contribution.
    for each pair (k, w_k^(i)) in ℰ_i do
        if 1 ≤ k ≤ L then
            Set W_{i,k} ← w_k^(i).
        end if
    end for
end for
Optionally, for each window i, compare the de-normalized S-step prediction of f_θ with the ground-truth capacity segment to compute MAE, RMSE and R^2, and save these as per-window prediction metrics.
return W and the per-window metrics.
```

In practice, the importance matrix $\mathbf{W}$ is saved as a CSV file where each row corresponds to a window (identified by its starting

cycle index) and each column corresponds to one time step in the look-back window. By stacking these rows and visualizing **W** as a heatmap, we obtain the LIME map for a given model and cell.

Interpretation of the LIME maps

In the LIME map, each cell (i,k) encodes the contribution weight $W_{i,k}$ assigned by LIME to the k-th time step in the look-back window of the i-th prediction window. We typically index $k=1$ as the oldest time step and $k=L$ as the most recent time step before the prediction horizon, so that the vertical axis represents *relative lag* and the horizontal axis represents *window location* along the degradation trajectory (e.g., the cycle index at which the forecast starts).

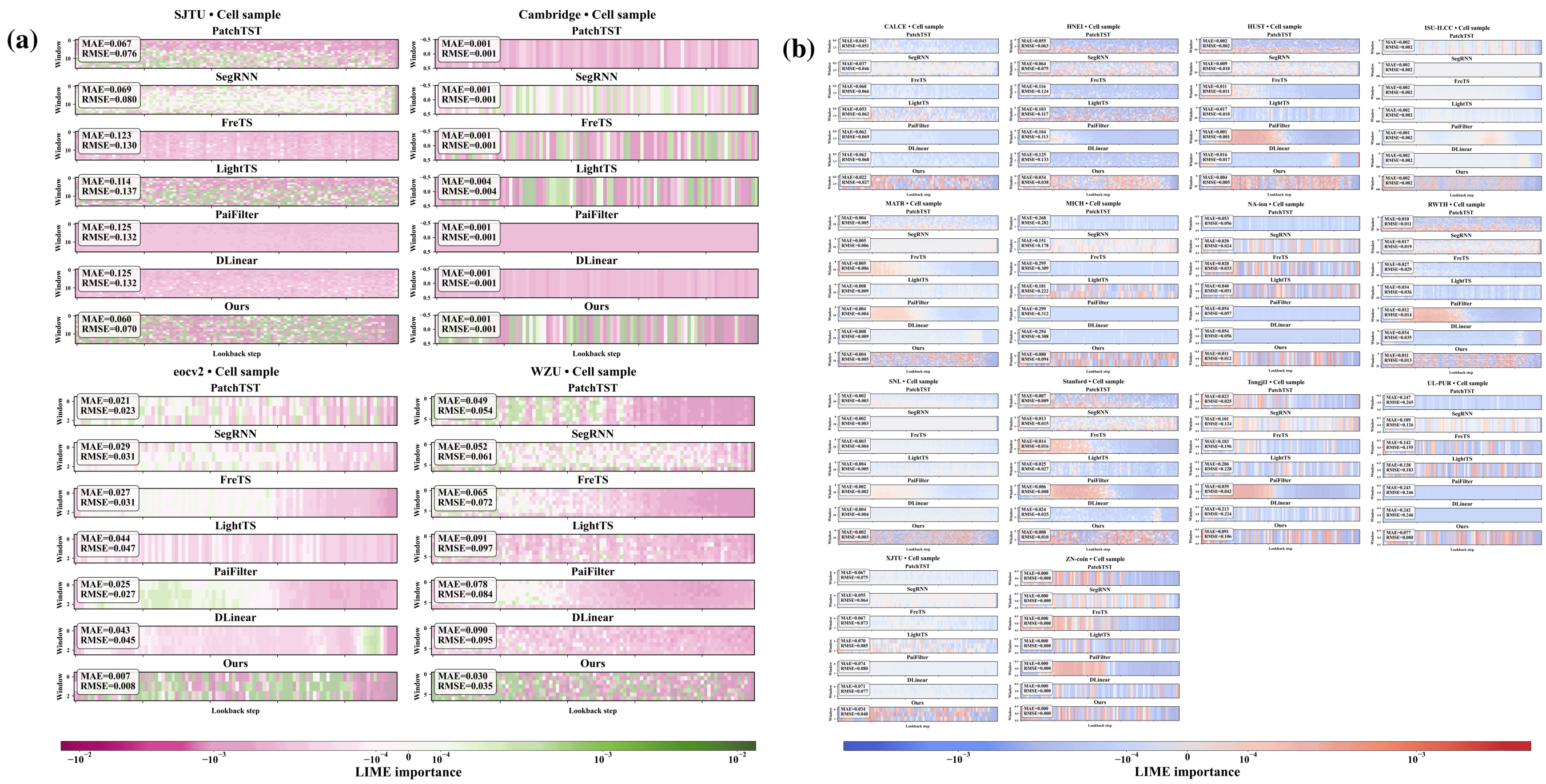

FIG. S6: **LIME-based interpretation of degradation forecasting.** **(a)** Comparison of LIME importance maps for the proposed method and competing baselines on unknown datasets. **(b)** Comparison of LIME importance maps for the proposed method and competing baselines on known datasets.

Using a diverging colormap, the LIME map can be read as follows:

- **Sign of the weight.** A *positive* value $W_{i,k}>0$ (typically shown in warm colors) indicates that, in the local neighborhood of window i, a higher normalized capacity at lag k tends to *increase* the surrogate prediction $g(\mathbf{x})$, i.e., it contributes positively to the predicted future capacity over the next S cycles. Conversely, a *negative* value $W_{i,k}<0$ (cool colors) indicates that higher capacity at that time step tends to *decrease* the predicted average future capacity, or equivalently that lower capacity at that time step is used by the model as a warning signal for future degradation.
- **Magnitude of the weight.** The absolute value $|W_{i,k}|$ reflects the *local importance* of the k-th time step for the prediction starting at window i. Large $|W_{i,k}|$ values correspond to time steps to which the model is most sensitive, while values close to zero indicate that perturbing that time step has little effect on the local prediction.
- **Temporal patterns.** Vertical patterns in the LIME map (along the lag axis) show which parts of the look-back window the model relies on: for example, strong weights near $k=L$ indicate that the model mainly uses very recent cycles, whereas non-negligible weights at smaller k reveal a longer effective memory. Horizontal patterns (along the window index) reveal how the model's focus shifts across different stages of the degradation trajectory, e.g., around the knee point or during late-life acceleration.
- **Shape-based interpretation.** Because each look-back segment is normalized independently before applying LIME, the attribution weights emphasize *shape* and *relative changes* in the capacity curve rather than absolute capacity values. This makes the LIME maps comparable across cells and datasets and allows us to analyze how the model learns degradation patterns under heterogeneous chemistries and operating conditions.

By generating such LIME maps for a wide range of battery datasets and models, we can visually compare how different forecasting methods attend to the degradation sequence, and quantitatively analyze the similarity between their attribution patterns (e.g., via distributional distances between **W** matrices), which is further exploited in the main text for model comparison and evaluation.

Fig. S6(a) compares the LIME maps of the proposed method and the baselines on the four unknown datasets (EOCV2, SJTU, WZU, and Cambridge). Unlike several baselines, which tend to assign uniformly positive importance to the most recent part of the lookback window and weak or noisy importance to earlier steps, our model produces structured patterns of positive and negative importance over specific segments of the history. Combined with the forecasting results in Fig. 3(a), this indicates that the unified TSFM attains its superior accuracy by selectively focusing on degradation-relevant portions of the trajectory, rather than relying solely on short-term trends. Fig. S6(b) presents analogous LIME maps for the 16 known datasets. Although the attribution patterns differ across chemistries, form factors, and operating conditions, the model consistently achieves high accuracy, suggesting that it has learned distinct yet meaningful attribution modes tailored to different battery families while still leveraging the shared trajectory-level degradation patterns revealed by the DF analysis.

Supplementary Note 9: LIME-based quantitative analysis of attribution maps

In Supplementary Note 8, we introduced the construction of LIME-based temporal attribution maps for sequence forecasting, which highlight the relative importance of different history steps in the look-back window. In this section, we quantitatively assess whether these learned attribution patterns are consistent across models and whether they correlate with predictive performance.

Concretely, we treat the LIME map of our proposed method (`Ours`) as a reference attribution pattern, and compare the attribution distributions of several strong baselines, including PatchTST, SegRNN, FreTS, LightTS, PaiFilter, and DLinear. For each cell that is jointly available across all methods, we compute: (i) the Jensen–Shannon (JS) divergence between the LIME importance distribution of a baseline method and that of `Ours`, and (ii) the logarithm of the mean absolute percentage error (log(MAPE)) of that method on the same cell. By plotting JS divergence versus log(MAPE) for each dataset and method, we can test whether methods whose attribution patterns are more similar to `Ours` also tend to achieve better forecasting accuracy.

Notation. Let $\mathscr{M}$ denote the set of all forecasting methods under comparison and let $m^\star \in \mathscr{M}$ denote our method (`Ours`), which is used as the reference model. Let $\mathscr{C}$ be the set of cells that are present in the LIME results of all methods. For a given method $m \in \mathscr{M}$ and cell $c \in \mathscr{C}$, let $\mathbf{L}^{(m,c)} \in \mathbb{R}^{N_w \times L}$ be the matrix of LIME importance scores, where N_w is the number of windows and L is the look-back length. We denote by

$$\mathbf{v}^{(m,c)} = \operatorname{vec}\big(\mathbf{L}^{(m,c)}\big) \in \mathbb{R}^{N_w L} \tag{S72}$$

the flattened vector of all LIME scores for method m on cell c.

For each cell c, let $\mathbf{y}_{\text{true}}^{(m,c)}$ and $\mathbf{y}_{\text{pred}}^{(m,c)}$ be the concatenated ground-truth and predicted capacity values (in the raw domain) over all forecasting windows produced by method m. The cell-wise mean absolute percentage error (MAPE) is defined as

$$\text{MAPE}^{(m,c)} = 100 \times \frac{1}{n_c} \sum_{j=1}^{n_c} \frac{\big|y_{\text{pred},j}^{(m,c)} - y_{\text{true},j}^{(m,c)}\big|}{\max\big(|y_{\text{true},j}^{(m,c)}|, \varepsilon\big)}, \tag{S73}$$

where n_c is the number of prediction points for cell c and $\varepsilon > 0$ is a small constant (e.g., 10^{-8}) that prevents division by zero when the true capacity is very small. For numerical stability and to compress the dynamic range, we work with the logarithmic performance variable

$$\text{logMAPE}^{(m,c)} = \log\big(\text{MAPE}^{(m,c)} + \varepsilon\big). \tag{S74}$$

To compare attribution patterns, we convert each LIME vector $\mathbf{v}^{(m,c)}$ into a discrete probability distribution by histogramming. Let B be the number of bins used to discretize the LIME scores (here $B = 50$). For a given cell c, let $\{b_1, \ldots, b_{B+1}\}$ be a shared set of bin edges computed from the pooled LIME scores $\mathbf{v}^{(m^\star,c)}$ and $\mathbf{v}^{(m,c)}$. Let $p^{(c)} \in \mathbb{R}^B$ and $q^{(m,c)} \in \mathbb{R}^B$ denote the normalized histograms of $\mathbf{v}^{(m^\star,c)}$ and $\mathbf{v}^{(m,c)}$ respectively, i.e.,

$$p_k^{(c)} = \frac{h_k\big(\mathbf{v}^{(m^\star,c)}\big) + \delta}{\sum_{\ell=1}^{B}\big(h_\ell\big(\mathbf{v}^{(m^\star,c)}\big) + \delta\big)}, \quad q_k^{(m,c)} = \frac{h_k\big(\mathbf{v}^{(m,c)}\big) + \delta}{\sum_{\ell=1}^{B}\big(h_\ell\big(\mathbf{v}^{(m,c)}\big) + \delta\big)}, \tag{S75}$$

where $h_k(\cdot)$ counts the number of entries that fall into bin k, and $\delta > 0$ is a small pseudo-count to avoid empty bins. We then define the Jensen–Shannon divergence between the two LIME distributions as

$$\text{JS}^{(m,c)} = D_{\text{JS}}\big(p^{(c)} \,\|\, q^{(m,c)}\big) = \tfrac{1}{2} D_{\text{KL}}\big(p^{(c)} \,\|\, m^{(m,c)}\big) + \tfrac{1}{2} D_{\text{KL}}\big(q^{(m,c)} \,\|\, m^{(m,c)}\big), \tag{S76}$$

where $m^{(m,c)} = \frac{1}{2}\big(p^{(c)} + q^{(m,c)}\big)$ and $D_{\text{KL}}(\cdot\|\cdot)$ is the Kullback–Leibler divergence. By construction, $\text{JS}^{(m,c)} \geq 0$ and equals zero if and only if the two histograms coincide exactly; larger values indicate that the baseline method assigns LIME importance in a way that is distributionally farther from our reference model.

In the implementation, cells are grouped by dataset when visualizing the relationship between LIME distribution distance and predictive accuracy. For example, multiple datasets such as `Tongji1`, `Tongji2`, and `Tongji3` are treated as a single normalized dataset label (`Tongji`) for aggregation and visualization purposes. For each dataset and each method, we then plot $\text{JS}^{(m,c)}$ on the x-axis and $\text{logMAPE}^{(m,c)}$ on the y-axis, together with a least-squares linear trend line and the Pearson correlation coefficient r between the two quantities. A negative r indicates that cells whose LIME distributions are closer to those of the reference model tend to yield lower prediction error, supporting the interpretive consistency of the LIME maps.

Algorithm5 Computation of LIME JS divergence and log(MAPE) pairs

Require: Set of methods $\mathscr{M}$, reference method $m^\star \in \mathscr{M}$, LIME matrices $\mathbf{L}^{(m,c)}$ for all $m \in \mathscr{M}$ and $c \in \mathscr{C}$, prediction scatter data (true and predicted capacities) for all methods and cells, number of histogram bins B.

Ensure: For each dataset, method and cell, pairs $\left(\mathrm{JS}^{(m,c)}, \log\mathrm{MAPE}^{(m,c)}\right)$.

1: Determine the set of common cells $\mathscr{C} = \bigcap_{m\in\mathscr{M}} \mathscr{C}_m$, where $\mathscr{C}_m$ is the set of cells for which method m has a valid LIME matrix.
2: **for** each cell $c \in \mathscr{C}$ **do**
3: Parse the dataset name from the cell identifier and normalize it to a canonical dataset label (e.g., map `Tongji1/2/3` to `Tongji`).
4: Let $\mathbf{L}^{(m^\star,c)} \in \mathbb{R}^{N_w \times L}$ be the LIME matrix of the reference method and flatten it to $\mathbf{v}^{(m^\star,c)} = \mathrm{vec}\left(\mathbf{L}^{(m^\star,c)}\right)$.
5: **for** each method $m \in \mathscr{M}$ with $m \neq m^\star$ **do**
6: **if** method m has no valid LIME matrix for cell c **then**
7: **continue** to the next method.
8: **end if**
9: Flatten the LIME matrix $\mathbf{L}^{(m,c)}$ into $\mathbf{v}^{(m,c)} = \mathrm{vec}\left(\mathbf{L}^{(m,c)}\right)$.
10: Concatenate the LIME vectors $\mathbf{v}^{\mathrm{all}} = \left(\mathbf{v}^{(m^\star,c)}, \mathbf{v}^{(m,c)}\right)$ and compute the global minimum and maximum over all entries. If all entries are numerically identical, set $\mathrm{JS}^{(m,c)} = 0$ and skip to Step 15.
11: Construct B equally spaced bin edges covering the range of $\mathbf{v}^{\mathrm{all}}$ and compute the histogram counts $\mathbf{h}^{(m^\star,c)}$ and $\mathbf{h}^{(m,c)}$ for $\mathbf{v}^{(m^\star,c)}$ and $\mathbf{v}^{(m,c)}$, respectively.
12: Add a small pseudo-count δ to each bin and normalize to obtain discrete distributions $p^{(c)}$ and $q^{(m,c)}$.
13: Form the mixture distribution $m^{(m,c)} = \frac{1}{2}\left(p^{(c)} + q^{(m,c)}\right)$.
14: Compute the Jensen–Shannon divergence

$$\mathrm{JS}^{(m,c)} = \frac{1}{2}\sum_{k=1}^{B} p_k^{(c)} \log\left(\frac{p_k^{(c)}}{m_k^{(m,c)}}\right) + \frac{1}{2}\sum_{k=1}^{B} q_k^{(m,c)} \log\left(\frac{q_k^{(m,c)}}{m_k^{(m,c)}}\right).$$

15: From the prediction scatter data of method m on cell c, collect all pairs $\left(y_{\mathrm{true},j}^{(m,c)}, y_{\mathrm{pred},j}^{(m,c)}\right)$ and compute the MAPE $\mathrm{MAPE}^{(m,c)}$ and $\log\mathrm{MAPE}^{(m,c)} = \log\left(\mathrm{MAPE}^{(m,c)} + \varepsilon\right)$.
16: Store the pair $\left(\mathrm{JS}^{(m,c)}, \log\mathrm{MAPE}^{(m,c)}\right)$ under the corresponding dataset label and method m.
17: **end for**
18: **end for**
19: **return** all stored $\left(\mathrm{JS}^{(m,c)}, \log\mathrm{MAPE}^{(m,c)}\right)$ pairs.

Algorithm : Computation of LIME JS divergence and log(MAPE) pairs

For each dataset, we then visualize the relationship between LIME distribution distance and predictive accuracy by plotting $\mathrm{JS}^{(m,c)}$ versus $\log\mathrm{MAPE}^{(m,c)}$ for all cells and methods, using distinct colors for different methods and annotating the Pearson correlation coefficient between the two variables in the legend. In these scatter plots, points near the left bottom corner correspond to methods whose attribution distributions are close to those of the reference model and whose errors are small. Empirically, we observe that methods with lower LIME JS divergence from `Ours` tend to achieve lower log(MAPE), especially on the more challenging datasets, which supports the conclusion that the learned LIME attribution patterns are not arbitrary but are closely tied to forecasting performance.

Supplementary Note 10: LoRA-based contrastive fine-tuning scheme

Building on Supplementary Note 4, which introduces the basic idea of Low-Rank Adaptation (LoRA)[2], this section provides a detailed description of how LoRA is integrated into the Timer backbone and how the model is fine-tuned for battery discharge capacity degradation forecasting under a contrastive learning framework.

Model backbone and tokenization

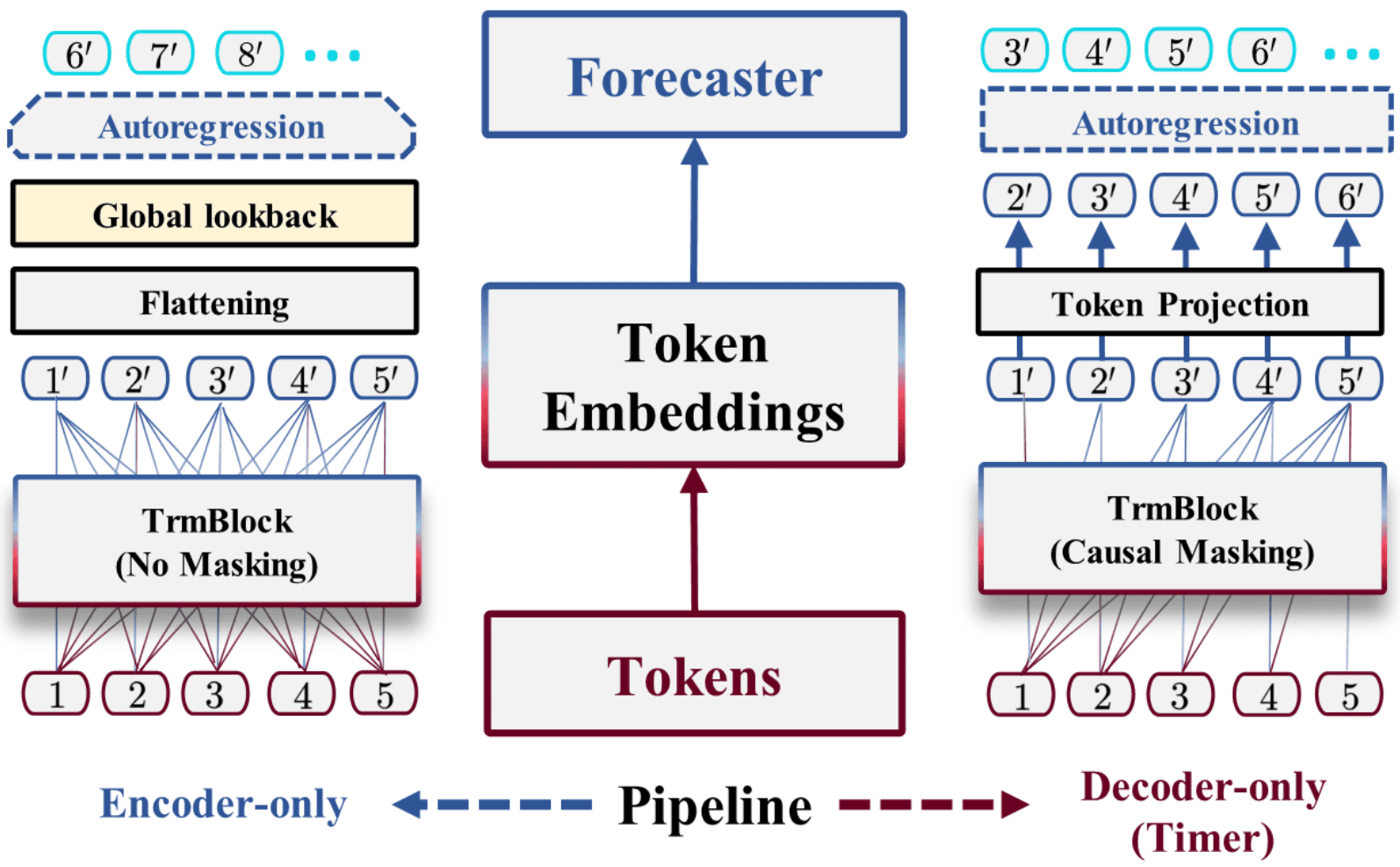

FIG. S7: **Architectures of Timer:** a Transformer-decoder-based forecasters.

As shown in **Fig.**S7 Timer is a decoder-only, generative pre-trained Transformer for time series forecasting. It tokenizes a univariate time series $\{x_t\}_{t=1}^{NS}$ into N consecutive segments (tokens) of length S,

$$\mathbf{s}_i = \{x_{(i-1)S+1}, \ldots, x_{iS}\} \in \mathbb{R}^S, \quad i = 1, \ldots, N, \tag{S77}$$

and applies a GPT-style autoregressive objective on these tokens. Given token embeddings $\mathbf{h}_i^0$ and L Transformer layers, the forward propagation is

$$\begin{aligned} \mathbf{h}_i^0 &= \mathbf{W}_e \mathbf{s}_i + \mathbf{TE}_i, \quad i = 1, \ldots, N, \\ \mathbf{H}^l &= \text{TrmBlock}(\mathbf{H}^{l-1}), \quad l = 1, \ldots, L, \\ \hat{\mathbf{s}}_{i+1} &= \mathbf{h}_{i+1}^L \mathbf{W}_d, \end{aligned} \tag{S78}$$

where $\mathbf{H}^l = \{\mathbf{h}_i^l\}_{i=1}^N$, $\mathbf{W}_e, \mathbf{W}_d \in \mathbb{R}^{D \times S}$ are the encoder/decoder matrices and $\mathbf{TE}_i$ denotes optional time embeddings. The pre-training loss is the token-wise mean squared error (MSE) between $\hat{\mathbf{s}}_i$ and $\mathbf{s}_i$, which enables flexible multi-step autoregressive forecasting during inference.

In our application, we adopt the open-source 84M-parameter Timer model (`thuml/timer-base-84m`) as the backbone. For fine-tuning, we use a fixed context length $L = 96$ and forecast horizon $H = 96$, which matches the base configuration of Timer and the windowing strategy described in the main text.

LoRA strategy and injected modules

We follow the standard LoRA formulation from Supplementary Note 4. For a given pre-trained weight matrix $\mathbf{W}_0 \in \mathbb{R}^{d_{\text{out}} \times d_{\text{in}}}$ inside Timer, LoRA introduces a low-rank update

$$\mathbf{W} = \mathbf{W}_0 + \Delta\mathbf{W} = \mathbf{W}_0 + \mathbf{B}\mathbf{A}, \tag{S79}$$

where $\mathbf{A} \in \mathbb{R}^{r \times d_{\text{in}}}$ and $\mathbf{B} \in \mathbb{R}^{d_{\text{out}} \times r}$ with $r \ll d_{\text{in}}, d_{\text{out}}$. During fine-tuning, only $\mathbf{A}$ and $\mathbf{B}$ are updated, while $\mathbf{W}_0$ remains frozen, which significantly reduces memory and computation cost.

In practice, we inject LoRA modules into the key projection layers that control self-attention and the feed-forward network of Timer:

- self-attention projections: `q_proj`, `k_proj`, `v_proj`, `o_proj`;
- feed-forward projections: `up_proj`, `gate_proj`, `down_proj`.

These modules determine how the model attends over historical capacity trajectories and how it transforms hidden states, and are therefore most relevant for capturing long-term degradation patterns and subtle changes in slope or curvature.

We use a single LoRA configuration across all experiments:

$$r = 8, \tag{S80}$$
$$\alpha_{\text{LoRA}} = 16, \tag{S81}$$
$$p_{\text{drop}} = 0.05, \tag{S82}$$

with `task_type = CAUSAL_LM`. Under this setting, the additional LoRA parameters amount to only about 1.3% of the total parameter count of Timer, while still providing sufficient capacity to adapt the model to the battery domain.

Construction of contrastive training samples

To inject physically meaningful structure into the learned representations, we combine normalized capacity trajectories with per-cycle physical descriptors derived from charge–discharge data (see the Supplementary Note on physical feature extraction).

For a given cell, the preprocessed CSV file contains the cycle index `cycle_number`, the discharge capacity `discharge_capacity_in` and a set of $d_{\text{phys}} = 9$ statistical descriptors per cycle (e.g., current and voltage mean, standard deviation, skewness, kurtosis and RMS, and discharge duration). Let $\mathbf{z}_t \in \mathbb{R}^{1+d_{\text{phys}}}$ denote the feature vector at cycle t, where the first entry is the discharge capacity and the remaining entries are physical features. We first apply a Min–Max normalization jointly over all non-index columns:

$$\tilde{\mathbf{z}}_t = \text{MinMaxNorm}(\mathbf{z}_t), \quad t = 1, \ldots, T. \tag{S83}$$

From the normalized matrix we extract:

- the *capacity series* $\tilde{c}_t$ (the normalized `discharge_capacity_in_Ah`),
- the *physical feature vectors* $\tilde{\mathbf{f}}_t \in \mathbb{R}^{d_{\text{phys}}}$ (all remaining columns).

Given a context length L and forecast horizon H (both set to 96), each training sample is defined by a starting index s:

$$\mathbf{c}^{(\text{in})} = (\tilde{c}_s, \tilde{c}_{s+1}, \ldots, \tilde{c}_{s+L-1}), \tag{S84}$$
$$\mathbf{c}^{(\text{out})} = (\tilde{c}_{s+L}, \tilde{c}_{s+L+1}, \ldots, \tilde{c}_{s+L+H-1}). \tag{S85}$$

These sequences correspond to the input and target for supervised capacity forecasting.

In addition, we construct a triplet of physical-feature windows from the same cell for contrastive learning:

$$\mathbf{F}^{(\text{anchor})} = (\tilde{\mathbf{f}}_s, \ldots, \tilde{\mathbf{f}}_{s+L-1}), \tag{S86}$$
$$\mathbf{F}^{(\text{pos})} = (\tilde{\mathbf{f}}_{s+H}, \ldots, \tilde{\mathbf{f}}_{s+H+L-1}), \tag{S87}$$
$$\mathbf{F}^{(\text{neg})} = (\tilde{\mathbf{f}}_{s+3H}, \ldots, \tilde{\mathbf{f}}_{s+3H+L-1}), \tag{S88}$$

where the positive window is delayed by one forecast horizon (similar degradation stage but different cycles), and the negative window is sampled from a temporally distant segment (wrapped cyclically via modulo indexing when needed). This design reflects a practical perspective: in real BESS operation, segments close in time tend to share similar operating conditions and degradation levels, whereas far-apart segments are more likely to correspond to different usage regimes or aging stages.

To feed physical descriptors into Timer, we use a small feature-expansion network

$$\phi : \mathbb{R}^{d_{\text{phys}}} \to \mathbb{R}^{D}, \qquad \phi(\mathbf{f}) = \mathbf{W}_\phi \mathbf{f} + \mathbf{b}_\phi, \tag{S89}$$

implemented as a single linear layer (`FeatureExpansionNN`) with

$$d_{\text{phys}} = 9, \qquad D = 1024. \tag{S90}$$

Applied at each time step, ϕ maps $\mathbf{F}^{(\cdot)}$ into sequences $\Phi(\mathbf{F}^{(\cdot)}) \in \mathbb{R}^{L \times D}$, which are provided to Timer as `inputs_embeds` in the contrastive branch.

Joint contrastive and supervised objective

Each mini-batch forward pass consists of two coupled branches:

Supervised capacity forecasting. The normalized capacity input $\mathbf{c}^{(\text{in})}$ is fed to Timer through its standard time-series input interface. The model produces a predicted sequence $\hat{\mathbf{c}}^{(\text{out})} \in \mathbb{R}^H$, and we define the supervised loss as

$$\mathscr{L}_{\text{sup}} = \frac{1}{H}\sum_{h=1}^{H}\left(\hat{c}_h^{(\text{out})} - c_h^{(\text{out})}\right)^2. \tag{S91}$$

This term drives the model to accurately predict future discharge capacities, which is the primary requirement in battery lifetime prognosis.

Contrastive representation learning from physical features. The embedded sequences $\Phi(\mathbf{F}^{(\text{anchor})})$, $\Phi(\mathbf{F}^{(\text{pos})})$ and $\Phi(\mathbf{F}^{(\text{neg})})$ are fed into Timer via `inputs_embeds`, and we obtain sequence-level representations $\mathbf{z}^{(\text{anchor})}$, $\mathbf{z}^{(\text{pos})}$ and $\mathbf{z}^{(\text{neg})}$ by reshaping the model logits. To improve robustness, we add Gaussian perturbations

$$\tilde{\mathbf{z}}^{(\text{anchor})} \sim \mathscr{N}\left(\mathbf{z}^{(\text{anchor})}, 0.1^2\mathbf{I}\right), \tag{S92}$$

$$\tilde{\mathbf{z}}^{(\text{pos})} \sim \mathscr{N}\left(\mathbf{z}^{(\text{pos})}, 0.1^2\mathbf{I}\right), \tag{S93}$$

$$\tilde{\mathbf{z}}^{(\text{neg})} \sim \mathscr{N}\left(\mathbf{z}^{(\text{neg})}, 0.1^2\mathbf{I}\right), \tag{S94}$$

and compute cosine similarities

$$s_{\text{ap}} = \cos\left(\tilde{\mathbf{z}}^{(\text{anchor})}, \tilde{\mathbf{z}}^{(\text{pos})}\right), \quad s_{\text{an}} = \cos\left(\tilde{\mathbf{z}}^{(\text{anchor})}, \tilde{\mathbf{z}}^{(\text{neg})}\right). \tag{S95}$$

With temperature $\tau = 0.07$, the contrastive loss for a batch of size B is

$$\mathscr{L}_{\text{cl}} = -\frac{1}{B}\sum_{i=1}^{B}\log\frac{\exp(s_{\text{ap},i}/\tau)}{\exp(s_{\text{ap},i}/\tau) + \exp(s_{\text{an},i}/\tau)}, \tag{S96}$$

where the positive pair promotes similarity between physically nearby segments, and the negative pair discourages similarity with distant segments along the same degradation trajectory.

Overall objective. The final training objective combines the two branches:

$$\mathscr{L}_{\text{total}} = \alpha\,\mathscr{L}_{\text{cl}} + \beta\,\mathscr{L}_{\text{sup}}, \tag{S97}$$

with fixed weights

$$\alpha = 0.2, \qquad \beta = 0.8. \tag{S98}$$

Thus, capacity forecasting remains the main task, while contrastive learning regularizes the representation space so that Timer organizes cycles according to their physical similarity and degradation stage. This is particularly important in real BESS applications, where operating conditions (current, voltage profile, and cycle duration) directly influence degradation trajectories.

Optimization setup and practical training protocol

For reproducibility and clarity, we summarize the main hyperparameters used in our LoRA-based contrastive fine-tuning scheme:

- **Base model:** `thuml/timer-base-84m` (84M parameters).
- **LoRA configuration:** rank $r = 8$; LoRA scaling $\alpha_{\text{LoRA}} = 16$; dropout $p_{\text{drop}} = 0.05$; `task_type = CAUSAL_LM`; target modules {`q_proj`, `k_proj`, `v_proj`, `o_proj`, `up_proj`, `gate_proj`, `down_proj`}.
- **Feature expansion network:** `FeatureExpansionNN(input_dim = 9, expanded_dim = 1024).`
- **Windowing:** context length $L = 96$, forecast horizon $H = 96$, built on the processed per-cell feature CSVs.
- **Optimization:** Adam optimizer for both the LoRA-augmented Timer and the feature-expansion network, learning rate 1×10^{-5}, batch size 64, number of epochs per file 3.
- **Device:** GPU training when available (`device = "cuda"`), otherwise CPU.

Operationally, we iterate over all processed feature folders under `data/processed_features_data`, treat each CSV file (one cell) as a long degradation trajectory, and construct sliding-window samples according to the above scheme. For each file, we run contrastive + supervised training for 3 epochs and log the final contrastive loss, supervised loss, and total loss in a CSV log. After completing all files in a folder, we save the updated LoRA weights of the Timer model for subsequent evaluation and deployment.

This design allows the pre-trained Timer backbone to be adapted in a parameter-efficient way to heterogeneous battery datasets while explicitly encoding physical charge–discharge characteristics into the learned representations, which is crucial for robust capacity degradation forecasting and health management in real-world battery energy storage systems.

Supplementary Note 11: Deployment feasibility and computational cost

Because LoRA reduces the number of trainable parameters during adaptation but does not remove the need to execute the Timer backbone at inference time, we additionally profiled the deployment cost of the proposed forecaster under the same 96-cycle context and 96-cycle forecasting setting used in the main experiments. Importantly, inference uses only the historical capacity sequence; the charge–discharge physical descriptors and the contrastive branch are used only during training and are not required during deployment. The full fp32 Timer backbone occupies approximately 336.6 MB on disk, while the LoRA adapter occupies approximately 4.47 MB. On an NVIDIA GeForce RTX 3060 Laptop GPU, Timer+LoRA required 0.171 GFLOPs and 14.16 ms per single-window forecast, with a peak allocated memory of 432.5 MB. This footprint is larger than that of the lightweight forecasting baselines, indicating that the present 84M-parameter implementation is better suited to cloud-based BMS analytics, fleet-level health monitoring, or enhanced edge platforms equipped with neural-network accelerators, rather than direct deployment on conventional low-power microcontrollers. For strictly MCU-level deployment, the present framework should be combined with model compression techniques such as quantization, structured pruning, or teacher–student distillation.

TABLE S2: **Inference cost and deployment footprint under the 96-cycle context and 96-cycle forecasting protocol.** Latency and peak-memory values were measured with batch size 1 on an NVIDIA GeForce RTX 3060 Laptop GPU using 10 warm-up runs and 50 repeated inference runs. For Timer+LoRA, only the capacity forecasting branch is evaluated at inference; the physical-feature contrastive branch is not used during deployment.

Model	Params (M)	Trainable / Adapter Params (M)	Storage (MB)	FLOPs (G)	Latency (ms)	Peak Memory (MB)
Timer-base-84M + LoRA	85.256	1.114	336.58 + 4.47	0.1706	14.16 ± 0.92	432.54
DLinear	0.0186	0.0186	0.0745	3.70×10^{-5}	0.316 ± 0.009	9.65
LightTS	0.0106	0.0106	0.0424	2.43×10^{-5}	1.113 ± 0.224	9.63
FreTS	3.237	3.237	12.95	0.00640	0.795 ± 0.080	22.67
SegRNN	0.00709	0.00709	0.0284	1.24×10^{-5}	0.770 ± 0.141	17.08
PaiFilter	0.0496	0.0496	0.198	9.87×10^{-5}	0.539 ± 0.034	9.77
PatchTST	0.1138	0.1138	0.455	7.99×10^{-4}	4.969 ± 0.428	10.70

Supplementary Note 12: Stage-wise verification of capacity degradation forecasting

Battery capacity degradation does not necessarily proceed at a uniform rate throughout the entire aging process. Many cells exhibit a two-stage degradation pattern, consisting of a relatively slow and approximately linear capacity-fade stage followed by an accelerated degradation stage near the end of life. Because the accelerated stage strongly affects the remaining usable life of a battery, forecasting performance in this regime is particularly important for practical battery health management. We therefore conduct an additional stage-wise evaluation to directly assess the predictive performance of the proposed model in different degradation regimes.

Not all datasets in the unified corpus contain cells that reach a clearly observable accelerated degradation stage within the recorded cycling period. Accordingly, we select four representative datasets exhibiting evident two-stage degradation behavior: SJTU, HUST, Stanford, and MICH. For each cell, the capacity trajectory is divided into a slow-degradation stage and an accelerated-degradation stage according to the identified transition in the capacity-fade process. The forecasting errors of the proposed method and the comparison models are then evaluated separately in the two stages under the same 96-cycle context and 96-cycle forecasting setting used in the main experiments.

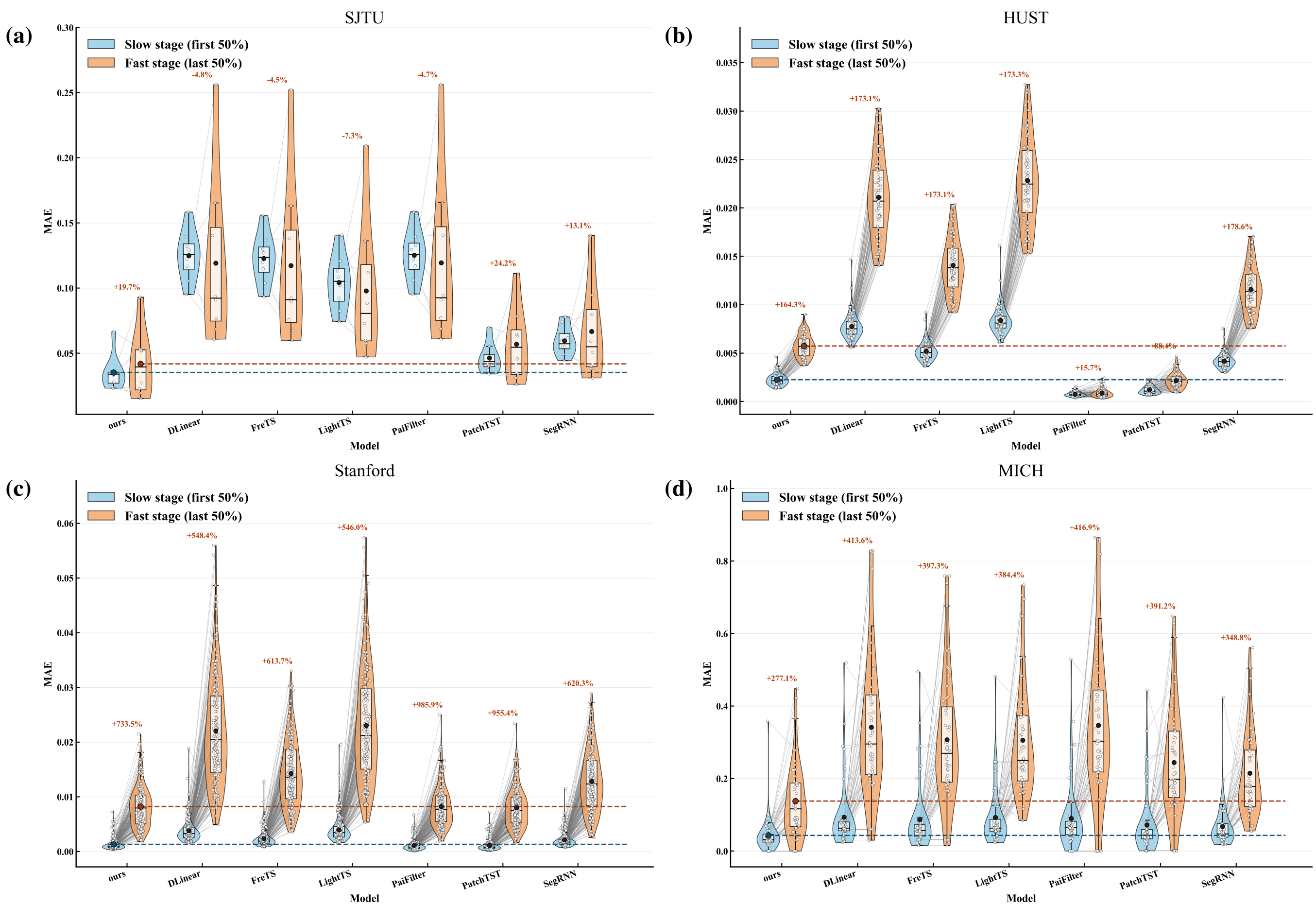

FIG. S8: **Stage-wise evaluation of capacity degradation forecasting performance on datasets exhibiting two-stage degradation behavior.** Per-cell forecasting-error distributions are reported separately for the slow-degradation and accelerated-degradation stages under the 96-cycle context and 96-cycle forecasting setting. **(a)** SJTU. **(b)** HUST. **(c)** Stanford. **(d)** MICH. The accelerated-degradation stage produces larger forecasting errors than the slow-degradation stage for most evaluated methods, reflecting the greater difficulty of predicting rapid and nonlinear capacity fade. Nevertheless, the proposed unified model maintains strong performance in both stages, with a particularly clear advantage on the SJTU dataset and competitive results on HUST, Stanford, and MICH.

As shown in Fig. S8, all evaluated methods generally produce substantially larger forecasting errors in the accelerated-degradation stage than in the slow-degradation stage. This result confirms that rapid capacity fade is more difficult to predict because of its stronger nonlinearity and more abrupt temporal variation. Nevertheless, the proposed unified capacity-forecasting

model maintains strong performance in both degradation stages. In particular, it clearly outperforms the comparison methods in both stages on the SJTU dataset, while achieving best or near-best performance on the HUST, Stanford, and MICH datasets.

These results demonstrate that the proposed method does not achieve favorable overall accuracy merely by fitting the relatively smooth early-life degradation trajectories. It also retains reliable forecasting capability in the more challenging accelerated-degradation stage, thereby providing direct evidence of its ability to predict capacity evolution across different phases of battery aging.

Supplementary Note 13:Ablation study of the physics-guided embedding

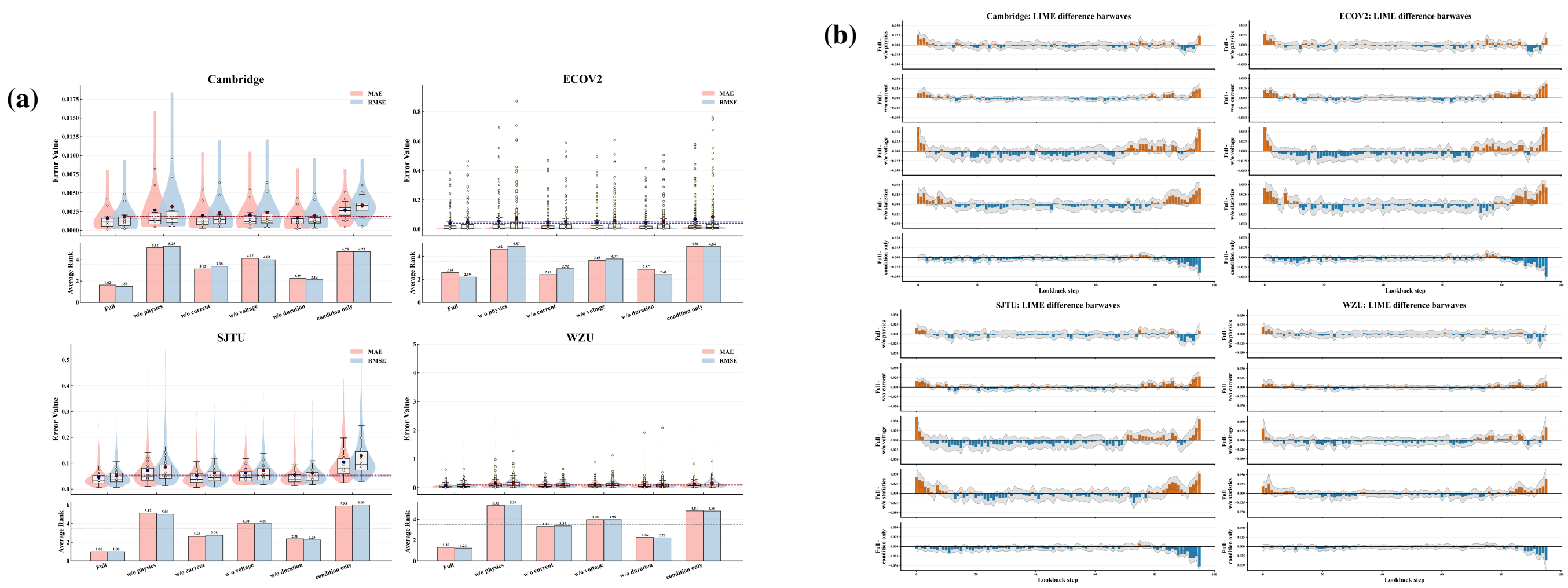

FIG. S9: **Ablation and LIME-based interpretation of the physics-guided embedding.** **(a)** Forecasting performance of the complete model and the ablated variants on the four unknown-condition datasets SJTU, Cambridge, EOCV2, and WZU. The complete physics-guided model achieves the best overall performance. Removing discharge duration causes only a minor change, whereas removing voltage descriptors results in a more pronounced deterioration. The PF-only variant, which excludes the endogenous capacity sequence, produces the largest forecasting errors. **(b)** Paired full-minus-ablation LIME differences over the 96-cycle look-back window. For each dataset, identical validation windows are explained using the complete model and each ablated variant. Each row represents one paired comparison, Full $-\,a$. Bars show the mean LIME coefficient difference across sampled windows, and shaded bands show the 25th–75th percentile range. Positive values indicate that the complete model assigns greater local importance to the corresponding historical capacity position, whereas negative values indicate greater importance under the ablated model. The alternating signs across the look-back window show that the physics-guided embedding redistributes, rather than uniformly amplifies, the temporal evidence used by the forecaster.

The physics-guided embedding is introduced as an auxiliary training component to incorporate cycle-level operating information into the representation learning process. Although the capacity history remains the primary input for forecasting, the physical descriptors extracted from the discharge process may help the model distinguish degradation patterns generated under different operating conditions. We therefore conduct an ablation study to quantify the contribution of the complete physics-guided embedding and its individual feature groups. In addition to comparing forecasting errors, we use Local Interpretable Model-Agnostic Explanations (LIME) to examine how the physics-guided component changes the temporal evidence used by the forecaster.

Ablation settings. The complete model uses the endogenous capacity sequence together with ten physical features during training. The physical features comprise five current-based descriptors, four voltage-based descriptors, and discharge duration. We compare the complete model with the following five ablated variants:

- **No physics**: All physical features and the associated physics-guided embedding branch are removed, leaving only the capacity-forecasting objective.
- **No current**: All current-based descriptors are removed, including current mean, standard deviation, skewness, kurtosis, and root-mean-square value.
- **No voltage**: All voltage-based descriptors are removed, including voltage mean, standard deviation, skewness, and kurtosis.
- **No duration**: The discharge-duration descriptor is removed, while the current- and voltage-based descriptors are retained.
- **PF only**: The endogenous capacity sequence is removed, and forecasting is performed using only the exogenous physical-feature sequence.

All variants are evaluated using the same training protocol, data partitions, 96-cycle historical context, and 96-cycle forecasting horizon as the complete model. The evaluation is conducted on the four unknown-condition datasets SJTU, Cambridge, EOCV2, and WZU.

Forecasting performance. Figure S9(a) compares the forecasting performance of the complete model and the five ablated variants. The complete physics-guided model achieves the best overall performance across the evaluated datasets, demonstrating that the joint use of capacity history and cycle-level physical descriptors contributes to cross-dataset forecasting accuracy.

Among the ablated variants, **No duration** produces results closest to those of the complete model, indicating that discharge duration provides only a limited incremental contribution under the current feature design. One possible explanation is that discharge duration is strongly and directly correlated with discharge capacity under many of the cycling protocols considered here. Consequently, it may contain less complementary information than descriptors characterizing the statistical behavior of voltage and current.

Removing the voltage-based descriptors causes a more pronounced deterioration in forecasting performance. This result indicates that cycle-level voltage statistics contain particularly useful information about operating-condition-dependent degradation. Voltage responses reflect changes in polarization, operating range, and electrochemical state during discharge and therefore provide complementary information that cannot be fully rEOCVered from the capacity trajectory alone. The effect of removing the current descriptors is comparatively smaller, suggesting that current statistics are informative but less discriminative than voltage descriptors within the present heterogeneous corpus.

The **No physics** variant also performs worse than the complete model, confirming that the observed improvement is not solely attributable to the Timer-based forecasting backbone. Instead, the auxiliary physical information contributes to learning representations that are more transferable across previously unseen battery chemistries and operating conditions.

The **PF-only** variant yields the weakest overall performance. This result is expected because the physical descriptors characterize the operating behavior of individual cycles but do not directly preserve the cumulative degradation state, long-term trajectory continuity, or historical capacity trend of the cell. Physical variables are therefore better suited as auxiliary conditioning signals than as substitutes for the endogenous capacity sequence. The result also confirms that the forecasting improvement of the complete model arises from the complementary use of endogenous and exogenous information rather than from the physical descriptors alone.

LIME-based analysis of temporal evidence. To further investigate how the physics-guided embedding affects the historical information used for forecasting, we perform a large-scale LIME analysis on validation windows from SJTU, Cambridge, EOCV2, and WZU. For each sampled window, the same historical capacity sequence is explained under the complete model and the corresponding ablated variant. The explained output is the mean predicted capacity over the entire forecasting horizon.

Let $\phi_{m,j}^{(s)}$ denote the LIME coefficient assigned by model m to look-back position j in sampled window s. The coefficient quantifies the local contribution of the capacity value at that historical position to the horizon-level forecast.

For dataset D and ablation variant a, the paired explanation difference is defined as

$$\Delta_{a,j}^{(s)} = \phi_{\mathrm{Full},j}^{(s)} - \phi_{a,j}^{(s)}. \tag{S99}$$

The average explanation difference over all sampled windows in dataset D is

$$\overline{\Delta}_{a,j}^{(D)} = \frac{1}{|\Omega_D|} \sum_{s \in \Omega_D} \Delta_{a,j}^{(s)}, \tag{S100}$$

where Ω_D denotes the set of sampled validation windows from dataset D, and $|\Omega_D|$ denotes the number of sampled windows.

In Fig. S9(b), the bars show $\overline{\Delta}_{a,j}^{(D)}$, and the shaded bands represent the corresponding interquartile ranges. A positive value indicates that the complete model assigns greater local importance to a historical capacity position than the ablated model, whereas a negative value indicates greater local importance under the ablated variant.

The results show that the physics-guided embedding does not uniformly increase the importance of all historical positions. Instead, it redistributes local sensitivity across the 96-cycle look-back window. Among the directly matched feature-removal variants, removing voltage descriptors produces the largest mean absolute LIME difference, 9.09×10^{-3}, whereas the corresponding values for removing current descriptors and removing the complete physics branch are 4.31×10^{-3} and 4.12×10^{-3}, respectively. This observation is consistent with the forecasting-error results and further indicates that voltage descriptors exert the strongest influence on how the model allocates temporal evidence.

At the dataset level, EOCV2 and SJTU exhibit the largest average absolute explanation differences, 7.26×10^{-3} and 7.13×10^{-3}, respectively, followed by Cambridge at 6.25×10^{-3} and WZU at 4.36×10^{-3}. The analysis includes 72 paired windows for EOCV2 and WZU and 48 paired windows for Cambridge and SJTU, indicating that these patterns represent dataset-level behavior rather than isolated examples.

Only approximately 31%–36% of the look-back positions exhibit positive mean differences. The mixed signs across the historical window confirm that the complete model does not simply amplify the influence of every capacity observation. Instead,

the physics-guided objective changes the relative contribution of different temporal positions by increasing the importance of some historical observations while suppressing others.

This result suggests that the physical descriptors act as auxiliary representation constraints during training. They help the model associate operating-condition information with the capacity trajectory and thereby reorganize the temporal evidence used for forecasting. The LIME analysis should not be interpreted as a direct visualization of Transformer attention; rather, it provides local input–output sensitivity evidence that the physics-guided embedding changes how the trained forecaster uses historical capacity observations.

Summary. Overall, the ablation results demonstrate that the complete physics-guided embedding provides a measurable improvement over capacity-only forecasting. Voltage descriptors contribute most strongly among the investigated physical-feature groups, whereas discharge duration provides relatively limited additional information. The poor performance of the PF-only variant further confirms that physical descriptors cannot replace the endogenous capacity trajectory but can effectively complement it during representation learning. Together, the forecasting metrics and LIME analysis indicate that the proposed embedding improves generalization by reshaping the model's use of temporal capacity evidence rather than by uniformly increasing sensitivity across the look-back window.

[1] Z. Tan, Y. Tian, and J. Li, Glime: General, stable and local lime explanation, Advances in Neural Information Processing Systems **36**, 36250 (2023).
[2] E. J. Hu, Y. Shen, P. Wallis, Z. Allen-Zhu, Y. Li, S. Wang, L. Wang, W. Chen, *et al.*, Lora: Low-rank adaptation of large language models., ICLR **1**, 3 (2022).
