# Supplementary material for: Universal Battery Capacity Degradation Forecasting Driven by Foundation Models Across Diverse Chemistries and Conditions
Source: Adv Sci (Weinh). 2026 Aug 24:e77261. Online ahead of print. doi: 10.1002/advs.77261 (PMC13502271; doi:10.1002/advs.77261)
Supplement: Supplementary file 2 [file ADVS-9999-e77261-s003.pdf]

All Experiment result

tri˙horizon˙shared˙axes

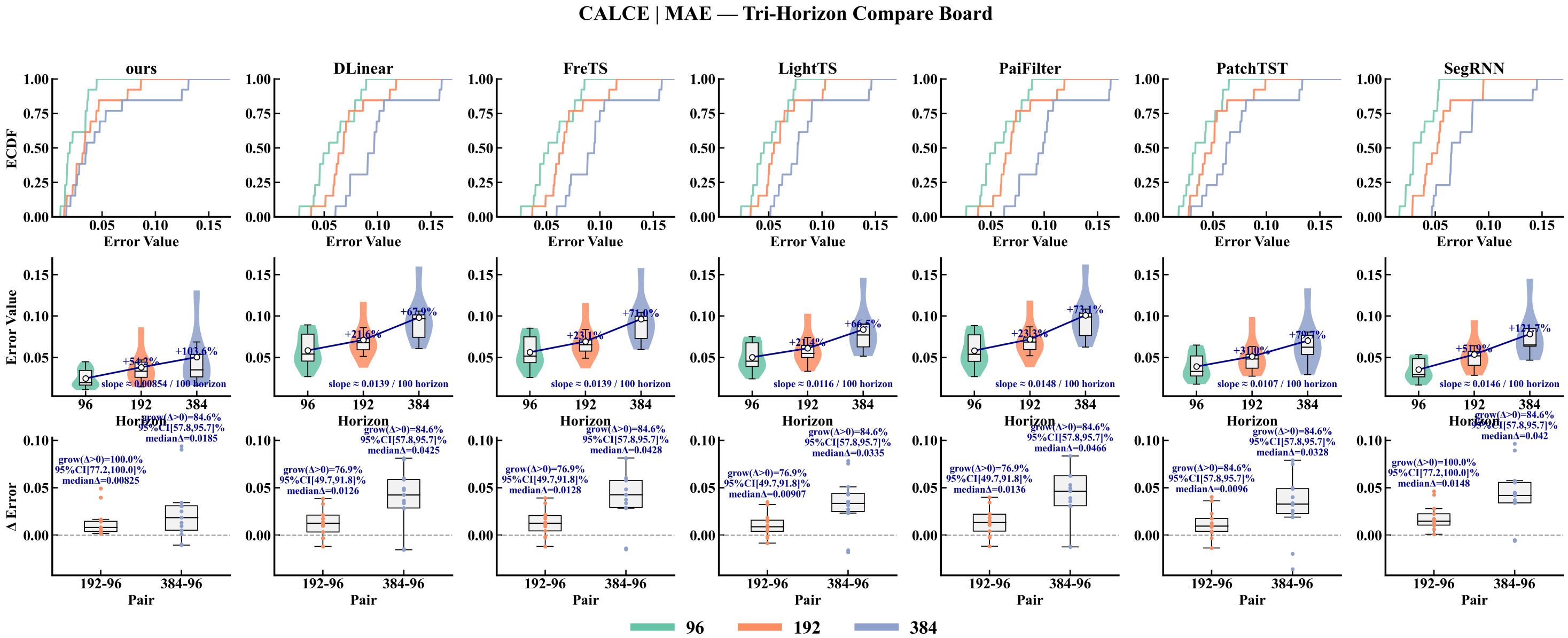

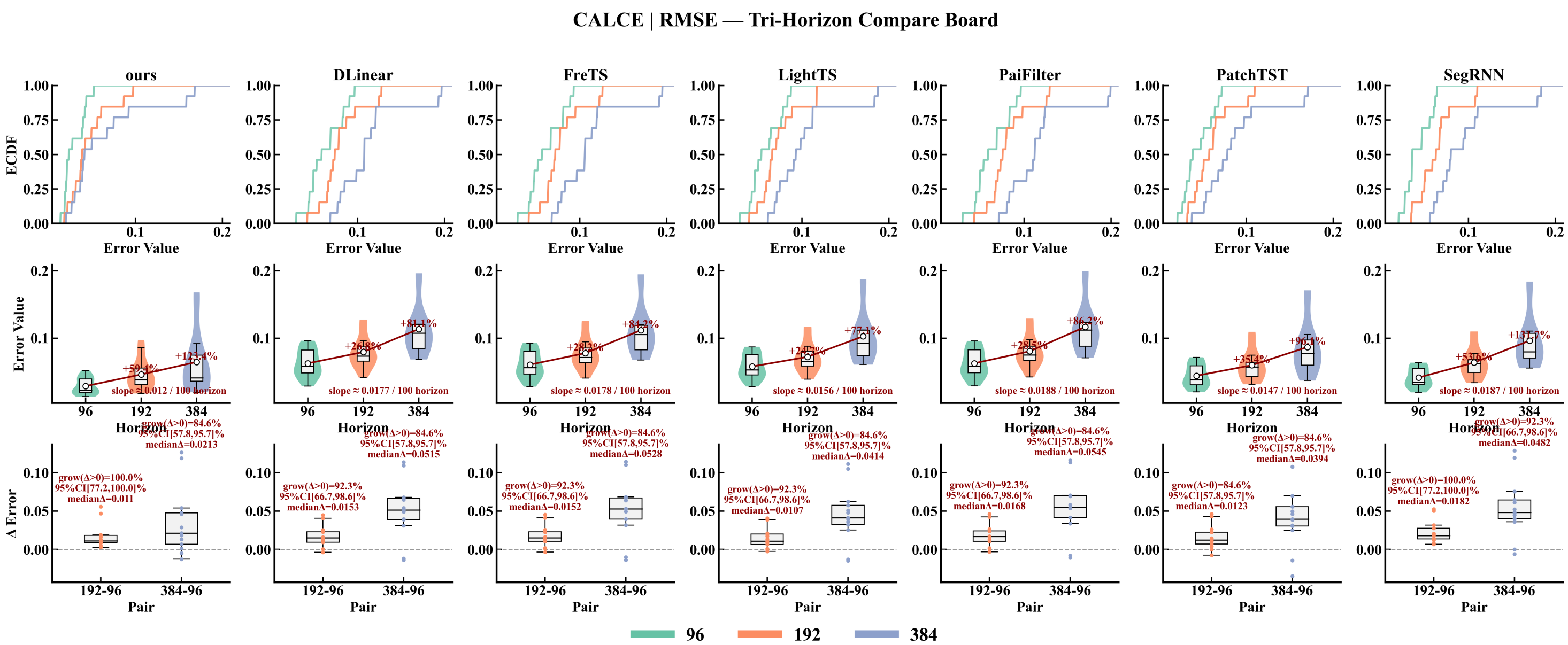

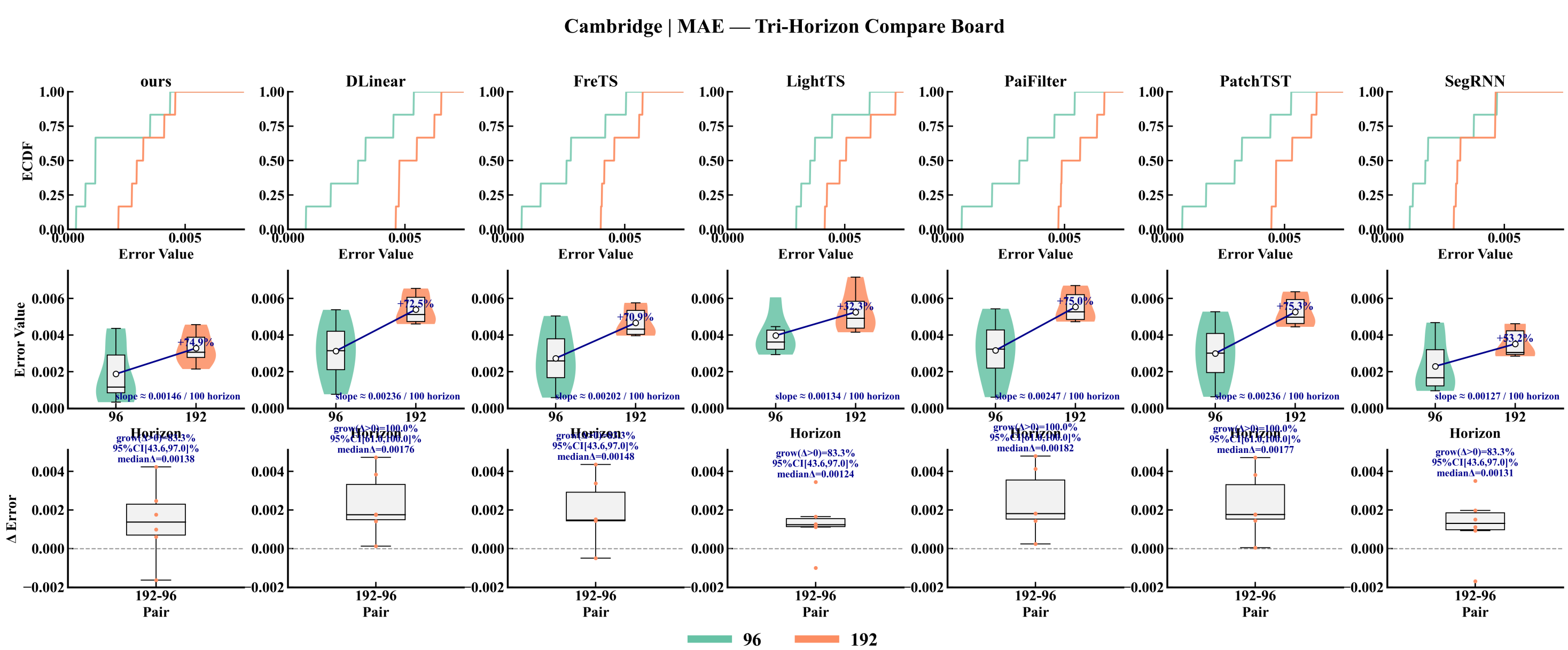

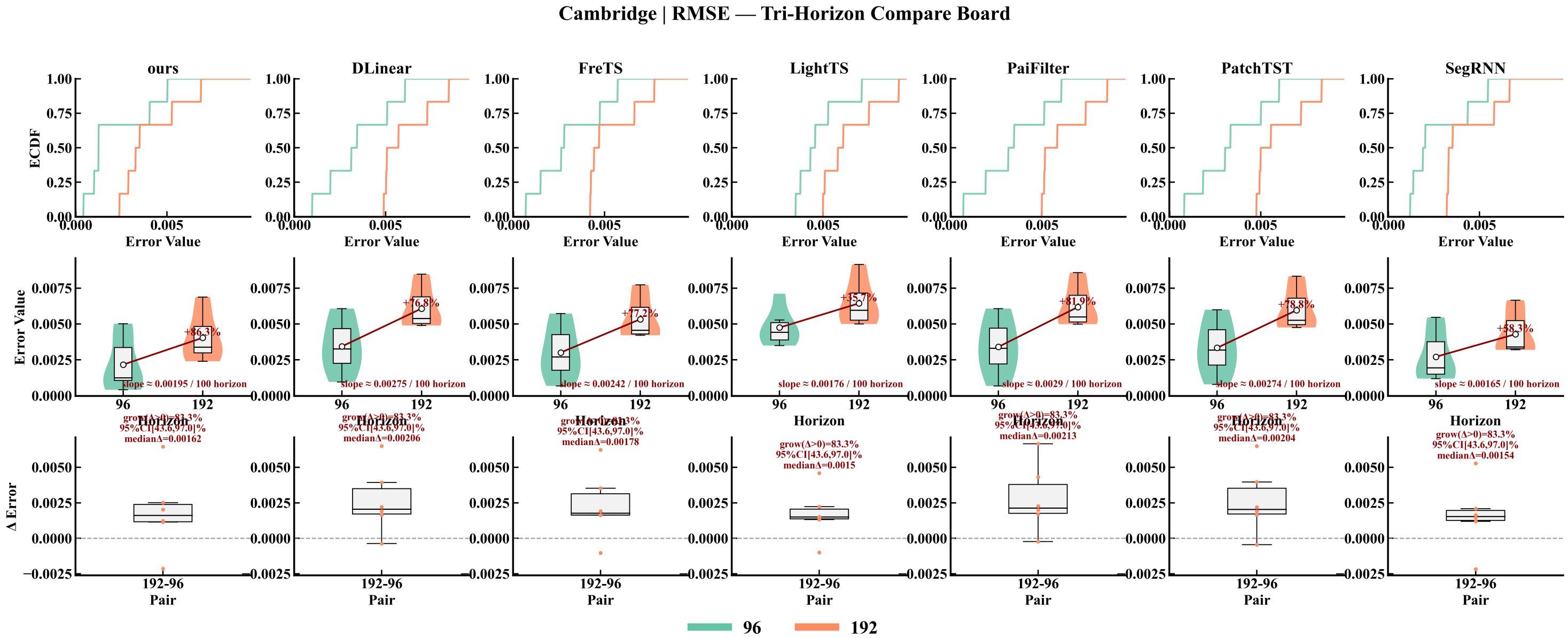
Cambridge | RMSE — Tri-Horizon Compare Board
ours
DLinear
FreTS
LightTS
PaiFilter
PatchTST
SegRNN
ECDF
Error Value
Error Value
Horizon
Δ Error
Pair
192-96
slope ≈ 0.00195 / 100 horizon
slope ≈ 0.00275 / 100 horizon
slope ≈ 0.00242 / 100 horizon
slope ≈ 0.00176 / 100 horizon
slope ≈ 0.0029 / 100 horizon
slope ≈ 0.00274 / 100 horizon
slope ≈ 0.00165 / 100 horizon
96
192

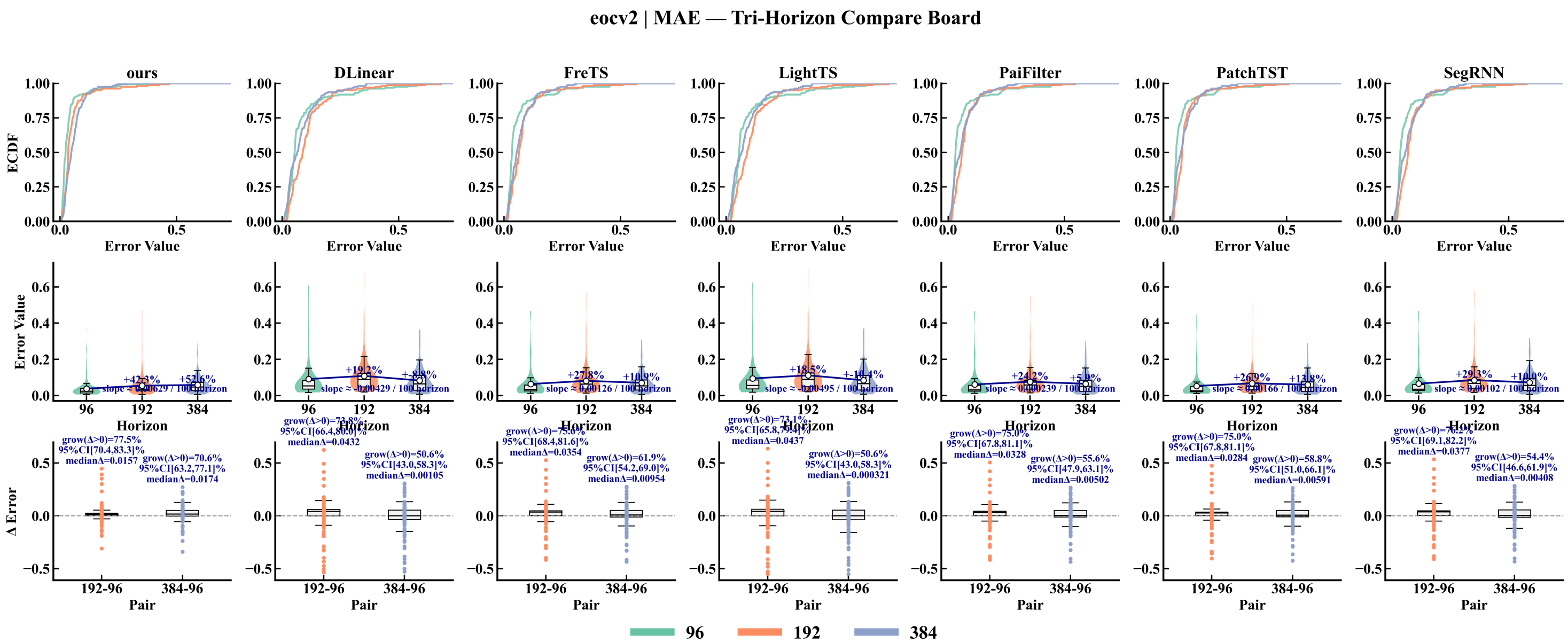
eocv2 | MAE — Tri-Horizon Compare Board
ours
DLinear
FreTS
LightTS
PaiFilter
PatchTST
SegRNN
ECDF
Error Value
Error Value
Horizon
Δ Error
Pair
192-96
384-96
96
192
384

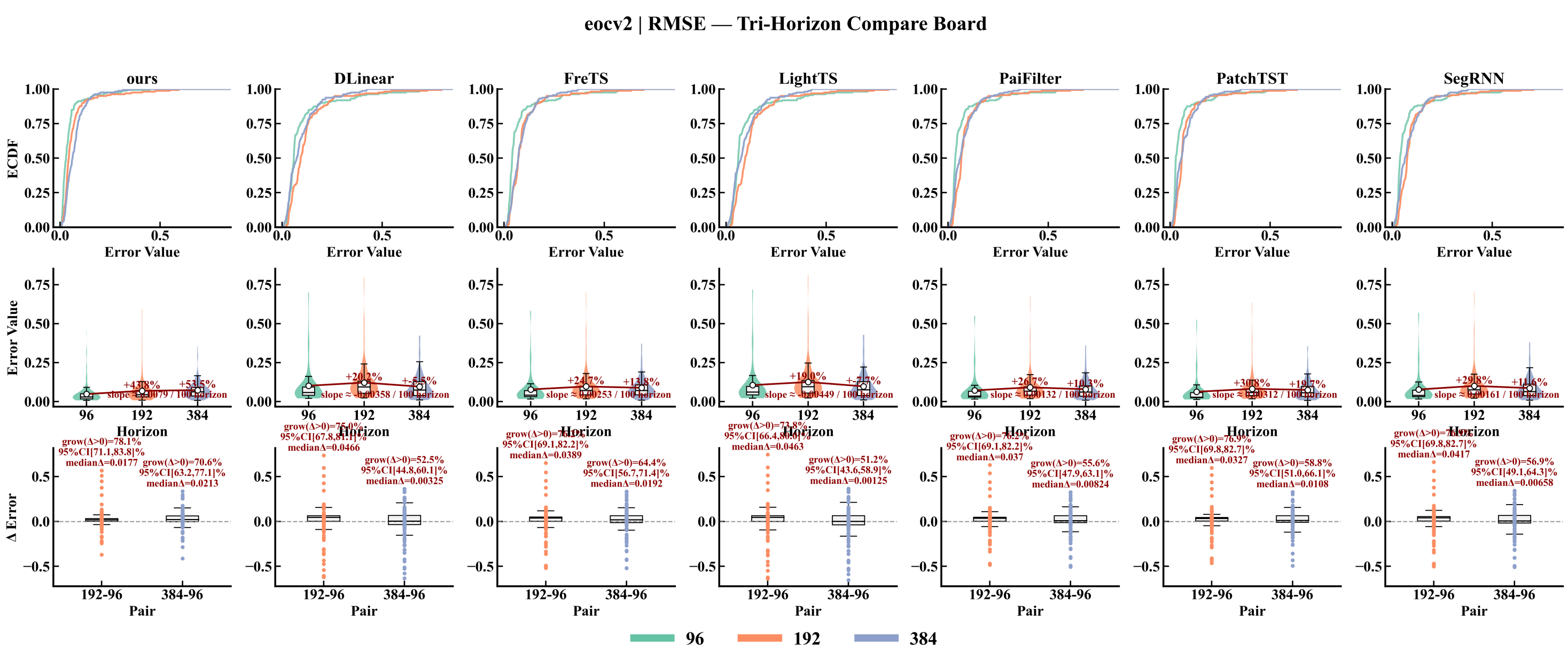
eocv2 | RMSE — Tri-Horizon Compare Board
ours
DLinear
FreTS
LightTS
PaiFilter
PatchTST
SegRNN
ECDF
Error Value
Error Value
Horizon
Δ Error
Pair
192-96
384-96
96
192
384

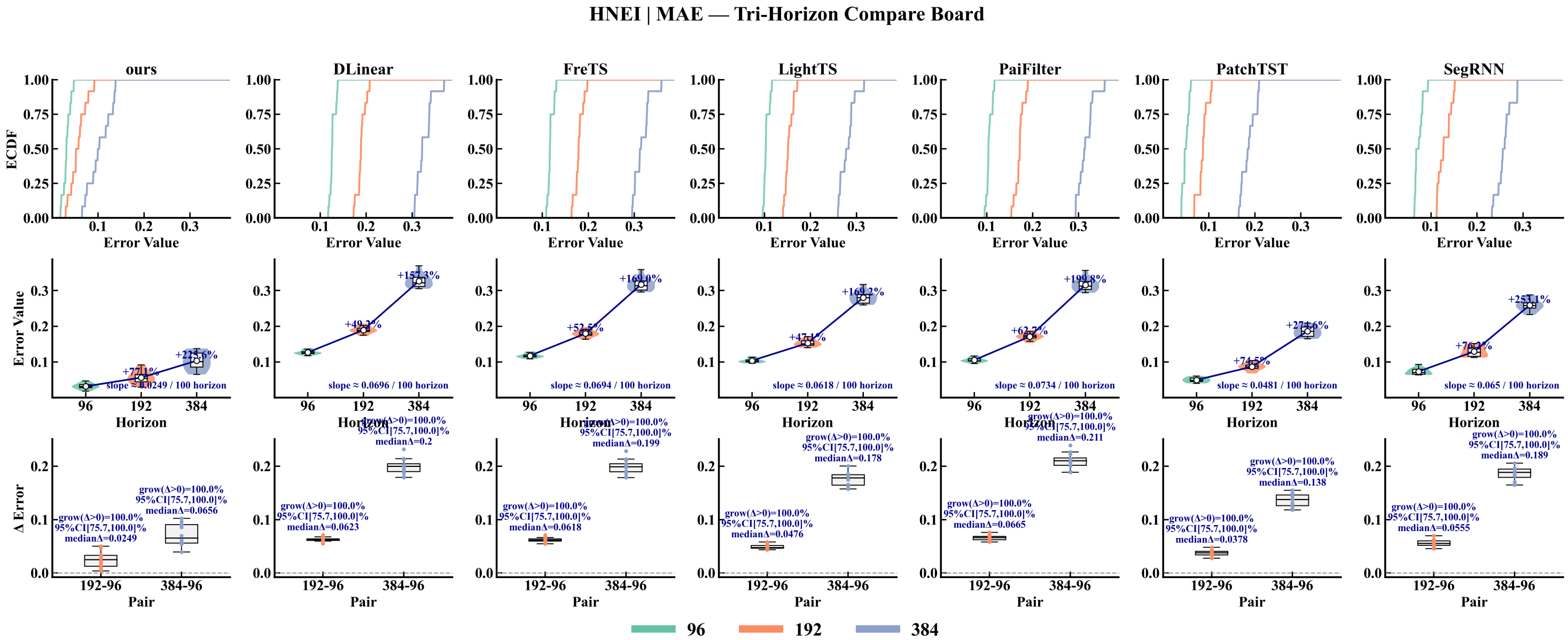
HNEI | MAE — Tri-Horizon Compare Board
ours
DLinear
FreTS
LightTS
PaiFilter
PatchTST
SegRNN
ECDF
Error Value
Horizon
Δ Error
Pair
96
192
384

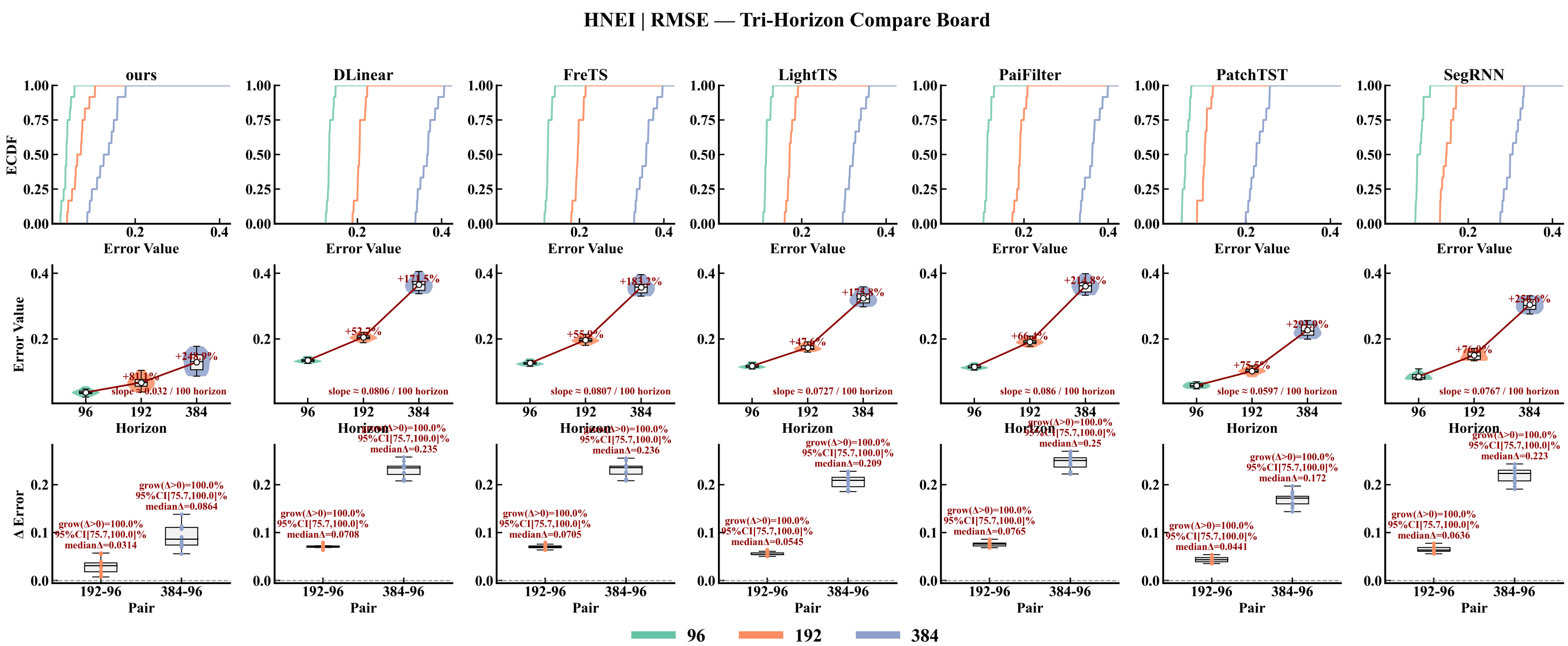
HNEI | RMSE — Tri-Horizon Compare Board
ours
DLinear
FreTS
LightTS
PaiFilter
PatchTST
SegRNN
ECDF
Error Value
Horizon
Δ Error
Pair
96
192
384

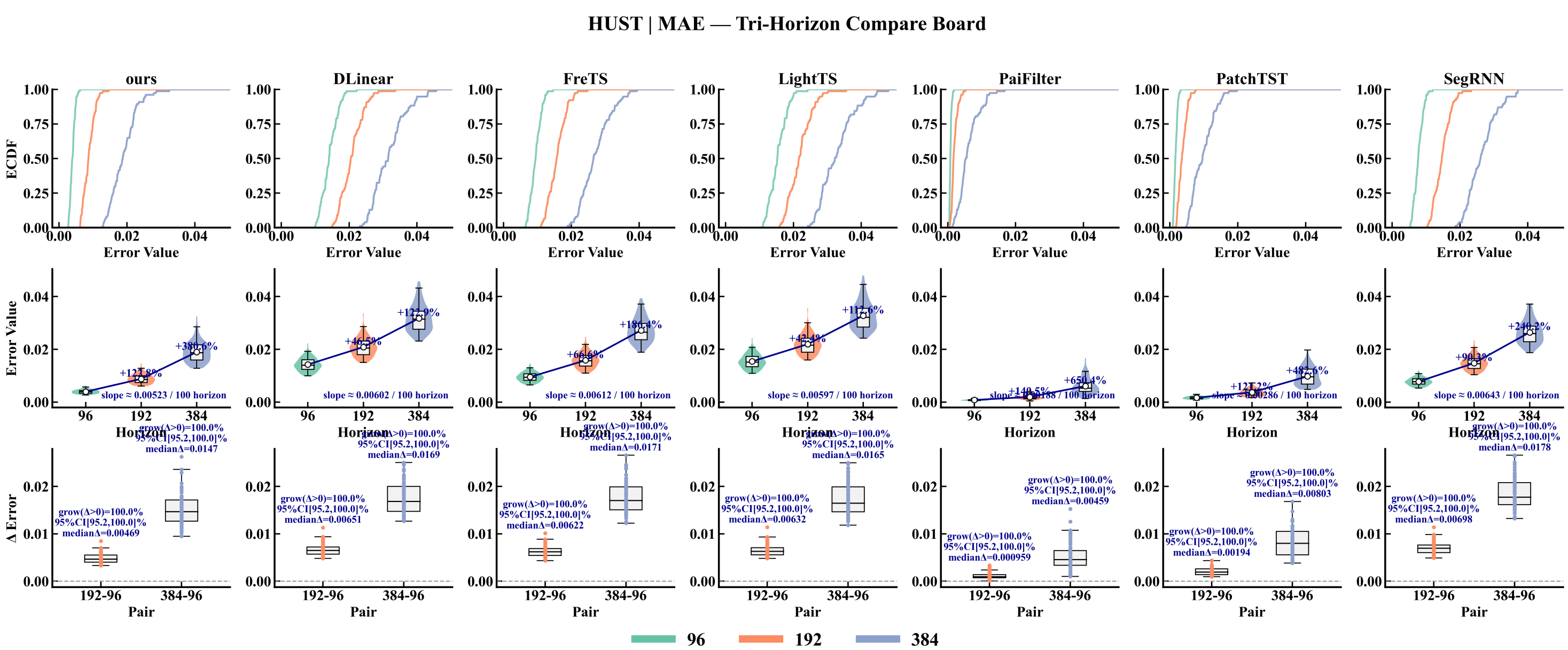
HUST | MAE — Tri-Horizon Compare Board
ours
DLinear
FreTS
LightTS
PaiFilter
PatchTST
SegRNN
ECDF
Error Value
Horizon
Δ Error
Pair
96
192
384

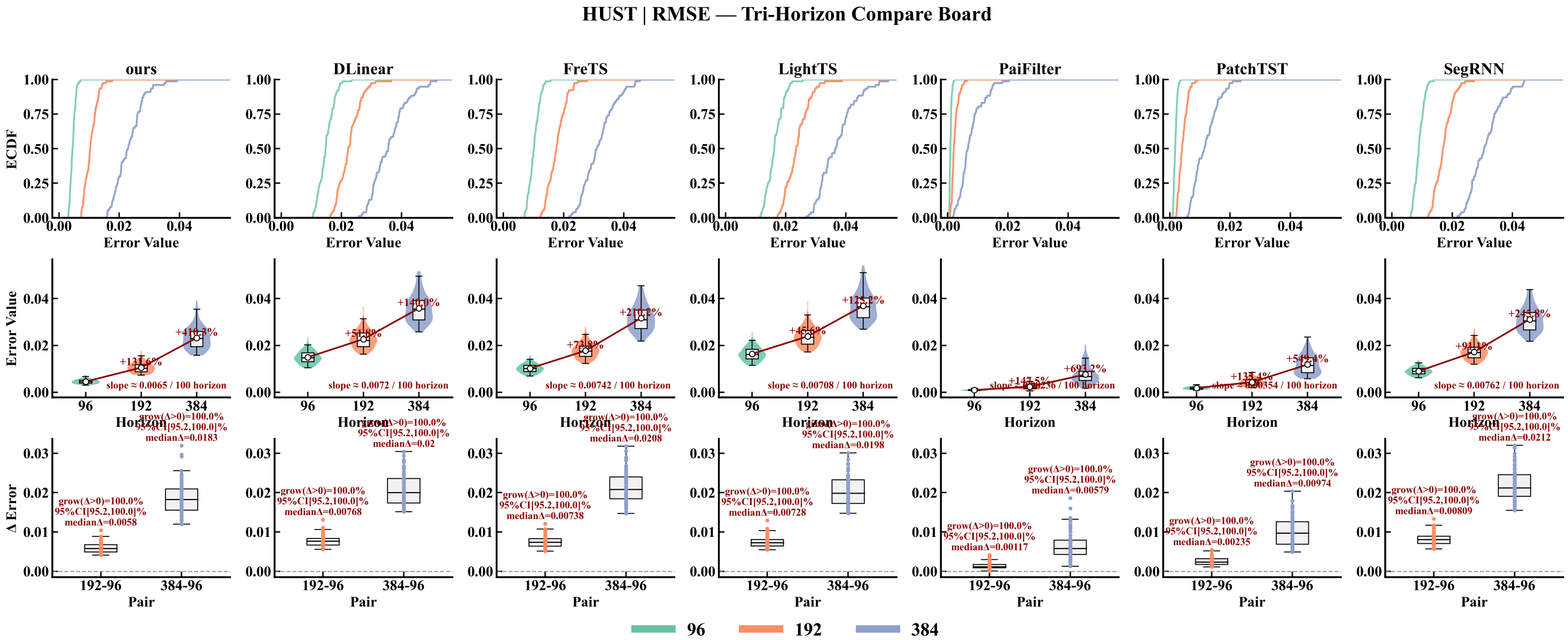
HUST | RMSE — Tri-Horizon Compare Board
ours
DLinear
FreTS
LightTS
PaiFilter
PatchTST
SegRNN
ECDF
Error Value
Horizon
Δ Error
Pair
192-96
384-96
96
192
384

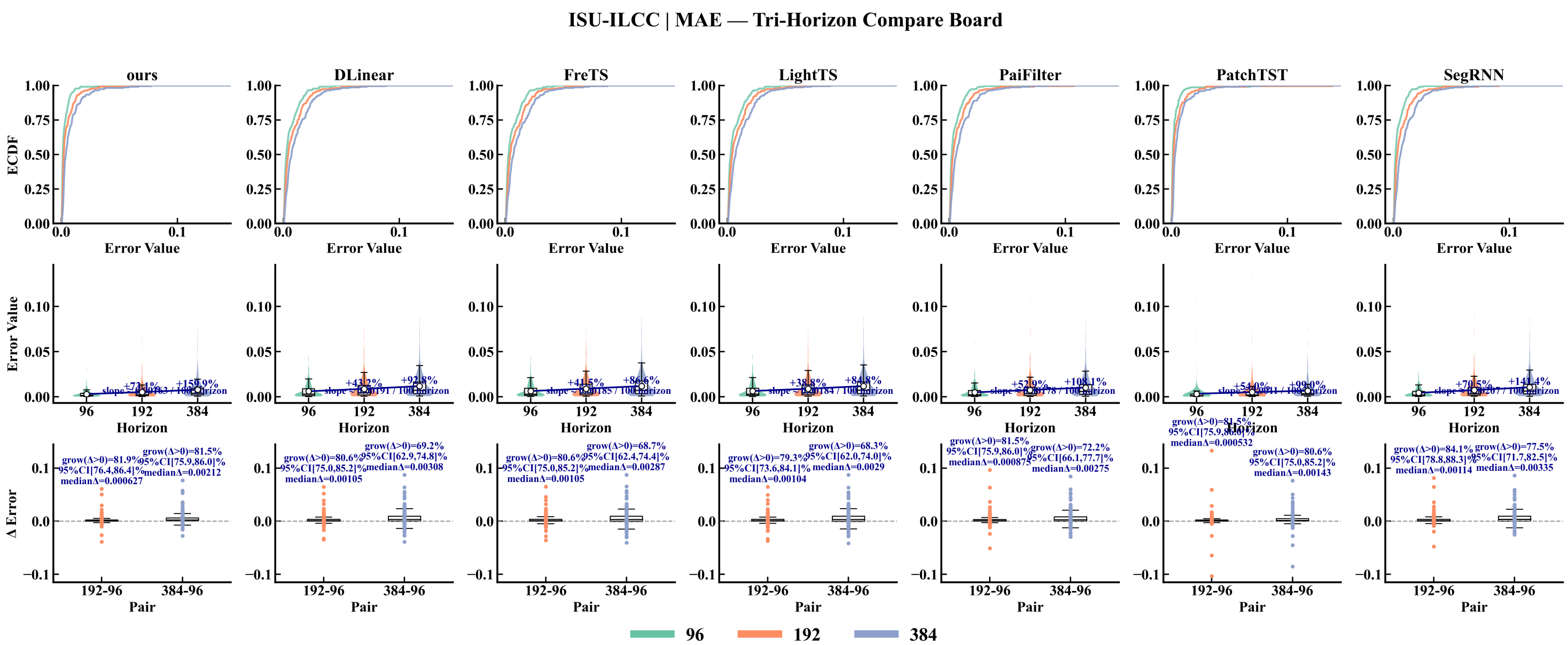
ISU-ILCC | MAE — Tri-Horizon Compare Board
ours
DLinear
FreTS
LightTS
PaiFilter
PatchTST
SegRNN
ECDF
Error Value
Horizon
Δ Error
Pair
192-96
384-96
96
192
384

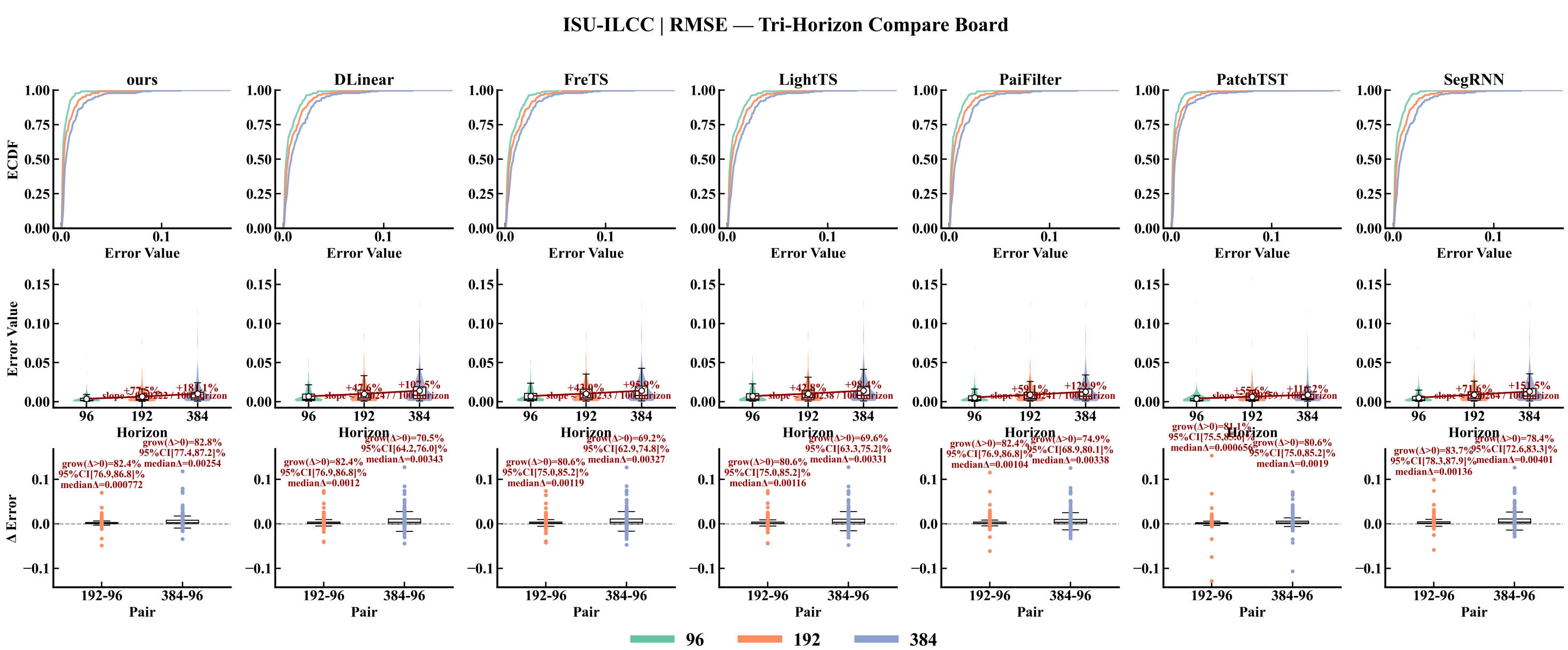
ISU-ILCC | RMSE — Tri-Horizon Compare Board
ours
DLinear
FreTS
LightTS
PaiFilter
PatchTST
SegRNN
ECDF
Error Value
Horizon
Δ Error
Pair
192-96
384-96
96
192
384

MATR | MAE — Tri-Horizon Compare Board

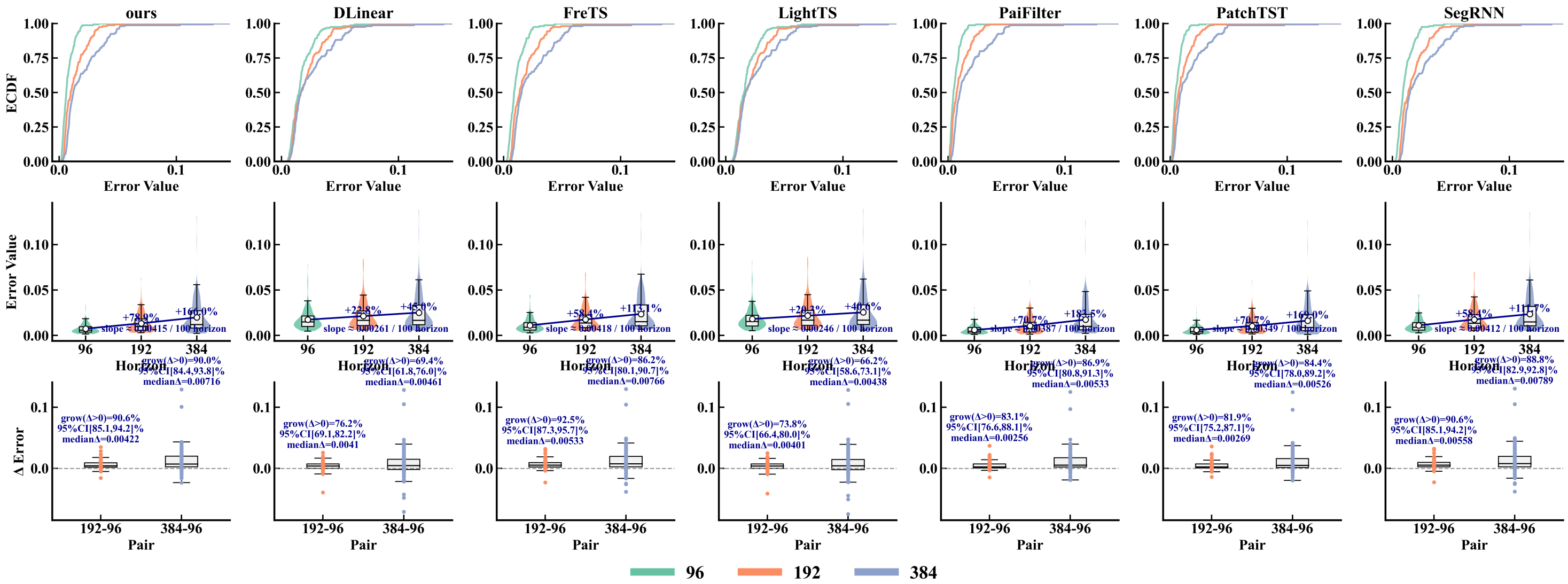

MATR | RMSE — Tri-Horizon Compare Board

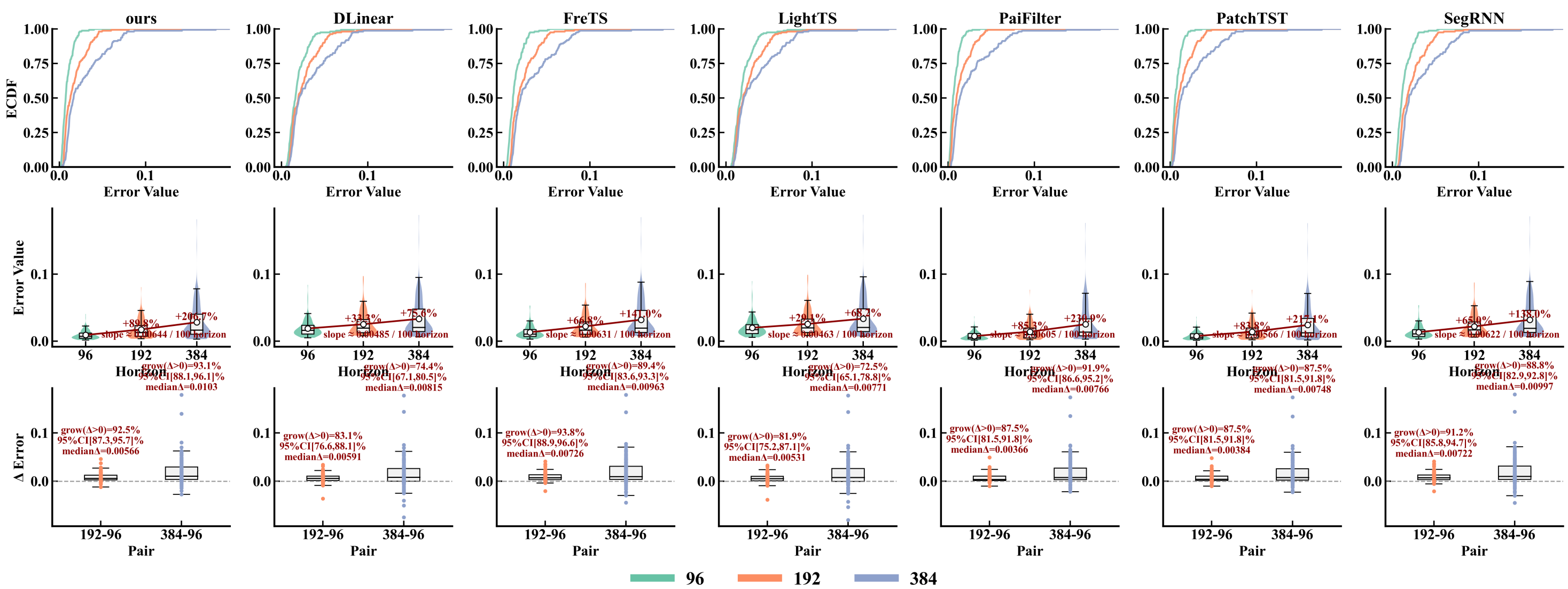

MICH | MAE — Tri-Horizon Compare Board

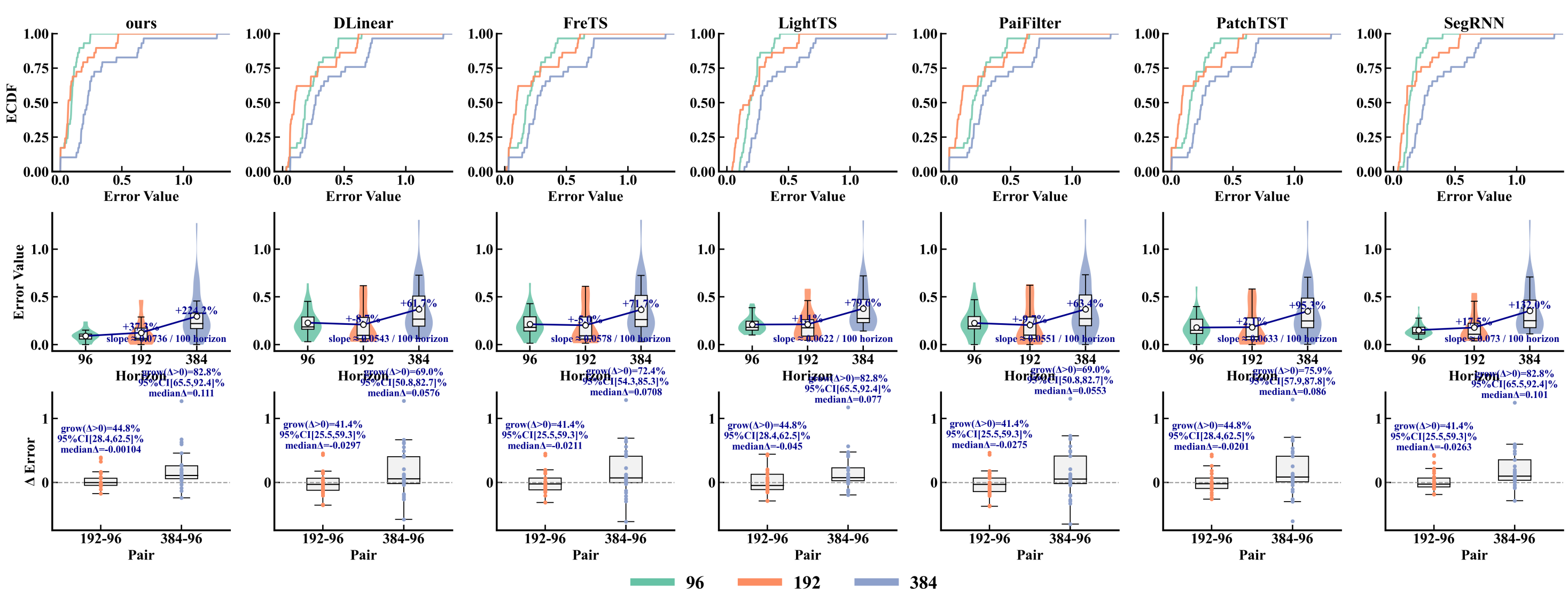

MICH | RMSE — Tri-Horizon Compare Board

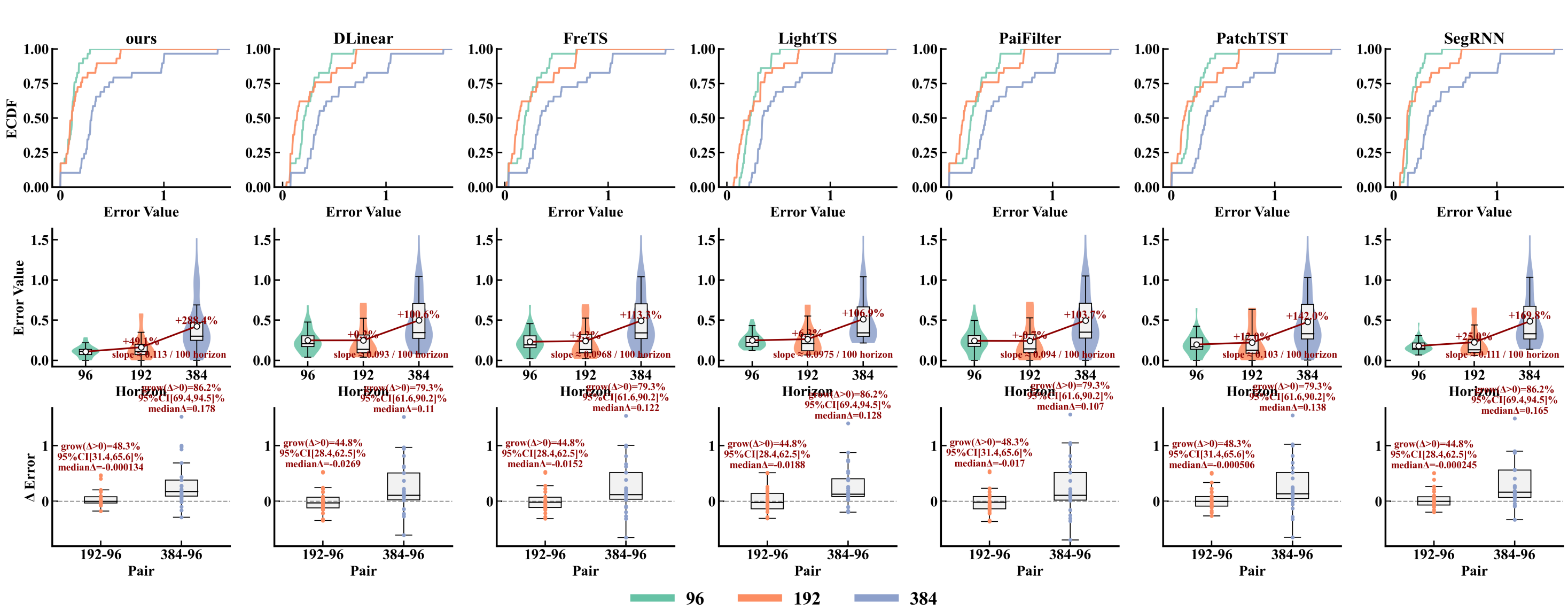

NA-ion | MAE — Tri-Horizon Compare Board

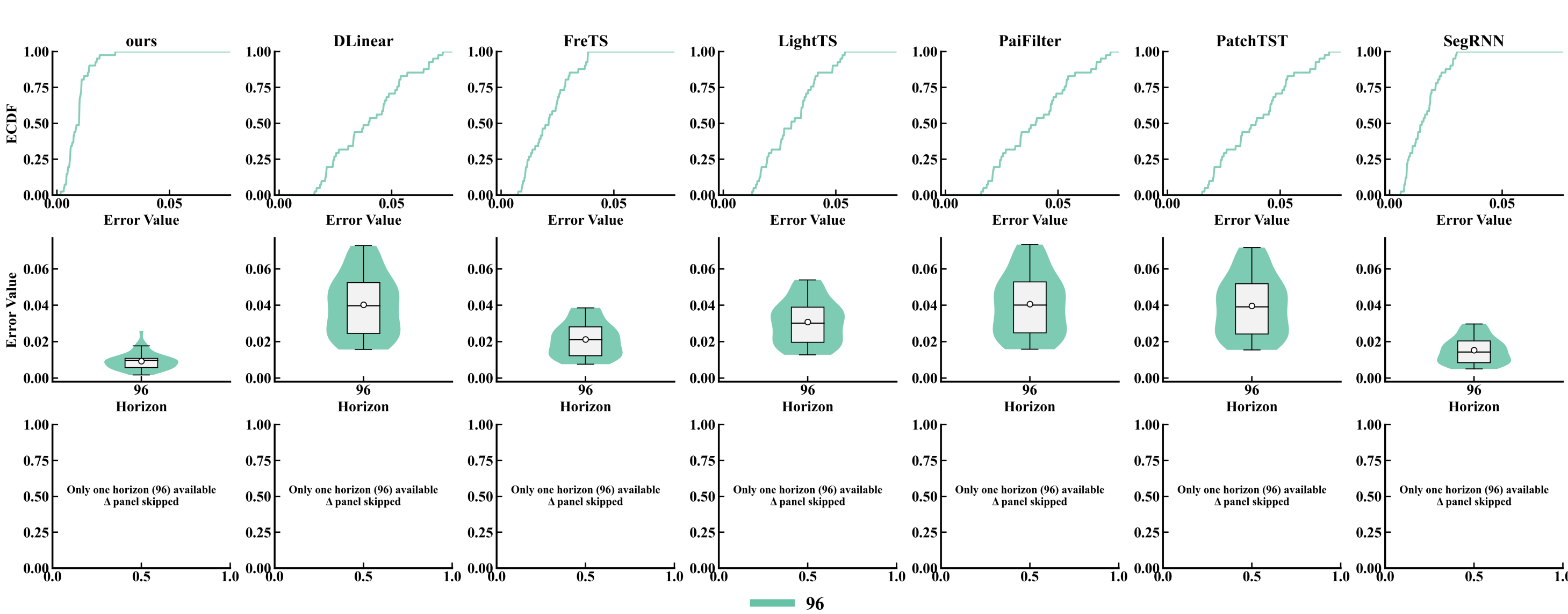

NA-ion | RMSE — Tri-Horizon Compare Board

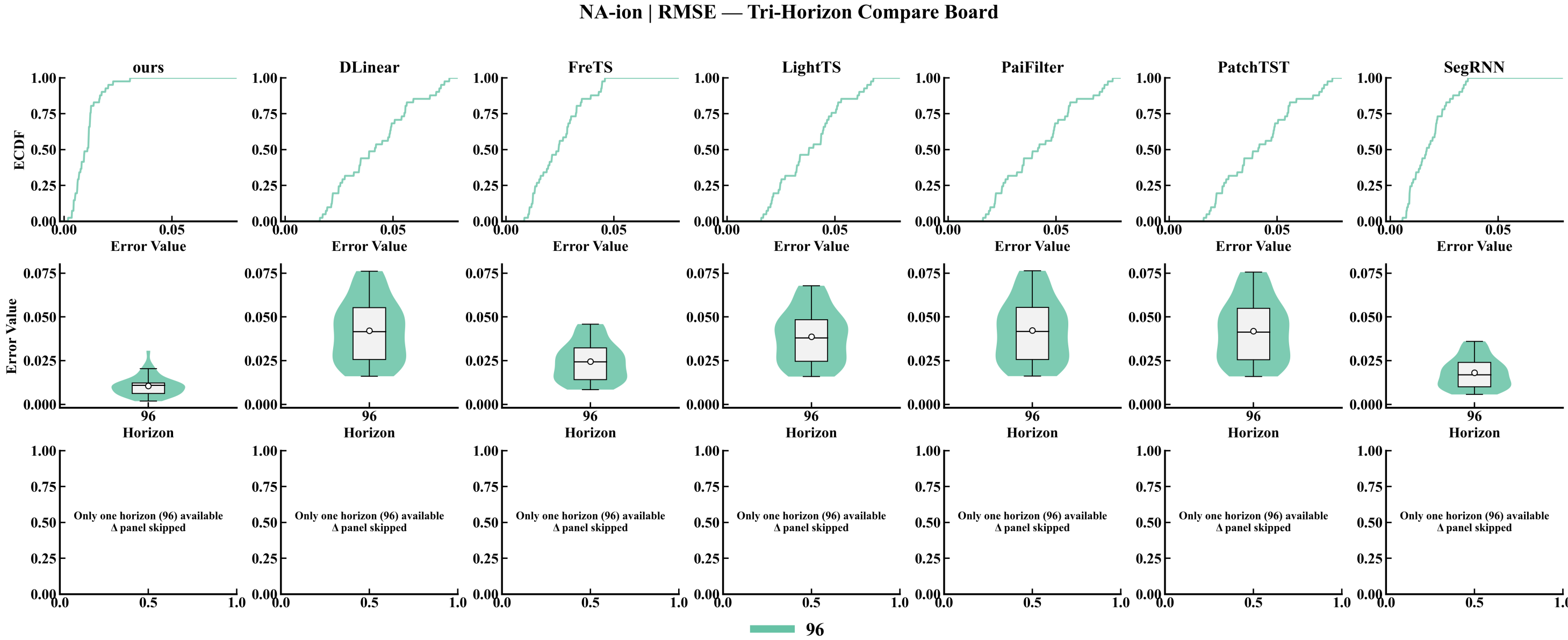

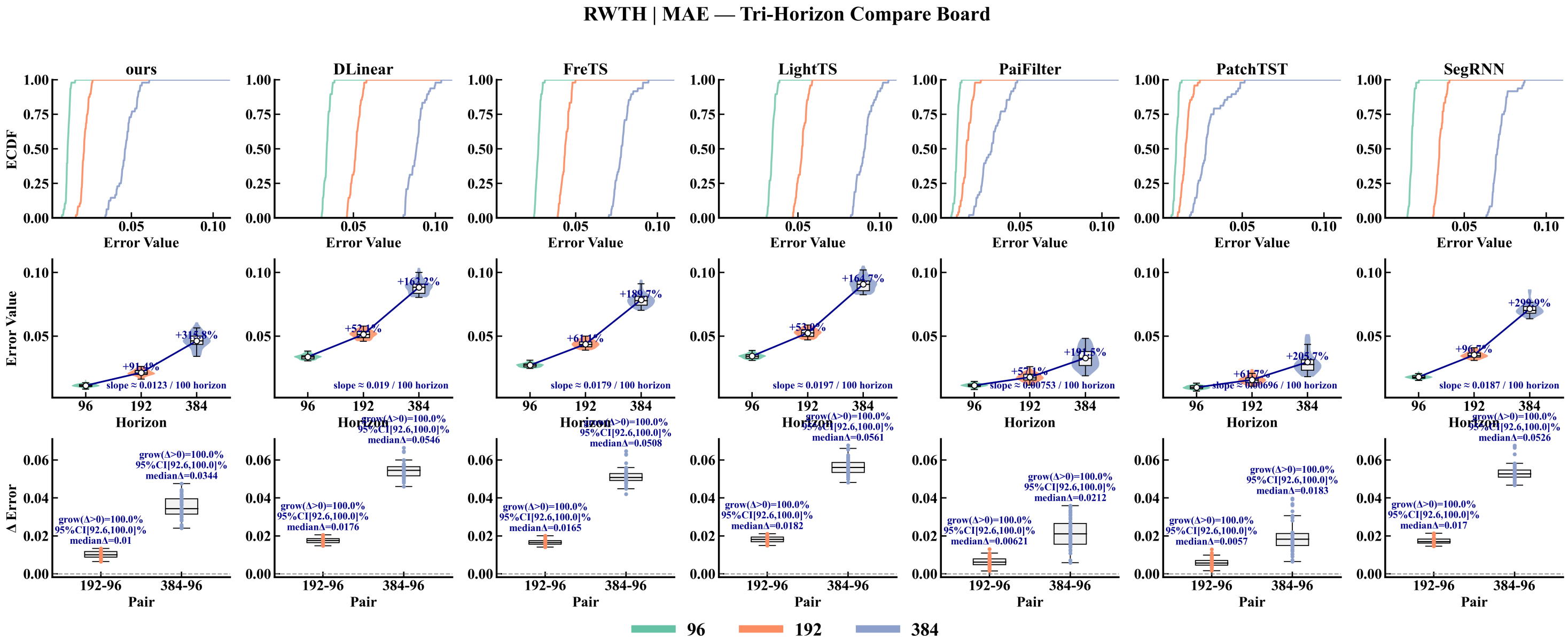
RWTH | MAE — Tri-Horizon Compare Board
ours
DLinear
FreTS
LightTS
PaiFilter
PatchTST
SegRNN
ECDF
Error Value
Horizon
Δ Error
Pair
96
192
384

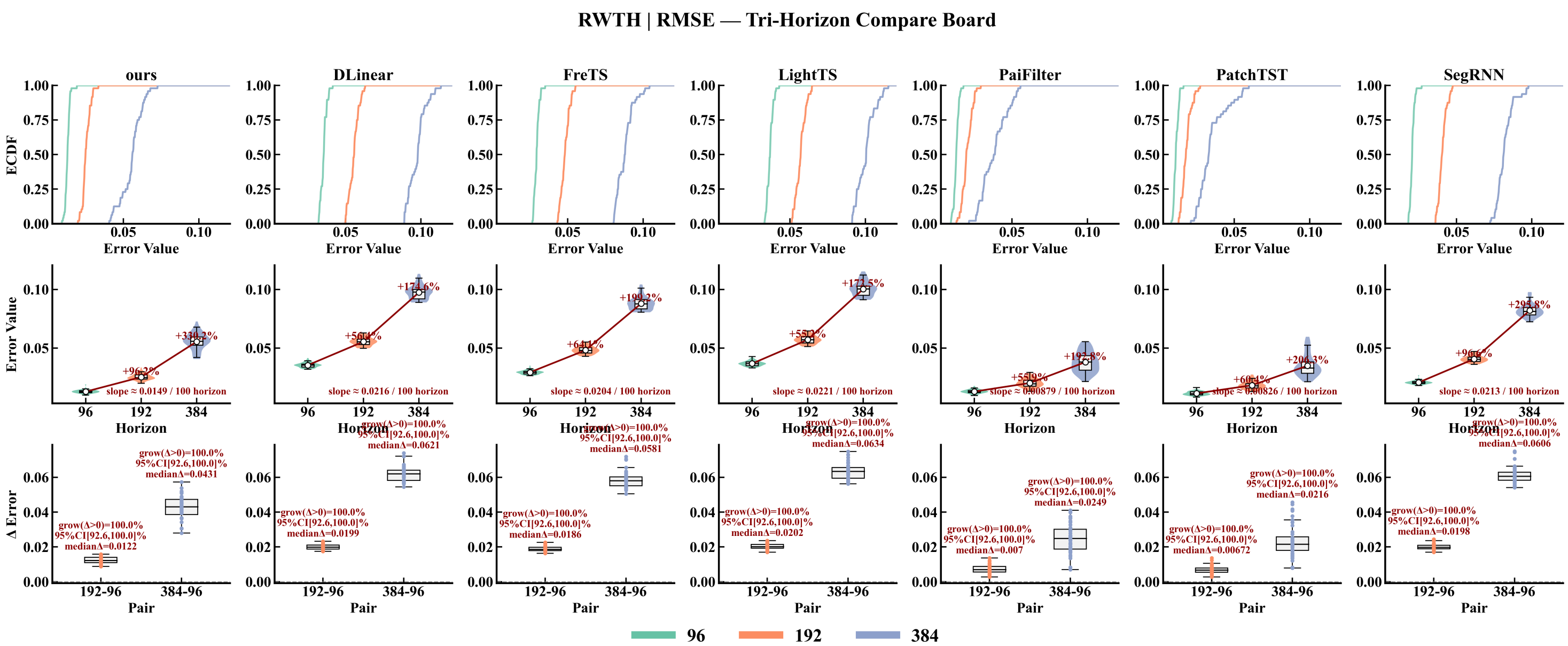
RWTH | RMSE — Tri-Horizon Compare Board
ours
DLinear
FreTS
LightTS
PaiFilter
PatchTST
SegRNN
ECDF
Error Value
Horizon
Δ Error
Pair
96
192
384

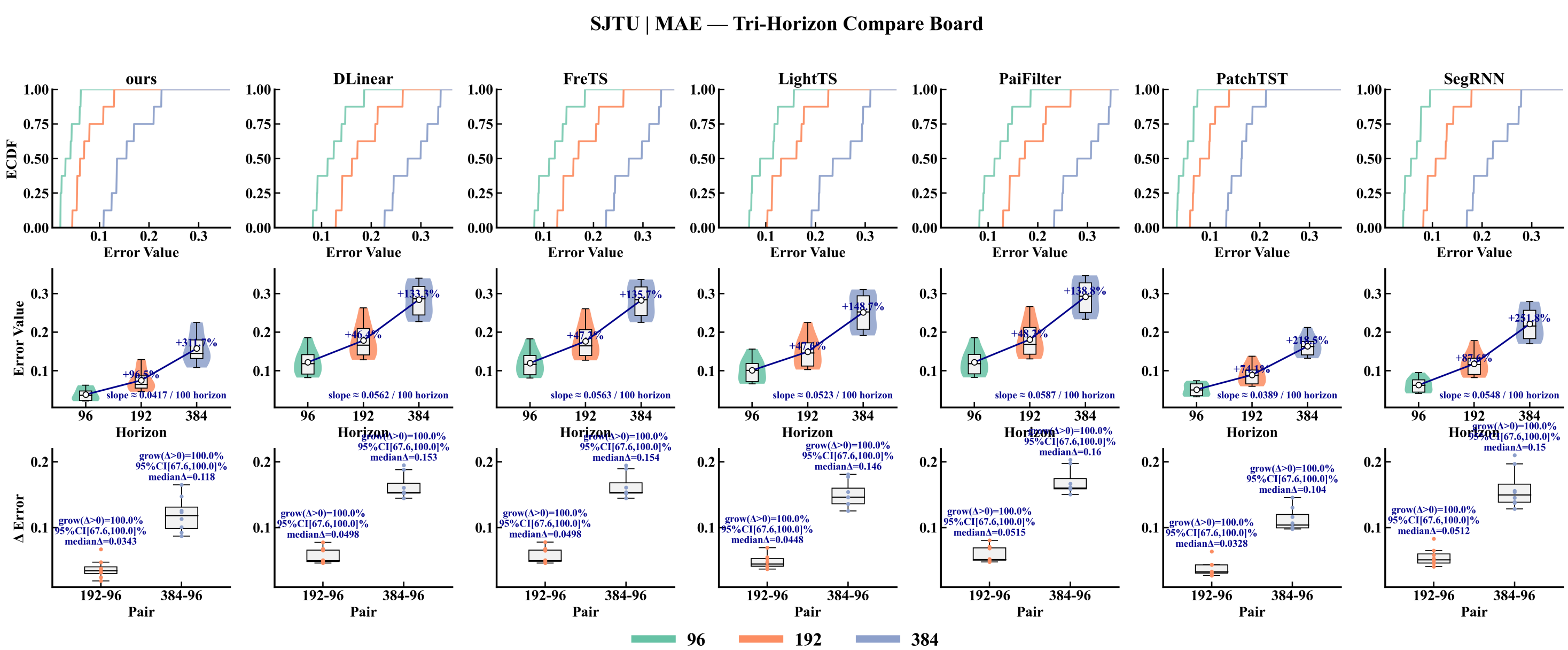
SJTU | MAE — Tri-Horizon Compare Board
ours
DLinear
FreTS
LightTS
PaiFilter
PatchTST
SegRNN
ECDF
Error Value
Horizon
Δ Error
Pair
96
192
384

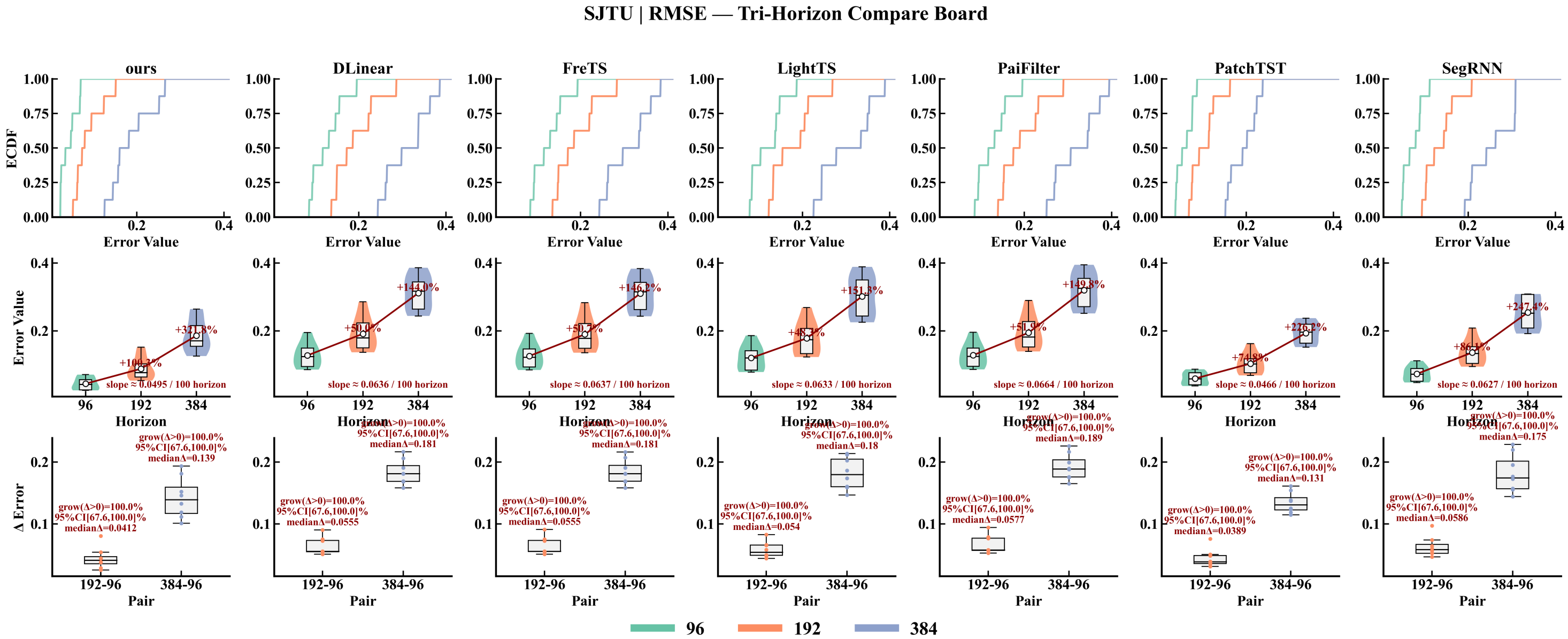
SJTU | RMSE — Tri-Horizon Compare Board
ours
DLinear
FreTS
LightTS
PaiFilter
PatchTST
SegRNN
ECDF
Error Value
Horizon
Δ Error
Pair
slope ≈ 0.0495 / 100 horizon
slope ≈ 0.0636 / 100 horizon
slope ≈ 0.0637 / 100 horizon
slope ≈ 0.0633 / 100 horizon
slope ≈ 0.0664 / 100 horizon
slope ≈ 0.0466 / 100 horizon
slope ≈ 0.0627 / 100 horizon
192-96
384-96
96
192
384

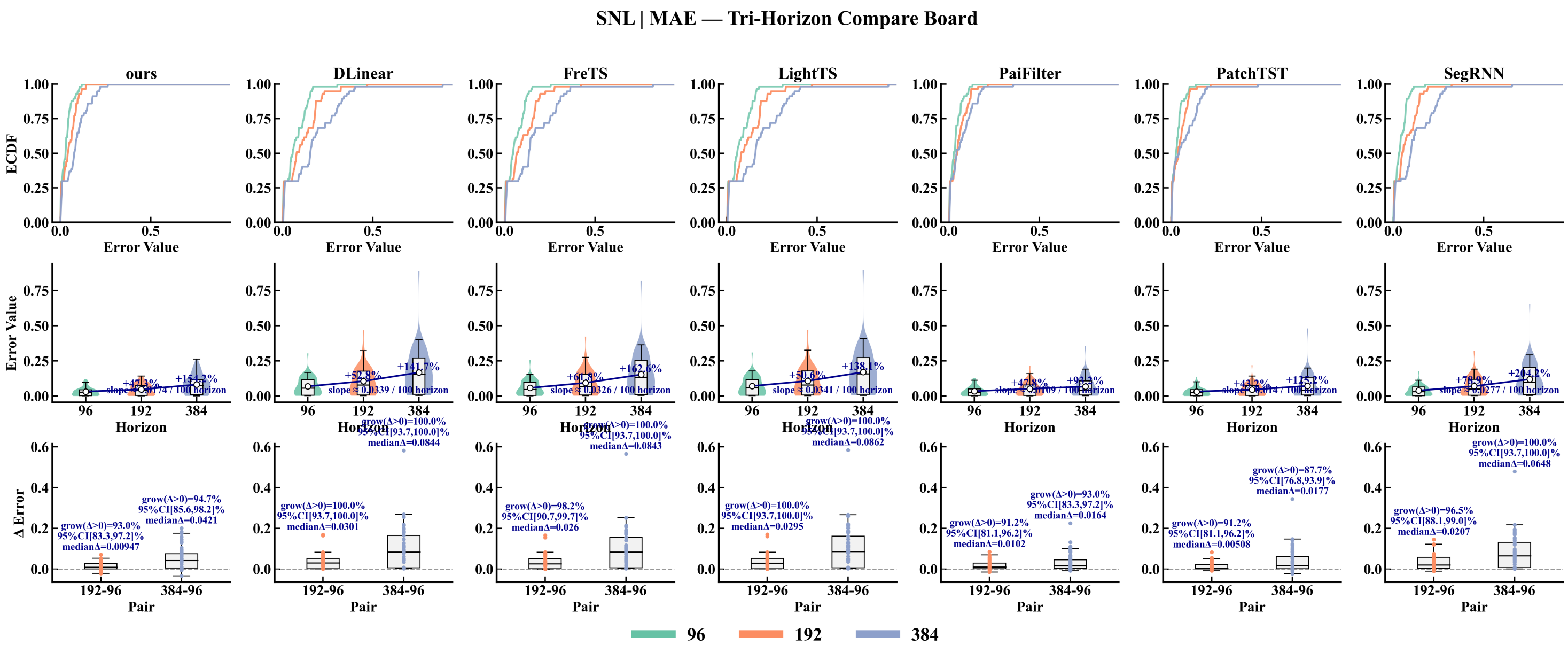
SNL | MAE — Tri-Horizon Compare Board
ours
DLinear
FreTS
LightTS
PaiFilter
PatchTST
SegRNN
ECDF
Error Value
Horizon
Δ Error
Pair
192-96
384-96
96
192
384

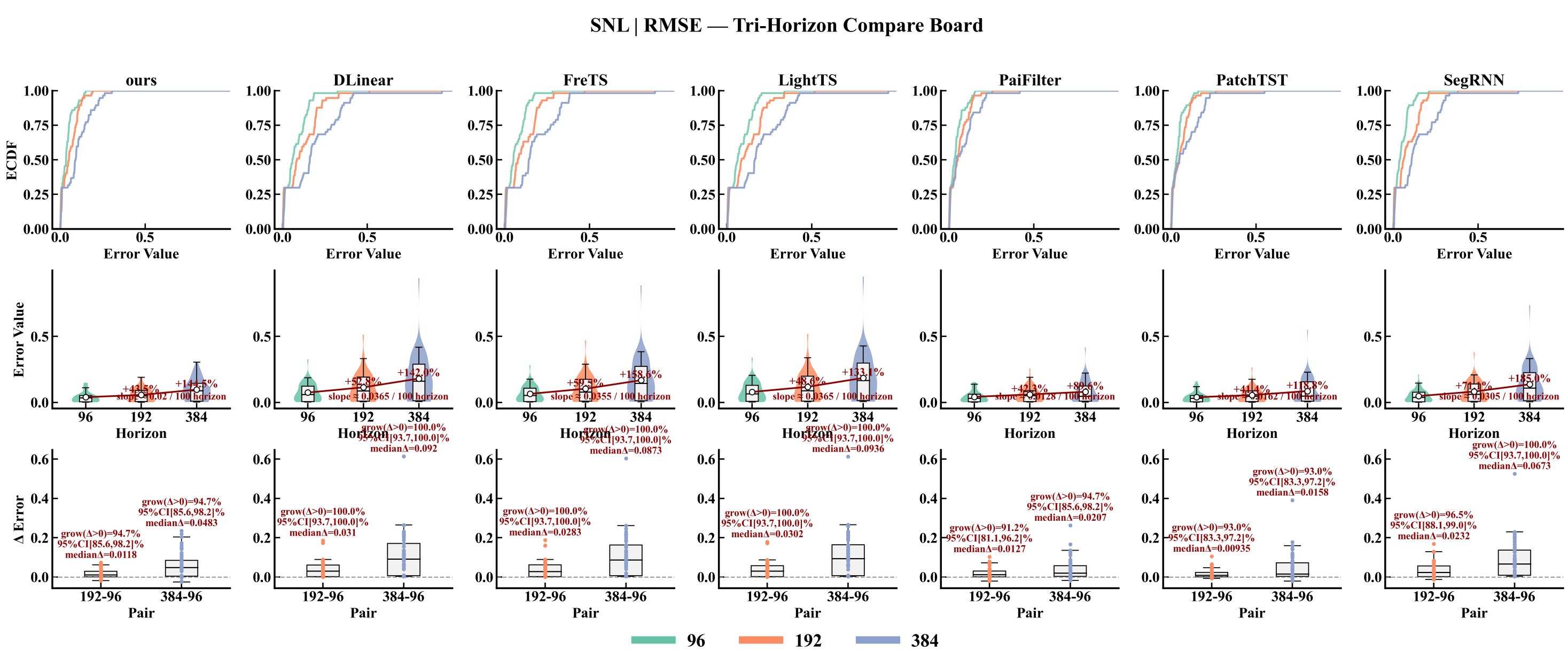
SNL | RMSE — Tri-Horizon Compare Board
ours
DLinear
FreTS
LightTS
PaiFilter
PatchTST
SegRNN
ECDF
Error Value
Horizon
Δ Error
Pair
192-96
384-96
96
192
384

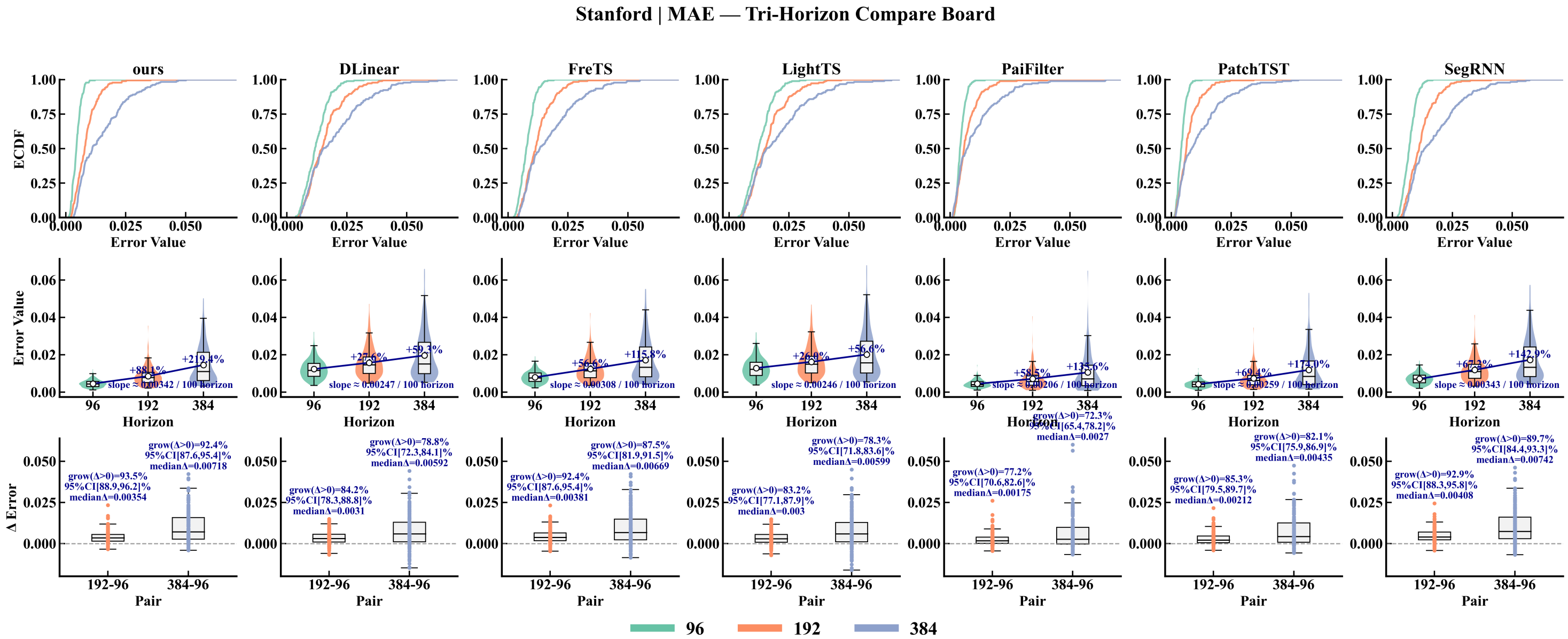
Stanford | MAE — Tri-Horizon Compare Board
ours
DLinear
FreTS
LightTS
PaiFilter
PatchTST
SegRNN
ECDF
Error Value
Horizon
Δ Error
Pair
192-96
384-96
96
192
384

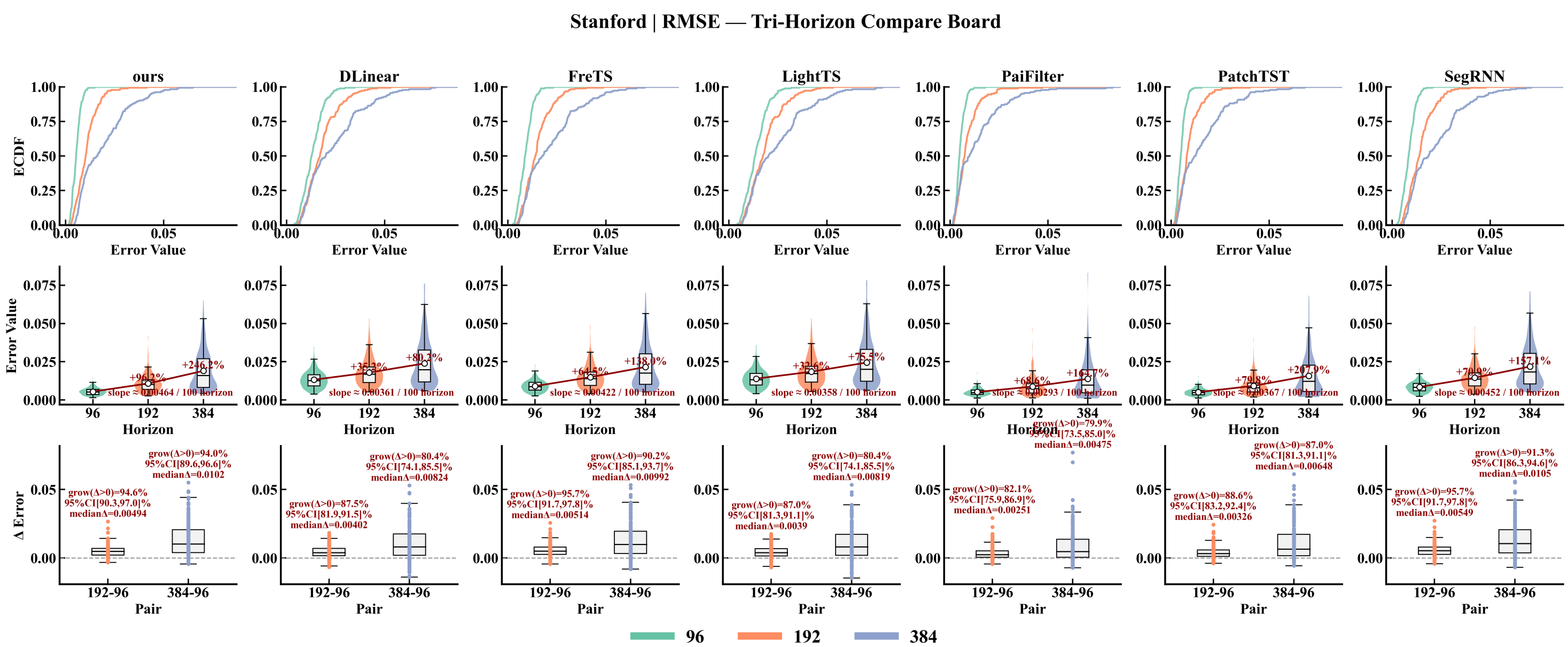
Stanford | RMSE — Tri-Horizon Compare Board
ours
DLinear
FreTS
LightTS
PaiFilter
PatchTST
SegRNN
ECDF
Error Value
Horizon
Δ Error
Pair
192-96
384-96
96
192
384

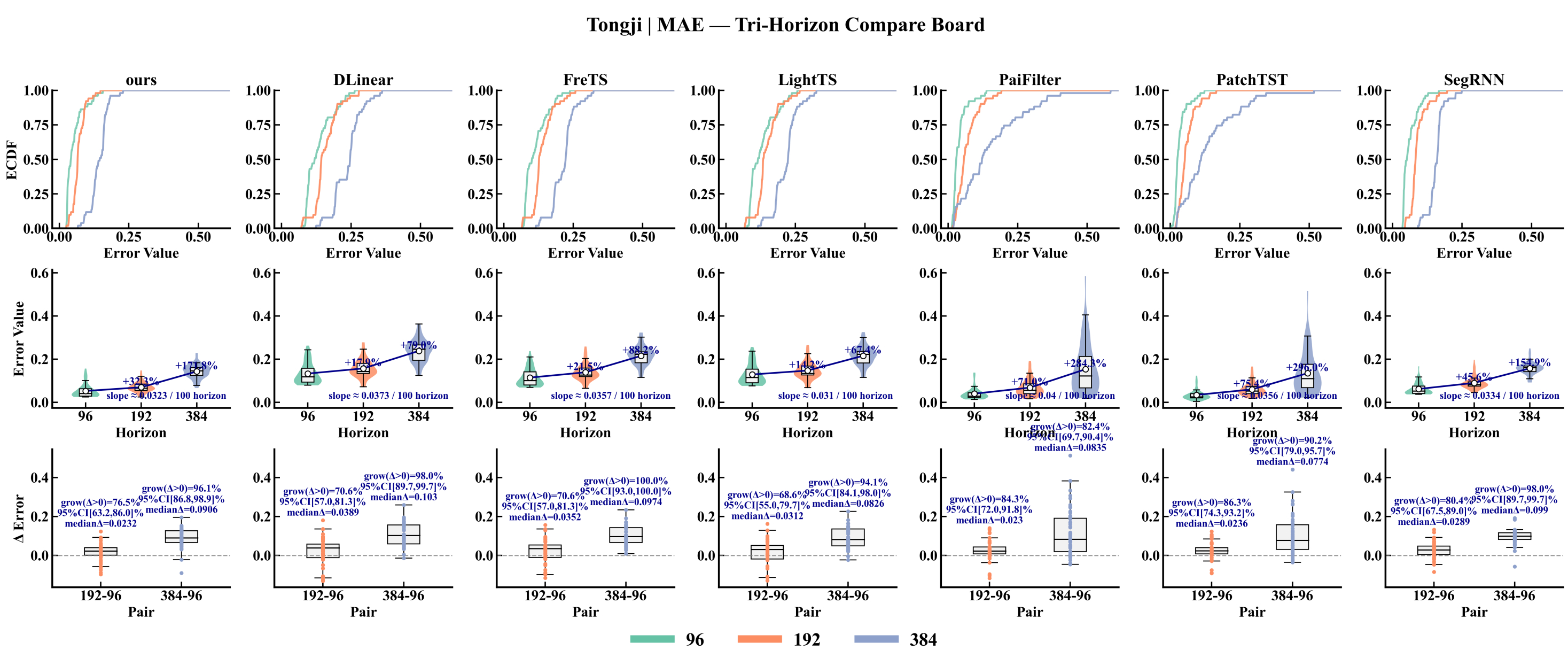
Tongji | MAE — Tri-Horizon Compare Board
ours
DLinear
FreTS
LightTS
PaiFilter
PatchTST
SegRNN
ECDF
Error Value
Horizon
Δ Error
Pair
192-96
384-96
96
192
384

Tongji | RMSE — Tri-Horizon Compare Board

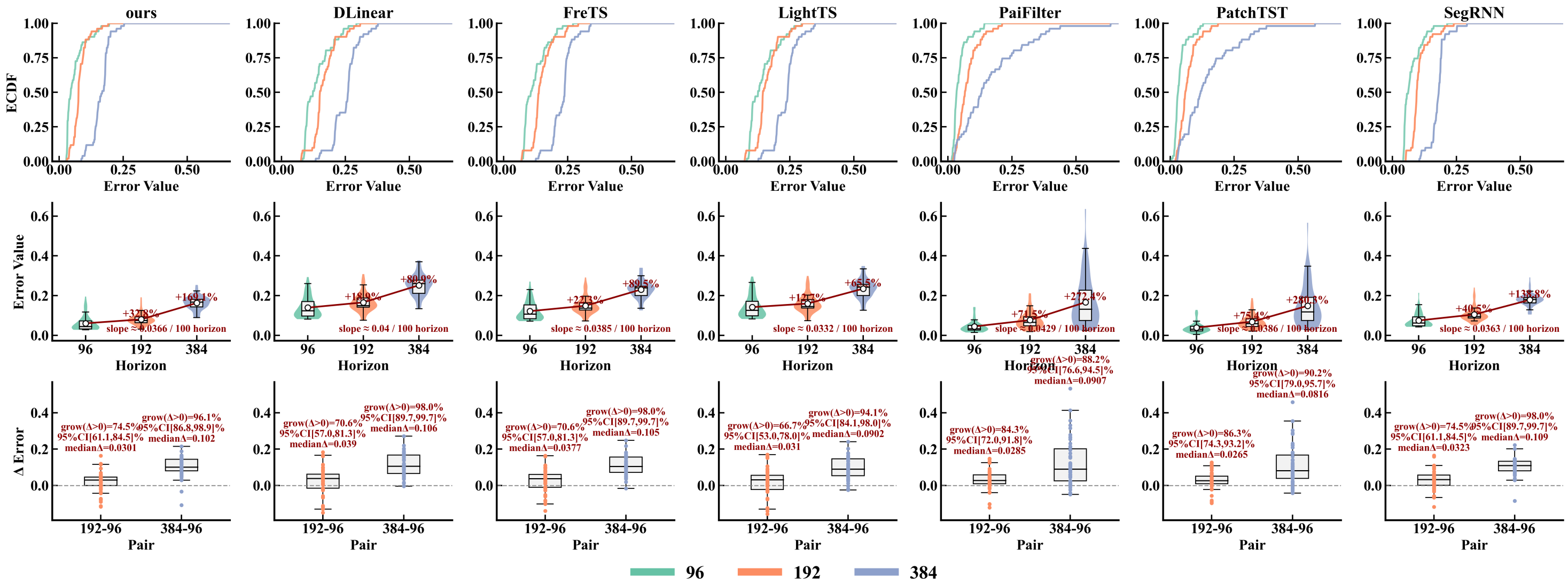

UL-PUR | MAE — Tri-Horizon Compare Board

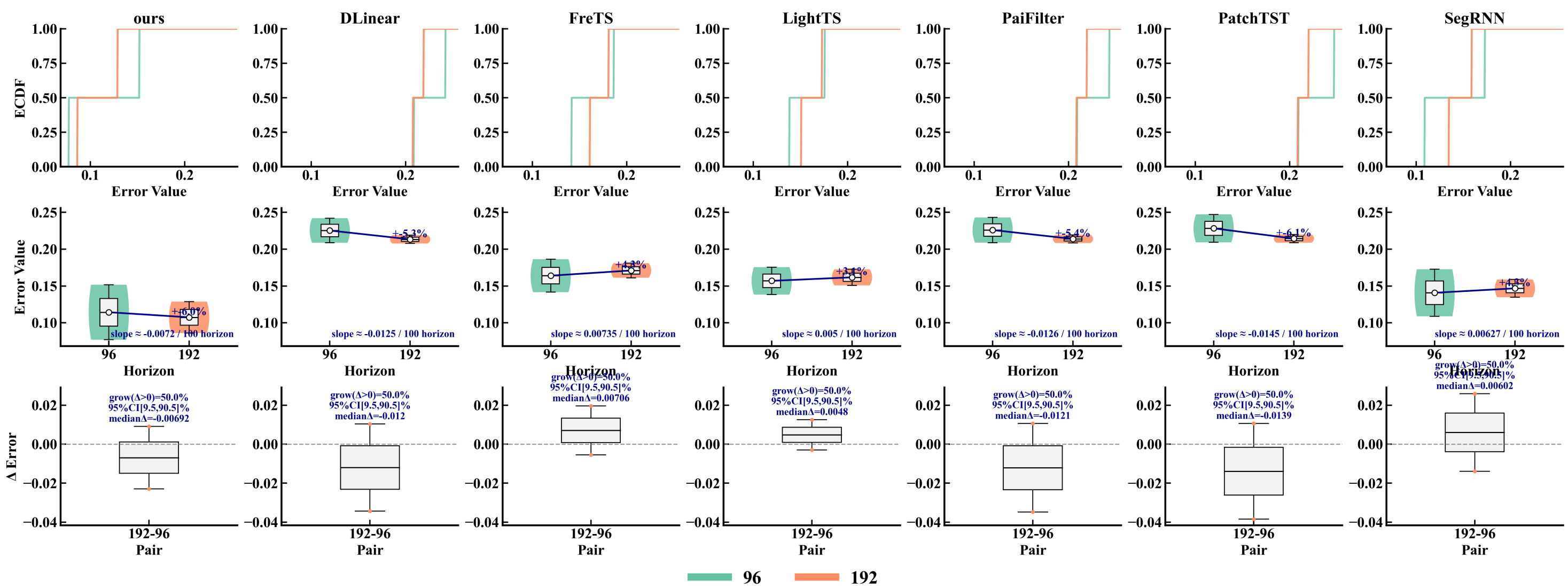

UL-PUR | RMSE — Tri-Horizon Compare Board

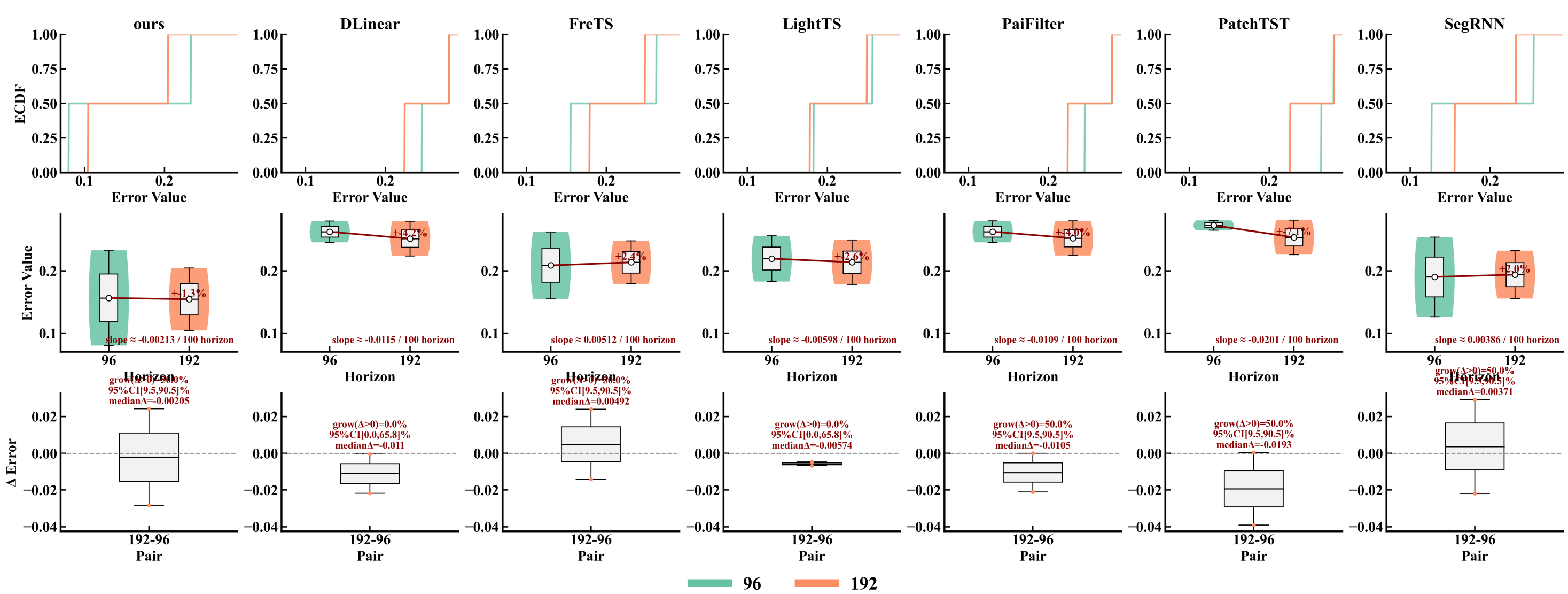

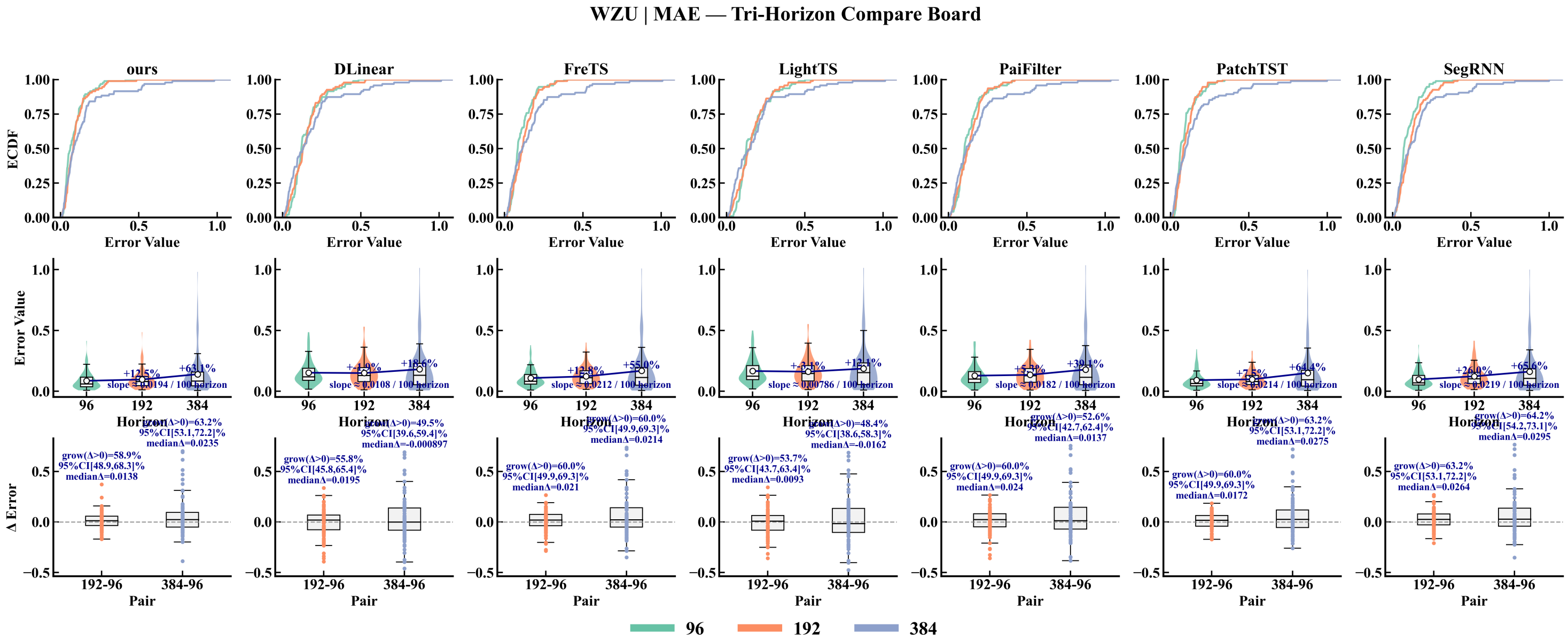
WZU | MAE — Tri-Horizon Compare Board
ours
DLinear
FreTS
LightTS
PaiFilter
PatchTST
SegRNN
ECDF
Error Value
Horizon
Δ Error
Pair
192-96
384-96
96
192
384

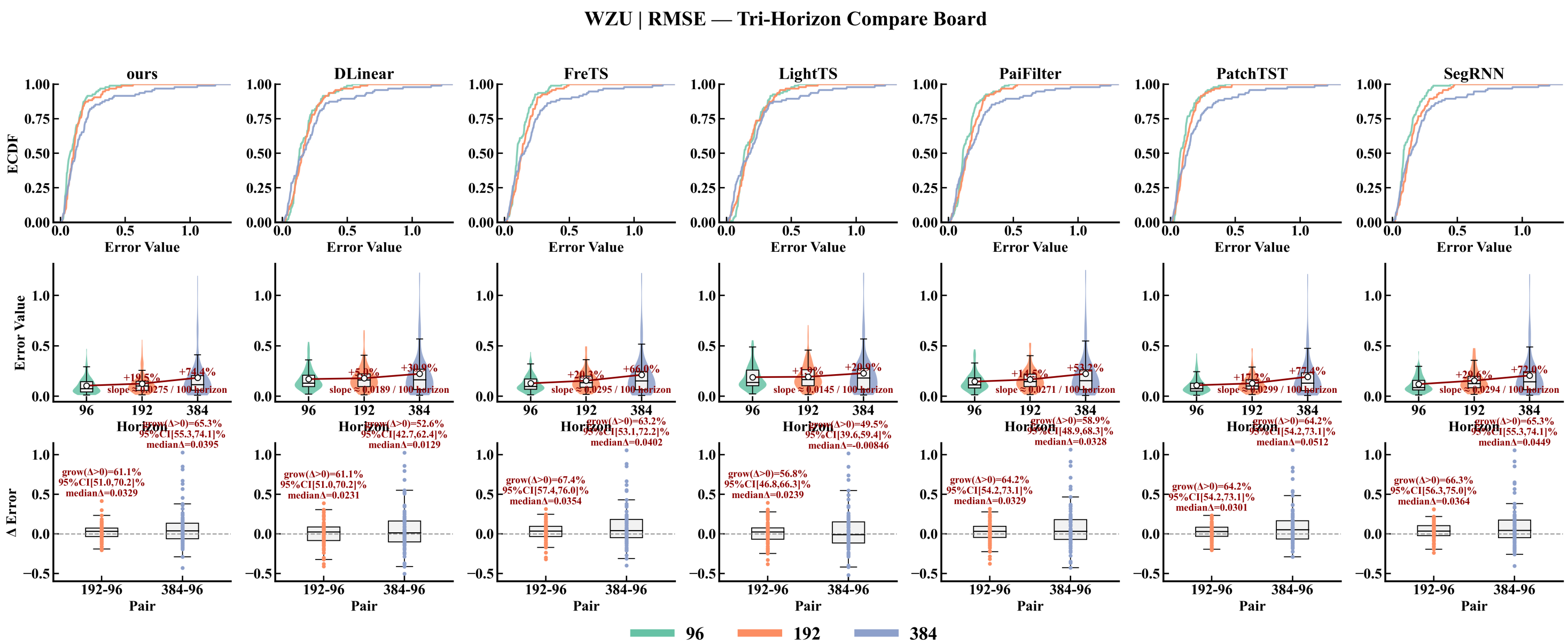
WZU | RMSE — Tri-Horizon Compare Board
ours
DLinear
FreTS
LightTS
PaiFilter
PatchTST
SegRNN
ECDF
Error Value
Horizon
Δ Error
Pair
192-96
384-96
96
192
384

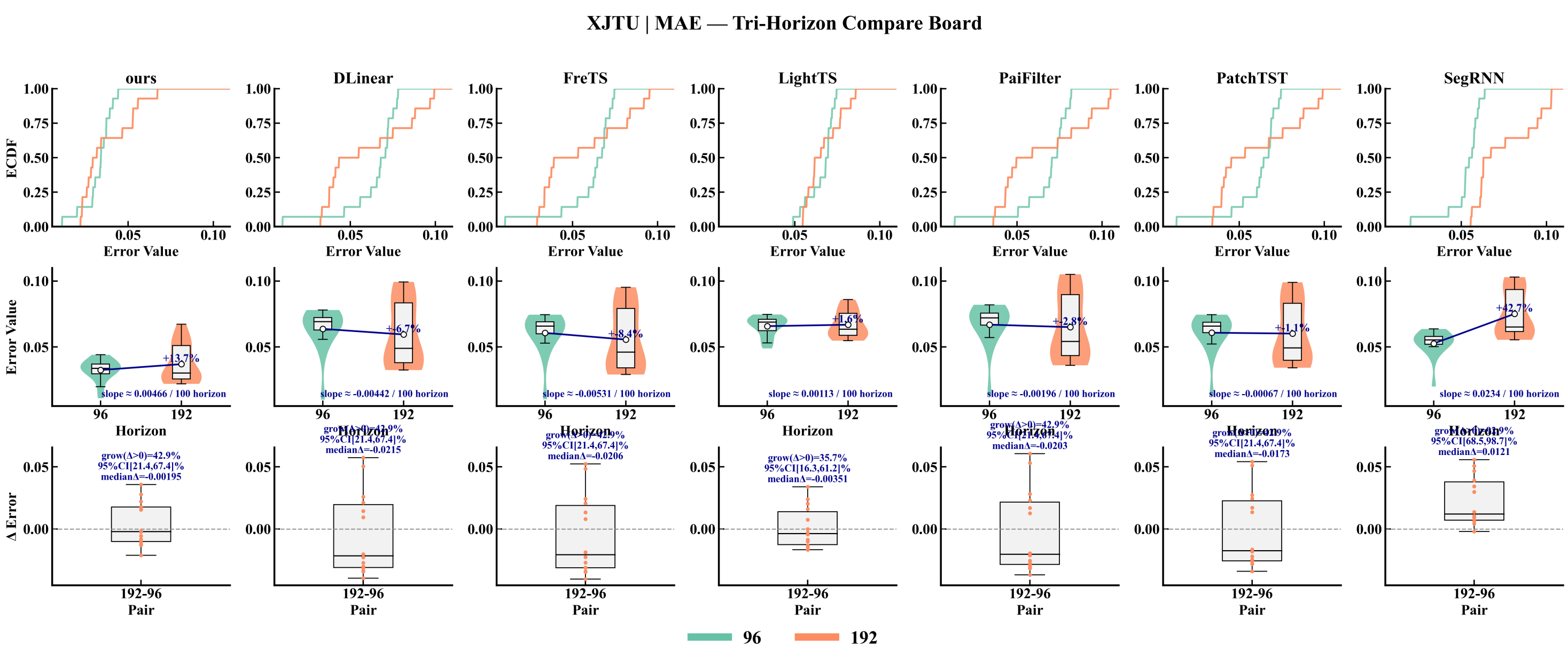
XJTU | MAE — Tri-Horizon Compare Board
ours
DLinear
FreTS
LightTS
PaiFilter
PatchTST
SegRNN
ECDF
Error Value
Horizon
Δ Error
Pair
192-96
96
192

XJTU | RMSE — Tri-Horizon Compare Board

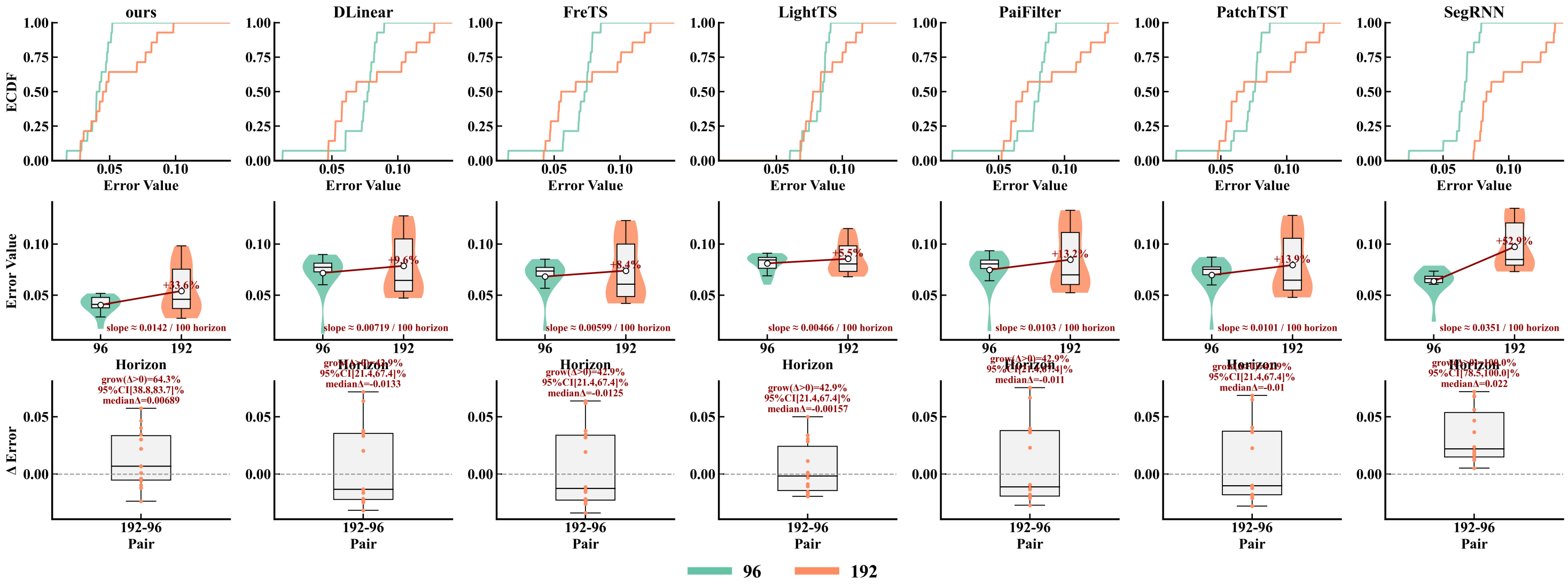

ZN-coin | MAE — Tri-Horizon Compare Board

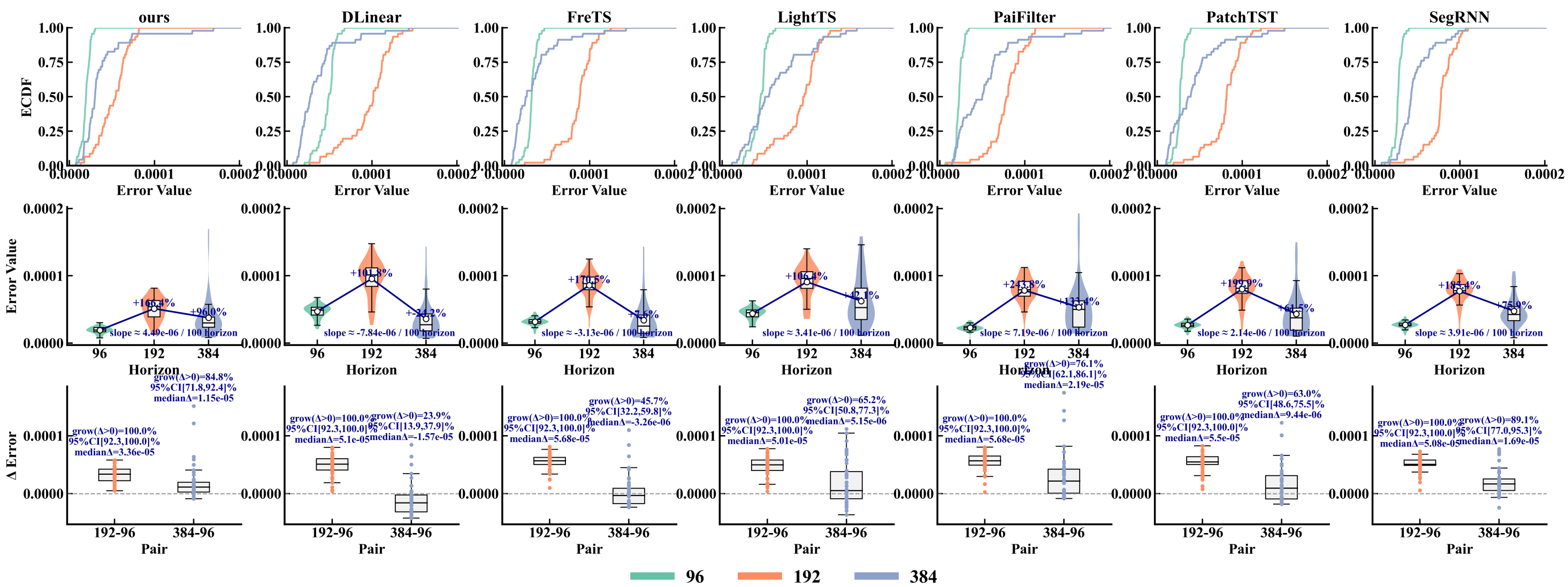

ZN-coin | RMSE — Tri-Horizon Compare Board

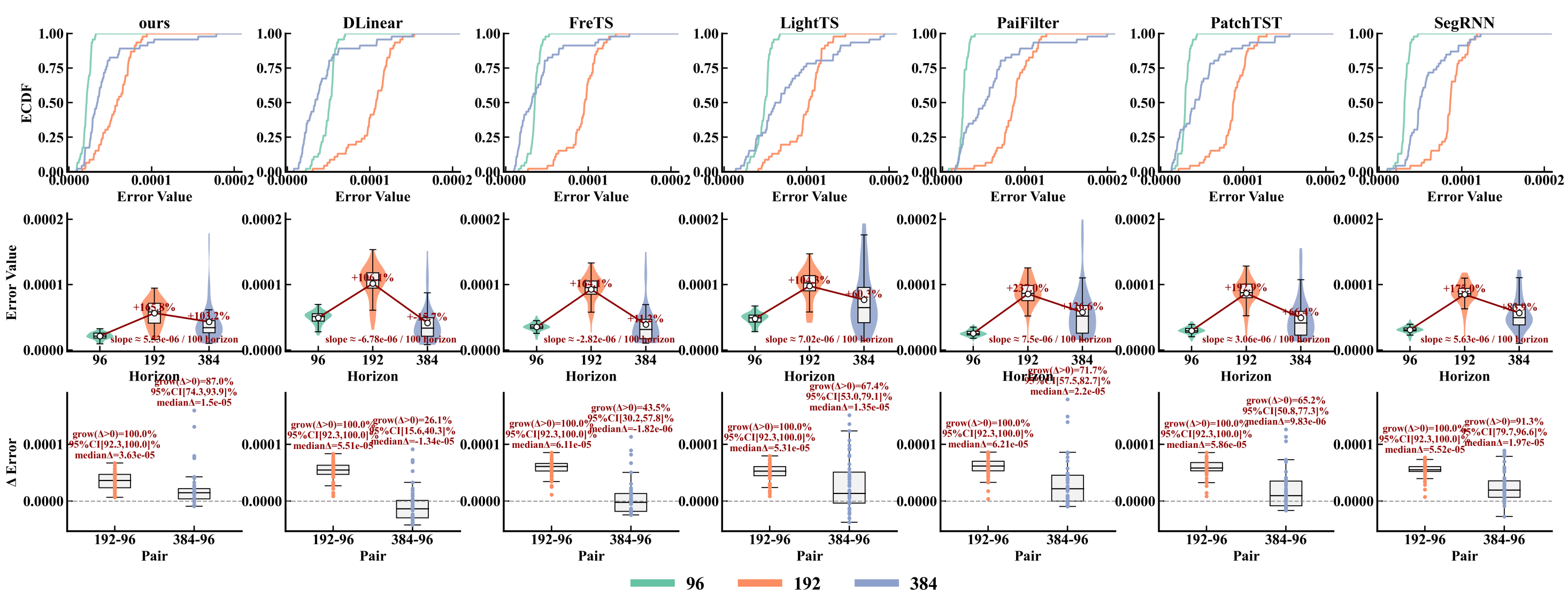

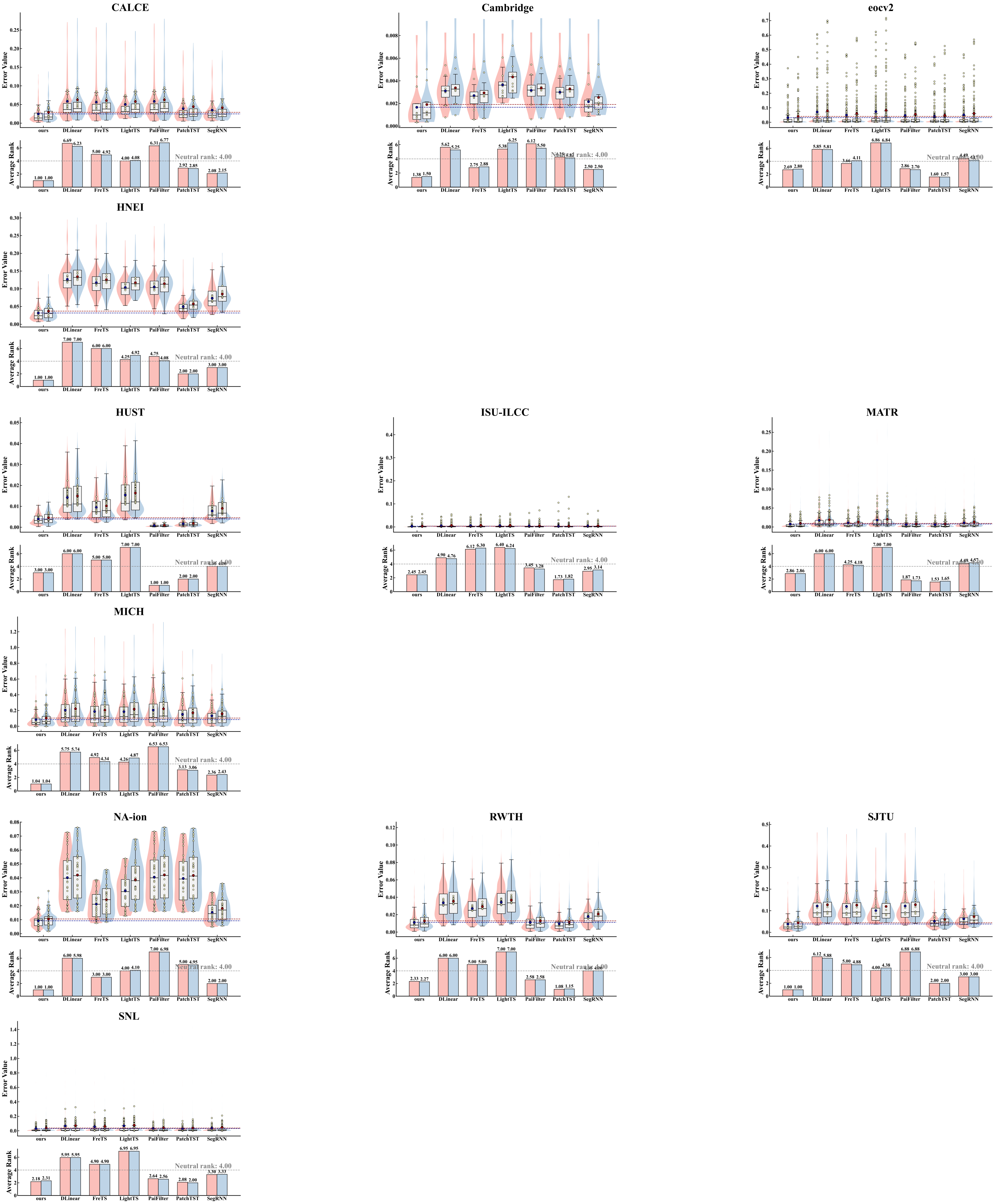
CALCE
Cambridge
eocv2
HNEI
HUST
ISU-ILCC
MATR
MICH
NA-ion
RWTH
SJTU
SNL
Error Value
Average Rank
Neutral rank: 4.00
ours
DLinear
FreTS
LightTS
PaiFilter
PatchTST
SegRNN

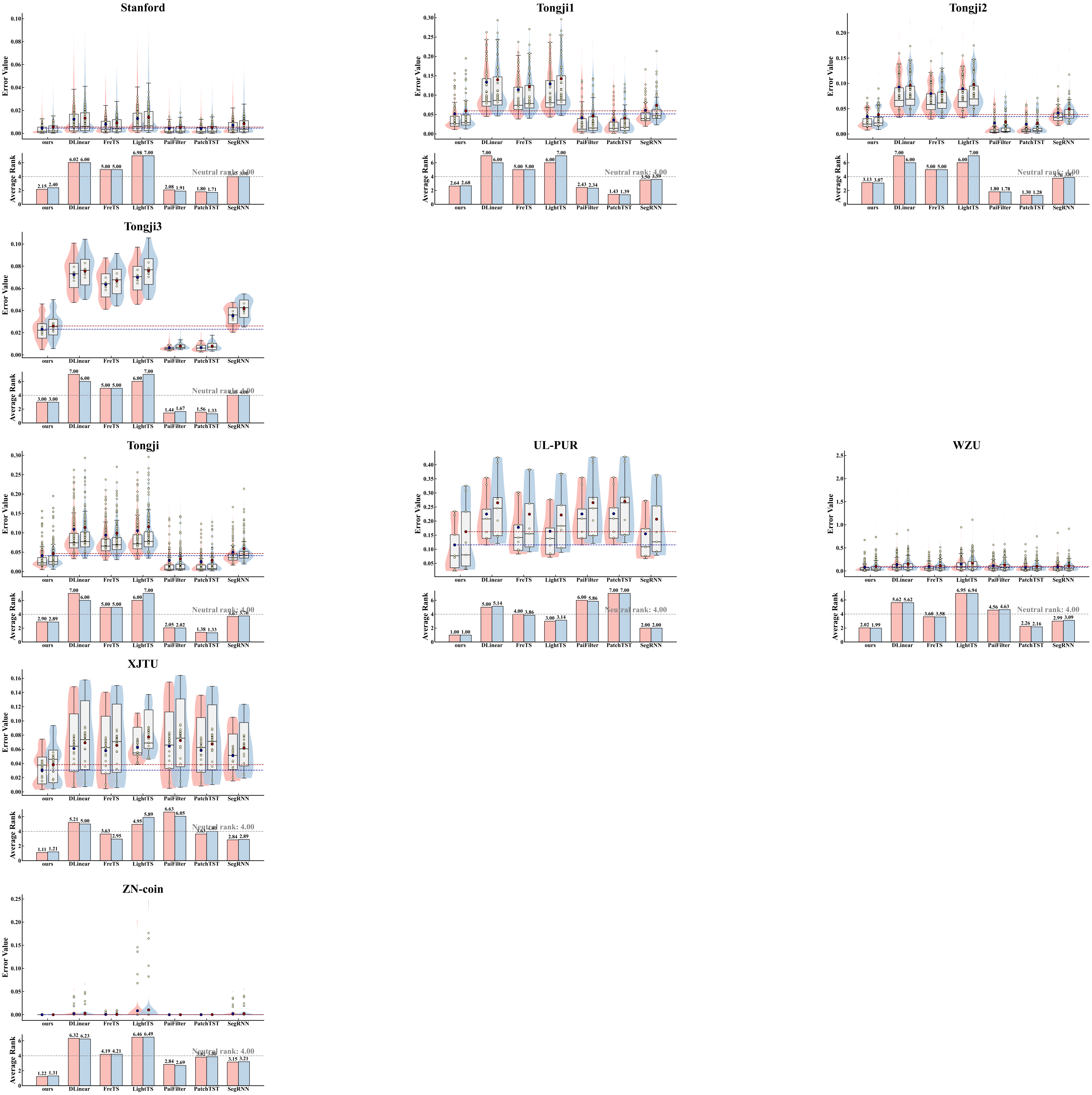
Stanford
Tongji1
Tongji2
Tongji3
Tongji
UL-PUR
WZU
XJTU
ZN-coin
Error Value
Average Rank
Neutral rank: 4.00
ours
DLinear
FreTS
LightTS
PaiFilter
PatchTST
SegRNN

192

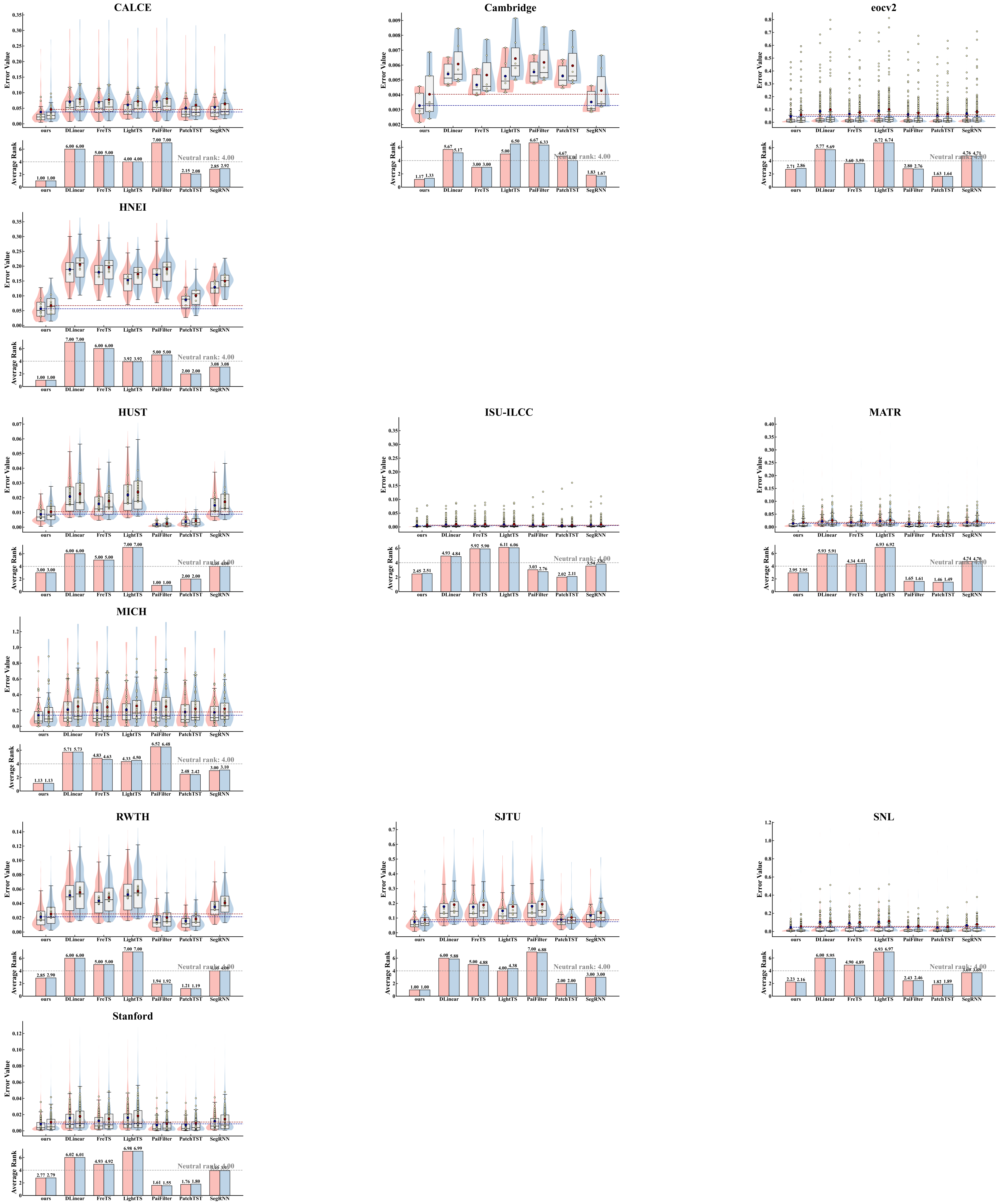
CALCE
Cambridge
eocv2
HNEI
HUST
ISU-ILCC
MATR
MICH
RWTH
SJTU
SNL
Stanford
Error Value
Average Rank
Neutral rank: 4.00
ours
DLinear
FreTS
LightTS
PaiFilter
PatchTST
SegRNN

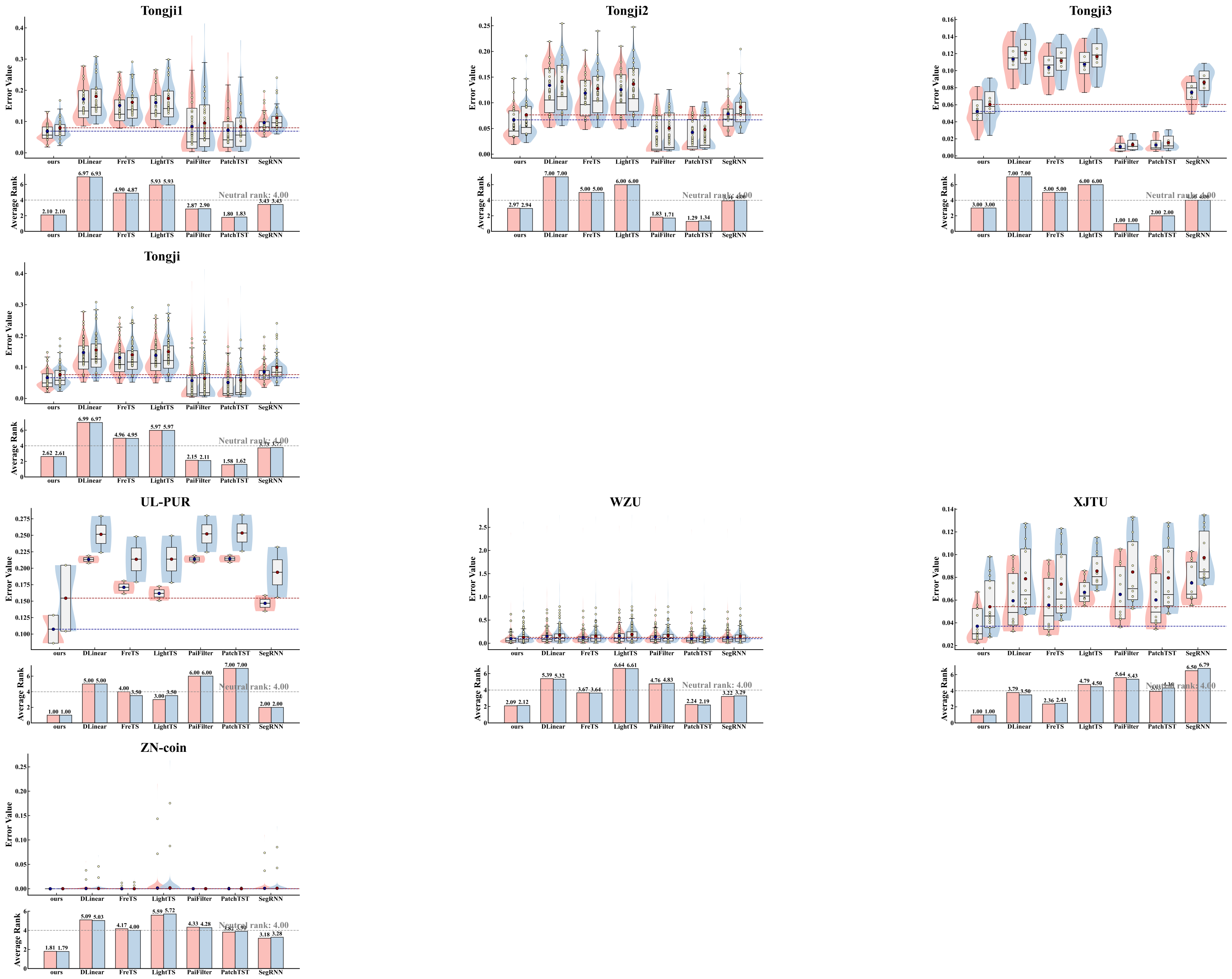
Tongji1
Tongji2
Tongji3
Tongji
UL-PUR
WZU
XJTU
ZN-coin
Error Value
Average Rank
ours
DLinear
FreTS
LightTS
PaiFilter
PatchTST
SegRNN
Neutral rank: 4.00

384

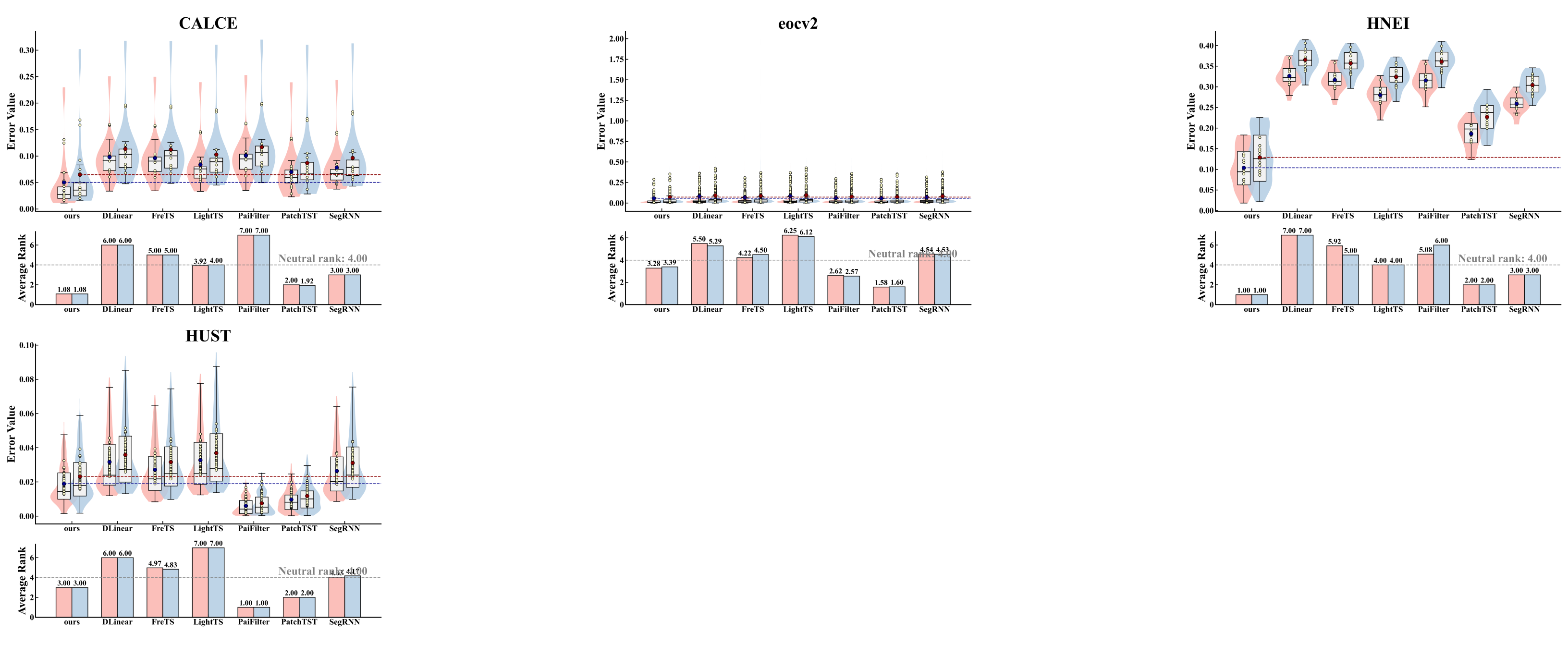
CALCE
eocv2
HNEI
HUST
Error Value
Average Rank
ours
DLinear
FreTS
LightTS
PaiFilter
PatchTST
SegRNN
Neutral rank: 4.00

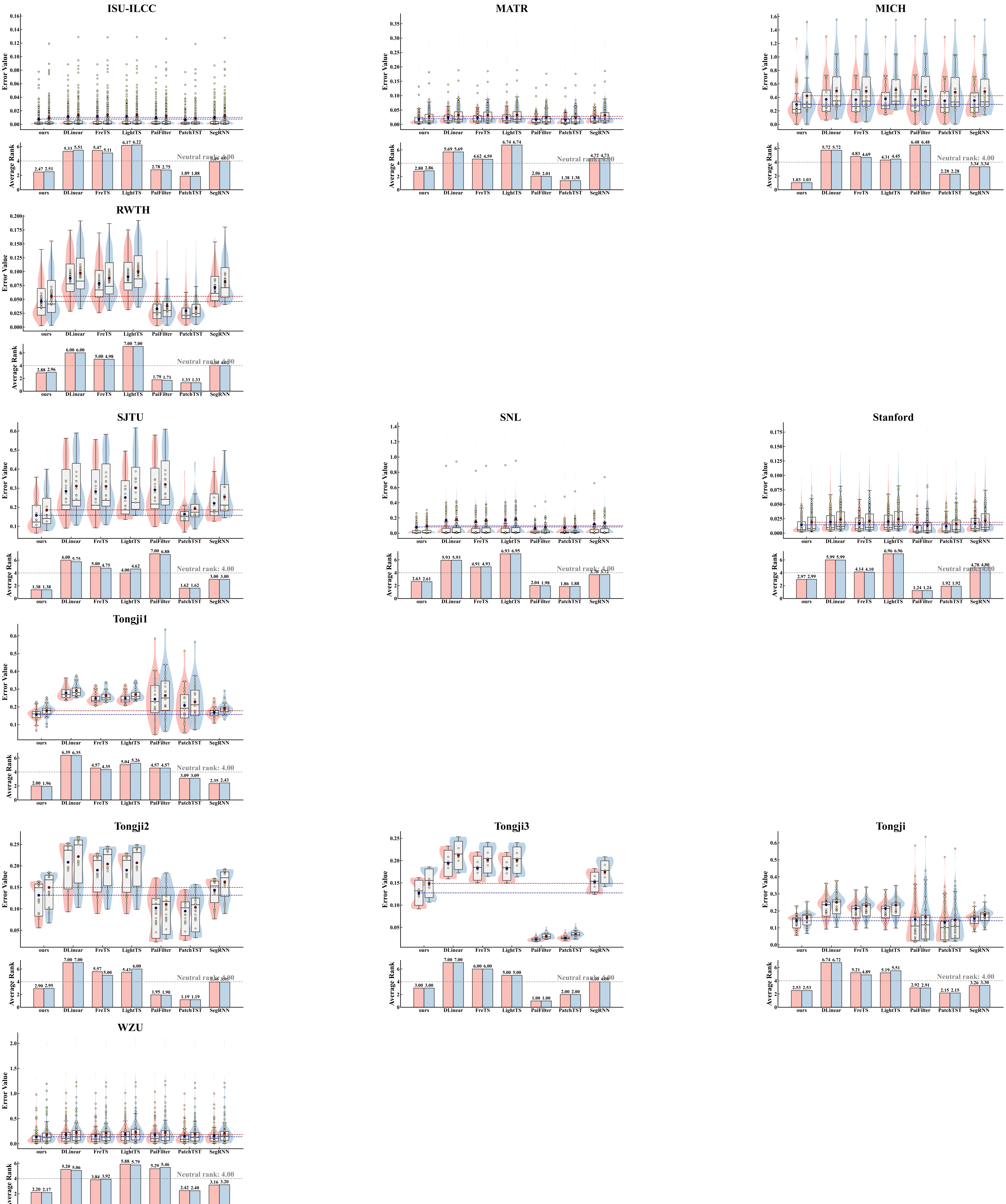

ISU-ILCC
MATR
MICH
RWTH
SJTU
SNL
Stanford
Tongji1
Tongji2
Tongji3
Tongji
WZU
Error Value
Average Rank
Neutral rank: 4.00
ours
DLinear
FreTS
LightTS
PaiFilter
PatchTST
SegRNN

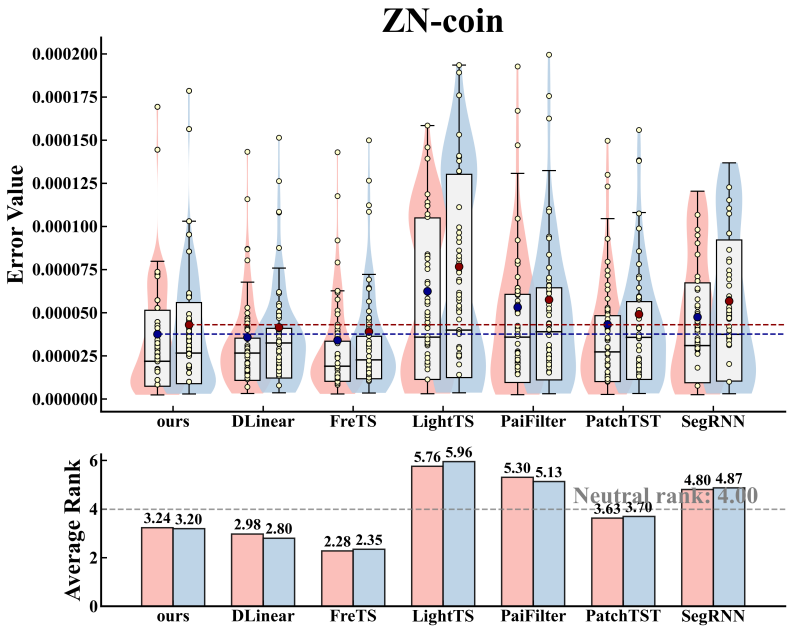
ZN-coin
Error Value
0.000200
0.000175
0.000150
0.000125
0.000100
0.000075
0.000050
0.000025
0.000000
ours
DLinear
FreTS
LightTS
PaiFilter
PatchTST
SegRNN
Average Rank
Neutral rank: 4.00
3.24
3.20
2.98
2.80
2.28
2.35
5.76
5.96
5.30
5.13
3.63
3.70
4.80
4.87
